# Floral infrared emissivity estimates using simple tools

**DOI:** 10.1186/s13007-021-00721-w

**Published:** 2021-02-25

**Authors:** Michael J. M. Harrap, Sean A. Rands

**Affiliations:** University of Bristol, Life Sciences Building, Tyndall Ave, Bristol, BS8 1TQ UK

**Keywords:** Infrared thermography, Thermal imaging, Emissivity, Floral temperature, Angiosperms

## Abstract

**Background:**

Floral temperature has important consequences for plant biology, and accurate temperature measurements are therefore important to plant research. Thermography, also referred to as thermal imaging, is beginning to be used more frequently to measure and visualize floral temperature. Accurate thermographic measurements require information about the object’s emissivity (its capacity to emit thermal radiation with temperature), to obtain accurate temperature readings. However, there are currently no published estimates of floral emissivity available. This is most likely to be due to flowers being unsuitable for the most common protocols for emissivity estimation. Instead, researchers have used emissivity estimates collected on vegetative plant tissue when conducting floral thermography, assuming these tissues to have the same emissivity. As floral tissue differs from vegetative tissue, it is unclear how appropriate and accurate these vegetative tissue emissivity estimates are when they are applied to floral tissue.

**Results:**

We collect floral emissivity estimates using two protocols, using a thermocouple and a water bath, providing a guide for making estimates of floral emissivity that can be carried out without needing specialist equipment (apart from the thermal camera). Both protocols involve measuring the thermal infrared radiation from flowers of a known temperature, providing the required information for emissivity estimation. Floral temperature is known within these protocols using either a thermocouple, or by heating the flowers within a water bath. Emissivity estimates indicate floral emissivity is high, near 1, at least across petals. While the two protocols generally indicated the same trends, the water bath protocol gave more realistic and less variable estimates. While some variation with flower species and location on the flower is observed in emissivity estimates, these are generally small or can be explained as resulting from artefacts of these protocols, relating to thermocouple or water surface contact quality.

**Conclusions:**

Floral emissivity appears to be high, and seems quite consistent across most flowers and between species, at least across petals. A value near 1, for example 0.98, is recommended for accurate thermographic measurements of floral temperature. This suggests that the similarly high values based on vegetation emissivity estimates used by previous researchers were appropriate.

## Background

The temperature of plants has a strong influence on their biology [1–5], and this is particularly true in the case of floral temperature [6]. Floral temperature influences floral metabolism [7–9], development [10, 11], pollen viability [12, 13] and transpiration [14, 15]. It also plays a role in how flowers interact with other organisms, acting as a floral reward for pollinators [16–20] and a floral cue influencing pollinator innate preferences [21–24] and learning [25–30]. However, floral temperature can become a deterrent to flower visitors when flowers overheat [22, 31] and, floral temperature can influence floral susceptibility to disease [32–34]. Consequently, accurate measurement of floral temperature is key to botanical research [6] and may have important applications in horticultural industries [33–36], particularly in monitoring the risk of plant disease and in monitoring the quality of floriculture produce.

Infrared thermography (also called thermal imaging) has been utilised on several occasions to study floral temperature (e.g. [23, 24, 28, 34, 37–43]). As thermography is non-contact, highly responsive, and allows simultaneous measurements of temperature across a target, it has many advantages over other methods of measuring floral temperature. Thermography works on the following principles. All objects hotter than absolute zero produce thermal infrared radiation [44, 45]. Thermal cameras measure this radiation and use this, along with thermography parameters that are input into the camera, to estimate the temperature of an object [46–49]. The most important of these input thermography parameters is the emissivity of the thermography target. Emissivity refers to the object’s capacity to radiate thermal infrared radiation relative to a black body of equal temperature, where the black body is a theoretical body which is non-transmissive and non-reflective, and thus completely absorbs any kind of incident electromagnetic radiation. Emissivity is represented as a fraction between 0 and 1, with black bodies having an emissivity of 1. Accurate thermographic temperature estimates depend on accurate estimates of all these thermography parameters, particularly emissivity. Inaccurate estimates of target emissivity would lead to inaccurate thermographic temperature estimates. As the relationship between temperature and radiation is non-linear [46], the extent of temperature measurement error as a result of emissivity error depends on the true emissivity of the target and the extent of inaccuracy of the emissivity value used.

Despite the importance of floral temperature to the biology of plants and increasing use of thermal cameras to study floral temperature, there are no published estimates of the emissivity of floral tissues [49]. Floral emissivity estimates are required to ensure that appropriate values are chosen for thermographic floral temperature measurements, use of more accurate emissivity values will also ensure a greater level of accuracy and confidence in the thermographic measurements based on these values. When emissivity values for the target itself are not available, and measurement of the target emissivity impossible or not practical, it is best practice to select emissivity values from a similar target. Consequentially, previous floral thermography studies (e.g. [23, 28, 34, 37–43]) have used emissivity estimates made of vegetative tissues of plants (primarily leaves), frequently those made by Gates [50], Idso et al. [51], Rubio et al. [52] and López et al. [53]. Emissivity values used for thermography of vegetation are on average 0.957 ± 0.038 (mean ± SD), but range from 0.8 to 1 [49]. Use of these vegetative emissivity values in floral thermography assumes that floral tissues have the same emissivity as whichever vegetative tissues are used for those estimates. While floral tissue is likely to have a similar emissivity to vegetative plant tissues (and most organic tissues), the emissivity of a given object can vary depending on the composition (the chemical makeup of the surface), geometry (the three-dimensional shape, particularly its convexity and concavity) and surface texture (the fine scale deviation from a flat surface, such as ridges, also known as surface ‘finish’ or ‘roughness’) of the object [47, 48]. Differences in emissivity have been observed between different vegetative plant tissues, such as the adaxial and abaxial leaf surface, and between-species differences in vegetative tissue emissivity have been recorded [49, 52–54], hence the range of emissivity values seen across plant vegetative tissues. Floral epidermal surfaces differ from those of vegetative plant tissues in the coverage and chemical composition of cuticle waxes, as well as their epidermal cell shape and thus surface texture [55–58]. Flowers also have more complicated morphologies [59, 60], that is their three-dimensional shape or geometry, when leaves tend to be largely flat. As floral tissues differ from vegetative tissues in these characteristics, it is possible that floral emissivity differs from that of many vegetative plant tissues. Without emissivity estimates made on floral tissue, it is unclear how accurate emissivity values chosen based on estimates of vegetative plant tissues are to floral tissues. Inaccurate emissivity values will have consequences for the accuracy of temperature measurements and potentially the validity of findings of those studies [49]. If emissivity values chosen based on vegetative tissue estimates are appropriate for floral tissue, obtaining floral tissue emissivity estimates would confirm these choices and improve our confidence in previous floral thermography work. Furthermore, the flowers of different species can differ in surface texture [61–66] and flowers show a wide diversity in morphology and geometry [59, 60]. It is also likely that the between-species differences in epidermal chemical composition seen in vegetative tissues [55–58, 67, 68] also occur between the floral tissue of different species. This may mean floral emissivity varies between different flower species. Additionally, many flower species show changes in surface textures across their flowers, particularly across petal surfaces [61–66]. Geometry of the floral surface also differs greatly between floral reproductive structures, which typically have protruding structures, and petals, which are comparably flat [59, 60]. It is therefore possible that there are also differences in emissivity between different locations on flowers. Estimates of floral emissivity as well as information on how it varies, both from other vegetative plant tissues as well as within and between flower species, represents an important unknown with consequences on the accuracy of thermographic floral temperature measurements. To ensure we have the required information to conduct accurate floral thermography, we require estimates of floral emissivity taken across species and at different locations within flowers.

The simplest methods for measuring the emissivity of a material is to thinly coat part of the material with something of a known emissivity and then heat the object up evenly [47, 69, 70]. This known emissivity coating should ideally be of a higher emissivity (such as electrical tape, or emissivity-controlled paint), which allows temperature measurements to be taken more easily. This coating should be thin to ensure it heats up evenly with the target object, additionally applying certain coatings too think or unevenly might influence their surface roughness and thus emissivity. Elevated temperature is advised for emissivity measurements as the relationship between radiation and temperature is made clearer at higher temperature [44–46]. Using this method, a true temperature estimate can be made using the coated area and the known emissivity value. If the object is evenly-heated, emissivity can then be adjusted until a matching temperature estimate is achieved on the uncoated region or resolved based on the apparent temperature of that region [47], where the apparent temperature is the estimate made by the thermal camera before any adjustments for emissivity, reflections or the sampling environment are applied. However, coating and evenly-heating flowers can be difficult due to their structural complexity, the ease with which they can be damaged and, frequently, their small size. These difficulties in applying normal protocols for emissivity measurements to flowers perhaps explains why such measurements do not appear to have been carried out. Alternative protocols need to be used to estimate floral emissivity [52, 53, 71–74]. Ideally these alternative protocols should be suitable for all flower species and not require specialist tools or setups (apart from a thermal camera), so they can be more easily repeated by a wide range of researchers on different study species.

In this study we evaluate two methods for obtaining rough estimates of floral emissivity, adapting alternative methods of emissivity estimation applied to other targets [53, 71, 72]. The two protocols are used to collect thermal images of flowers at a known temperature, and from these emissivity estimates can be obtained to inform parameter choices. Additionally, we demonstrate two ways by which emissivity estimates can be resolved from data extracted from these thermal images: calculating emissivity or resolving emissivity with *Solver* software. We demonstrate these methods, obtaining floral emissivity estimates from twelve different species (four by both methods) with varied flower forms. Estimates collected by these methods are used to evaluate these protocols, and to assess floral emissivity, as well as its variation across flower species and between structures of the flower. This allows us to provide recommendations for emissivity values to use for floral thermography measurements of flowers, as well as identify suitable protocols for estimation of floral emissivity. This information allows a more informed choice of floral emissivity, based on our estimates made on floral tissue, and facilitates collection of floral emissivity estimates on specific targets by other researchers. This will improve confidence in emissivity values chosen for floral thermography as well as the accuracy of floral thermography measurements in future botanical studies and monitoring schemes. Obtaining emissivity values for floral tissue additionally allows us to assess how appropriate the vegetation emissivity values used by past studies are, and what impact that choice has on the findings of those studies.

## Methods

### Emissivity resolution

In this section we will discuss what information is required for emissivity estimation at a given point and how emissivity can be resolved if this information is known. In the sections following this we will cover the two protocols by which we obtain this information for floral emissivity estimates, the ‘thermocouple’ and ‘water bath’ protocols. The total thermal infrared radiation entering the thermal camera relates to the target object’s temperature and its emissivity by the following relationship:

1 $$ W\,=\,\varepsilon \cdot \sigma \cdot \rho \cdot \left( {T_{obj} } \right)^{4} + \left( {1 - \varepsilon } \right) \cdot \sigma \cdot \rho \cdot \left( {T_{ref} } \right)^{4} + \sigma \cdot \left( {1 - \rho } \right) \cdot \left( {T_{env} } \right)^{4} . $$

In Eq. 1, *W* is the total radiation entering the camera. *ε* is emissivity, *σ* is the Stefan Boltzmann constant (c. 5.7 × 10^−8^ W m^−2^ K^−4^), and *ρ* is transmissivity of the air between the camera and target. Transmissivity is normally calculated as a function of relative humidity, air temperature, and distance between the camera and target [46, 49, 75]. All temperatures presented in Eq. 1 are in Kelvin. *T*_*obj*_ is the temperature of the object being imaged, *T*_*ref*_ is the reflected temperature of the object being imaged, and *T*_*env*_ is the air temperature of its environment. Each of the parameters and terms relating to Eq. 1 are described in further detail elsewhere [46–49]. Equation 1 assumes the target object is non-transmissive to thermal infra-red radiation, which is true of most biological targets [46–49]. *W* can be calculated from the apparent temperature, *T*_*app*_, where

2 $$ W = \sigma \cdot \left( {T_{app} } \right)^{4} . $$

Apparent temperature is the temperature estimated by a thermal camera before any adjustments for emissivity, reflections or the camera’s environment are applied. Apparent temperature of a thermal image can be obtained by setting emissivity to 1 and distance to 0 (so that *ρ* = 1), which removes these adjustments.

If all other values across Eqs. 1 and 2 are known for a given point, we can estimate emissivity, *ε*. Emissivity estimates can be resolved for a given measurement location by calculation or using *Solver* software. A calculated emissivity estimate can be found by rearranging Eq. 1:

3 $$ \varepsilon = \frac{{W - \sigma \cdot \rho \cdot \left( {T_{ref} } \right)^{4} - \sigma \cdot \left( {1 - \rho } \right) \cdot \left( {T_{env} } \right)^{4} }}{{\sigma \cdot \rho \cdot \left( {T_{obj} } \right)^{4} - \sigma \cdot \rho \cdot \left( {T_{ref} } \right)^{4} }} . $$

Alternatively, *Solver* software can be used to find the best value for emissivity given the known values. This *Solver* estimation of emissivity is carried out by finding the value of *ε* that allows Eq. 1 to best estimate the observed value of *W* calculated using thermal camera measurements and Eq. 2. To find this value of *ε* we must calculate *W* in two ways: *W*_*exp*_, the value of *W* that is expected knowing *T*_*obj*_ and for a given value of *ε*, calculated as in Eq. 1; and *W*_*obs*_, the observed *W* calculated using *T*_*app*_ measurements made with the thermal camera and Eq. 2. The value of emissivity where *W*_*exp*_ = *W*_*obs*_ is the emissivity of the target object, as it predicts the correct amount of radiation measured by the thermal camera given the known temperature of the object (*T*_*obj*_). For the estimates below, the value of emissivity used to calculate *W*_*exp*_ was varied using *Microsoft Excel Solver* (in Excel for Office 365, Microsoft Corporation, Redmond, USA) to find the values of *ε* that resulted in the sum *∆W* across all measurement points having the value closest to zero, where

4 $$ \Delta W = \sqrt {(W_{obs} - W_{exp} )^{2} } , $$

at each measurement point. These values of *ε* are our ‘*Solver* emissivity estimates’. The initial value of *ε* for *W*_*exp*_ calculation given to *Excel Solver* (i.e. the value which *Excel Solver* varies *ε* from to find the best fitting *ε*) was 0.98, a value frequently used in floral thermography [49]. As floral emissivity was likely to be in this region this choice reduced *Solver* processing time. To meet constraints on the number of values *Excel Solver* can adjust at once, solutions were carried out in groups of 100 measurement points. *Solver* solutions used the default *Excel Solver* settings (the GRG Nonlinear solving method with a constraint precision set at 10^–6^), and no further limits were applied to *Excel Solver*.

It is worth highlighting that both the protocols of resolving emissivity allow emissivity to have a values greater than 1 or less than 0, the theoretical limits of emissivity [44–49]. If all parameters required for emissivity estimation were measured perfectly, estimates should remain within these bounds. Estimates outside these bounds may occur due to a combination of, the ‘true’ emissivity value being close to 0 or 1, and measurement errors in the values of Eqs. 1 and 2 (as observed previously in [53, 71, 76]). We chose not to limit values to between 0 and 1, this is in line with previous emissivity estimation studies [53, 71], as doing so may conceal the extents of error in protocols. For example, if bounds were applied an (otherwise) excessive overestimate or underestimate might still read as 1 or 0 respectively, potentially concealing a larger inaccuracy and apparently achieving the same result as an (otherwise) very slight overestimate. Thus, imposing bounds may lead to incorrect appraisal of emissivity estimation protocols or, at least, complicates this aim of the present study. Furthermore, such bounds may create skew in data distributions, increasing complexity of required statistical analyses.

If one were to use coating methods of emissivity estimation described in the previous section, *T*_*obj*_ is the temperature of the coated area (which can be accurately measured as emissivity of the coating is known). Assuming even heating of the target, *T*_*app*_ can also be obtained from the uncoated area. Other parameters in Eqs. 1, 2 and 3 can be measured alongside *T*_*obj*_ and *T*_*app*_ allowing emissivity estimation. *T*_*ref*_ can be measured using a multidirectional mirror (best practice, where it is taken to be the average apparent temperature of the mirror) or taken to equal *T*_*env*_ [49]. *T*_*env*_, relative humidity and distance between the camera and the target can be measured using appropriate measurement tools (for example: a thermometer, hygrometer, and ruler respectively). As flowers can often be unsuitable for this method, other protocols to obtain *T*_*obj*_ and *T*_*app*_ are required. In the following sections we shall describe two protocols by which these parameters are measured for flowers.

### Thermocouple protocol

**Fig. 1:**
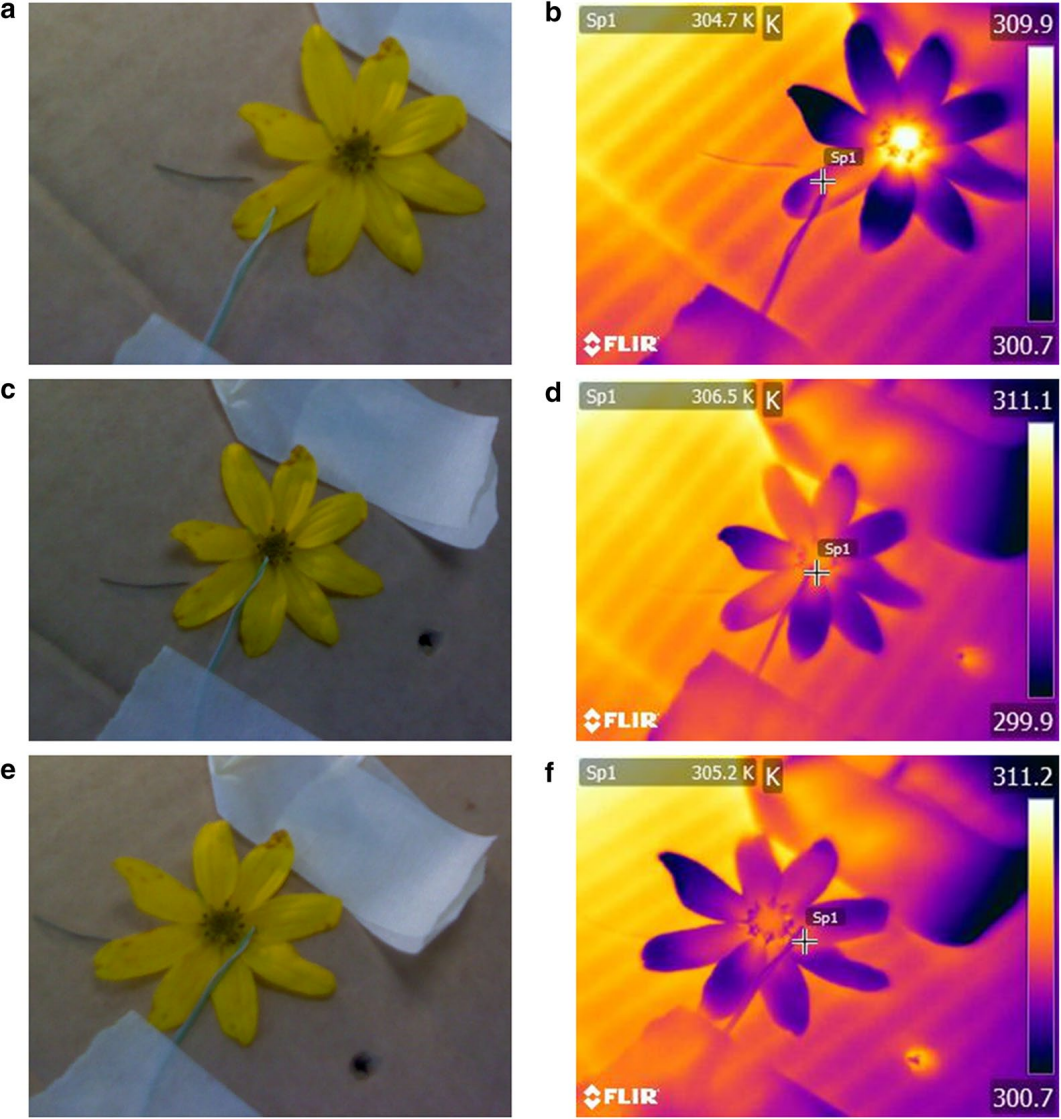
A demonstration of thermocouple measurement locations. Placement of thermocouple is shown in photographs (**a**, **c**, **e**) and the placement of thermograph point measurements relative to them are shown by point *sp1* in thermographs (**b**, **d**, **f**). Images are those taken for *Coreopsis verticillata* (individual 13 of that species). Measurements are taken from: **a**, **b** the petal edge; **c**, **d** reproductive structures; and **e**, **f** the petal base. The thermocouple is the green wire visible in each photograph, the measurement bulb being at the end of the wire. For each thermograph the apparent temperature value measured at point sp1 is given in the top left of each image. Apparent temperature is indicated in thermographs via the colour scale to the right of each thermograph. In thermal images all values are in Kelvin

Emissivity was first estimated using a K-type exposed wire thermocouple (HI-766F1, Hanna instruments, Leighton Buzzard, UK). Here a thermocouple temperature reading carried out simultaneously with thermographic image collection allows measurement of *T*_*obj*_ and *T*_*app*_ at the point of thermocouple measurement. As this protocol compares radiation and temperature at points of thermocouple measurements, it should not depend on even heating across the whole flower. As flowers typically do not heat evenly [28, 40, 41], this may reduce a common issue encountered when applying coating methods to flowers. Emissivity estimates for ten flower species were collected using this method, listed in Table 1. All flowers used for these estimates were grown at the National Botanic Garden of Wales, Carmarthenshire (51° 50′ 28″ N 4° 08′ 52″ W), over summer 2016 and summer 2017. Flowers were picked and brought to the Garden’s laboratory. Flowers were not collected from the field if they appeared damaged, aged, diseased or wet, as this may alter flower tissue composition and texture or change floral geometry, thus altering emissivity making emissivity estimates less applicable. Once inside the lab, flowers were placed within holes in a cardboard stand above an electrical greenhouse heater (Lighthouse Ecolight Heater IP55) and a desk lamp. The heater and lamp maintained flower temperature slightly above room temperature (assumed be at 25 °C, 298.2 K, and 50% relative humidity). Flowers were collected at suitable intervals to ensure heating began within an hour of picking. Heated flowers were taken one at a time from above the heater and moved to a separate section of the cardboard stand where thermographs and thermocouple measurements for emissivity estimates were made. Thermocouple measurements were not taken on parts of the flower where the flower surface had begun to visibly wrinkle and crease due to heating and drying, as this might affect surface texture, and thus cause emissivity to not be representative of flowers under natural circumstances. If there were no locations that were undamaged the flowers would be replaced. In *Cistus* ‘Snow Fire’ flowers petals occasionally detached at the base during thermocouple application. If this occurred measurements were carried out as normal on the recently detached petals. A schematic diagram of the cardboard stand used for thermocouple measurements and flower heating is available in Additional file 1: Fig. S1. Thermocouple temperature measurements were taken at three locations on each flower (Fig. 1): (i) ‘reproductive structures’, either on or about the carpel for flowers, or on disc florets of compound inflorescences, as appropriate; (ii) towards the periphery of the flower or ray floret’s adaxial petal surface, the ‘petal edge’; and (iii) towards the base of these petals, the ‘petal base’. These multiple measurement locations allow assessment of how emissivity changes across the flower surface, potentially influenced by changes in tissue composition, texture, and geometry. As one goal of this study is to obtain emissivity estimates that can be used for thermography of flowers as they occur naturally, we did not attempt to manipulate or control these aspects across the flower, as this would cause measurements to not be representative of real flowers in the field. In *Taraxacum* agg*.*, measurements were taken on the petals of inner and outer ray floret petals for the petal base and petal edge respectively. Alongside each thermocouple measurement a thermographic image was taken using a FLIR E60bx thermal camera (FLIR systems Inc., Wilsonville, USA) at a distance of approximately 0.5 m. Reflected temperature measurements were taken periodically throughout sampling using a tin foil multidirectional mirror as described by [49], reflective temperatures ranging between 287.0 and 300.5 K (13.8 to 27.3 °C). Thermocouple measurements of floral temperature were between 296.9 and 309.2 K (23.7 to 36 °C). Numbers of individual flowers of each species used for thermocouple estimates are given in Table 1. Simultaneous measurements with the thermocouple and thermal camera at each location were collected for each individual flower. However, due to mechanical failure of the thermal camera some thermographs of certain locations on individual flowers were not saved. A full breakdown of replication at each measurement location is given in Table 1.

**Table 1:** A breakdown of the species and replication levels sampled by both data collection protocols

Species	Thermocouple protocol				Water bath protocol			
	*N*	Repro struc	Petal base	Petal edge	*n*	Repro struc	Petal base	Petal edge
*Bellis perennis* L.	19	19	19	18	12	12	12	12
*Campanula* sp.	21	21	21	21	12	12	12	12
*Cistus* ‘Snow Fire’	21	21	21	21	–			
*Coreopsis verticillata* L.	20	20	20	20	–			
*Eschscholzia californica* Cham.	–				12	12	12	12
*Geranium pratense* L.	20	20	19	20	–			
*Geranium psilostemon* Ledeb.	20	19	20	20	–			
*Helianthemum* sp.	20	20	19	20	–			
*Leucanthemum vulgare* (Vaill.) Lam.	20	20	20	20	12	12	12	12
*Papaver cambricum* L.	20	20	20	20	–			
*Potentilla fruticosa* L.	–				12	12	12	12
*Taraxacum* agg.	20	20	20	19	12	12	12	12

For each species in each protocol, the number of individual flowers sampled (*n*) and the breakdown of measurements taken at each measurement location are given. measurement location ‘Repro struc’ indicating reproductive structures. Where total number of individuals and number of measurements at a location differ, this indicates a measurement is missing due to camera saving error. An *n* value of ‘–’ indicates that the species was not measured by that protocol

The parameters required for floral emissivity estimation were obtained at each measurement point from the corresponding thermocouple readings, thermographs and reflected temperature measurements. The thermocouple temperature readings (in K) are *T*_*obj*_. From the thermograph, *T*_*app*_ was obtained using the point measurement function in *FLIR Tools* [77]. This *FLIR Tools* point measurement was manually placed on the thermograph proximal to the thermocouple bulb (Fig. 1). As the thermocouple bulb obscures the point at which temperature was being measured it was assumed that the point proximal to it was of the same temperature and emissivity. Setting *ε* to 1 and distance to 0 in the thermograph parameter inputs in *FLIR Tools* provided *T*_*app*_ from this point measurement. The *T*_*ref*_ values taken alongside thermocouple measurements with a multidirectional mirror were used in emissivity estimation. The environmental temperature of the lab in which this protocol was conducted was not actively controlled. For emissivity estimation, *T*_*env*_ was assumed to be 25 °C (298.2 K), a value close to the room’s approximate temperature. The function used to estimate *ρ* by the FLIR thermal camera is proprietary knowledge, and so *ρ* was instead assumed to be 0.99. This value is consistent with the near-perfect transmission of thermal infrared radiation at short distances based on various thermal infrared transmission functions, including that used by the FLIR camera [75]. These assumed values for *T*_*env*_ and *ρ* would have only small influences on emissivity estimates (Eq. 1). With these values for *T*_*obj*_, *T*_*app*_, *T*_*ref*_, *T*_*env*_, and *ρ* (and the Steffan–Boltzman constant, *σ*) emissivity could be estimated using the calculation and *Solver* methods described above.

From this protocol we obtained the necessary information for emissivity estimates at each location (with the exclusions noted above) across each flower. For each measurement location we made emissivity estimates by calculation and by *Solver* software, however only the emissivity estimates produced by *Solver* were analysed (as estimates were very similar, see below, the effect on conclusions would be minimal). The effects of flower species and measurement location on floral emissivity was assessed using a repeated measures ANOVA including individual flower as a random factor. If interacting effects occurred, measurement location effects were compared within each species and differences between species were compared at each measurement location. Pairwise comparisons within locations and species were compared with a post hoc Tukey’s test. All analyses were carried out in *R* 3.6.3 [78], ANOVAs were carried out using *LmerTest* 3.1-1 [79], while post hoc tests used *emmeans* 1.4.5 [80].

### Water bath protocol

**Fig. 2:**
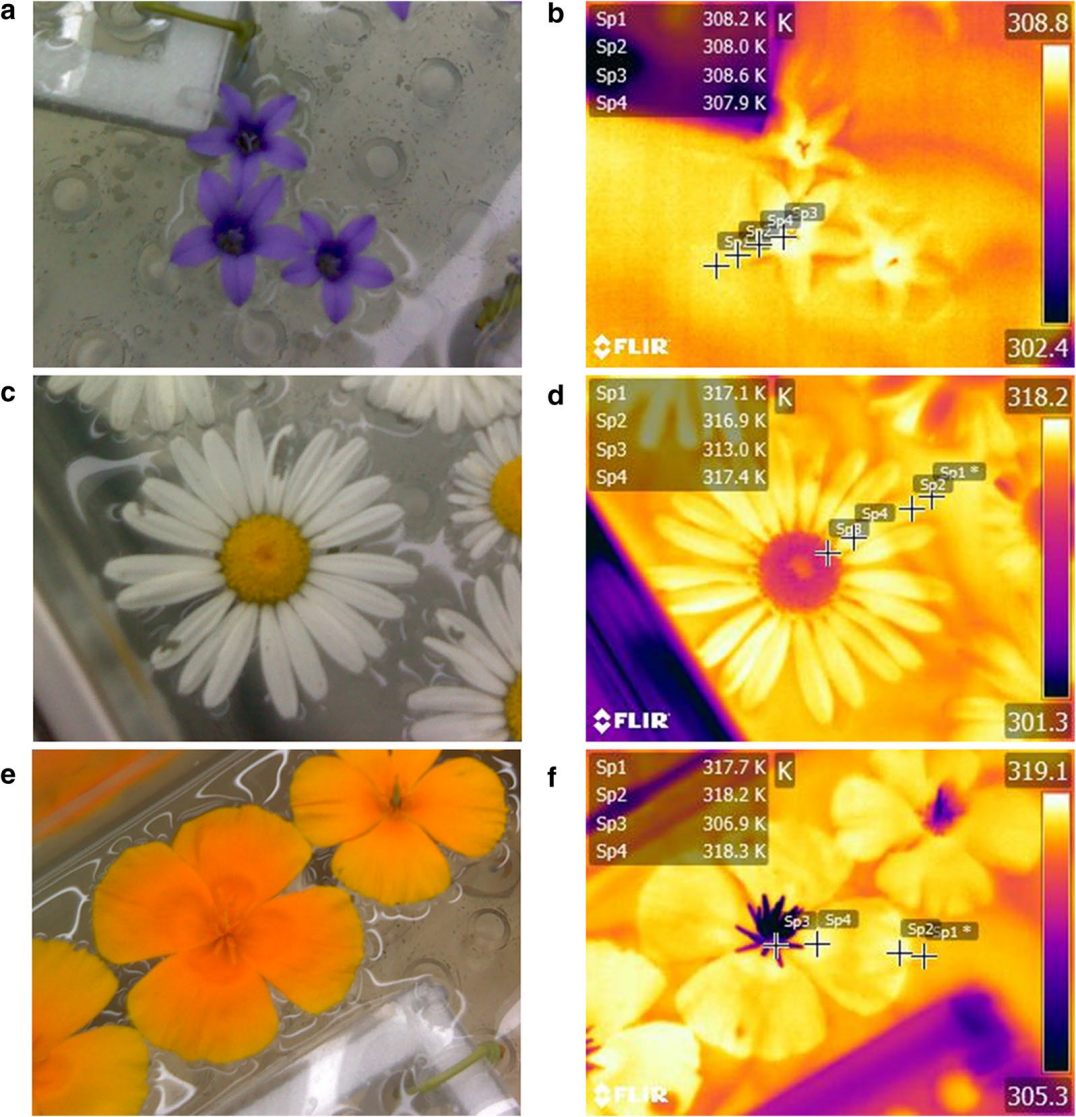
A demonstration of measurements collected from the water bath protocol. Paired photographs (**a**, **c**, **e**) and thermographs are given (**b**, **d**, **f**) for: **a** and **b** *Campanula* (individual 13 at set bath temperature of 35 °C); **c**, **d** *Leucanthemum vulgare* (individual 3 at set bath temperature of 45 °C); and **e**, **f** *Eschscholzia californica* (individual 3 at set bath temperature of 45 °C). On each thermograph four point measurements are taken. ‘sp1*’ measures water temperature, the ‘*’ indicates it has separate parameters (pertaining to water emissivity, described in main text) from other point measurements. ‘sp2’, ‘sp3’ and ‘sp4’ measure the apparent temperature of the petal edge, reproductive structures and petal base respectively. For each thermograph the temperature value measured by each point is given in the top left of each image. Apparent temperature is indicated in thermographs via the colour scale to the right of each thermograph. In thermal images all values are in Kelvin

This method for emissivity estimation is an adapted version of that used by López et al. [53] on leaves. Here flowers floating in a water bath are heated to a set temperature and emissivity is estimated. This protocol assumes flowers have been heated to the same temperature as the water to make emissivity estimates. In summer 2018, flowers were picked from the University of Bristol Royal Fort Gardens (51° 27′ 29″ N 2° 36′ 10″ W) and brought to and adjacent, climate-controlled lab (maintained at 25 °C, 298.2 K, and 50% humidity, University of Bristol Life Sciences Building). Flowers were collected in batches of about 10 to 20 and would be brought to the lab within an hour of collection. Flowers were again not collected from the field if they appeared aged, diseased, damaged or wet. Once in the lab, flowers were immediately placed within a filled temperature-controlled unstirred water bath (SAP26, Grant Instruments, Cambridge, UK), upward facing so that they would float keeping their upper surface dry (Fig. 2). The upper floral surface of floating flowers had to remain dry to ensure apparent temperature measurements were taken on floral tissue, not a film of water on top of it. Alongside flowers, floating test tube racks were also placed in the water bath to prevent flowers from moving around on currents created as the bath heated up. The bath was then closed and allowed to reach a set temperature of 35 °C (308.2 K), and was then left for a further 15 min before verifying the water temperature with a fluid-in-glass thermometer. In all instances the thermometer reading found the bath temperature to be ± 1 K of the set bath temperature. After verification, thermographs would be taken of each flower using the FLIR E60bx camera at a distance of approximately 0.5 m, and care was taken to ensure some water was visible in each thermograph. The water bath was then set to 45 °C (318.2 K). This sequence (heating, waiting time, verification and thermal image collection) was then repeated on the same flowers for the new water bath temperature. Reflected temperature was estimated before each batch of thermal image collection and at each temperature setting using a multidirectional tin foil mirror placed over the corner of the water bath. The set temperatures of the water bath were chosen based on the findings of López et al. [53], who found emissivity estimates to have settled at consistent values by these temperatures (45 °C, or 318.2 K, being the highest temperature used for estimation by López et al.). Due to the influence of warming from solar radiation, flowers can reach temperatures comparable to these set temperatures of the water bath naturally without suffering damage [24, 28, 31]. Thus, it is unlikely these temperatures would damage floral tissue. Reflected temperature ranged from 299.5 to 301.6 K (26.3 to 28.4 °C) during water bath measurements. Numbers of individual flowers of each species used for water bath estimates are given in Table 1.

The parameters required for floral emissivity estimation could be extracted from thermographs and reflected temperature measurements. Only a single thermal image of each flower at each set temperature is required for the protocol. On each flower thermograph, *T*_*app*_ measurements were made in *FLIR Tools* at three manually placed points on the flower equivalent to those measured in with the thermocouple (Fig. 2), again to allow assessment of how emissivity changes across the flower. As with the thermocouple protocol, we did not attempt to manipulate or control floral composition, geometry, or surface texture, as this would impact how applicable emissivity estimates would be to natural flowers (see above). Flowers in the water bath did not appear to wrinkle or crease with heating in the water bath, likely due to the available water source preventing such drying, thus this did not need to be considered during placement of measurement points. Point measurements were taken from the lower regions, on reproductive structures in an attempt to measure regions most likely to be at the water temperature. This method assumes that the flower has reached a temperature equilibrium with the water in the bath, therefore *T*_*obj*_ was obtained by measuring the temperature of the water proximal to the flower in the thermograph (Fig. 2). For *T*_*obj*_ measurements of water temperature: emissivity of water is 0.98, an accepted emissivity for water [53, 81, 82]; distance as 0.5 m; relative humidity as 50%, *T*_*env*_ as 25 °C (298.2 K, the maintained lab temperature). For both emissivity estimates and collection of water temperatures for *T*_*obj*_ measurements the reflected temperatures measured throughout sampling with the multidirectional mirror were used. Values for *T*_*env*_ and *ρ* were 298.2 K (25 °C) and 0.99, as used in the thermocouple estimates protocol (see above).

From this protocol we obtained the necessary information for emissivity estimates at each flower measurement location when the bath was set to 35 °C and when the bath was set to 45 °C. Emissivity estimates were made at each measurement location and temperature setting by calculation and by *Solver*. Again, only the emissivity results calculated by *Solver* were analysed. The effects of set water bath temperature (here treated as a categorical factor with two levels: 35 °C and 45 °C), measurement location and flower species were compared using a repeated measures ANOVA with individual flower as a random factor. If interactions occurred the effects of measurement location and water bath temperature were assessed at the species level. Between-species differences were compared at each measurement location when the bath was set to 45 °C. Pairwise comparisons were assessed using post hoc Tukey’s tests.

### Using vegetative emissivity values for floral thermography

In addition to estimating emissivity, we used the thermographs collected across both emissivity estimation protocols to calculate how well the thermal camera measured floral temperature, when an emissivity value of 0.98 was used. An emissivity value of 0.98 is typical of that chosen by floral thermography studies [28, 41, 42, 49] based on vegetative tissue emissivity estimates. Assessing how well the camera measures flower temperature when using this value allows a further assessment of how appropriate this value choice is. In each flower thermograph collected during both protocols of emissivity estimation, using the same *FLIR Tools* measurement points placed for *T*_*app*_ measurements (described above), we obtained a temperature measurement using an emissivity value of 0.98, *T*_0.98_. This was done in *FLIR Tools* by setting parameters of each thermograph as follows: emissivity to 0.98; distance to 0.5 m; relative humidity to 50%; *T*_*env*_ to 25 °C; and the reflected temperature value to that measured during sampling. Each *T*_*obj*_ measurement was compared to the corresponding floral temperature measurement collected during emissivity estimation (*T*_*obj*_, collected as described above for each protocol). This was done by calculating at each measurement the difference point between the flower temperature measurement collected during emissivity estimation and the camera’s corresponding flower temperature measurement (*T*_*obj*_ minus *T*_0.98_).

## Results

### Thermocouple estimates

When emissivity estimates were made using the thermocouple protocol, estimates that were resolved by calculation (Eq. 3) differed little from those resolved using *Solver*. The difference between the two solutions at a measurement point (*ε* estimate from *Solver* minus the emissivity estimate when calculated) was on average very small, 0.001 ± 0.015 (mean ± SEM). Mean thermocouple estimates of emissivity by calculation and *Solver* across all species are available in Additional files 2 and 3 respectively.

**Fig. 3:**
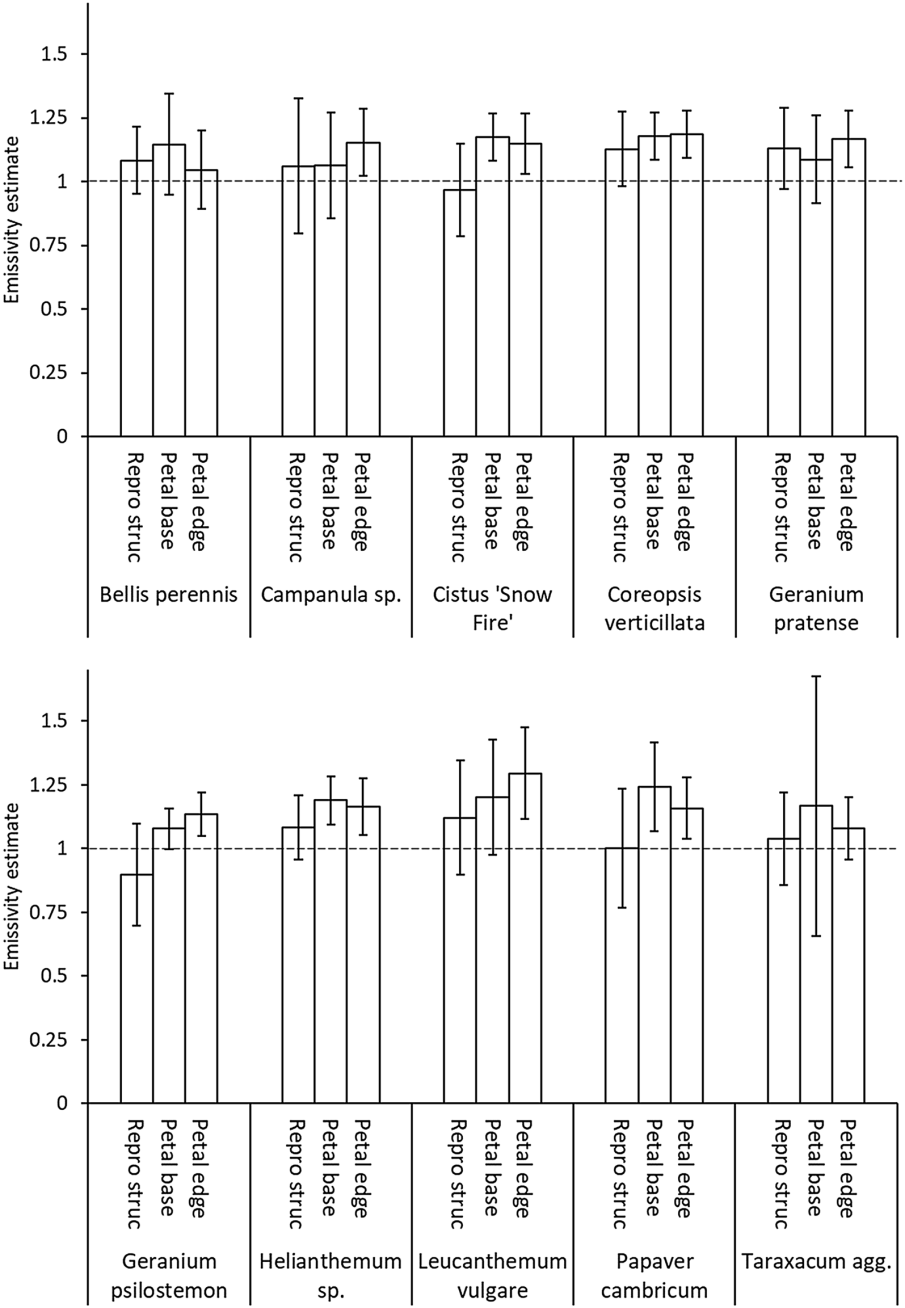
The emissivity estimates collected by the thermocouple protocol and resolved by *Solver*. Plotted are the mean estimates for each measurement location of each species. Error bars indicate ± a standard deviation of the mean. Measurement location are: Petal edge, Petal base and Reproductive structures (‘Repro struc’). Dashed line indicates an emissivity value of 1, the theoretical upper limit of emissivity

The (*Solver*) estimates of floral emissivity using the thermocouple method were regularly greater than 1, the theoretical upper limit of emissivity [44–49], with mean estimates of emissivity being below 1 only in the reproductive structures of *Cistus* ‘Snow Fire’ and *Geranium psilostemon* (Fig. 3). Emissivity was found to be influenced by a significant interaction between measurement location and species (*F*_18,379_ = 2.047, p = 0.007) suggesting some locations on some species vary from others in their emissivity. Emissivity was found to vary with measurement locations in five of the ten species sampled (Table 2). In each of these cases the reproductive structures of the flower had lower emissivity estimates than the measurements taken across the petals. In no instance was emissivity found to vary between the edge and base of the petal.

**Table 2:** The summary results of ANOVA tests for the effect of measurement location within each species

Species	*F*	df	*P*	Post hoc
*B. perennis*	1.781	2, 53	0.178	–
*Campanula* sp.	1.357	2, 60	0.265	–
*Cistus* ‘Snow Fire’	16.772	2, 40	< 0.001	R ~ (B + E)
*C. verticillata*	2.071	2, 38	0.140	–
*G. pratense*	2.284	2, 37	0.116	–
*G. psilostemon*	19.047	2, 38	< 0.001	R ~ (B + E)
*Helianthemum* sp.	6.355	2, 38	0.004	R ~ (B + E)
*L. vulgare*	3.401	2, 57	0.040	R ~ E
*P. cambricum*	9.077	2, 39	< 0.001	R ~ (B + E)
*Taraxacum* agg.	0.825	2, 56	0.444	–

*F* statistics (*F*), degrees of freedom (df), and probability (*p*) are given, as are a summary of the results of post hoc Tukey’s tests where: ‘R ~ (B + E)’ indicates a significant difference (*p* ≤ 0.05) between reproductive structures and the petal base as well as a significant difference between the reproductive structures and the petal edge within that species; ‘R ~ E’ indicates a significant difference between the reproductive structures and the petal edge; – indicates no significant effect of measurement location

While the majority of species did not vary in emissivity according to estimates based on the thermocouple protocol, differences in emissivity estimates between species were found at some measurement locations. Significant differences between species were found in the emissivity estimates at their reproductive structures (*F*_9,190_ = 3.164, *p* = 0.001) and the petal edge (*F*_9,189_ = 5.283, *p* < 0.001) but not at estimates taken at the petal base (*F*_9,189_ = 1.423, *p* = 0.181). Post hoc tests revealed differences between emissivity estimates of reproductive structures were the result of significant differences between *Geranium psilostemon* and *G. pratense*, *Coreopsis verticillata* as well as *Leucanthemum vulgare*. Differences between the petal edges were the result of differences between *Bellis perennis* and *C. verticillata* and differences between *L. vulgare* and all other species except *C. verticillata*.

### Water bath estimates

Water bath emissivity estimates at each point differed less (compared to solutions for the thermocouple protocol) when solutions were reached by calculation or using *Solver*. The difference between the two solutions of a measurement point here being 0.0002 ± 0.0001 (mean ± SEM). Mean water bath estimates of emissivity by calculation and *Solver* across all species are available in Additional files 4 and 5 respectively.

**Fig. 4:**
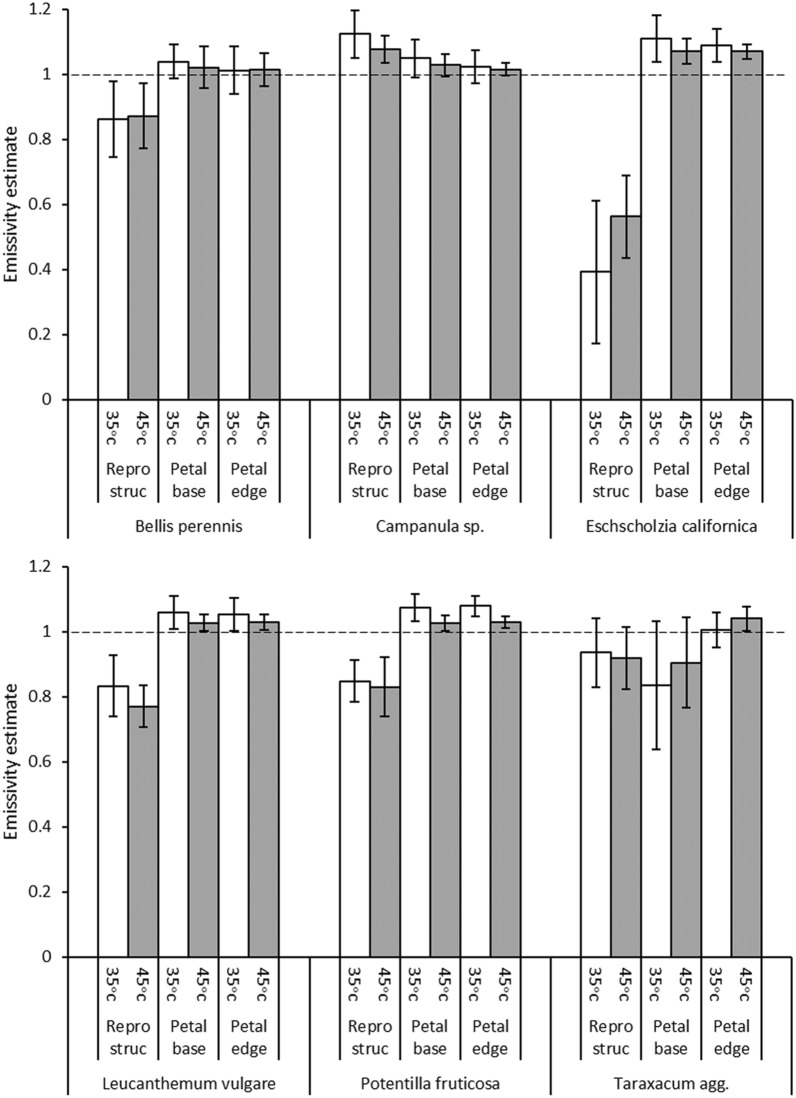
The emissivity estimates collected by the Water bath protocol and resolved by *Solver*. Plotted are the mean estimates for each measurement location of each species at each set temperature of the water bath. Error bars indicate ± a standard deviation of the mean. Measurement locations are: Petal edge, Petal base and Reproductive structures (‘Repro struc’). Dashed line indicates an emissivity value of 1, the theoretical upper limit of emissivity

The estimates of floral emissivity using the water bath method were also generally high, with the exception of the reproductive structures of *Eschscholzia californica* (Fig. 4). Emissivity estimates of petals, both at the edge and base, tended to be slightly higher than, but very close to, 1. Emissivity of reproductive structures tended to be lower than petal emissivity, except in *Campanula* sp. where reproductive structures were estimated to have an emissivity greater than 1. Emissivity estimates were found to be affected by a three-way interaction between the set temperature of the water bath, measurement location and species (*F*_10,330_ = 3.416, *p* < 0.001) suggesting the emissivity estimate is affected by each of these. Differences in emissivity estimates with measurement locations were found within each of the six species (see results in Table 3). In most species the reproductive structures differed from both locations on the petals. However, in *Taraxacum* agg. the petal edge differed from other locations, being of a higher emissivity. Water bath temperature was found to influence the emissivity estimate in *Campanula* sp.,* L. vulgare* and *Potentilla fruticosa*, where raised water bath temperature lowered emissivity estimates. Water bath temperature also had an interacting effect with measurement location on the emissivity estimates made on *E. californica*. Here emissivity estimates were lowered with increased bath temperature across the petal but were raised on reproductive structures. The changes in emissivity estimates across *E. californica* maintained the same relationship structure at both bath temperatures, with the reproductive structures showing lower emissivity than petals, but to a differing degree dependent on water bath temperature.

**Table 3:** The summary results of ANOVA tests for the effect of measurement location and the set water bath temperature within each species

Species	Effect	*F*	df	*p*	Post hoc
*B. perennis*	Interaction	0.30	2, 55	0.743	–
	Water bath temp	0.03	1, 55	0.859	–
	Measurement location	50.19	2, 55	< 0.001	R ~ (B + E)
*Campanula* sp.	Interaction	1.03	2, 66	0.362	–
	Water bath temp	4.52	1, 66	0.037	*
	Measurement location	17.70	2, 66	< 0.001	R ~ (B + E)
*E. californica*	Interaction	6.80	2, 55	0.002	*
	Water bath temp	2.07	1, 55	0.156	–
	Measurement location	249.31	2, 55	< 0.001	*
	Measurement location at 45 °C	215.59	2, 22	< 0.001	R ~ (B + E)
	Measurement location at 35 °C	109.84	2, 22	< 0.001	R ~ (B + E)
*L. vulgare*	Interaction	0.90	2, 55	0.411	–
	Water bath temp	10.55	1, 55	0.002	*
	Measurement location	167.56	2, 55	< 0.001	R ~ (B + E)
*P. fruticosa*	Interaction	0.71	2, 66	0.493	–
	Water bath temp	10.36	1, 66	0.002	*
	Measurement location	136.12	2, 66	< 0.001	R ~ (B + E)
*Taraxacum* agg.	Interaction	1.16	2, 55	0.320	–
	Water bath temp	1.53	1, 55	0.222	–
	Measurement location	14.23	2, 55	< 0.001	E ~ (B + R)

*F* statistics (*F*), degrees of freedom (df), and probability (*p*) are given for each effect and their interactions, as are a summary of the results of post hoc Tukey’s tests (Post hoc). * Indicates a significant effect of water bath temperature. Post hoc results for measurement location are indicated by: ‘R ~ (B + E)’ indicates a significant difference (*p* ≤ 0.05) between the reproductive structures and the petal base as well as a significant difference between the reproductive structures and the petal edge within that species; ‘E ~ (B + R)’ between the petal edge and the petal base as well as a significant difference between the petal edge and the reproductive structures of that species; ‘–’ indicates no significant effect at that level

When the differences in emissivity estimates between species were compared at each measurement location when the water bath was set to 45 °C, significant between-species differences in emissivity estimates were found at each measurement location (*reproductive structures F*_5,66_ = 42.26, *p* < 0.001; *petal base F*_5,66_ = 8.19, *p* < 0.001; *petal edge F*_5,66_ = 5.32, *p* < 0.001). Post hoc tests revealed differences between emissivity estimates of reproductive structures were the result of significant differences between *E. californica* and all other species, *Campanula* sp. and all other species and differences between *L. vulgare* and *Taraxacum* agg. Differences between species at the petal base were the result of *Taraxacum* agg. having a significantly different emissivity than all other species, differences between species at the petal edge were due to *E. californica* differing from all species except *Taraxacum* agg.

### Using vegetative emissivity values for floral thermography

Across both protocols, thermographic temperature measurements made using an emissivity value of 0.98 were normally similar to the floral temperature measurements obtained during emissivity estimation (Table 4). The differences between the two temperature measurements paralleled patterns of differences in emissivity estimates. Measurement locations on species estimated to have emissivity differing from other species tended to show greater differences between the two temperature measurements (cross reference Figs. 3 and 4 with Table 4). However, across most measurement locations and species, camera settings used for *T*_*0.98*_ normally matched *T*_*obj*_ closely (± 1 K difference). Consequently, conducting floral thermography with an emissivity value of 0.98 appears to obtain a close estimate of floral temperature across most floral tissue sampled.

**Table 4:** The summary of the difference between thermographic floral temperature measurements taken using an emissivity value of 0.98 (*T*_*0.98*_) and the corresponding object temperature measurement made during emissivity estimation (*T*_*obj*_)

Species	Thermocouple Protocol			Water bath protocol		
	Repro struc	Petal base	Petal edge	Repro struc	Petal base	Petal edge
*Bellis perennis*	− 0.37 ± 0.14	− 0.79 ± 0.13	− 0.39 ± 0.19	0.85 ± 0.22_35_	− 0.31 ± 0.08_35_	− 0.15 ± 0.13_35_
				1.74 ± 0.42_45_	− 0.50 ± 0.26_45_	− 0.39 ± 0.21_45_
*Campanula* sp.	− 0.24 ± 0.21	− 0.58 ± 0.19	− 0.81 ± 0.13	− 0.79 ± 0.10_35_	− 0.35 ± 0.09_35_	− 0.20 ± 0.08_35_
				− 1.35 ± 0.17_45_	− 0.63 ± 0.14_45_	− 0.43 ± 0.09_45_
*Cistus* ‘Snow Fire’	− 0.02 ± 0.15	− 0.84 ± 0.09	− 0.92 ± 0.13			
*Coreopsis verticillata*	− 0.92 ± 0.18	− 1.05 ± 0.12	− 1.21 ± 0.11			
*Eschscholzia californica*				3.75 ± 0.40_35_	− 0.77 ± 0.12_35_	− 0.63 ± 0.08_35_
				7.28 ± 0.67_45_	− 1.38 ± 0.17_45_	− 1.37 ± 0.09_45_
*Geranium pratense*	− 0.50 ± 0.13	− 0.42 ± 0.12	− 0.95 ± 0.12			
*Geranium psilostemon*	0.16 ± 0.15	− 0.49 ± 0.11	− 0.84 ± 0.12			
*Helianthemum* sp.	− 0.34 ± 0.10	− 1.15 ± 0.14	− 1.06 ± 0.14			
*Leucanthemum vulgare*	− 0.89 ± 0.16	− 0.90 ± 0.22	− 1.06 ± 0.12	0.98 ± 0.16_35_	− 0.45 ± 0.09_35_	− 0.38 ± 0.09_35_
				3.25 ± 0.28_45_	− 0.58 ± 0.10_45_	− 0.61 ± 0.10_45_
*Papaver cambricum*	− 0.02 ± 0.09	− 1.11 ± 0.16	− 0.85 ± 0.11			
*Potentilla fruticosa*				0.82 ± 0.11_35_	− 0.49 ± 0.07_35_	− 0.54 ± 0.05_35_
				2.63 ± 0.45_45_	− 0.65 ± 0.12_45_	− 0.70 ± 0.09_45_
*Taraxacum* agg.	− 0.16 ± 0.15	− 0.58 ± 0.32	− 0.53 ± 0.16	0.32 ± 0.19_35_	0.97 ± 0.35_35_	− 0.11 ± 0.11_35_
				1.05 ± 0.41_45_	1.13 ± 0.61_45_	− 0.79 ± 0.15_45_
Average	− 0.33 ± 0.05	− 0.79 ± 0.06	− 0.87 ± 0.04	0.99 ± 0.18_35_	− 0.23 ± 0.09_35_	− 0.33 ± 0.04_35_
				2.43 ± 0.35_45_	− 0.44 ± 0.15_45_	− 0.72 ± 0.06_45_

Provided are the mean ± SEM differences between these two temperature measurements (*T*_*obj*_ − *T*_0.98_) in degrees Celsius/Kelvin for each measurement location on each species (as well as the average for all species) across both measurement protocols. ‘Repro struc’ indicating reproductive structures. For the water bath protocol mean differences are provided for both set temperatures of the water bath (35 and 45 °C), these are indicated by subscript values following mean ± SEM values

## Discussion

The emissivity estimates collected by both protocols indicate that floral emissivity has a high value. Thermographic floral temperature measurements made using an emissivity value of 0.98 (*T*_0.98_) differed little from floral temperature measurements made during emissivity estimation (*T*_*obj*_). That these two temperature measurements aligned also indicates floral emissivity has a high value, likely near 0.98. Emissivity estimates differed between certain species and between different locations on the flower in several species. However, the emissivity estimates produced by these estimation protocols and differences observed between them should be evaluated within the context of the assumptions and potential weaknesses or shortcomings of the protocols that produced them. While all emissivity estimation protocols have assumptions, we must consider how well these assumptions are met. This must be done to ensure that our interpretation of these emissivity estimations, and ultimately choice of emissivity values used, are not influenced by artefacts of the two experimental protocols. Mean emissivity estimates were (to varying degrees depending on the protocol) frequently above 1, the theoretical upper limit of emissivity [44–49], dictating caution in accepting the mean estimates at face value. In this section we shall evaluate both protocols and the floral emissivity estimates they produce within the context of the assumptions and shortcomings of the protocols. In doing so we will form a practical interpretation of these results and provide recommendations for future researchers in both floral emissivity value choice, and choice of protocols for estimation floral emissivity. This information will help improve the accuracy of floral thermographic temperature measurements. We shall also discuss the extent our results influence our confidence in temperature measurements made by previous floral thermography research, and the findings of those studies.

### Evaluation of emissivity protocols

Many of the mean emissivity estimates exceeded 1, and were therefore values that cannot be input into most thermal cameras. This was particularly true of thermocouple emissivity estimates across petals (Fig. 3). While most emissivity estimates in both protocols were within a standard deviation of realistic emissivity values that could be used and input into the camera (values between 0 and 1), mean thermocouple estimates were often much higher and more variable (Fig. 3). Water bath mean emissivity estimates were generally lower, and this is clearest on species measured by both protocols. Estimates made by the water bath protocol gave emissivity values very near to or below 1, and were less variable. When mean water bath estimates exceeded 1, this was normally by a much smaller amount (Fig. 4) and such estimates were frequently within a single, much smaller, standard deviation of realistic values, at least when the bath was at its higher temperature setting (45 °C), where we would expect the relationships between radiation and temperature to be clearer. If the true emissivity of floral tissue was near 1, we might expect mean estimates to slightly exceed 1 due to small inaccuracies in measurements [53, 71, 76]. Such inaccuracies include the accuracy of temperature measurements made by both the thermal camera (quoted as ± 2 °C by the manufacturers) or thermocouple (roughly ± 2.2 °C or ± 0.75% of the measured value, whichever is greater) before errors in conducting measurements are considered. The assumed values for *T*_*env*_, *ρ*, distance and relative humidity used at various points in emissivity estimation may also have small contributions to inaccuracy [46–49]. Such sources of measurement inaccuracy explain the incidence of the mean emissivity estimates slightly above 1 produced by the water bath protocol. Considering this, we can interpret the water bath estimates slightly above 1 as indicating that the true emissivity of those flower locations to be less than but very close to 1. Similar slightly greater than 1 mean estimates of leaf and insect emissivity have been interpreted in this way for similar reasons [53, 71]. However, these small sources of inaccuracy do not explain the much higher and more variable thermocouple estimates, particularly across petals. These are likely to be the result of another larger source of error in the thermocouple protocol.

A weakness of both methods is that they assume we have the true *T*_*obj*_, i.e. the actual temperature of the flower, for emissivity estimation. This is true of other emissivity estimation methods, such as the coating method described above, which assumes even heating of the target object. In the water bath protocol, we assume the flower tissue has reached an equal temperature with the water proximal to it, and thus that water temperature is equal to the true *T*_*obj*_. In the thermocouple protocol, we assume thermocouple readings are a correct estimate of the true *T*_*obj*_. A potential cause of thermocouple estimates that are much greater than 1 may be that flowers do not facilitate a good quality thermocouple contact for accurate *T*_*obj*_ measurements, and so this particular assumption is not held in the thermocouple protocol. It is difficult to ensure a high-quality thermocouple contact on flowers, as flowers are soft and largely pliant (particularly across petals). Poor contact might result in consistent underestimation of *T*_*obj*_ particularly across flower petals, leading to overestimation of *ε* (see Eqs. 1, 2 and 3) by the thermocouple protocol. Additionally, emissivity estimation by the thermocouple protocol assumes that the point proximal to the thermocouple bulb is of the same temperature and same emissivity, and would consequentially have the same apparent temperature as the point of thermocouple contact. This is not necessarily true. As flowers do not heat up evenly, temperature may still differ across the small distances between these points. Due to it being a contact measurement tool, attachment of the thermocouple may alter the temperature of the flowers at point of contact but less at the point of *T*_*app*_ measurement. Also, there may be slight emissivity differences. This can mean accuracy of thermocouple emissivity estimates are also quite dependent on placement of apparent temperature measurement points, as some points proximal to the thermocouple bulb may meet this assumption better than others. This potential mismatch between locations where *T*_*obj*_ and *T*_*app*_ are collected in the thermocouple protocol, and that this protocol includes both the measurement inaccuracies from the thermal camera and the thermocouple, may increase error further. While thermocouple estimates of emissivity show a general trend for higher values that agrees with the results of the water bath protocol, these greater sources of error likely cause the more variable, less realistic and thus likely less accurate estimates produced by the thermocouple protocol.

The temperature at which emissivity estimation was carried out influenced the emissivity estimates. In the water bath protocol the higher temperature setting (45 °C) typically reduced emissivity estimates, except in the reproductive structures of *E. californica* where emissivity increased at higher temperatures. It is possible elevated temperatures of the water bath alter floral emissivity, due to heat damage to epidermal cells and denaturation of surface proteins, a greater risk with elevated temperature. However (as discussed above), flowers in nature often reach comparable temperatures to the set water bath temperatures [24, 28, 31, 41] and thus likely tolerate them for the timescales of our measurements without meaningful damage. Consequently, it is unlikely heat damage accounts for these differences in estimates. This change in estimates with temperature was often paired with a reduction in variation. Such changes can be interpreted as estimates approaching the true emissivity value [53], as the relationship described by emissivity becomes clearer [44–46]. That estimates generally approached 1 with elevated temperature, further suggests that the water bath estimates slightly above 1 indicate the true value of floral emissivity is near 1. This effect of the temperature at which measurements were carried out might also influence estimates made by the thermocouple protocol. Flowers used for thermocouple estimates were not heated to as great an extent as those in the water bath, only occasionally reaching temperatures comparable to the lower water bath setting (ranging between 23.8 and 36.0 °C, 296.9 and 309.2 K, according to the thermocouple). Thus, thermocouple estimates were taken at generally lower temperatures, perhaps further increasing the inaccuracy in this protocol. The temperature values chosen for the water bath were chosen based on the temperatures at which leaf emissivity estimates settled at López et al. [53]. Further heating beyond what flowers in nature normally experience may increase probability of heat damage to flower samples, conflating results. Furthermore, floral tissue is often more susceptible to water loss than leaf tissue as petal cuticles are typically more permeable [15, 56, 58, 83]. While there is a risk further heating might damage flowers, it is advised that measurements for floral emissivity estimation be carried out at as high a temperature as possible. This is particularly true if the thermocouple protocol is utilized.

In contrast to the two flower measurement protocols, whether emissivity was resolved by calculation or *Solver* gave very similar results for the same values. Though not apparent in the mean values, in rare instances resolving emissivity by calculation produced outlier values, where resolving by *Solver* did not (see raw data in Additional files 6 and 7). Such calculated values seemed to occur when reflected temperature, *T*_*ref*_, was greater than or equal to *T*_*obj*_ and/or *T*_*app*_ leading to negative or near zero nominator or denominator values in Eq. 3. It is unclear if such values are the result of error in a measurement of one or both temperature values. These outlier values suggest that the emissivity calculation protocol may not work as well when faced with imprecision or certain input value combinations. The nature of the *Solver* method, which is based on finding the most suitable *ε* value that leads to the best match between *W*_*exp*_ and *W*_*obs*_, seems to be more robust, perhaps making it the preferred approach. Another advantage of the *Solver* method (although not one implicated here for reasons described above) is that, if desired by experimenters, limits could be applied to how much *Solver* can vary emissivity to find the optimal *W*_*exp*_. This could allow resultant emissivity estimates to be constrained to what can be input into most cameras (0 to 1). Doing this might allow a straightforward answer to what emissivity value to use and avoid some of the interpretation of estimates we have made above. However, such instances of outlier calculated values were rare, and mean estimates largely similar (compare Additional files 2 and 4 with Additional files 3 and 5). Thus, using either calculation or *Solver* to resolve emissivity has little effect on conclusions.

### Between and within species differences

Incidences of variation in the floral emissivity estimates between different positions on the flower were found, generally with the lower reproductive structure emissivity estimates differing from those of petals (Tables 2 and 3). The slightly lower emissivity of reproductive structures such as disc florets, carpels and gynoecium estimated by the water bath protocol parallels emissivity estimates of 0.9 made on fruits [72]. These emissivity differences with location on the flower may be the result of differing tissue composition [55–58, 67, 68], surface structure [61–66] or geometry [59, 60] between locations. This pattern of emissivity was frequently seen in different species in both protocols. However, *L. vulgare* was the only species measured by both protocols to show a similar pattern of emissivity differences with measurement location through both protocols. There are alternative explanations for these results that relate to methodological weaknesses and assumptions in both protocols, discussed previously. Flower reproductive structures are normally firmer and less easily pliant than petals allowing for better thermocouple contacts. So, in the thermocouple protocol *T*_*obj*_ may be more frequently underestimated in petals than in reproductive structures. This would mean that reproductive structure emissivity estimates would not be so frequently overestimated, but would be lower and perhaps more accurately reflect the true emissivity. In this way variation in thermocouple contact quality may be creating differences in estimates with location as opposed to actual differences in tissue emissivity. Similar assumptions of the water bath protocol may explain location differences in emissivity estimates. In the water bath protocol, emissivity differences with measurement location correspond well to the vertical position of the structure or its contact with the water surface, suggesting this may be the underlying cause of these differences. Structures that lie flat on the water surface, like most petals, generally have an emissivity near 1. In contrast, structures sitting sufficiently high above the water to not contact the water surface such as the inner ray florets of *Taraxacum* agg. (its petal base measurement) and reproductive structures of all species, except *Campanula* sp., are estimated to have lower emissivity. In the water bath protocol it was assumed the whole flower is at the set temperature of the water bath. However, it is possible that flower locations not connected as well to the water surface do not reach this temperature level. This will mean we are not measuring the *T*_*app*_ for these locations at the set water bath temperatures. We may in fact be measuring the radiation of the structures (*T*_*app*_) for a lower object temperature. This effectively means the difference in emissivity estimates is because the *T*_*obj*_ used for calculation is too high (or depending on perspective, *T*_*app*_ too low), resulting in *ε* being underestimated. Likewise, Reproductive structures of *Campanula* sp., which is below the water level (Fig. 2a), are estimated to have a higher emissivity. *Campanula* sp. reproductive structures being below the water level may be slightly hotter than the water surface, as measured by the thermal camera, resulting in the opposite effect. The water bath protocol has been effectively used in the past for leaf emissivity estimation [53], but cut leaves do not have as complex vertical arrangements as flowers. Consequently, this issue was not encountered before. While locational emissivity differences may occur, that they seem to follow these trends in contact with the water surface suggest these locational differences may be artefacts of the measurement protocol. Even though the thermocouple protocol is generally less reliable, it is perhaps the better protocol for estimating emissivity of reproductive structures in light of this potential weakness of the water bath protocol. Considering this, our estimates imply that emissivity is largely uniform across petals, but we should perhaps be cautious to conclude reproductive structures have different emissivity from petals.

Considering these thermocouple and water surface contact artefacts on emissivity estimates, we can explain many of the emissivity estimate differences observed between species. Many of these differences may reflect differences between species in quality of thermocouple or water surface contact. This is particularly true of the reproductive structures of *E. californica* (cross reference locations on Fig. 2e, f with Fig. 4) and the petal base differences between *Taraxacum* and other species in the water bath protocol. In these species these structures are held protruding above the water level more so than corresponding locations in other species, and thus more likely to be underestimated (see Fig. 2e, f). These effects largely explain the differences in emissivity estimates seen between flower species, and the remaining estimates suggest that different flower species differ little in emissivity. However, certain between-species differences are not as easily explained by these methodological artefacts, suggesting emissivity may differ with flower species in rare instances. *Geranium psilostemon* was estimated to have a lower reproductive structure emissivity than other species. While this may reflect thermocouple contact (*G. psilostemon* perhaps allowing for better contact than other species), if this were the case, similar results would be expected for *G. pratense*, which has similar flower form*.* It is possible the glossier reflective surface of *G. psilostemon* reproductive structures, and changes in epidermal waxes and surface structure that create this [63, 66], lower emissivity in *Geranium psilostemon*. Although, similar decreases were not seen at *G. psilostemon* petal bases, which are also glossy, this may relate to problems with the thermocouple petal measurements. Likewise, the increased emissivity estimates of *E. californica* petal edges, relative to other species detected by the water bath protocol, does not seem to be explainable by surface water contact (see Figs. 2e, f) and may be the consequence of texture or petal composition differences.

## Conclusion

Of the two methods of emissivity estimation evaluated here, we recommend the water bath protocol as being most suitable for measuring petals. The thermocouple protocol is generally less accurate and produces less realistic values, but might still be of use to measure reproductive structure emissivity due to shortcomings of the water bath protocol in measuring emissivity of such structures. These protocols have been applied in this study over a range of species with varied flower structures so should be applicable, with minimal adaption, to any study species with floral blooms (flowers or inflorescences) large enough to be resolved by the thermal camera being used.

The emissivity estimates collected in this study indicate that flower petal emissivity is high and emissivity varies little across petals and between species. The water bath protocol indicated most flower petals have an emissivity near 1, but reproductive structure emissivity may have a lower emissivity. However, these differences, along with most instances of differences between species at given locations, appear to be an artefact of the measurement protocols. The thermocouple method (although generally less accurate) seems more reliable at estimating reproductive structure emissivity, and estimates a value near 1 across most species. Such higher emissivity values are typical for organic tissue [47, 49] and plant tissues [50–54]. This supports emissivity choices used previously for floral thermography based on vegetation emissivity measurements that are near 1 [23, 28, 37–42]. As floral emissivity is high, a small inaccuracy in values chosen should not affect accuracy of temperature measurements greatly [44–48]. Indeed, when floral temperature was measured using a high emissivity value (0.98) typical of those chosen previously, thermographic floral temperature measurements generally corresponded well to those taken during emissivity estimation. In most species and measurement locations the difference between *T*_*obj*_ and *T*_0.98_ was within that expected by the camera’s accuracy (± 2 °C) even though inaccuracies in *T*_*obj*_ measurement may also contribute to this difference. This suggests the camera measured floral temperature well with these settings, and that emissivity is near 0.98 across most floral tissue. Larger differences between *T*_*obj*_ and *T*_0.98_ may reflect differences in floral emissivity sufficient to impact accuracy, however these may also reflect larger inaccuracies in *T*_*obj*_ measurement, occurring due to artefacts of the measurement emissivity measurement protocols (as discussed above), widening differences. Thus, our findings do not suggest floral thermographic measurements based on similar vegetative tissue emissivity values need to be reconsidered. In this way, our results allow us to have greater confidence in the emissivity values chosen by past floral thermography studies and the temperature measurements made within them. A value of 0.98 can be considered an appropriate choice of floral emissivity value based on our findings. Choosing this value allows for the potential effects of reflections to be included but remains a high emissivity value as indicated by our estimates. This value is certainly appropriate for petal emissivity, based on the water bath estimates and appears to produce accurate thermographic measurements of floral temperature.

## Supplementary Information

**Additional file 1.** A photograph (**a**) and matching diagrammatic image (**b**) of the stand constructed for thermocouple estimates. Numbers on b indicate: 1) the desk lamp which aids flower heating; 2) the stand with holes above the greenhouse heater where flowers would be placed for heating; 3) the green house heater; 4) The stand’s support (not visible in **a**); 5) holes where flowers would be place for thermocouple measurement, there are two sizes to accommodate different flower sizes; 6) the thermocouple itself.

**Additional file 2. **Calculated thermocouple estimate summary. Full summary values of the calculated estimates collected *via* the thermocouple protocol. Column headings are as follows: ‘species’ the species of flower; ‘measurement.location’ the measurement location of estimates here reproductive structures are indicated as ‘A: repro struc’, petal base as ‘B: petal base’, petal edge as ‘C: petal edge’; ‘Ecal.mean’, ‘Ecal.SD’, ‘Ecal.Count’ and ‘Ecal.SEM’ are the mean, standard deviation, number of replicates and standard error of the mean calculated emissivity estimates respectively for the measurement location and species indicated in the previous columns. Additionally, there is a numbered index column (untitled).

**Additional file 3.** *Solver* thermocouple estimate summary. Full summary values of the *Solver* estimates collected *via* the thermocouple protocol. Column headings are as follows: ‘species’ the species of flower; ‘measurement.location’ the measurement location of estimates here reproductive structures are indicated as ‘A: repro struc’, petal base as ‘B: petal base’, petal edge as ‘C: petal edge’; ‘Esolv.mean’, ‘Esolv.SD’, ‘Esolv.Count’ and ‘Esolv.SEM’ are the mean, standard deviation, number of replicates and standard error of the mean *Solver* emissivity estimates respectively for the measurement location and species indicated in the previous columns. Additionally, there is a numbered index column (untitled). Mean and standard deviations listed here are also plotted points for Fig. 3.

**Additional file 4.** Calculated water bath estimate summary. Full summary values of the calculated estimates collected *via* the water bath protocol. Column headings are as follows: ‘species’ the species of flower; ‘measurement.location’ the measurement location of estimates here reproductive structures are indicated as ‘A: Repro Struc’, petal base as ‘B: Petal Base’, petal edge as ‘C: Petal Edge’; ‘Set.temperature.of.waterbath’ the water bath temperature; ‘Ecal.mean’, ‘Ecal.SD’, ‘Ecal.Count’ and ‘Ecal.SEM’ are the mean, standard deviation, number of replicates and standard error of the mean calculated emissivity estimates respectively for the measurement location of the species at the temperature indicated by the previous columns. Additionally, there is a numbered index column (untitled).

**Additional file 5.** *Solver* water bath estimate summary. Full summary values of the calculated estimates collected *via* the water bath protocol. Column headings are as follows: ‘species’ the species of flower; ‘measurement.location’ the measurement location of estimates here reproductive structures are indicated as ‘A: Repro Struc’, petal base as ‘B: Petal Base’, petal edge as ‘C: Petal Edge’; ‘Set.temperature.of.waterbath’ the water bath temperature; ‘Esolve.mean’, ‘Esolve.SD’, ‘Esolve.Count’ and ‘Esolve.SEM’ are the mean, standard deviation, number of replicates and standard error of the mean of the *Solver* emissivity estimates respectively for the measurement location of the species at the temperature indicated by the previous columns. Additionally, there is a numbered index column (untitled). Mean and standard deviations listed here are also plotted points for Fig. 4.

**Additional file 6.** Thermocouple estimation extracted data. The full data extracted from thermal images and the emissivity estimates from the thermocouple protocol. Column headings are as follows: ‘measurement’ a name identifier for each flower measurement. ‘Individual within species’ the numerical identifier of individual flowers within each species numbers are shared across species, this is used for flower identification not for analysis. ‘Individual’ numerical identifier of flower individual across all species. ‘Species’ the flower species. ‘measurement location’ the measurement location, here reproductive structures are indicated as ‘A: repro struc’, petal base as ‘B: petal base’, petal edge as ‘C: petal edge’. ‘temp on thermocouple = Tobj’ the thermocouple reading of flower temperature in degrees Celsius at the measurement indicated by the previous columns. ‘Tobj (k)’ the thermocouple reading of flower temperature in degrees Kelvin at the measurement indicated by the previous columns. ‘Tref’ the reflected temperature at the measurement indicated by previous columns. ‘at e = 1 d = 0, Temp uncorrected (for W)’ the apparent temperature at measurement indicated by the previous columns. ‘at e = 0.98 d = 0.5’ the camera’s temperature measurement, in kelvin, when emissivity was 0.98 at the measurement indicated by the previous columns. ‘DeltaTobj-T0.98’ the ‘Tobj (k)’ measurement minus that of the thermal camera when emissivity was 0.98 (‘at e = 0.98 d = 0.5’). ‘Ecal’ and ‘Esolve’ the calculated and *Solver* emissivity estimates respectively at the measurement indicated by the previous columns. ‘DeltaE’ the difference between calculated and *Solver* emissivity estimates at measurement indicated by the previous columns.

**Additional file 7.** Water bath estimation extracted data. The full data extracted from thermal images and the emissivity estimates from the water bath protocol. Column headings are as follows: ‘measurement’ a name identifier for each flower measurement. ‘individual within species’ the numerical identifier of individual flowers within each species numbers are shared across species, this is used for flower identification not for analysis. ‘Individual’ numerical identifier of flower individual across all species. ‘species’ the flower species. ‘measurement location’ the measurement location, here reproductive structures are indicated as ‘A: repro struc’, petal base as ‘B: petal base’, petal edge as ‘C: petal edge’. ‘Set temperature of waterbath’ the temperature to which the water bath was set to, written as ‘[temp]C’ to insure it is read as a categorical factor by R. ‘thermometer temp of waterbath’ the temperature of the water bath measured by the thermometer in degrees Celsius. ‘Tref’ the reflected temperature at the measurement indicated by previous columns. ‘IRT temp of waterbath e = 0.98, d = 0.5, rest asp per image’ the water temperature proximal to the flower as measured by the thermal camera, used to obtain Tobj. ‘at e = 1 d = 0, Temp uncorrected (for W)’ the apparent temperature at measurement indicated by the previous columns. ‘at e = 0.98 d = 0.5’ the camera’s temperature measurement, in kelvin, when emissivity was 0.98 at the measurement indicated by the previous columns. ‘DeltaTobj-T0.98’ the ‘Tobj (k)’ measurement minus that of the thermal camera when emissivity was 0.98 (‘at e = 0.98 d = 0.5’). ‘Ecal’ and ‘Esolve’ the calculated and *Solver* emissivity estimates respectively at the measurement indicated by the previous columns. ‘DeltaE’ the difference between calculated and *Solver* emissivity estimates at measurement indicated by the previous columns.

**Additional file 8.** Thermocouple estimation IR images. File containing the thermal imaging (and paired photographs) of all images used in data collection for the thermocouple protocol. Images are sorted by species and then by individual flower, flower file names are formatted as [flower identifier used for sorting e.g. ‘D’][number].

**Additional file 9. **Water bath estimation IR images. File containing the thermal imaging (and paired photographs) of all images used in data collection for the water bath protocol. Images are sorted by species, set temperature of the water bath and then by individual flower, flower file names are formatted as [flower identifier used for sorting e.g. ‘D’][number]. Additionally, where available, photos are provided to aid identification of individual flowers.

## Data Availability

All data generated or analysed during this study are included within the additional files associated with this article. Emissivity estimate values, along with the object and apparent temperature values extracted from IR images are available in Additional files 6 and 7 for the thermocouple and water bath estimates respectively. IR images are available in Additional files 8 and 9 for the thermocouple and water bath estimates respectively.
