# Supplementary figures and images for: Floral infrared emissivity estimates using simple tools (part 1 of 4)
Source: Plant Methods. 2021 Feb 25;17:23. doi: 10.1186/s13007-021-00721-w (PMC7905901; doi:10.1186/s13007-021-00721-w)

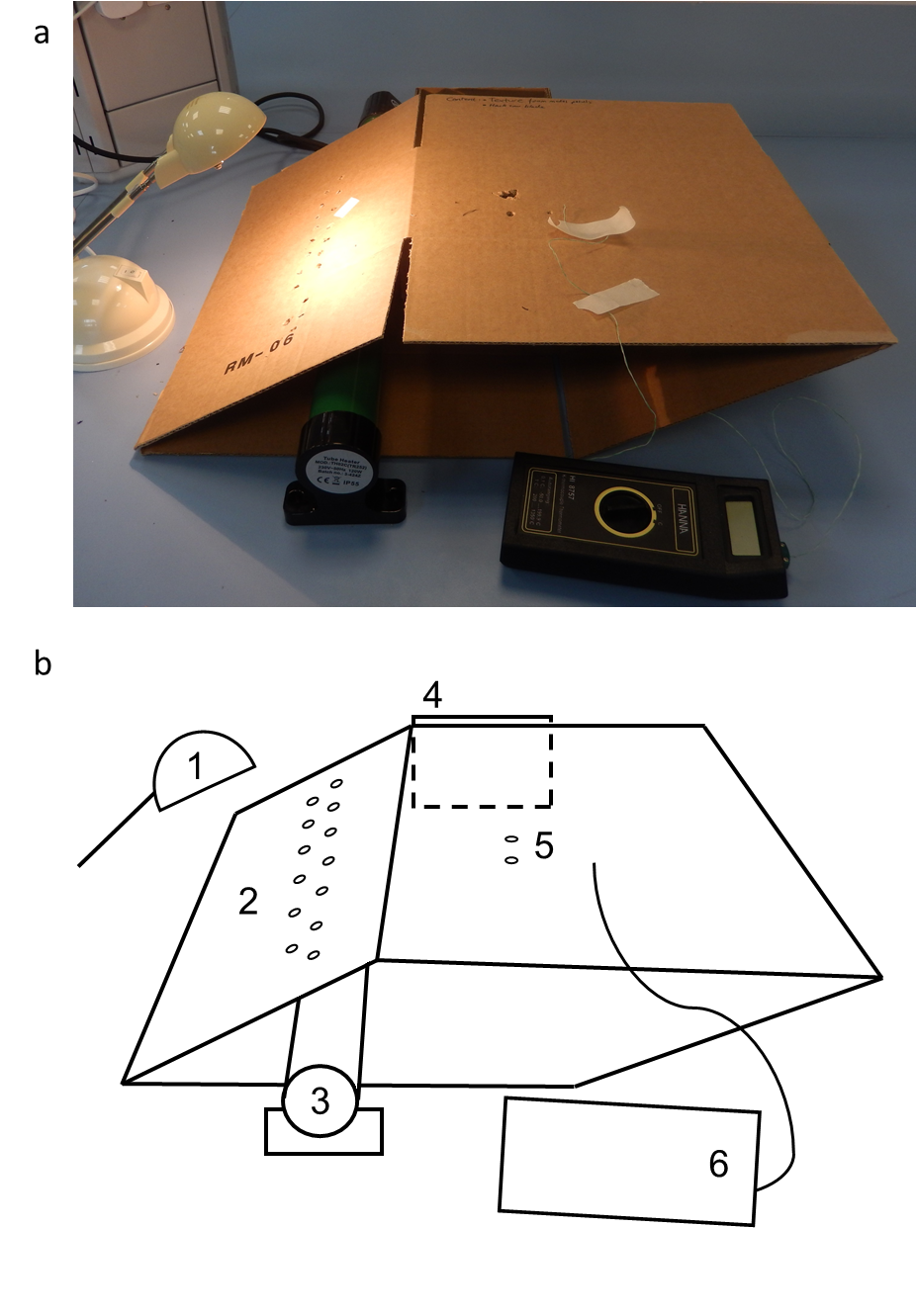

Supplement: Supplementary file 1 — Additional file 1. A photograph (a) and matching diagrammatic image (b) of the stand constructed for thermocouple estimates. Numbers on b indicate: 1) the desk lamp which aids flower heating; 2) the stand with holes above the greenhouse heater where flowers would be placed for heating; 3) the green house heater; 4) The stand’s support (not visible in a); 5) holes where flowers would be place for thermocouple measurement, there are two sizes to accommodate different flower sizes; 6) the thermocouple itself. [file 13007_2021_721_MOESM1_ESM.png]

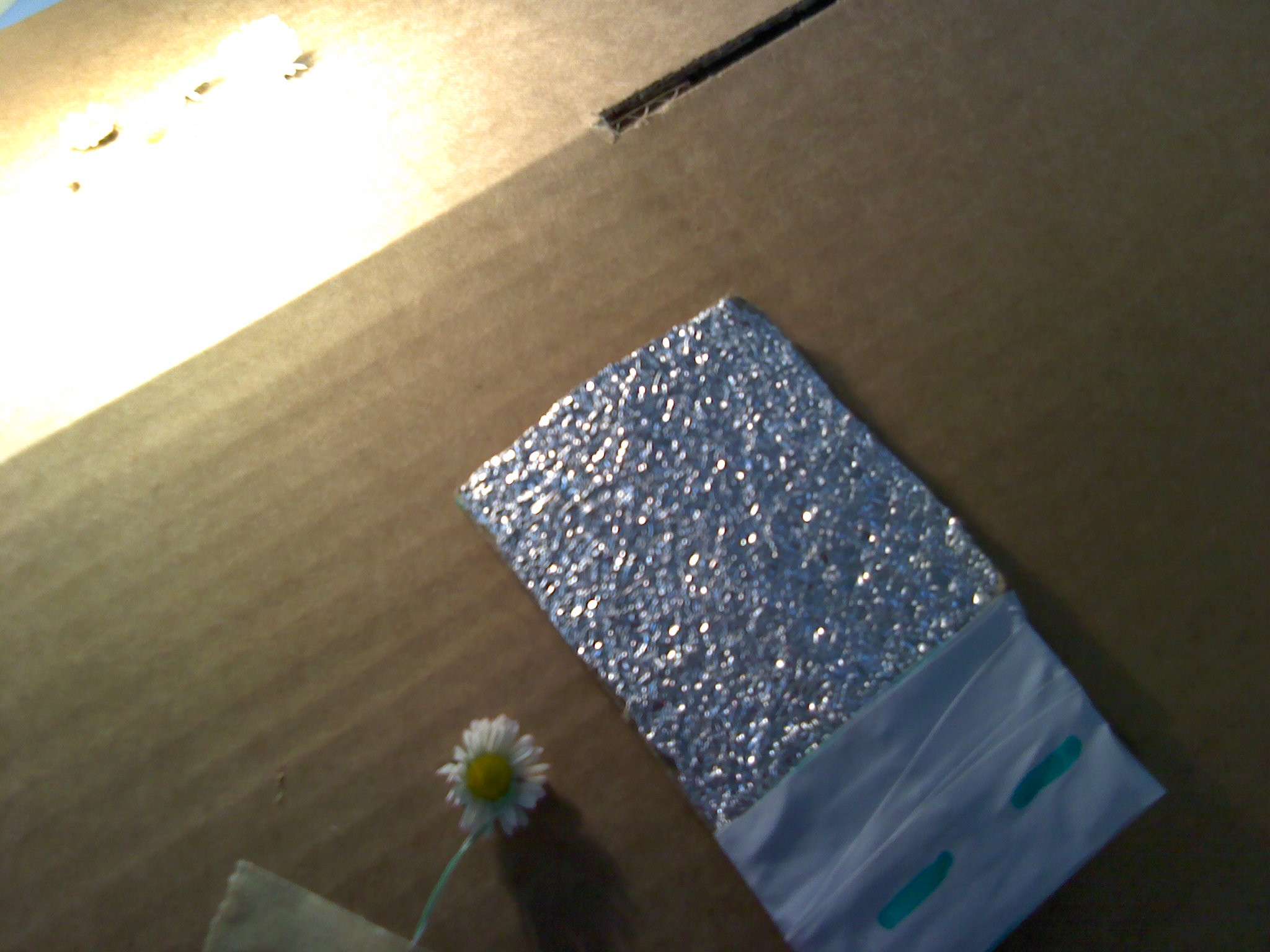

Supplement: Supplementary file 8 — Additional file 8. Thermocouple estimation IR images. File containing the thermal imaging (and paired photographs) of all images used in data collection for the thermocouple protocol. Images are sorted by species and then by individual flower, flower file names are formatted as [flower identifier used for sorting e.g. ‘D’][number]. [file 13007_2021_721_MOESM8_ESM.zip › Thermocouple IR images/Bellis/D1/DC_4874.jpg]

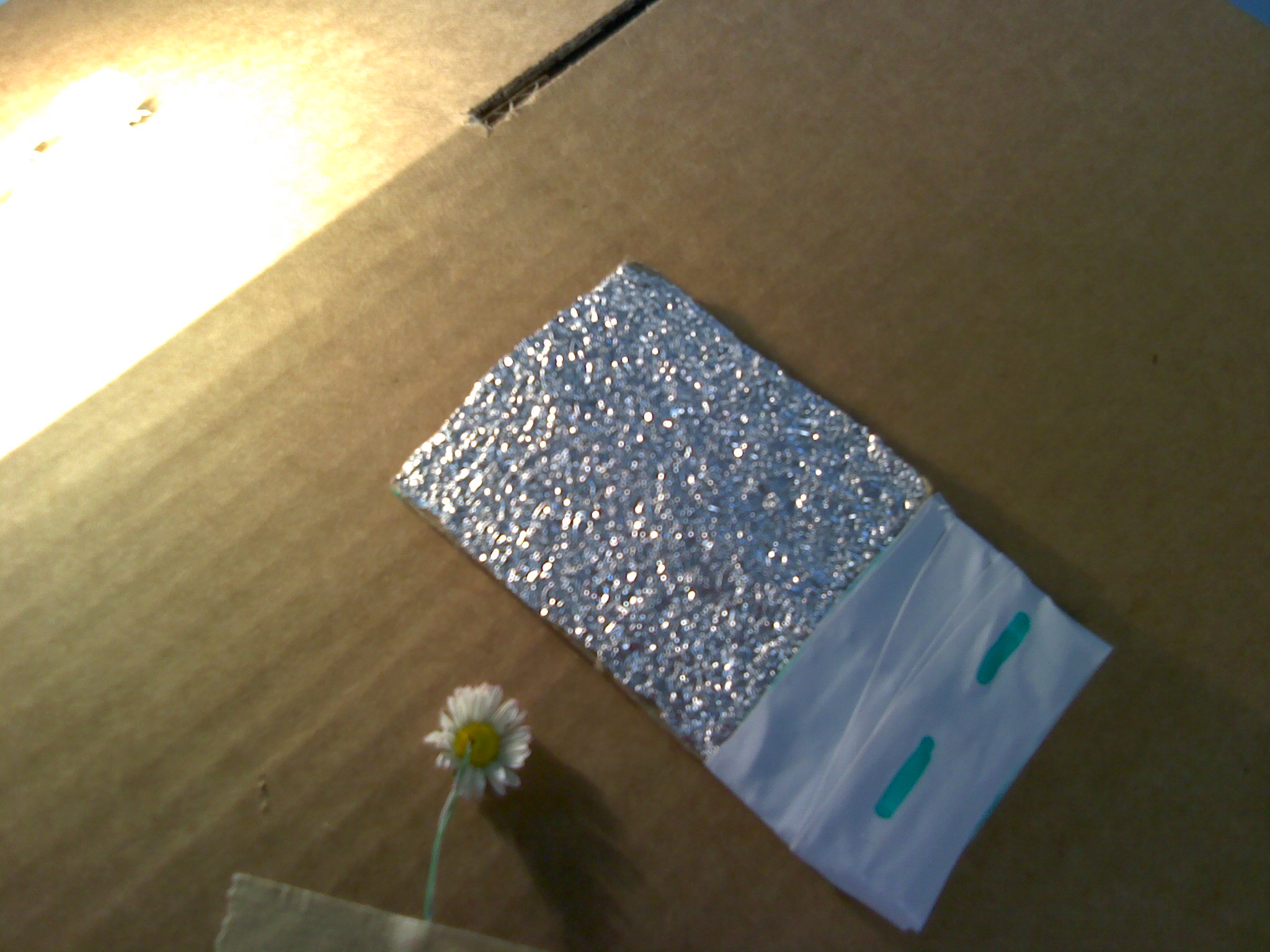

Supplement: Supplementary file 8 — Additional file 8. Thermocouple estimation IR images. File containing the thermal imaging (and paired photographs) of all images used in data collection for the thermocouple protocol. Images are sorted by species and then by individual flower, flower file names are formatted as [flower identifier used for sorting e.g. ‘D’][number]. [file 13007_2021_721_MOESM8_ESM.zip › Thermocouple IR images/Bellis/D1/DC_4876.jpg]

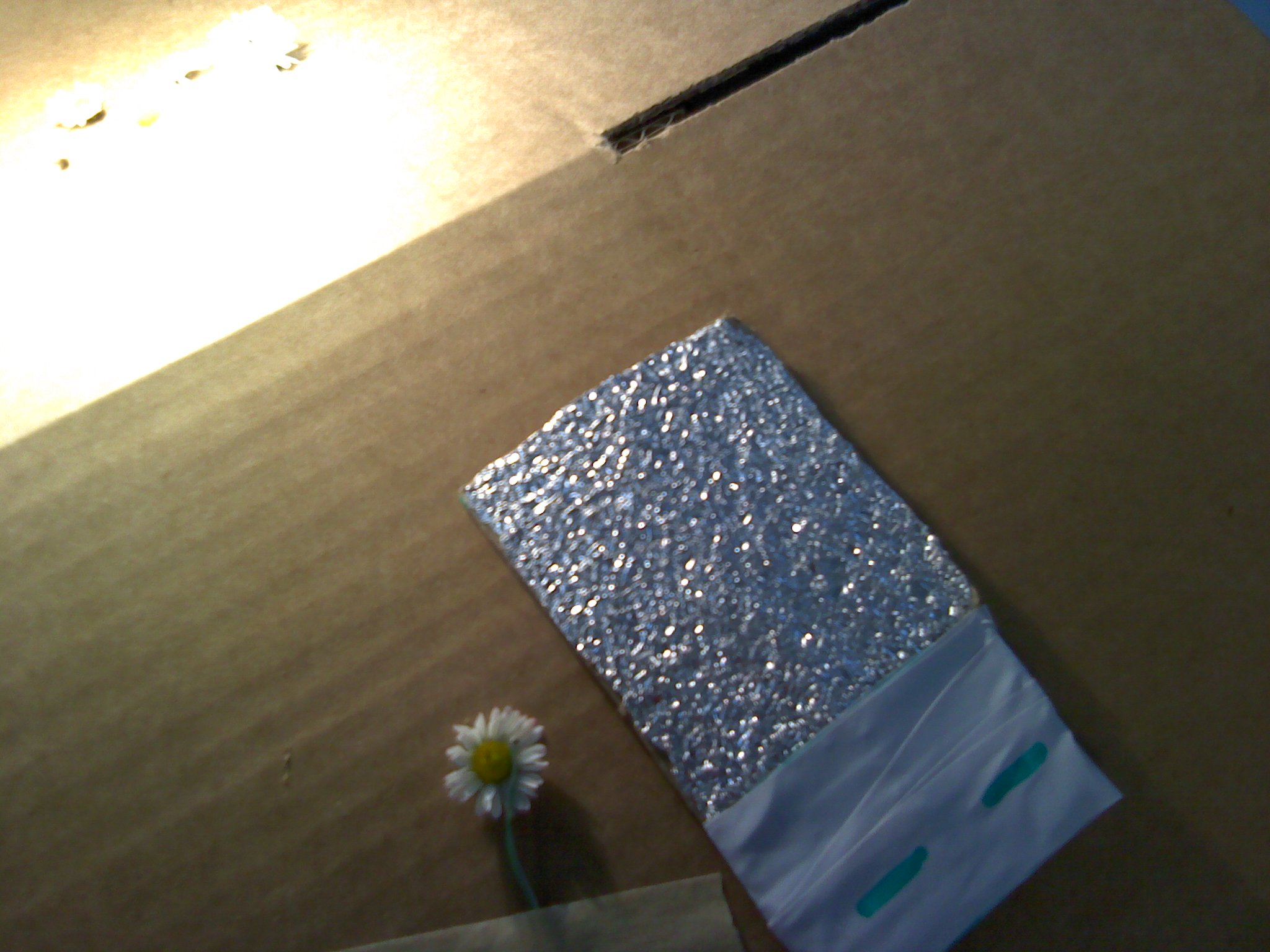

Supplement: Supplementary file 8 — Additional file 8. Thermocouple estimation IR images. File containing the thermal imaging (and paired photographs) of all images used in data collection for the thermocouple protocol. Images are sorted by species and then by individual flower, flower file names are formatted as [flower identifier used for sorting e.g. ‘D’][number]. [file 13007_2021_721_MOESM8_ESM.zip › Thermocouple IR images/Bellis/D1/DC_4878.jpg]

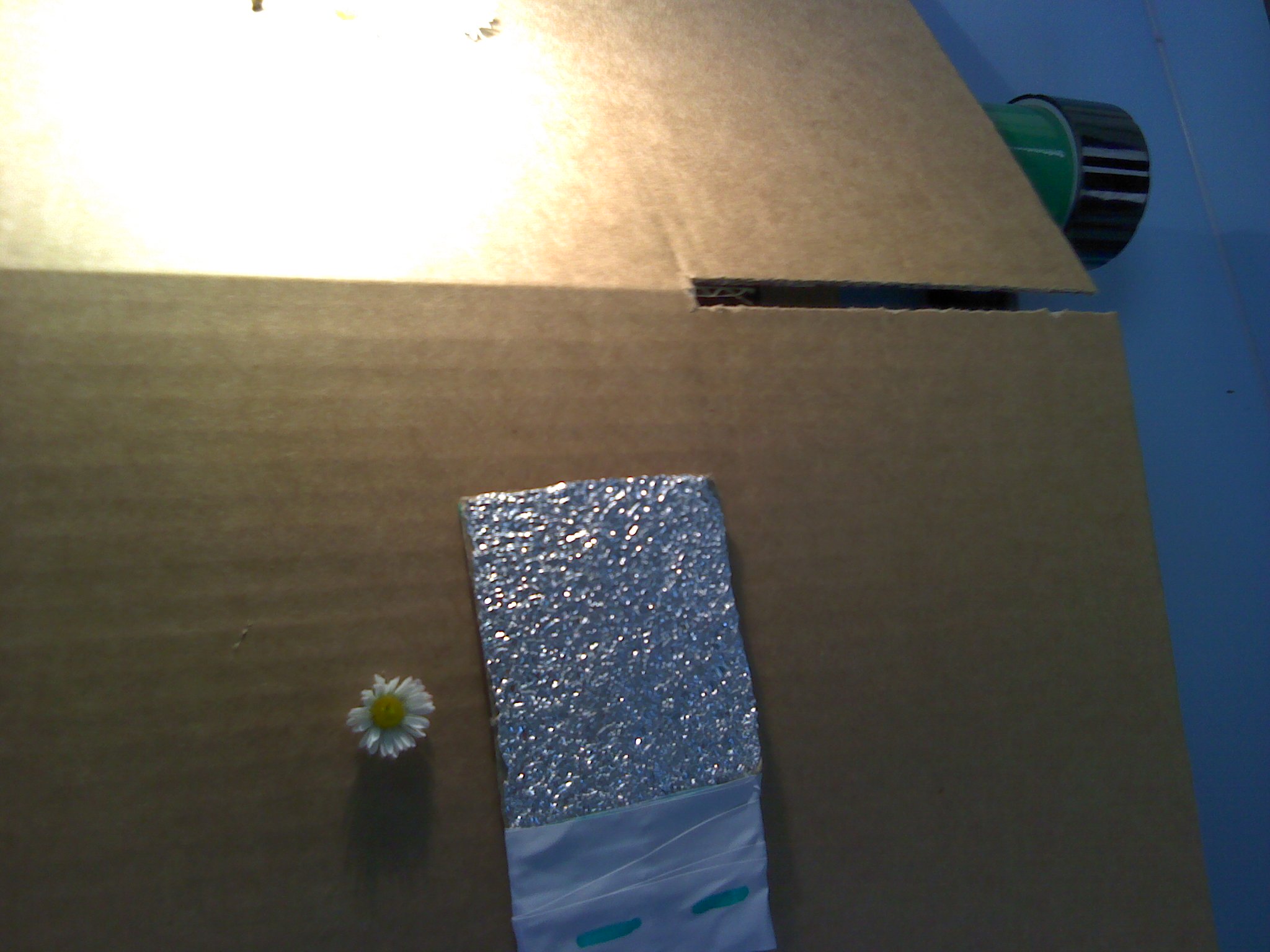

Supplement: Supplementary file 8 — Additional file 8. Thermocouple estimation IR images. File containing the thermal imaging (and paired photographs) of all images used in data collection for the thermocouple protocol. Images are sorted by species and then by individual flower, flower file names are formatted as [flower identifier used for sorting e.g. ‘D’][number]. [file 13007_2021_721_MOESM8_ESM.zip › Thermocouple IR images/Bellis/D1/DC_4880.jpg]

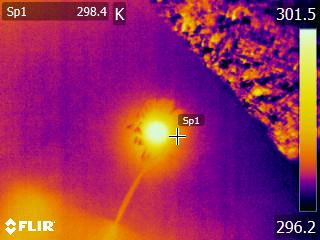

Supplement: Supplementary file 8 — Additional file 8. Thermocouple estimation IR images. File containing the thermal imaging (and paired photographs) of all images used in data collection for the thermocouple protocol. Images are sorted by species and then by individual flower, flower file names are formatted as [flower identifier used for sorting e.g. ‘D’][number]. [file 13007_2021_721_MOESM8_ESM.zip › Thermocouple IR images/Bellis/D1/IR_4873.jpg]

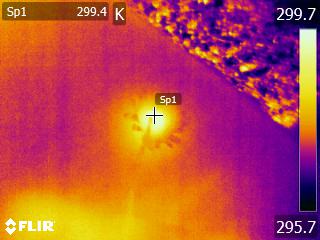

Supplement: Supplementary file 8 — Additional file 8. Thermocouple estimation IR images. File containing the thermal imaging (and paired photographs) of all images used in data collection for the thermocouple protocol. Images are sorted by species and then by individual flower, flower file names are formatted as [flower identifier used for sorting e.g. ‘D’][number]. [file 13007_2021_721_MOESM8_ESM.zip › Thermocouple IR images/Bellis/D1/IR_4875.jpg]

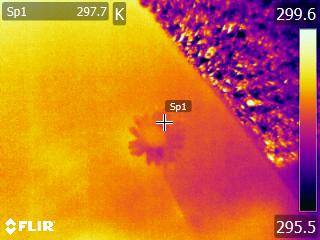

Supplement: Supplementary file 8 — Additional file 8. Thermocouple estimation IR images. File containing the thermal imaging (and paired photographs) of all images used in data collection for the thermocouple protocol. Images are sorted by species and then by individual flower, flower file names are formatted as [flower identifier used for sorting e.g. ‘D’][number]. [file 13007_2021_721_MOESM8_ESM.zip › Thermocouple IR images/Bellis/D1/IR_4877.jpg]

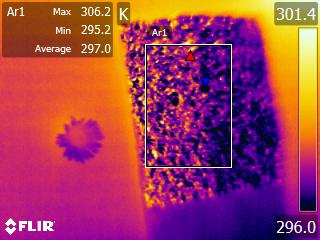

Supplement: Supplementary file 8 — Additional file 8. Thermocouple estimation IR images. File containing the thermal imaging (and paired photographs) of all images used in data collection for the thermocouple protocol. Images are sorted by species and then by individual flower, flower file names are formatted as [flower identifier used for sorting e.g. ‘D’][number]. [file 13007_2021_721_MOESM8_ESM.zip › Thermocouple IR images/Bellis/D1/IR_4879.jpg]

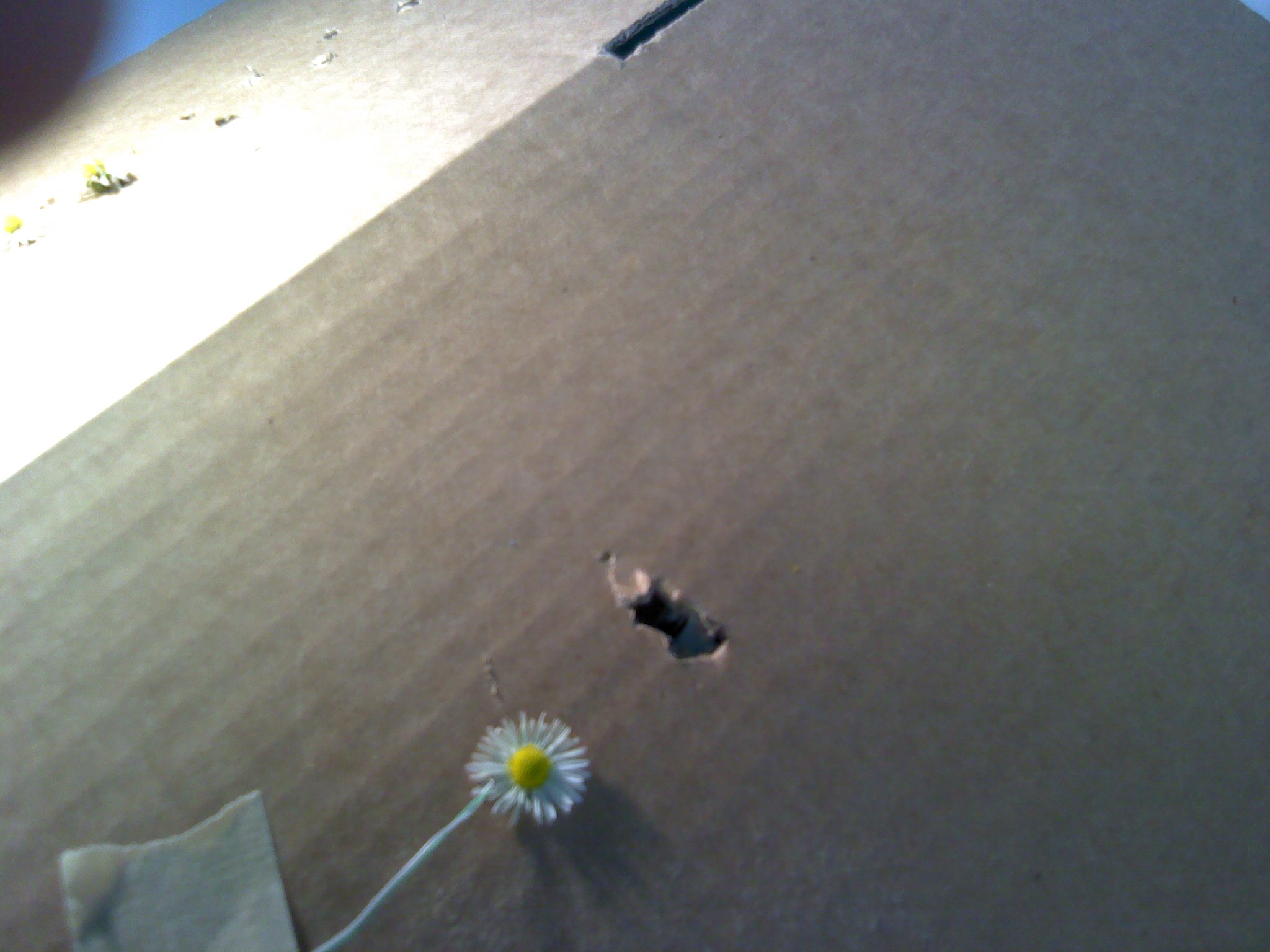

Supplement: Supplementary file 8 — Additional file 8. Thermocouple estimation IR images. File containing the thermal imaging (and paired photographs) of all images used in data collection for the thermocouple protocol. Images are sorted by species and then by individual flower, flower file names are formatted as [flower identifier used for sorting e.g. ‘D’][number]. [file 13007_2021_721_MOESM8_ESM.zip › Thermocouple IR images/Bellis/D10/DC_42388.jpg]

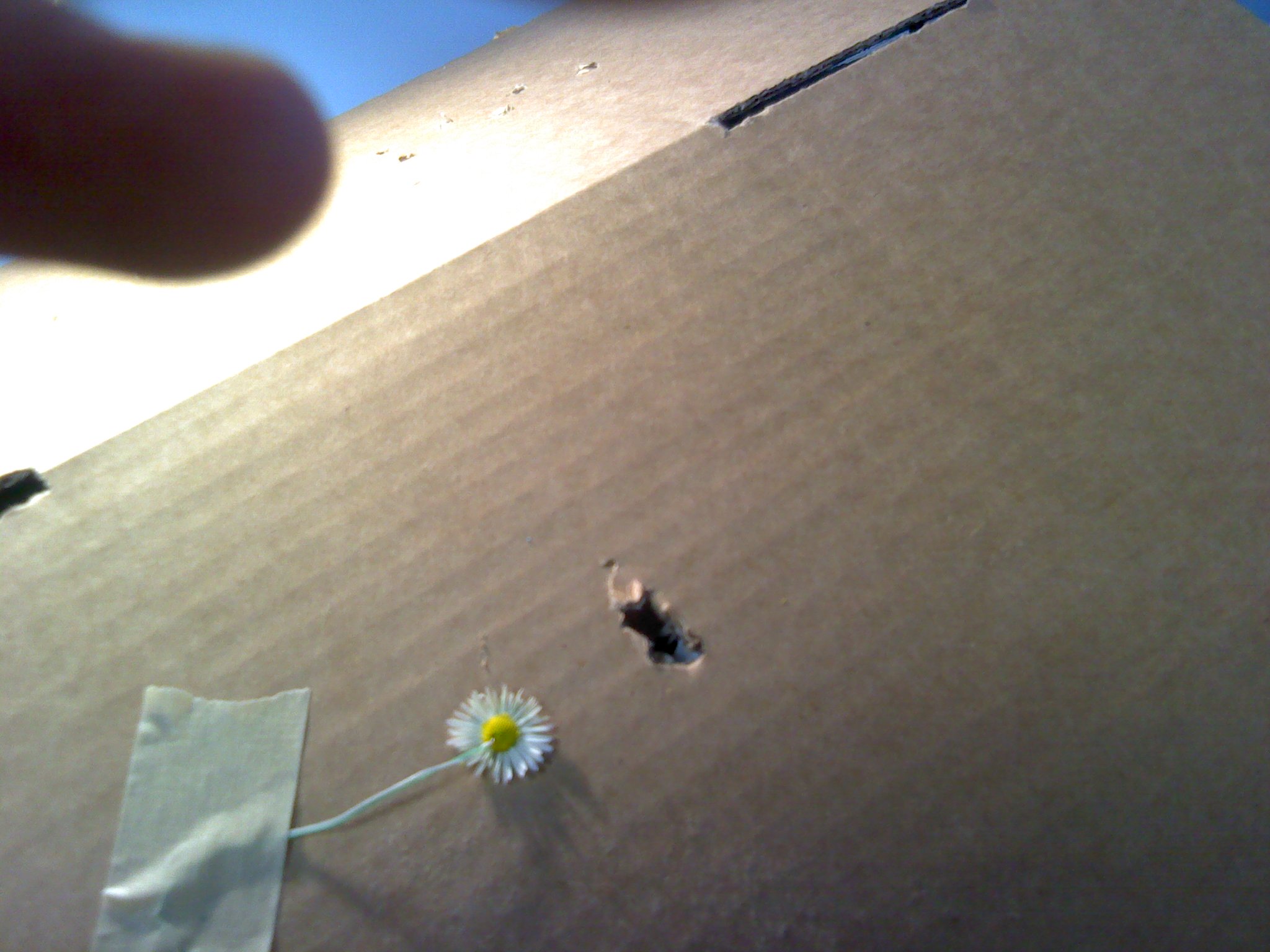

Supplement: Supplementary file 8 — Additional file 8. Thermocouple estimation IR images. File containing the thermal imaging (and paired photographs) of all images used in data collection for the thermocouple protocol. Images are sorted by species and then by individual flower, flower file names are formatted as [flower identifier used for sorting e.g. ‘D’][number]. [file 13007_2021_721_MOESM8_ESM.zip › Thermocouple IR images/Bellis/D10/DC_42390.jpg]

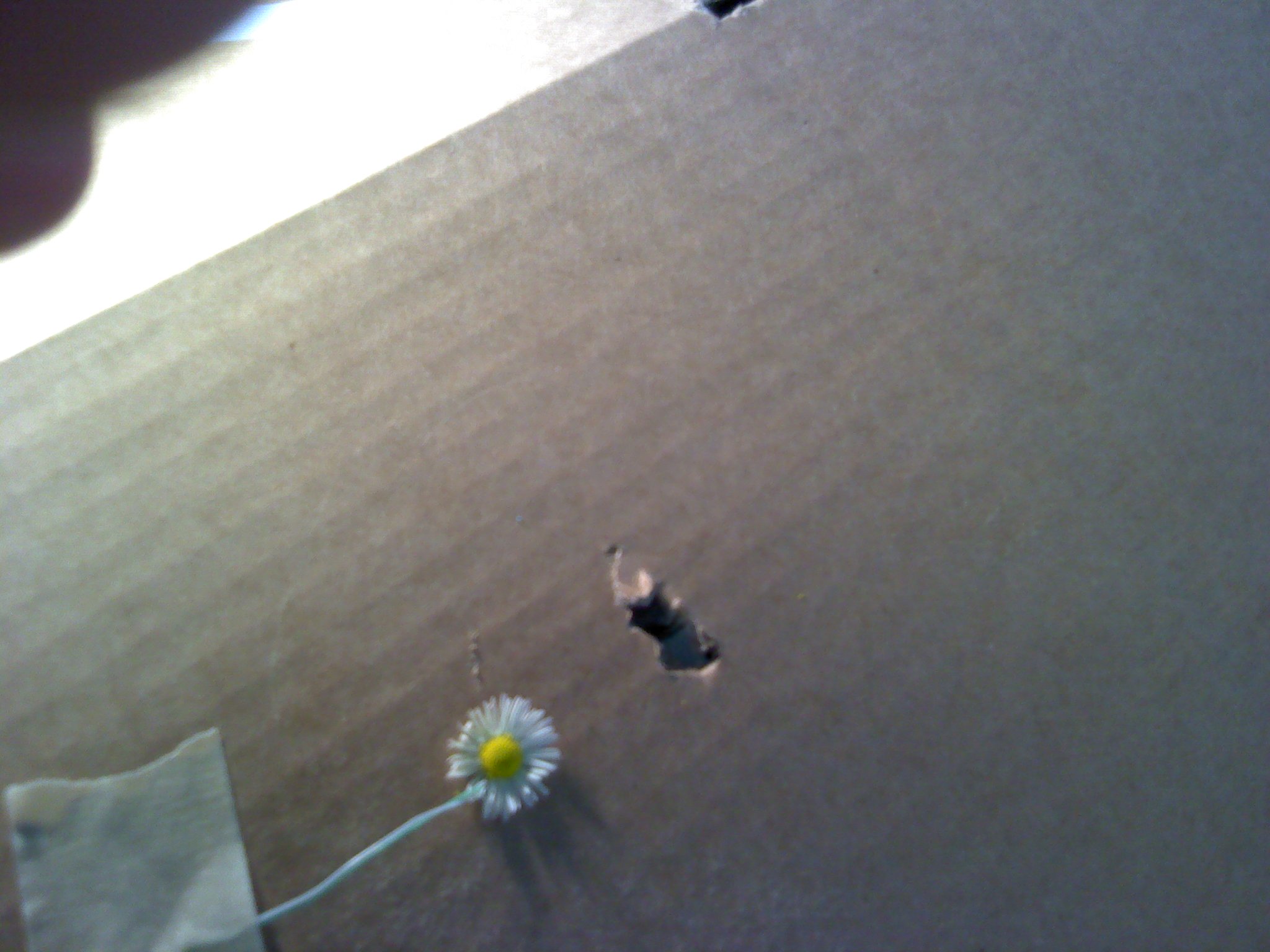

Supplement: Supplementary file 8 — Additional file 8. Thermocouple estimation IR images. File containing the thermal imaging (and paired photographs) of all images used in data collection for the thermocouple protocol. Images are sorted by species and then by individual flower, flower file names are formatted as [flower identifier used for sorting e.g. ‘D’][number]. [file 13007_2021_721_MOESM8_ESM.zip › Thermocouple IR images/Bellis/D10/DC_42392.jpg]

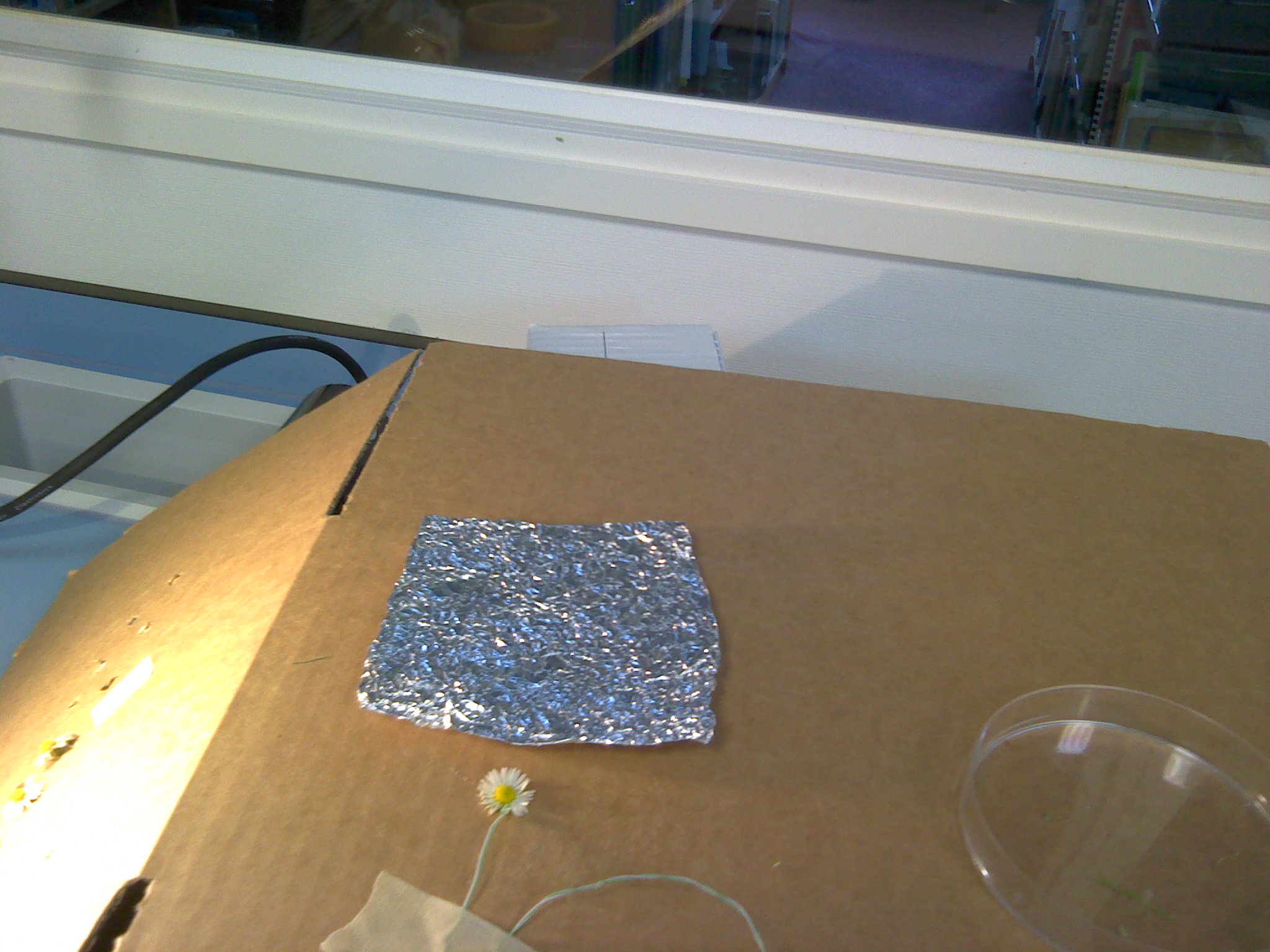

Supplement: Supplementary file 8 — Additional file 8. Thermocouple estimation IR images. File containing the thermal imaging (and paired photographs) of all images used in data collection for the thermocouple protocol. Images are sorted by species and then by individual flower, flower file names are formatted as [flower identifier used for sorting e.g. ‘D’][number]. [file 13007_2021_721_MOESM8_ESM.zip › Thermocouple IR images/Bellis/D10/DC_42394.jpg]

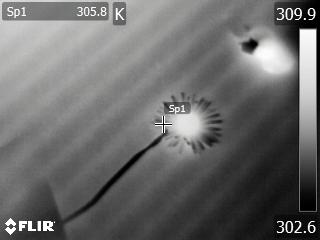

Supplement: Supplementary file 8 — Additional file 8. Thermocouple estimation IR images. File containing the thermal imaging (and paired photographs) of all images used in data collection for the thermocouple protocol. Images are sorted by species and then by individual flower, flower file names are formatted as [flower identifier used for sorting e.g. ‘D’][number]. [file 13007_2021_721_MOESM8_ESM.zip › Thermocouple IR images/Bellis/D10/IR_42387.jpg]

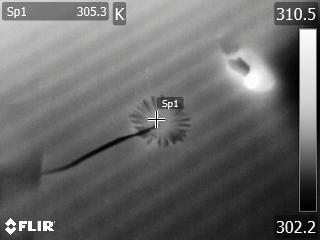

Supplement: Supplementary file 8 — Additional file 8. Thermocouple estimation IR images. File containing the thermal imaging (and paired photographs) of all images used in data collection for the thermocouple protocol. Images are sorted by species and then by individual flower, flower file names are formatted as [flower identifier used for sorting e.g. ‘D’][number]. [file 13007_2021_721_MOESM8_ESM.zip › Thermocouple IR images/Bellis/D10/IR_42389.jpg]

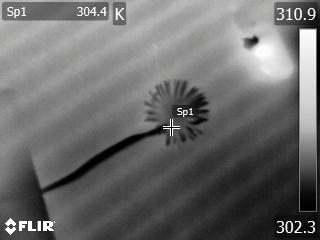

Supplement: Supplementary file 8 — Additional file 8. Thermocouple estimation IR images. File containing the thermal imaging (and paired photographs) of all images used in data collection for the thermocouple protocol. Images are sorted by species and then by individual flower, flower file names are formatted as [flower identifier used for sorting e.g. ‘D’][number]. [file 13007_2021_721_MOESM8_ESM.zip › Thermocouple IR images/Bellis/D10/IR_42391.jpg]

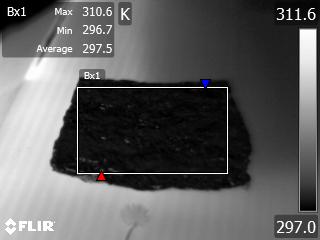

Supplement: Supplementary file 8 — Additional file 8. Thermocouple estimation IR images. File containing the thermal imaging (and paired photographs) of all images used in data collection for the thermocouple protocol. Images are sorted by species and then by individual flower, flower file names are formatted as [flower identifier used for sorting e.g. ‘D’][number]. [file 13007_2021_721_MOESM8_ESM.zip › Thermocouple IR images/Bellis/D10/IR_42393.jpg]

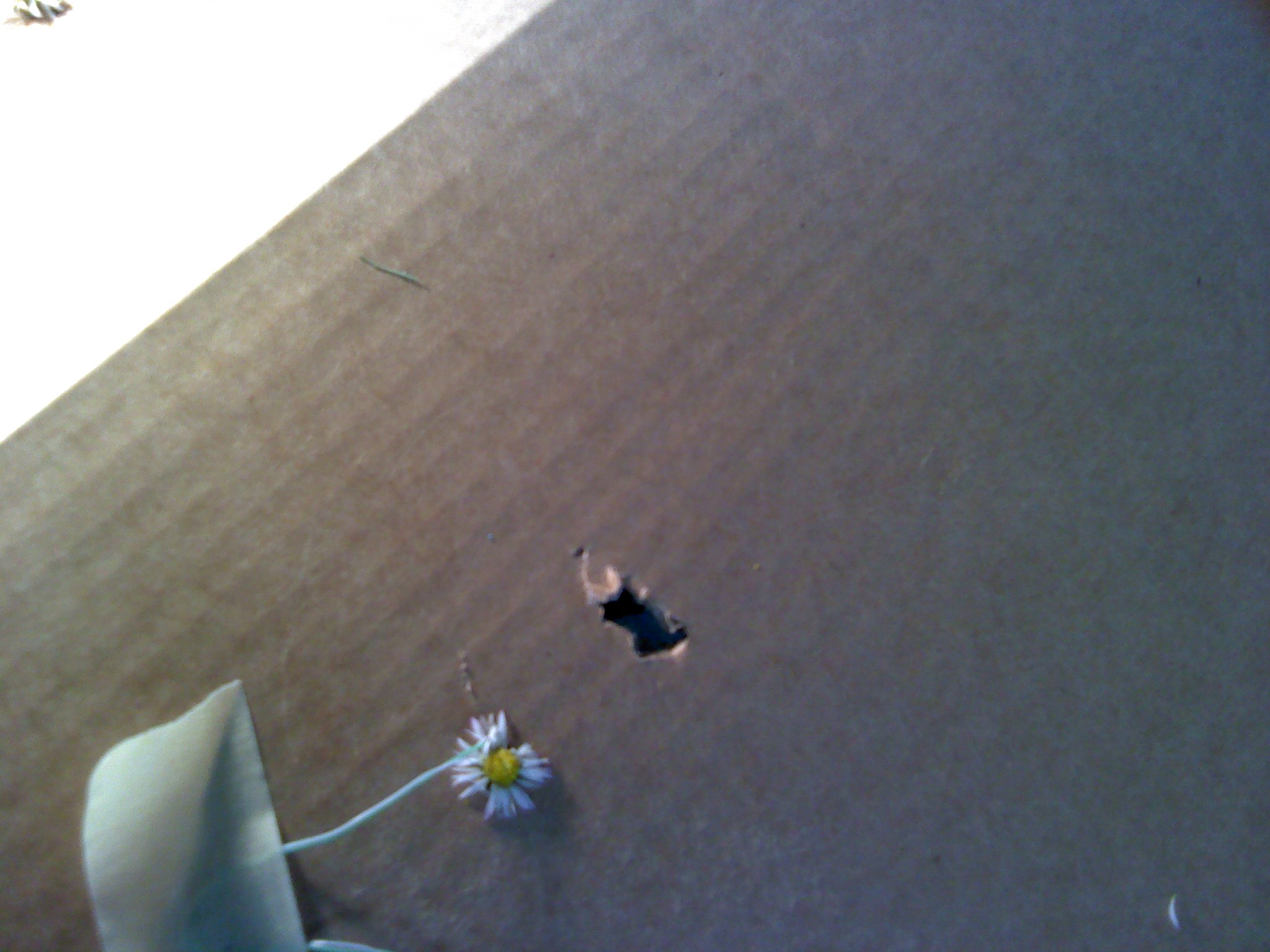

Supplement: Supplementary file 8 — Additional file 8. Thermocouple estimation IR images. File containing the thermal imaging (and paired photographs) of all images used in data collection for the thermocouple protocol. Images are sorted by species and then by individual flower, flower file names are formatted as [flower identifier used for sorting e.g. ‘D’][number]. [file 13007_2021_721_MOESM8_ESM.zip › Thermocouple IR images/Bellis/D11/DC_42396.jpg]

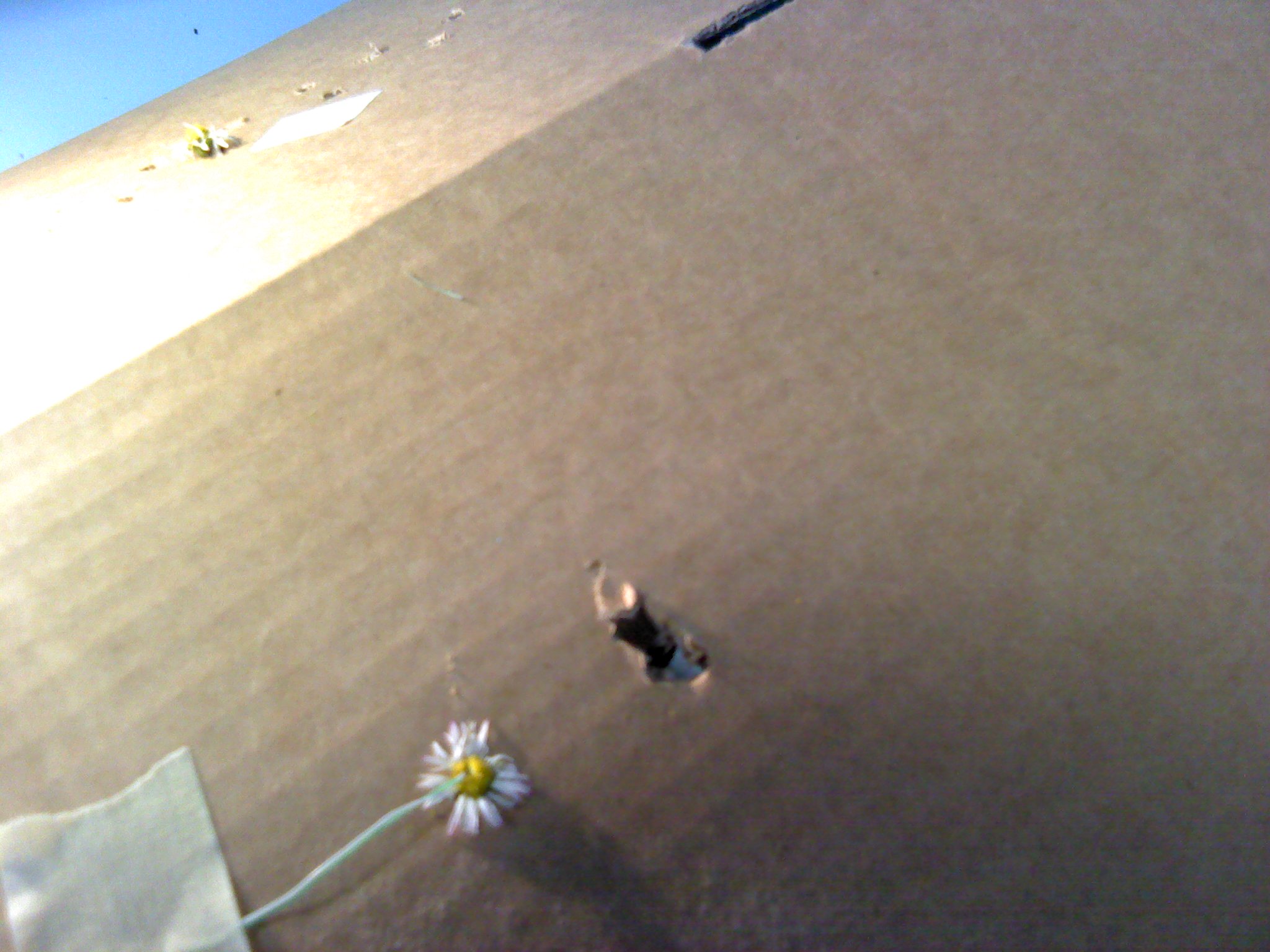

Supplement: Supplementary file 8 — Additional file 8. Thermocouple estimation IR images. File containing the thermal imaging (and paired photographs) of all images used in data collection for the thermocouple protocol. Images are sorted by species and then by individual flower, flower file names are formatted as [flower identifier used for sorting e.g. ‘D’][number]. [file 13007_2021_721_MOESM8_ESM.zip › Thermocouple IR images/Bellis/D11/DC_42398.jpg]

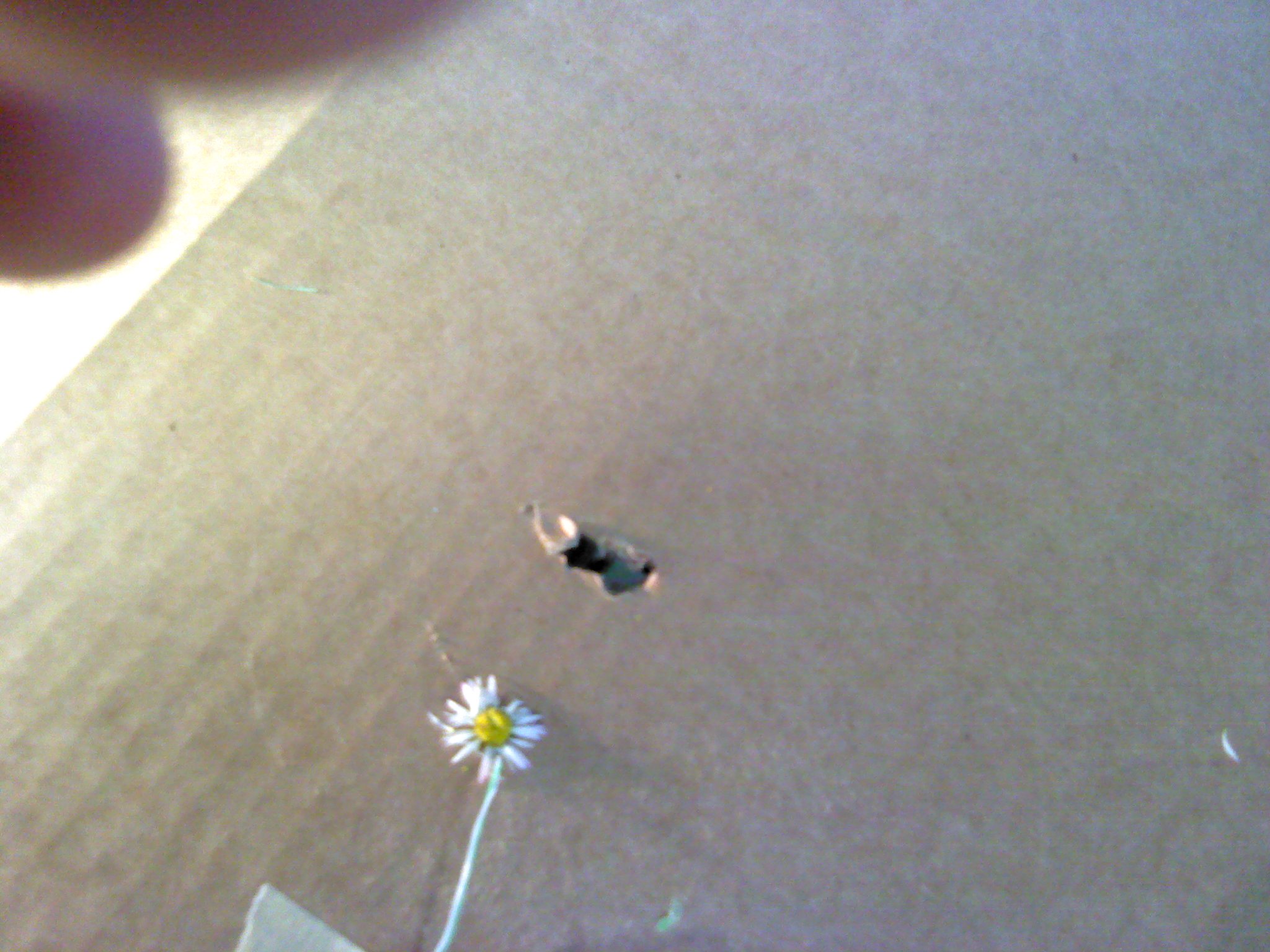

Supplement: Supplementary file 8 — Additional file 8. Thermocouple estimation IR images. File containing the thermal imaging (and paired photographs) of all images used in data collection for the thermocouple protocol. Images are sorted by species and then by individual flower, flower file names are formatted as [flower identifier used for sorting e.g. ‘D’][number]. [file 13007_2021_721_MOESM8_ESM.zip › Thermocouple IR images/Bellis/D11/DC_42400.jpg]

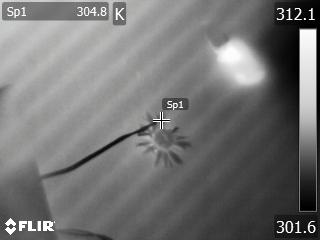

Supplement: Supplementary file 8 — Additional file 8. Thermocouple estimation IR images. File containing the thermal imaging (and paired photographs) of all images used in data collection for the thermocouple protocol. Images are sorted by species and then by individual flower, flower file names are formatted as [flower identifier used for sorting e.g. ‘D’][number]. [file 13007_2021_721_MOESM8_ESM.zip › Thermocouple IR images/Bellis/D11/IR_42395.jpg]

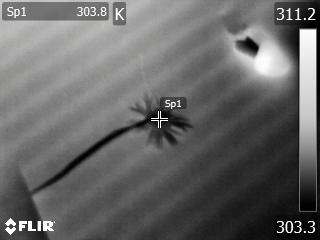

Supplement: Supplementary file 8 — Additional file 8. Thermocouple estimation IR images. File containing the thermal imaging (and paired photographs) of all images used in data collection for the thermocouple protocol. Images are sorted by species and then by individual flower, flower file names are formatted as [flower identifier used for sorting e.g. ‘D’][number]. [file 13007_2021_721_MOESM8_ESM.zip › Thermocouple IR images/Bellis/D11/IR_42397.jpg]

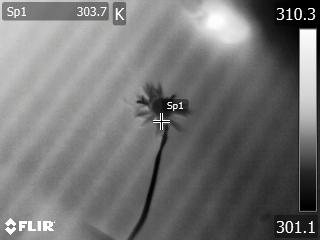

Supplement: Supplementary file 8 — Additional file 8. Thermocouple estimation IR images. File containing the thermal imaging (and paired photographs) of all images used in data collection for the thermocouple protocol. Images are sorted by species and then by individual flower, flower file names are formatted as [flower identifier used for sorting e.g. ‘D’][number]. [file 13007_2021_721_MOESM8_ESM.zip › Thermocouple IR images/Bellis/D11/IR_42399.jpg]

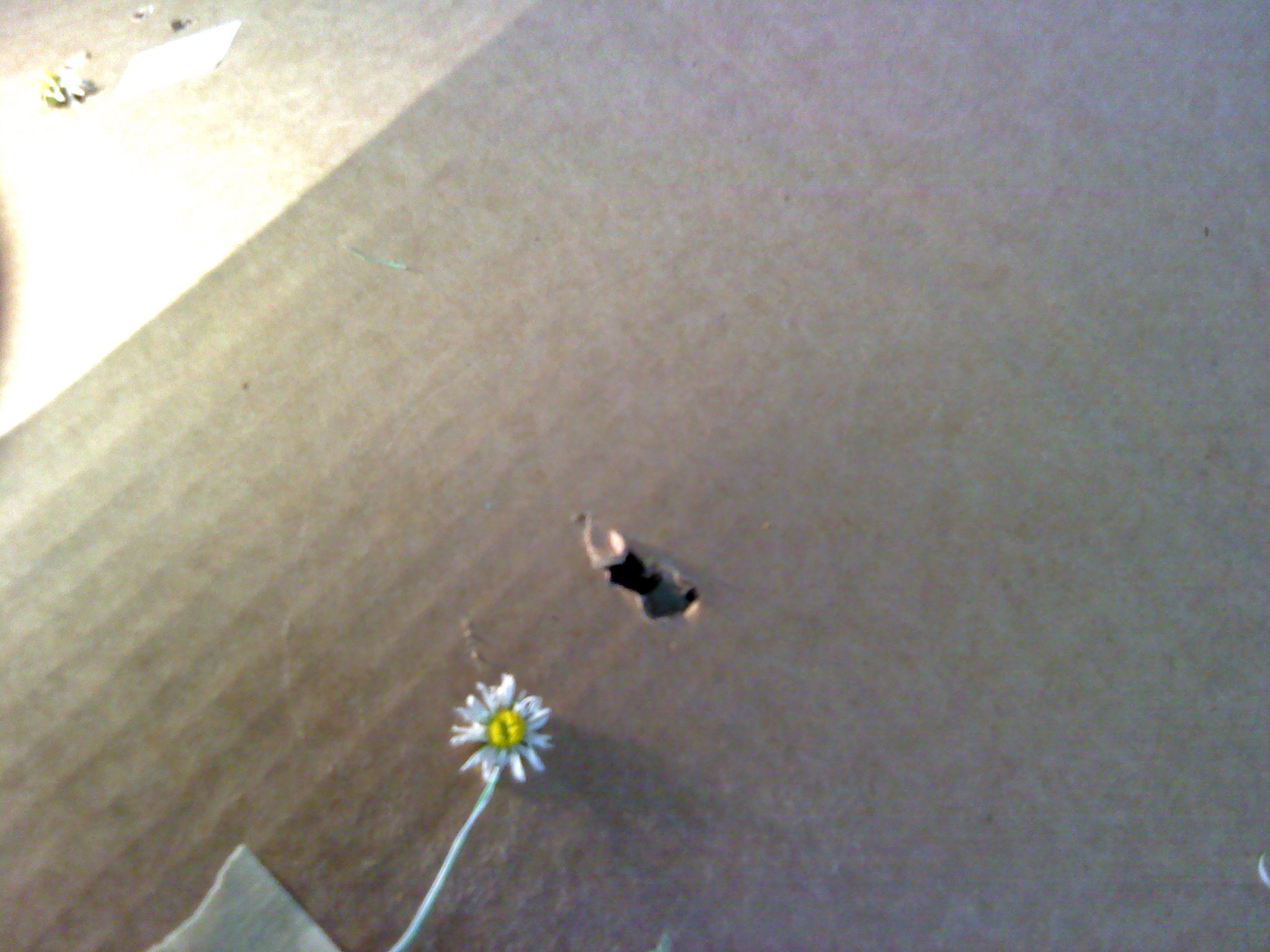

Supplement: Supplementary file 8 — Additional file 8. Thermocouple estimation IR images. File containing the thermal imaging (and paired photographs) of all images used in data collection for the thermocouple protocol. Images are sorted by species and then by individual flower, flower file names are formatted as [flower identifier used for sorting e.g. ‘D’][number]. [file 13007_2021_721_MOESM8_ESM.zip › Thermocouple IR images/Bellis/D12/DC_42402.jpg]

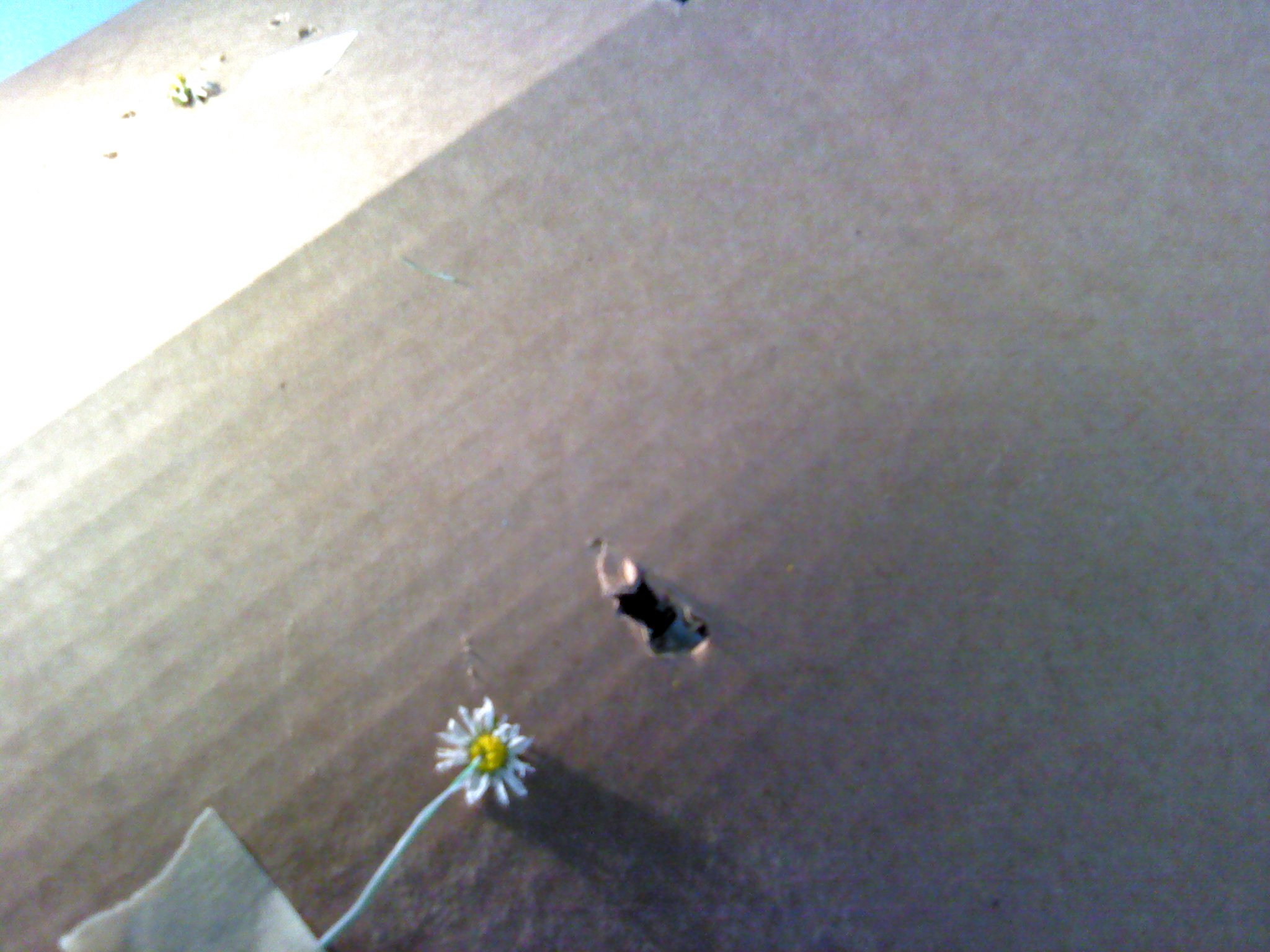

Supplement: Supplementary file 8 — Additional file 8. Thermocouple estimation IR images. File containing the thermal imaging (and paired photographs) of all images used in data collection for the thermocouple protocol. Images are sorted by species and then by individual flower, flower file names are formatted as [flower identifier used for sorting e.g. ‘D’][number]. [file 13007_2021_721_MOESM8_ESM.zip › Thermocouple IR images/Bellis/D12/DC_42404.jpg]

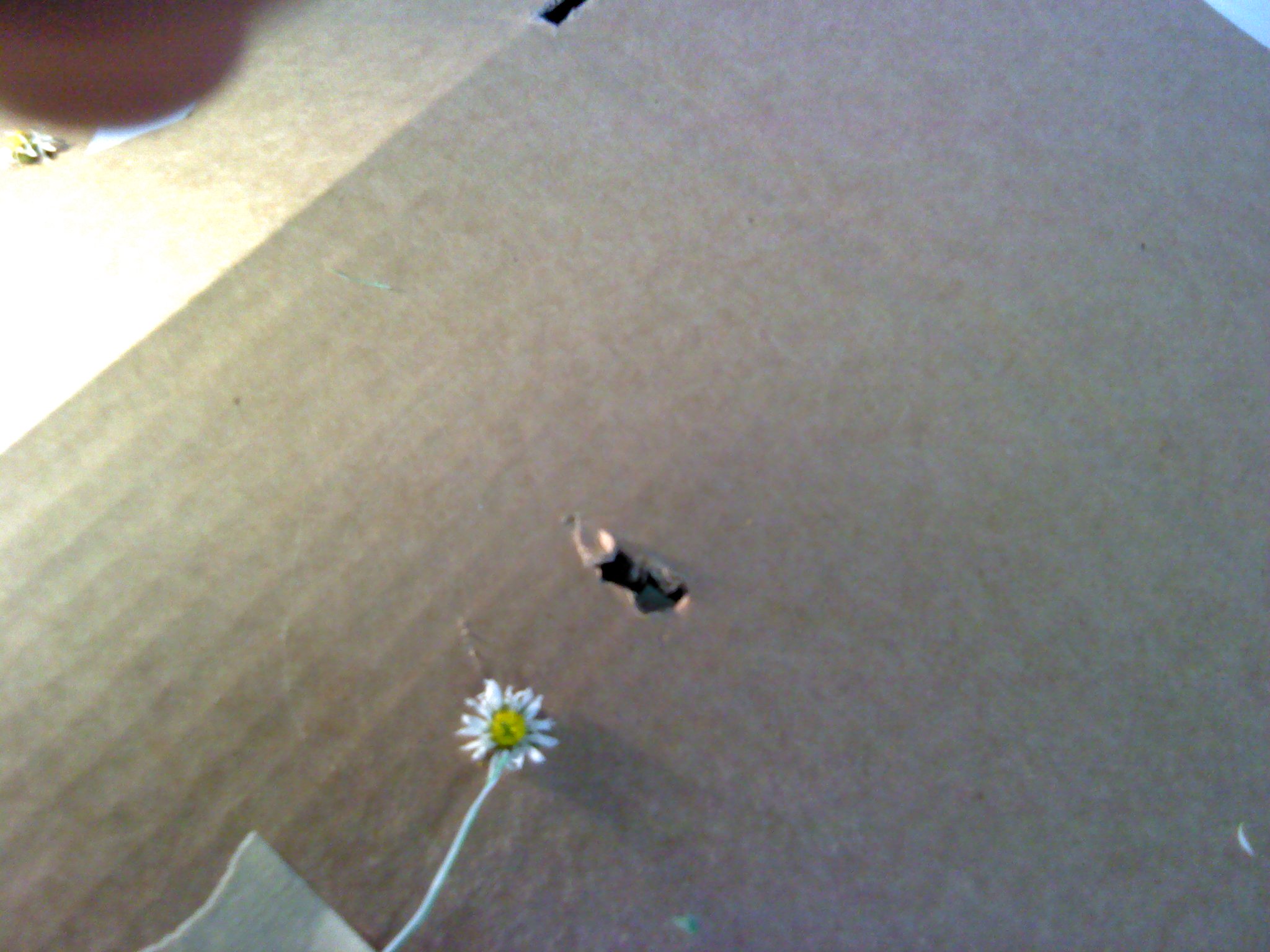

Supplement: Supplementary file 8 — Additional file 8. Thermocouple estimation IR images. File containing the thermal imaging (and paired photographs) of all images used in data collection for the thermocouple protocol. Images are sorted by species and then by individual flower, flower file names are formatted as [flower identifier used for sorting e.g. ‘D’][number]. [file 13007_2021_721_MOESM8_ESM.zip › Thermocouple IR images/Bellis/D12/DC_42406.jpg]

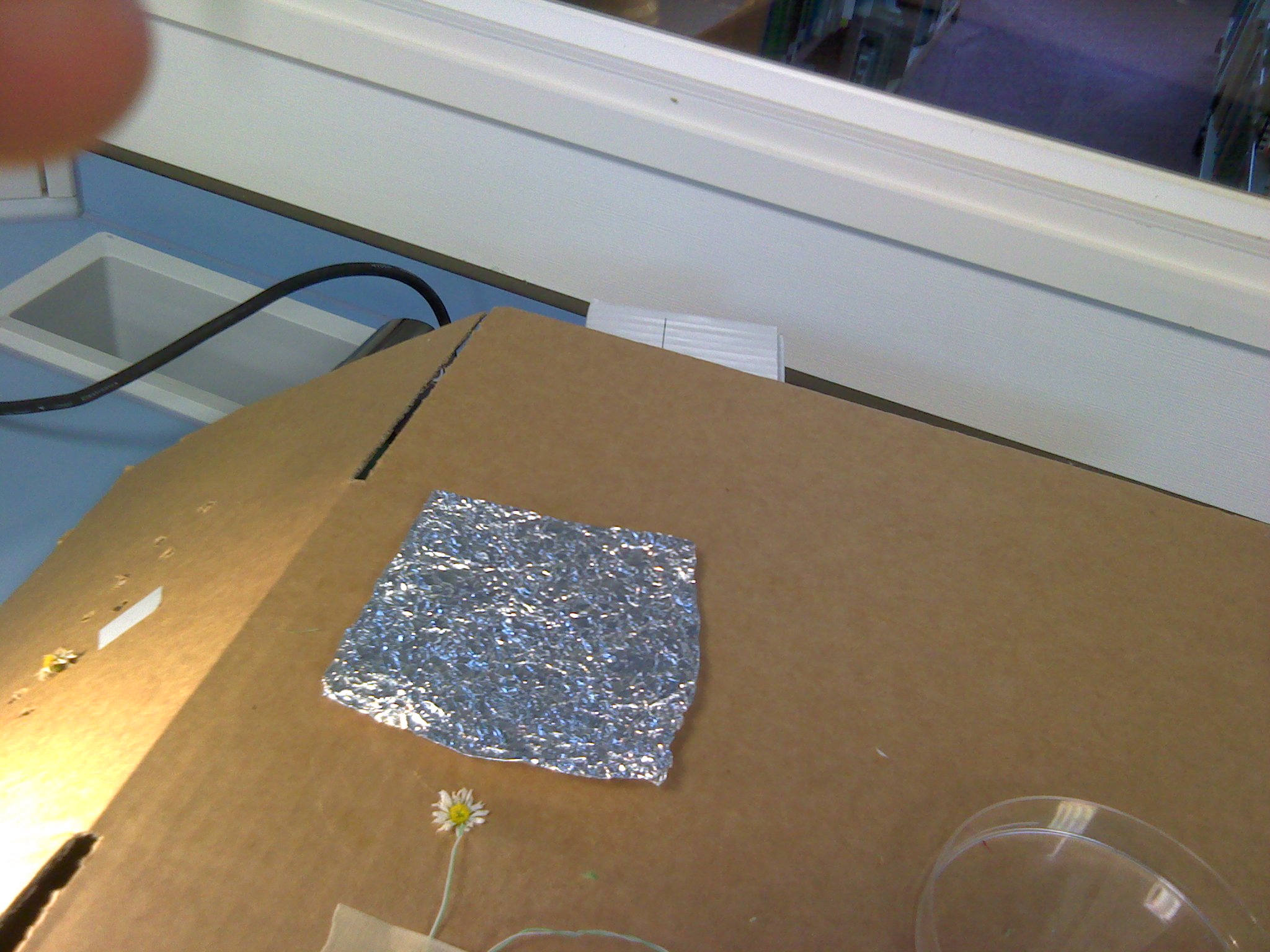

Supplement: Supplementary file 8 — Additional file 8. Thermocouple estimation IR images. File containing the thermal imaging (and paired photographs) of all images used in data collection for the thermocouple protocol. Images are sorted by species and then by individual flower, flower file names are formatted as [flower identifier used for sorting e.g. ‘D’][number]. [file 13007_2021_721_MOESM8_ESM.zip › Thermocouple IR images/Bellis/D12/DC_42408.jpg]

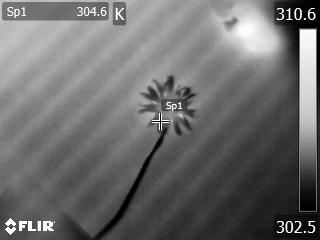

Supplement: Supplementary file 8 — Additional file 8. Thermocouple estimation IR images. File containing the thermal imaging (and paired photographs) of all images used in data collection for the thermocouple protocol. Images are sorted by species and then by individual flower, flower file names are formatted as [flower identifier used for sorting e.g. ‘D’][number]. [file 13007_2021_721_MOESM8_ESM.zip › Thermocouple IR images/Bellis/D12/IR_42401.jpg]

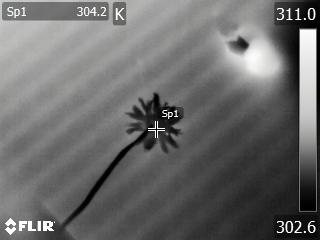

Supplement: Supplementary file 8 — Additional file 8. Thermocouple estimation IR images. File containing the thermal imaging (and paired photographs) of all images used in data collection for the thermocouple protocol. Images are sorted by species and then by individual flower, flower file names are formatted as [flower identifier used for sorting e.g. ‘D’][number]. [file 13007_2021_721_MOESM8_ESM.zip › Thermocouple IR images/Bellis/D12/IR_42403.jpg]

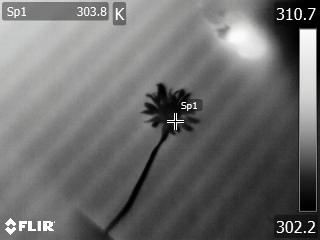

Supplement: Supplementary file 8 — Additional file 8. Thermocouple estimation IR images. File containing the thermal imaging (and paired photographs) of all images used in data collection for the thermocouple protocol. Images are sorted by species and then by individual flower, flower file names are formatted as [flower identifier used for sorting e.g. ‘D’][number]. [file 13007_2021_721_MOESM8_ESM.zip › Thermocouple IR images/Bellis/D12/IR_42405.jpg]

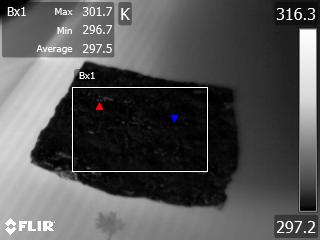

Supplement: Supplementary file 8 — Additional file 8. Thermocouple estimation IR images. File containing the thermal imaging (and paired photographs) of all images used in data collection for the thermocouple protocol. Images are sorted by species and then by individual flower, flower file names are formatted as [flower identifier used for sorting e.g. ‘D’][number]. [file 13007_2021_721_MOESM8_ESM.zip › Thermocouple IR images/Bellis/D12/IR_42407.jpg]

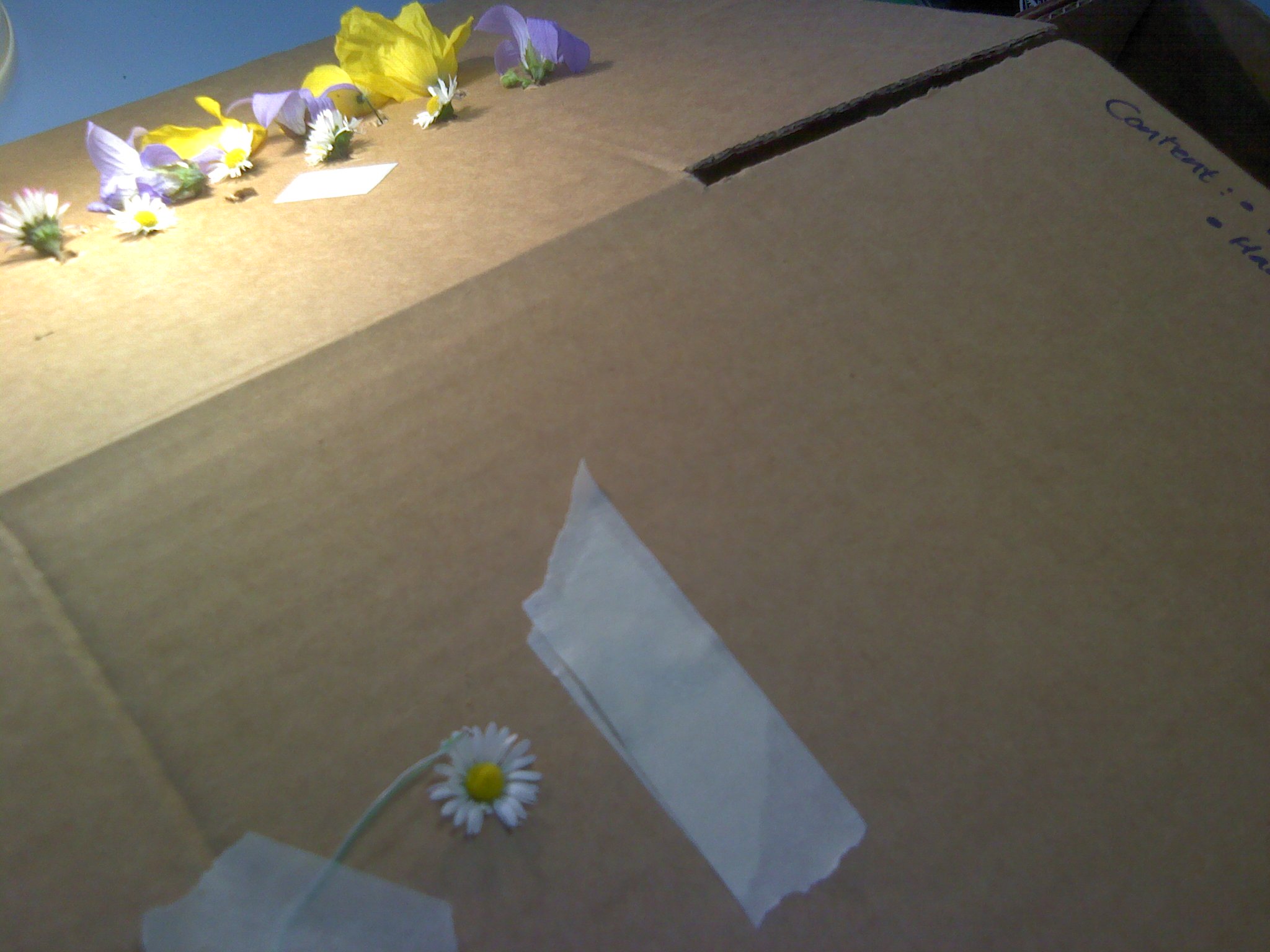

Supplement: Supplementary file 8 — Additional file 8. Thermocouple estimation IR images. File containing the thermal imaging (and paired photographs) of all images used in data collection for the thermocouple protocol. Images are sorted by species and then by individual flower, flower file names are formatted as [flower identifier used for sorting e.g. ‘D’][number]. [file 13007_2021_721_MOESM8_ESM.zip › Thermocouple IR images/Bellis/D13/DC_58462.jpg]

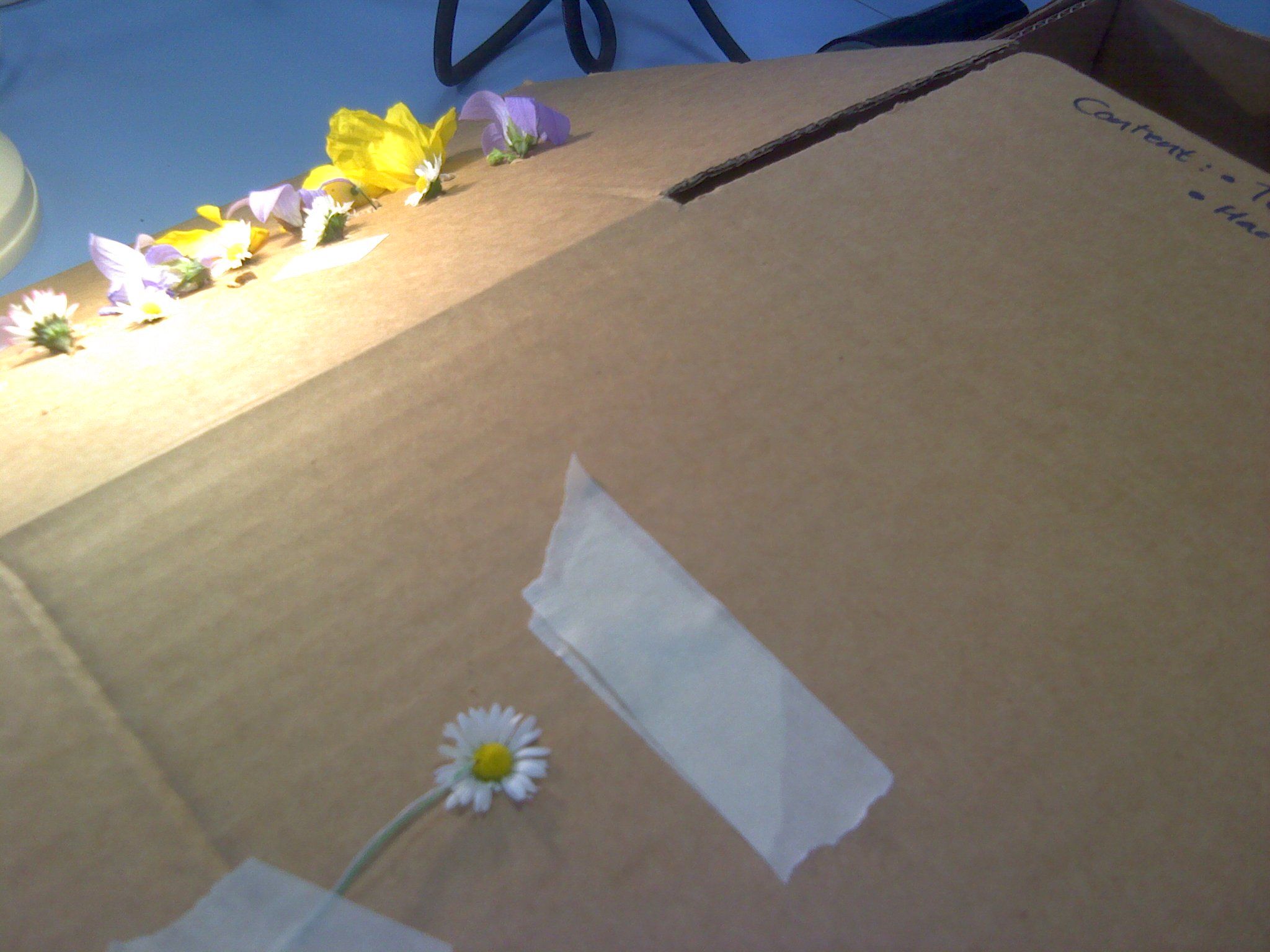

Supplement: Supplementary file 8 — Additional file 8. Thermocouple estimation IR images. File containing the thermal imaging (and paired photographs) of all images used in data collection for the thermocouple protocol. Images are sorted by species and then by individual flower, flower file names are formatted as [flower identifier used for sorting e.g. ‘D’][number]. [file 13007_2021_721_MOESM8_ESM.zip › Thermocouple IR images/Bellis/D13/DC_58468.jpg]

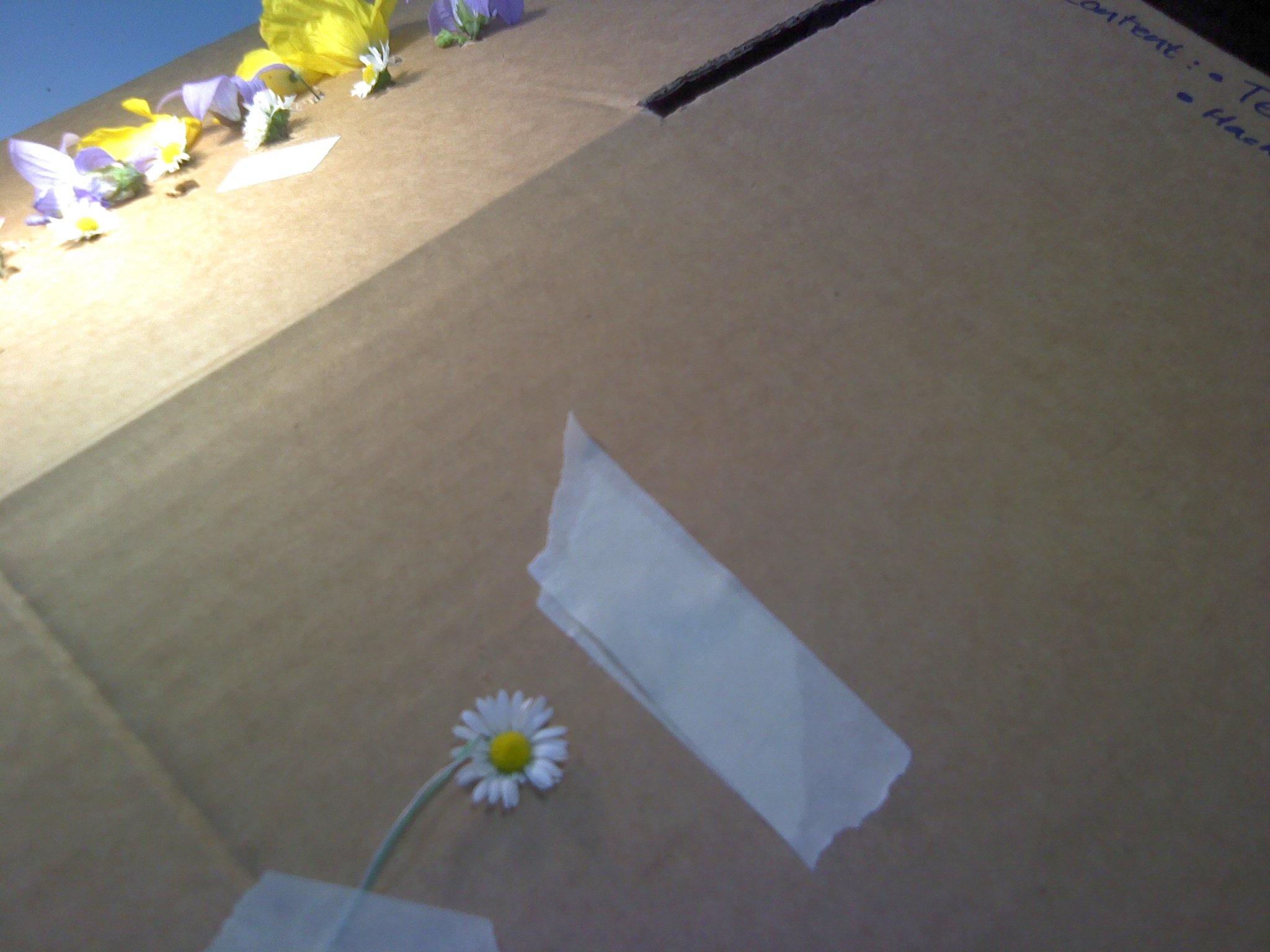

Supplement: Supplementary file 8 — Additional file 8. Thermocouple estimation IR images. File containing the thermal imaging (and paired photographs) of all images used in data collection for the thermocouple protocol. Images are sorted by species and then by individual flower, flower file names are formatted as [flower identifier used for sorting e.g. ‘D’][number]. [file 13007_2021_721_MOESM8_ESM.zip › Thermocouple IR images/Bellis/D13/DC_58472.jpg]

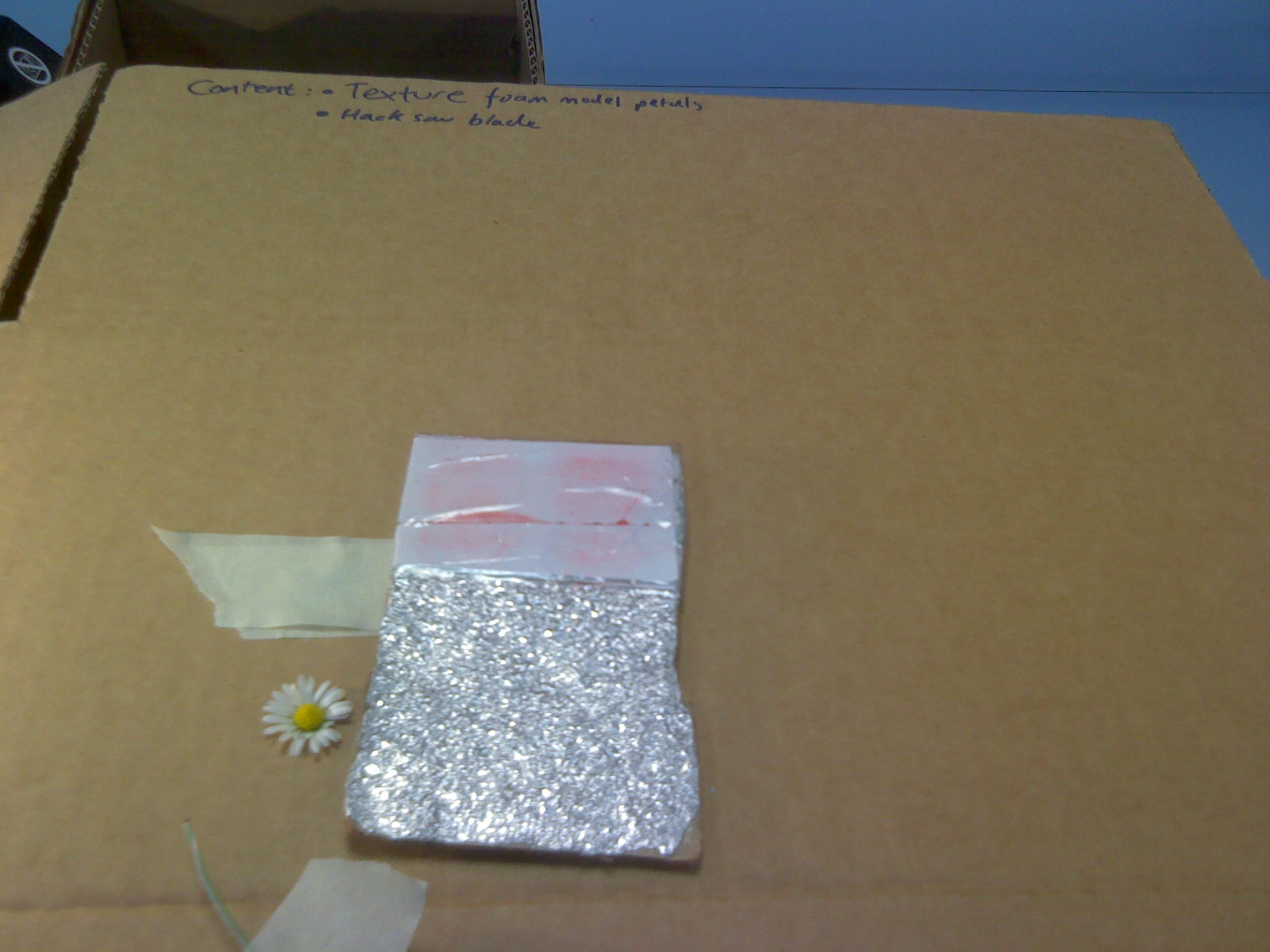

Supplement: Supplementary file 8 — Additional file 8. Thermocouple estimation IR images. File containing the thermal imaging (and paired photographs) of all images used in data collection for the thermocouple protocol. Images are sorted by species and then by individual flower, flower file names are formatted as [flower identifier used for sorting e.g. ‘D’][number]. [file 13007_2021_721_MOESM8_ESM.zip › Thermocouple IR images/Bellis/D13/DC_58474.jpg]

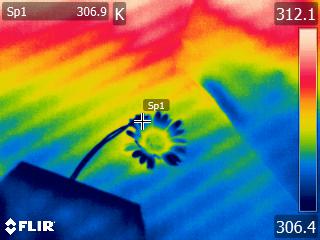

Supplement: Supplementary file 8 — Additional file 8. Thermocouple estimation IR images. File containing the thermal imaging (and paired photographs) of all images used in data collection for the thermocouple protocol. Images are sorted by species and then by individual flower, flower file names are formatted as [flower identifier used for sorting e.g. ‘D’][number]. [file 13007_2021_721_MOESM8_ESM.zip › Thermocouple IR images/Bellis/D13/IR_58461.jpg]

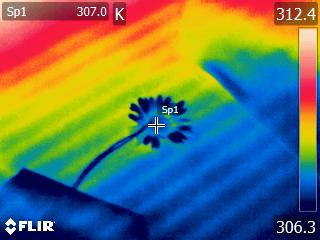

Supplement: Supplementary file 8 — Additional file 8. Thermocouple estimation IR images. File containing the thermal imaging (and paired photographs) of all images used in data collection for the thermocouple protocol. Images are sorted by species and then by individual flower, flower file names are formatted as [flower identifier used for sorting e.g. ‘D’][number]. [file 13007_2021_721_MOESM8_ESM.zip › Thermocouple IR images/Bellis/D13/IR_58467.jpg]

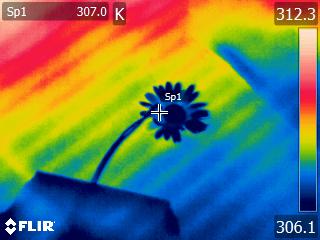

Supplement: Supplementary file 8 — Additional file 8. Thermocouple estimation IR images. File containing the thermal imaging (and paired photographs) of all images used in data collection for the thermocouple protocol. Images are sorted by species and then by individual flower, flower file names are formatted as [flower identifier used for sorting e.g. ‘D’][number]. [file 13007_2021_721_MOESM8_ESM.zip › Thermocouple IR images/Bellis/D13/IR_58471.jpg]

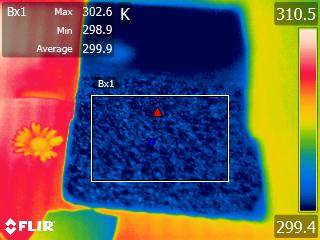

Supplement: Supplementary file 8 — Additional file 8. Thermocouple estimation IR images. File containing the thermal imaging (and paired photographs) of all images used in data collection for the thermocouple protocol. Images are sorted by species and then by individual flower, flower file names are formatted as [flower identifier used for sorting e.g. ‘D’][number]. [file 13007_2021_721_MOESM8_ESM.zip › Thermocouple IR images/Bellis/D13/IR_58473.jpg]

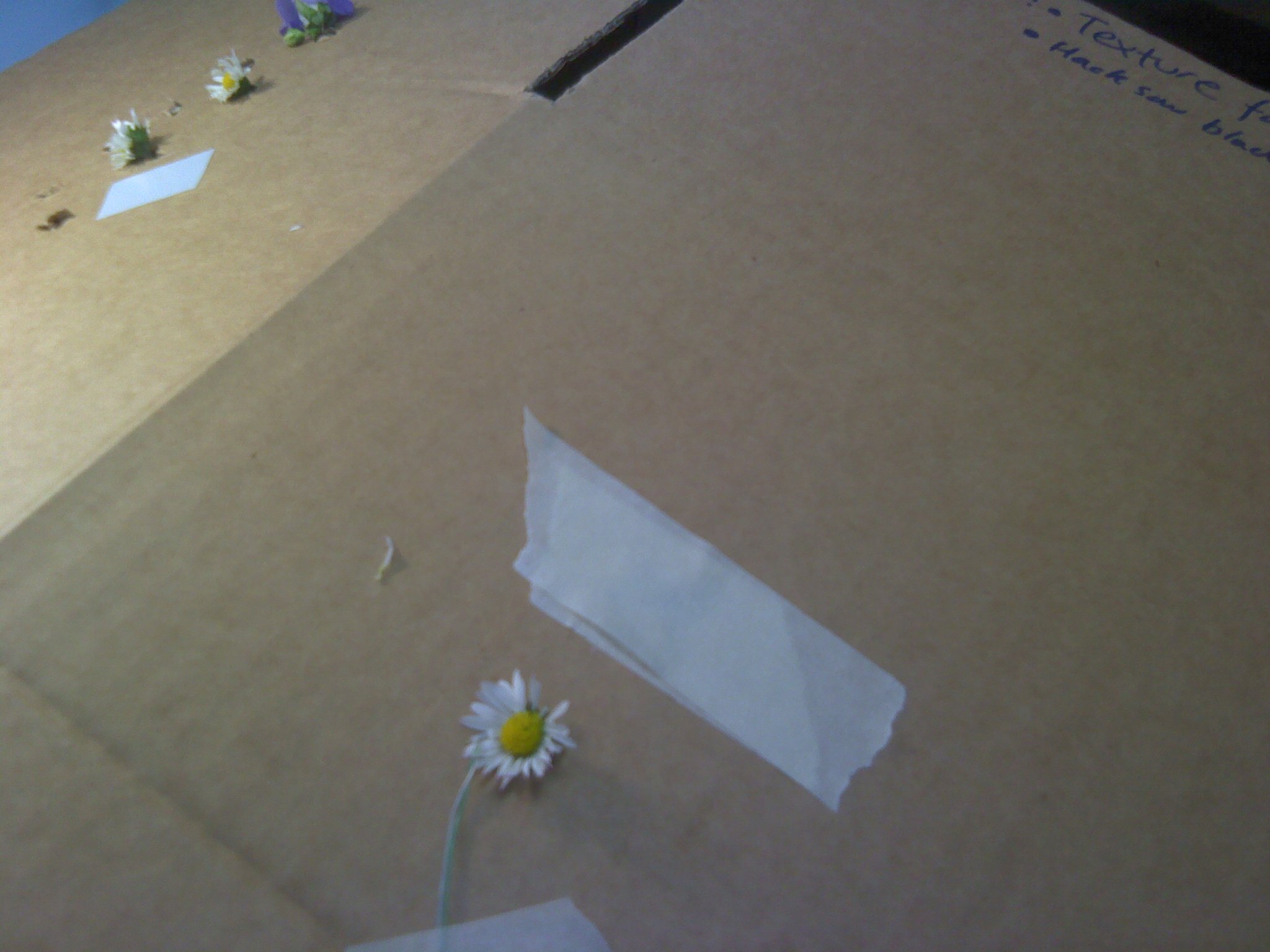

Supplement: Supplementary file 8 — Additional file 8. Thermocouple estimation IR images. File containing the thermal imaging (and paired photographs) of all images used in data collection for the thermocouple protocol. Images are sorted by species and then by individual flower, flower file names are formatted as [flower identifier used for sorting e.g. ‘D’][number]. [file 13007_2021_721_MOESM8_ESM.zip › Thermocouple IR images/Bellis/D14/DC_58562.jpg]

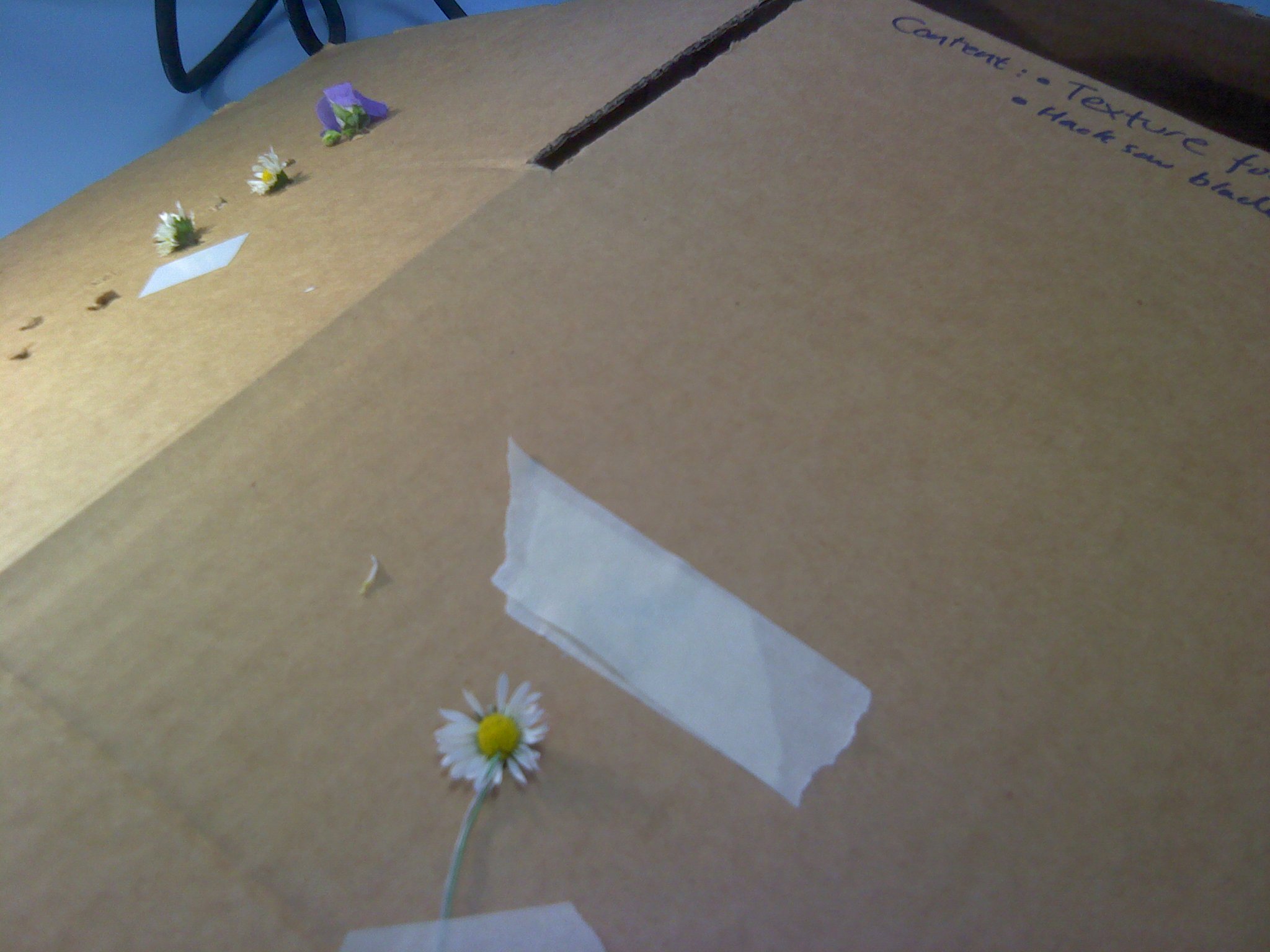

Supplement: Supplementary file 8 — Additional file 8. Thermocouple estimation IR images. File containing the thermal imaging (and paired photographs) of all images used in data collection for the thermocouple protocol. Images are sorted by species and then by individual flower, flower file names are formatted as [flower identifier used for sorting e.g. ‘D’][number]. [file 13007_2021_721_MOESM8_ESM.zip › Thermocouple IR images/Bellis/D14/DC_58564.jpg]

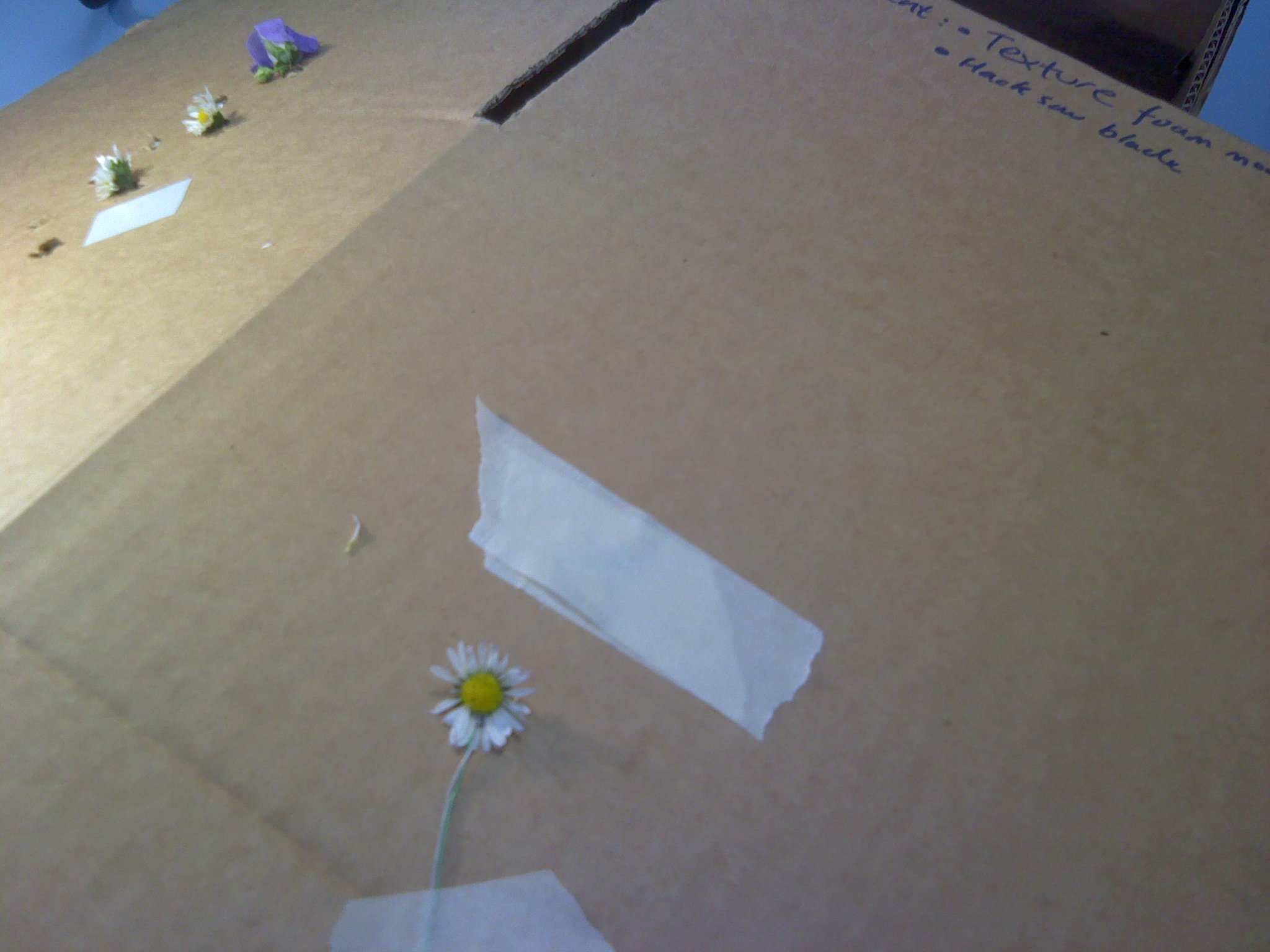

Supplement: Supplementary file 8 — Additional file 8. Thermocouple estimation IR images. File containing the thermal imaging (and paired photographs) of all images used in data collection for the thermocouple protocol. Images are sorted by species and then by individual flower, flower file names are formatted as [flower identifier used for sorting e.g. ‘D’][number]. [file 13007_2021_721_MOESM8_ESM.zip › Thermocouple IR images/Bellis/D14/DC_58568.jpg]

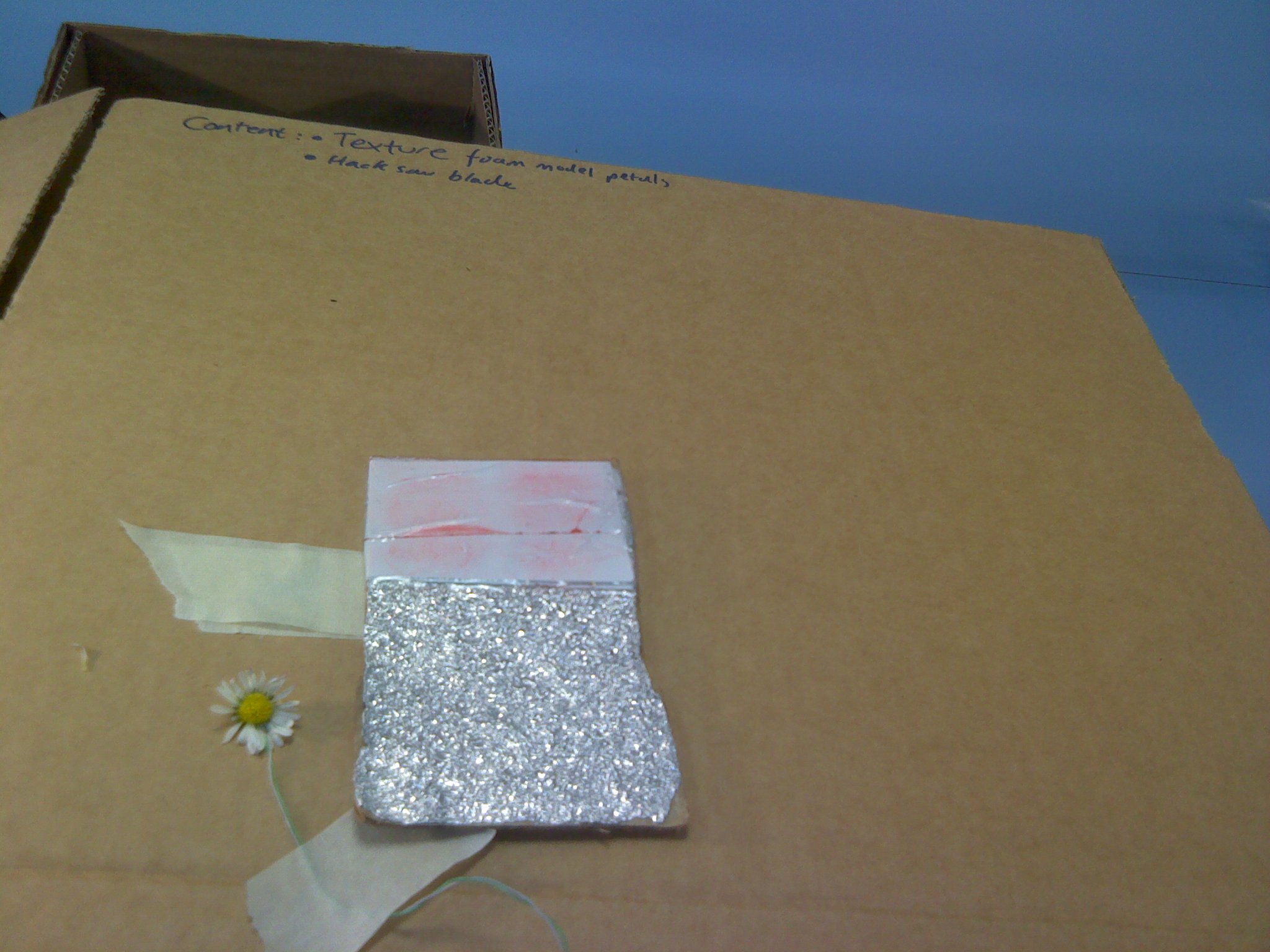

Supplement: Supplementary file 8 — Additional file 8. Thermocouple estimation IR images. File containing the thermal imaging (and paired photographs) of all images used in data collection for the thermocouple protocol. Images are sorted by species and then by individual flower, flower file names are formatted as [flower identifier used for sorting e.g. ‘D’][number]. [file 13007_2021_721_MOESM8_ESM.zip › Thermocouple IR images/Bellis/D14/DC_58570.jpg]

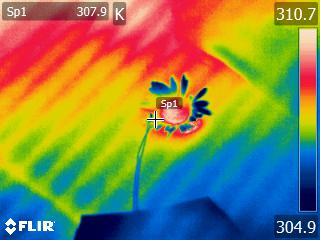

Supplement: Supplementary file 8 — Additional file 8. Thermocouple estimation IR images. File containing the thermal imaging (and paired photographs) of all images used in data collection for the thermocouple protocol. Images are sorted by species and then by individual flower, flower file names are formatted as [flower identifier used for sorting e.g. ‘D’][number]. [file 13007_2021_721_MOESM8_ESM.zip › Thermocouple IR images/Bellis/D14/IR_58561.jpg]

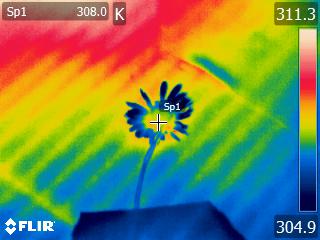

Supplement: Supplementary file 8 — Additional file 8. Thermocouple estimation IR images. File containing the thermal imaging (and paired photographs) of all images used in data collection for the thermocouple protocol. Images are sorted by species and then by individual flower, flower file names are formatted as [flower identifier used for sorting e.g. ‘D’][number]. [file 13007_2021_721_MOESM8_ESM.zip › Thermocouple IR images/Bellis/D14/IR_58563.jpg]

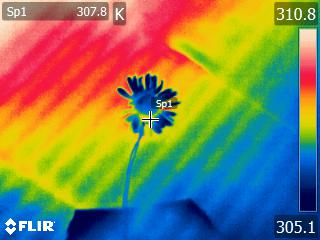

Supplement: Supplementary file 8 — Additional file 8. Thermocouple estimation IR images. File containing the thermal imaging (and paired photographs) of all images used in data collection for the thermocouple protocol. Images are sorted by species and then by individual flower, flower file names are formatted as [flower identifier used for sorting e.g. ‘D’][number]. [file 13007_2021_721_MOESM8_ESM.zip › Thermocouple IR images/Bellis/D14/IR_58567.jpg]

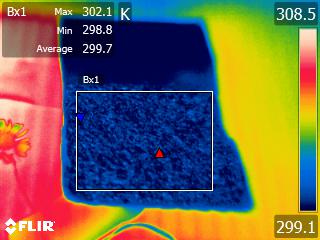

Supplement: Supplementary file 8 — Additional file 8. Thermocouple estimation IR images. File containing the thermal imaging (and paired photographs) of all images used in data collection for the thermocouple protocol. Images are sorted by species and then by individual flower, flower file names are formatted as [flower identifier used for sorting e.g. ‘D’][number]. [file 13007_2021_721_MOESM8_ESM.zip › Thermocouple IR images/Bellis/D14/IR_58569.jpg]

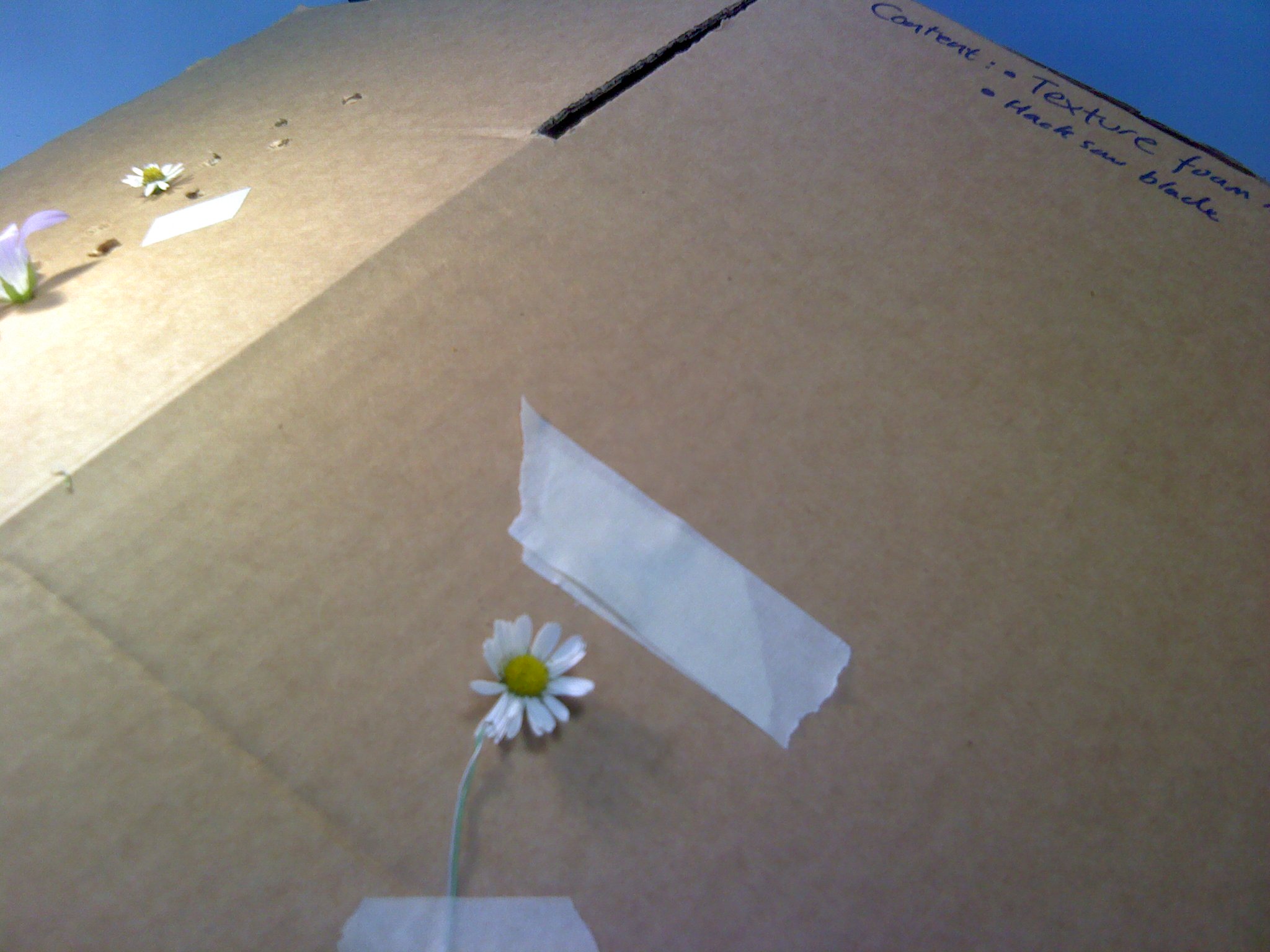

Supplement: Supplementary file 8 — Additional file 8. Thermocouple estimation IR images. File containing the thermal imaging (and paired photographs) of all images used in data collection for the thermocouple protocol. Images are sorted by species and then by individual flower, flower file names are formatted as [flower identifier used for sorting e.g. ‘D’][number]. [file 13007_2021_721_MOESM8_ESM.zip › Thermocouple IR images/Bellis/D15/DC_58600.jpg]

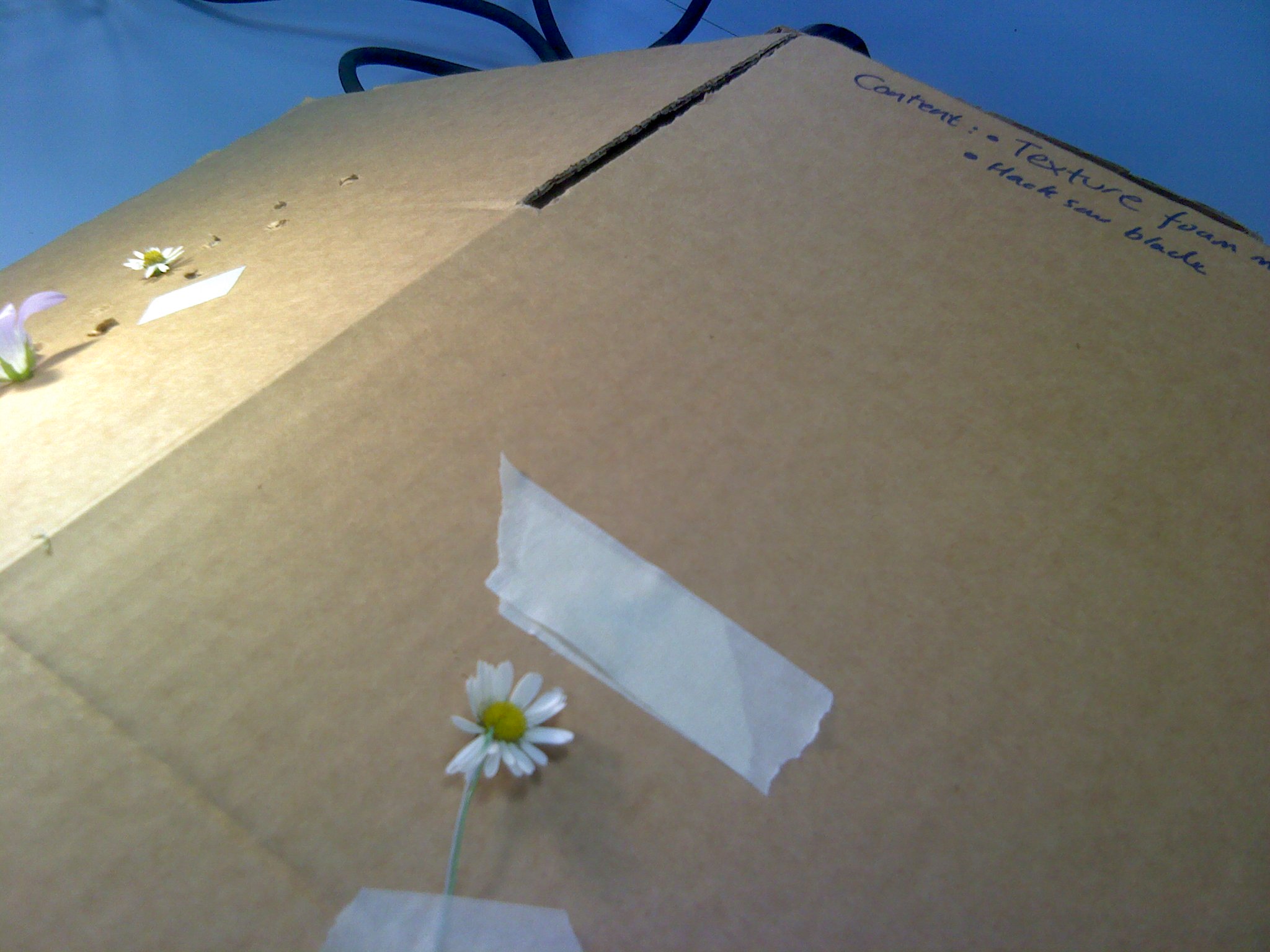

Supplement: Supplementary file 8 — Additional file 8. Thermocouple estimation IR images. File containing the thermal imaging (and paired photographs) of all images used in data collection for the thermocouple protocol. Images are sorted by species and then by individual flower, flower file names are formatted as [flower identifier used for sorting e.g. ‘D’][number]. [file 13007_2021_721_MOESM8_ESM.zip › Thermocouple IR images/Bellis/D15/DC_58604.jpg]

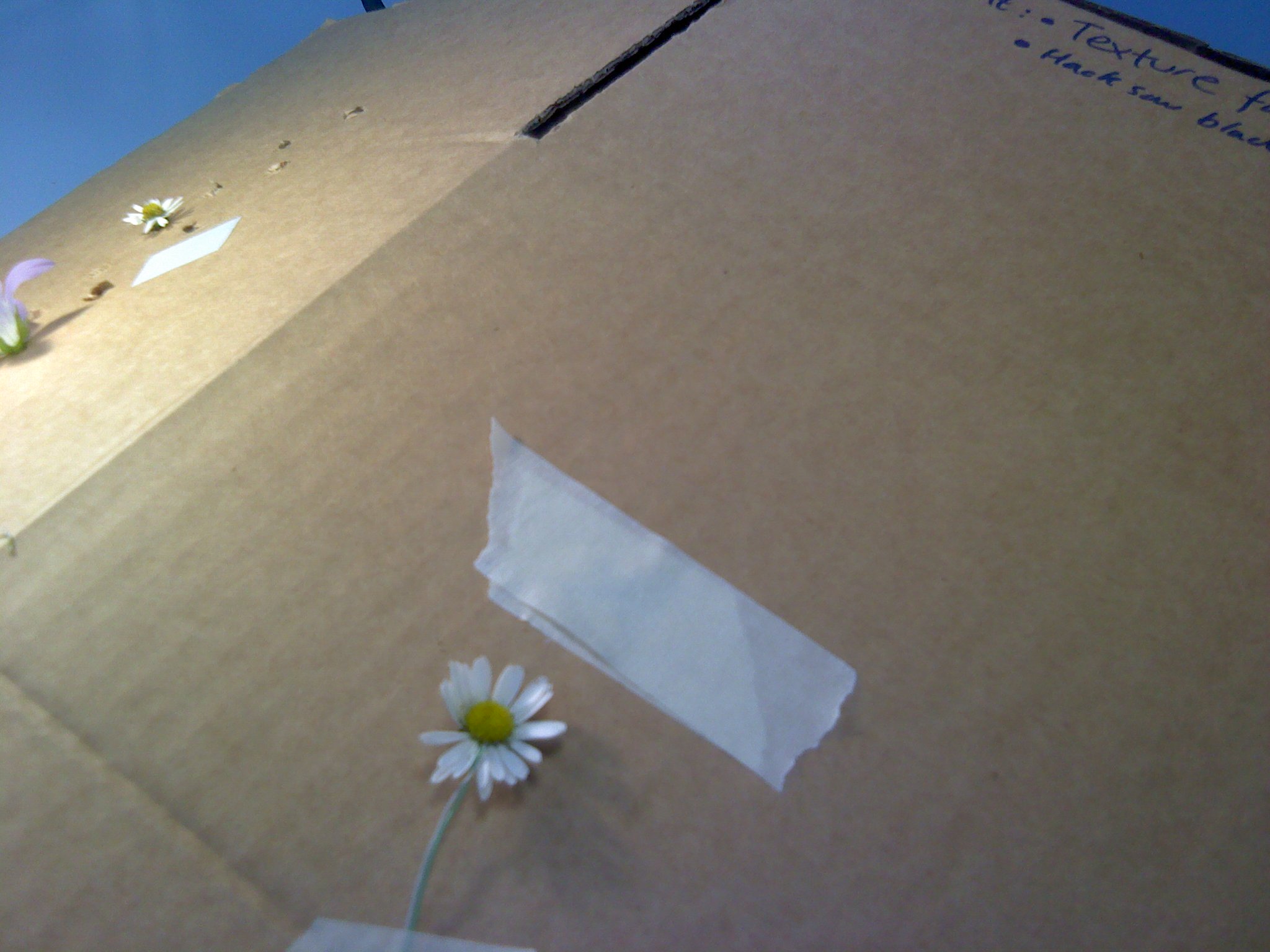

Supplement: Supplementary file 8 — Additional file 8. Thermocouple estimation IR images. File containing the thermal imaging (and paired photographs) of all images used in data collection for the thermocouple protocol. Images are sorted by species and then by individual flower, flower file names are formatted as [flower identifier used for sorting e.g. ‘D’][number]. [file 13007_2021_721_MOESM8_ESM.zip › Thermocouple IR images/Bellis/D15/DC_58608.jpg]

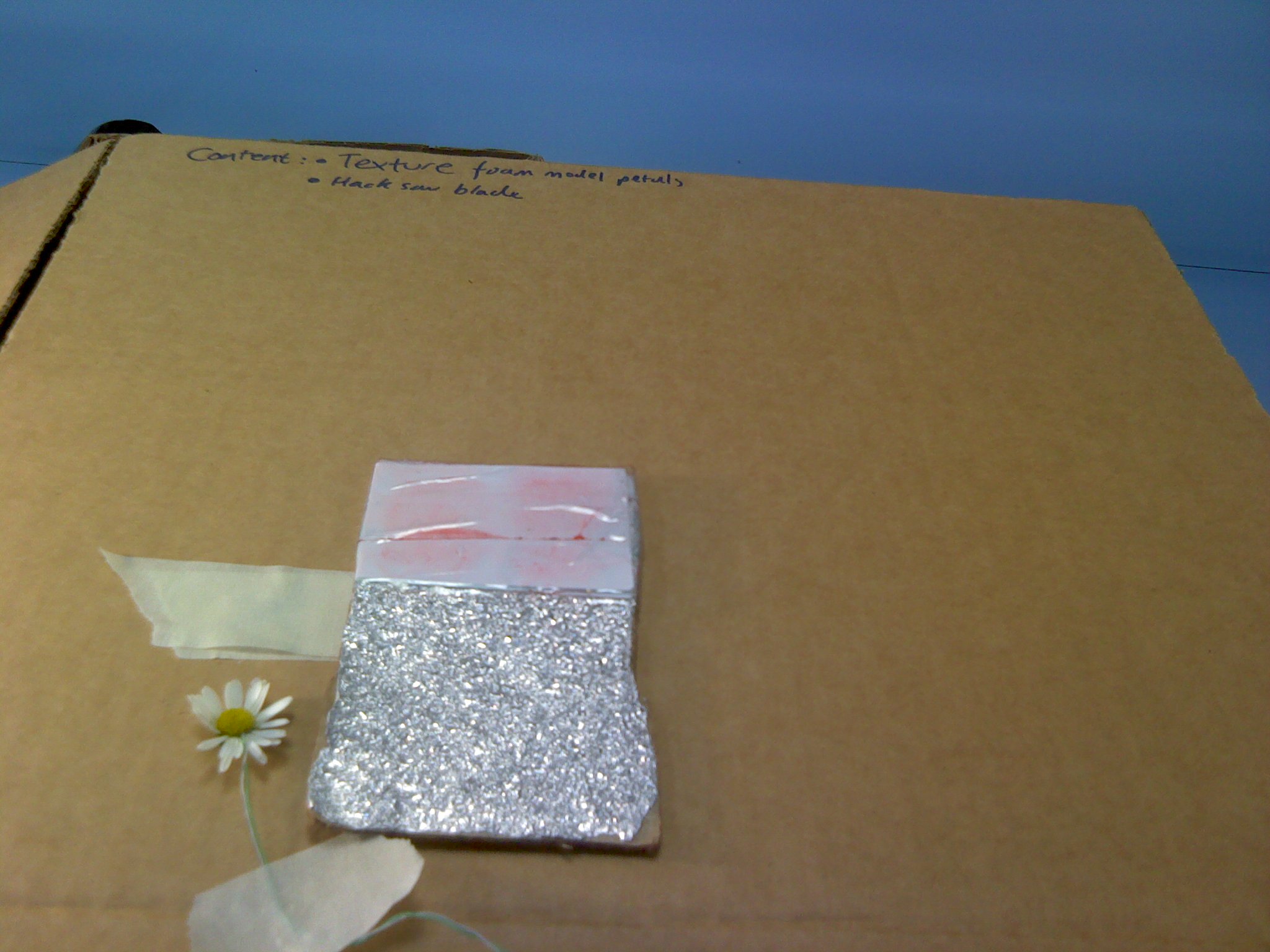

Supplement: Supplementary file 8 — Additional file 8. Thermocouple estimation IR images. File containing the thermal imaging (and paired photographs) of all images used in data collection for the thermocouple protocol. Images are sorted by species and then by individual flower, flower file names are formatted as [flower identifier used for sorting e.g. ‘D’][number]. [file 13007_2021_721_MOESM8_ESM.zip › Thermocouple IR images/Bellis/D15/DC_58610.jpg]

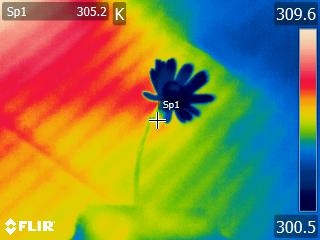

Supplement: Supplementary file 8 — Additional file 8. Thermocouple estimation IR images. File containing the thermal imaging (and paired photographs) of all images used in data collection for the thermocouple protocol. Images are sorted by species and then by individual flower, flower file names are formatted as [flower identifier used for sorting e.g. ‘D’][number]. [file 13007_2021_721_MOESM8_ESM.zip › Thermocouple IR images/Bellis/D15/IR_58599.jpg]

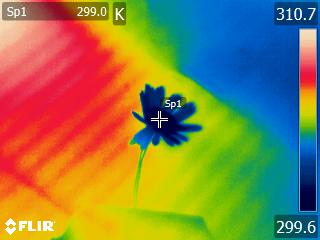

Supplement: Supplementary file 8 — Additional file 8. Thermocouple estimation IR images. File containing the thermal imaging (and paired photographs) of all images used in data collection for the thermocouple protocol. Images are sorted by species and then by individual flower, flower file names are formatted as [flower identifier used for sorting e.g. ‘D’][number]. [file 13007_2021_721_MOESM8_ESM.zip › Thermocouple IR images/Bellis/D15/IR_58603.jpg]

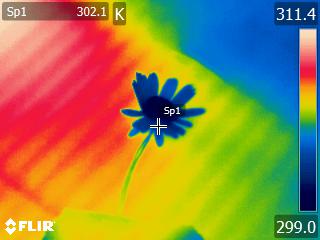

Supplement: Supplementary file 8 — Additional file 8. Thermocouple estimation IR images. File containing the thermal imaging (and paired photographs) of all images used in data collection for the thermocouple protocol. Images are sorted by species and then by individual flower, flower file names are formatted as [flower identifier used for sorting e.g. ‘D’][number]. [file 13007_2021_721_MOESM8_ESM.zip › Thermocouple IR images/Bellis/D15/IR_58607.jpg]

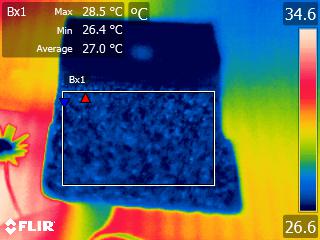

Supplement: Supplementary file 8 — Additional file 8. Thermocouple estimation IR images. File containing the thermal imaging (and paired photographs) of all images used in data collection for the thermocouple protocol. Images are sorted by species and then by individual flower, flower file names are formatted as [flower identifier used for sorting e.g. ‘D’][number]. [file 13007_2021_721_MOESM8_ESM.zip › Thermocouple IR images/Bellis/D15/IR_58609.jpg]

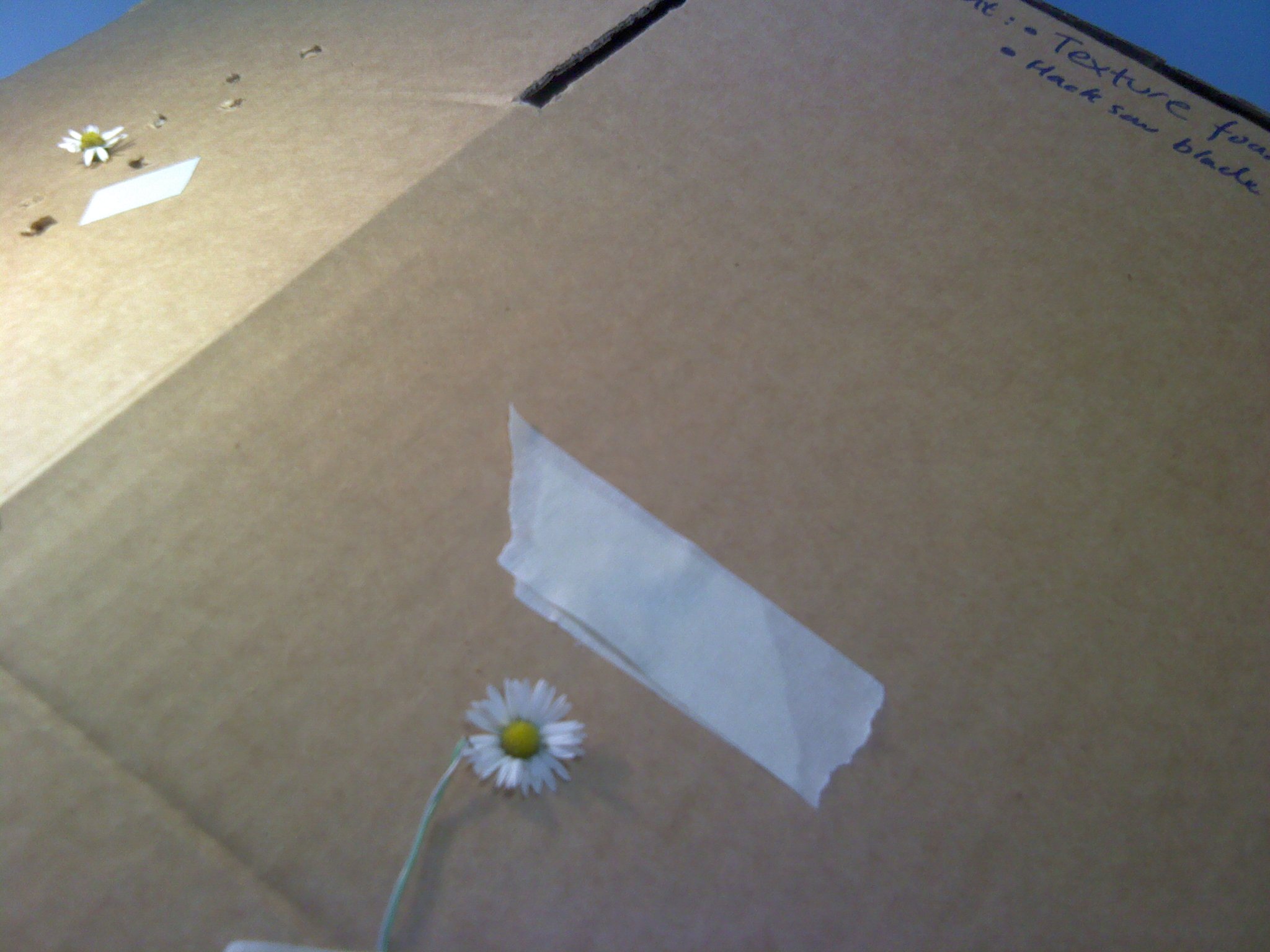

Supplement: Supplementary file 8 — Additional file 8. Thermocouple estimation IR images. File containing the thermal imaging (and paired photographs) of all images used in data collection for the thermocouple protocol. Images are sorted by species and then by individual flower, flower file names are formatted as [flower identifier used for sorting e.g. ‘D’][number]. [file 13007_2021_721_MOESM8_ESM.zip › Thermocouple IR images/Bellis/D16/DC_58648.jpg]

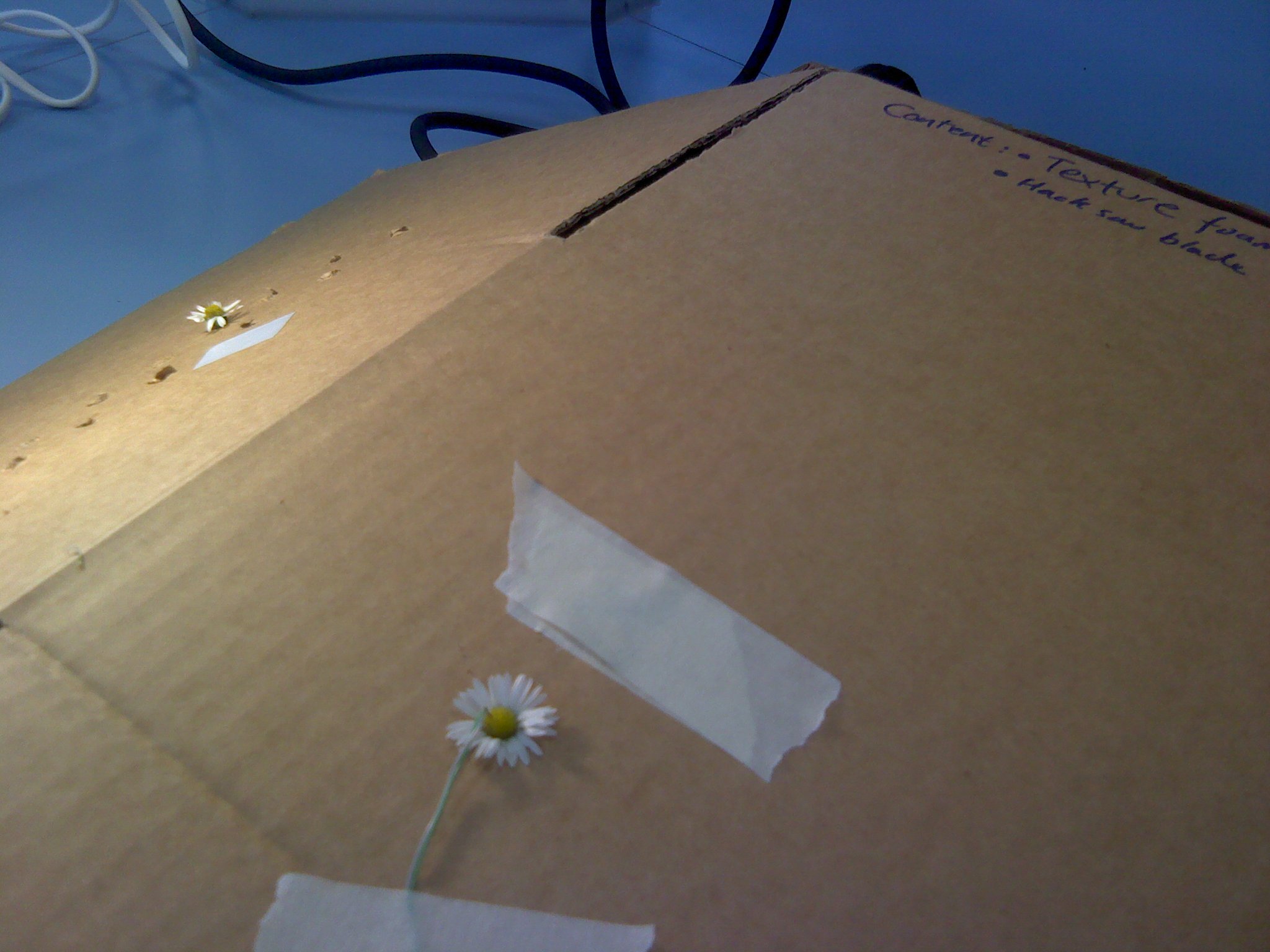

Supplement: Supplementary file 8 — Additional file 8. Thermocouple estimation IR images. File containing the thermal imaging (and paired photographs) of all images used in data collection for the thermocouple protocol. Images are sorted by species and then by individual flower, flower file names are formatted as [flower identifier used for sorting e.g. ‘D’][number]. [file 13007_2021_721_MOESM8_ESM.zip › Thermocouple IR images/Bellis/D16/DC_58650.jpg]

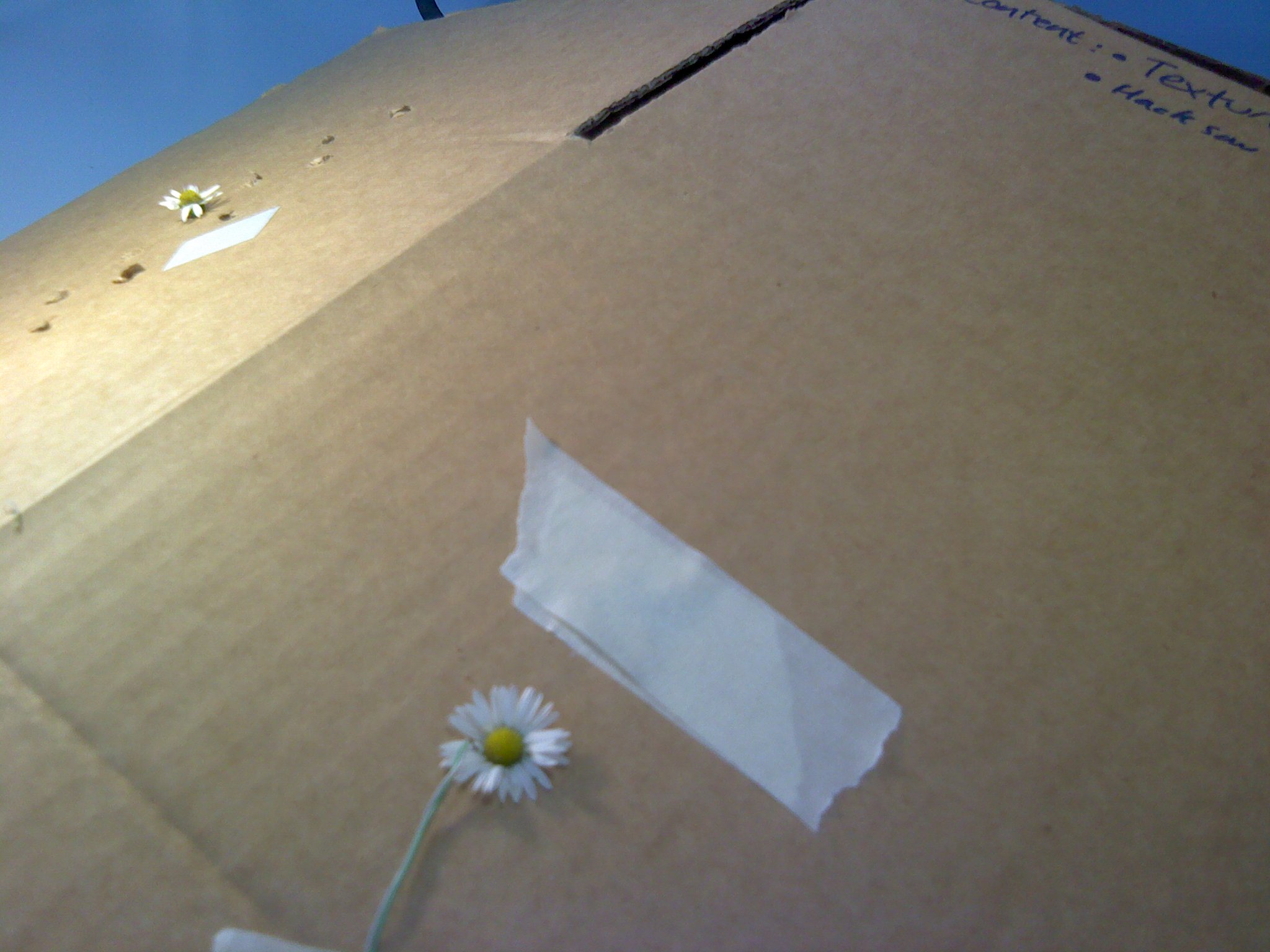

Supplement: Supplementary file 8 — Additional file 8. Thermocouple estimation IR images. File containing the thermal imaging (and paired photographs) of all images used in data collection for the thermocouple protocol. Images are sorted by species and then by individual flower, flower file names are formatted as [flower identifier used for sorting e.g. ‘D’][number]. [file 13007_2021_721_MOESM8_ESM.zip › Thermocouple IR images/Bellis/D16/DC_58652.jpg]

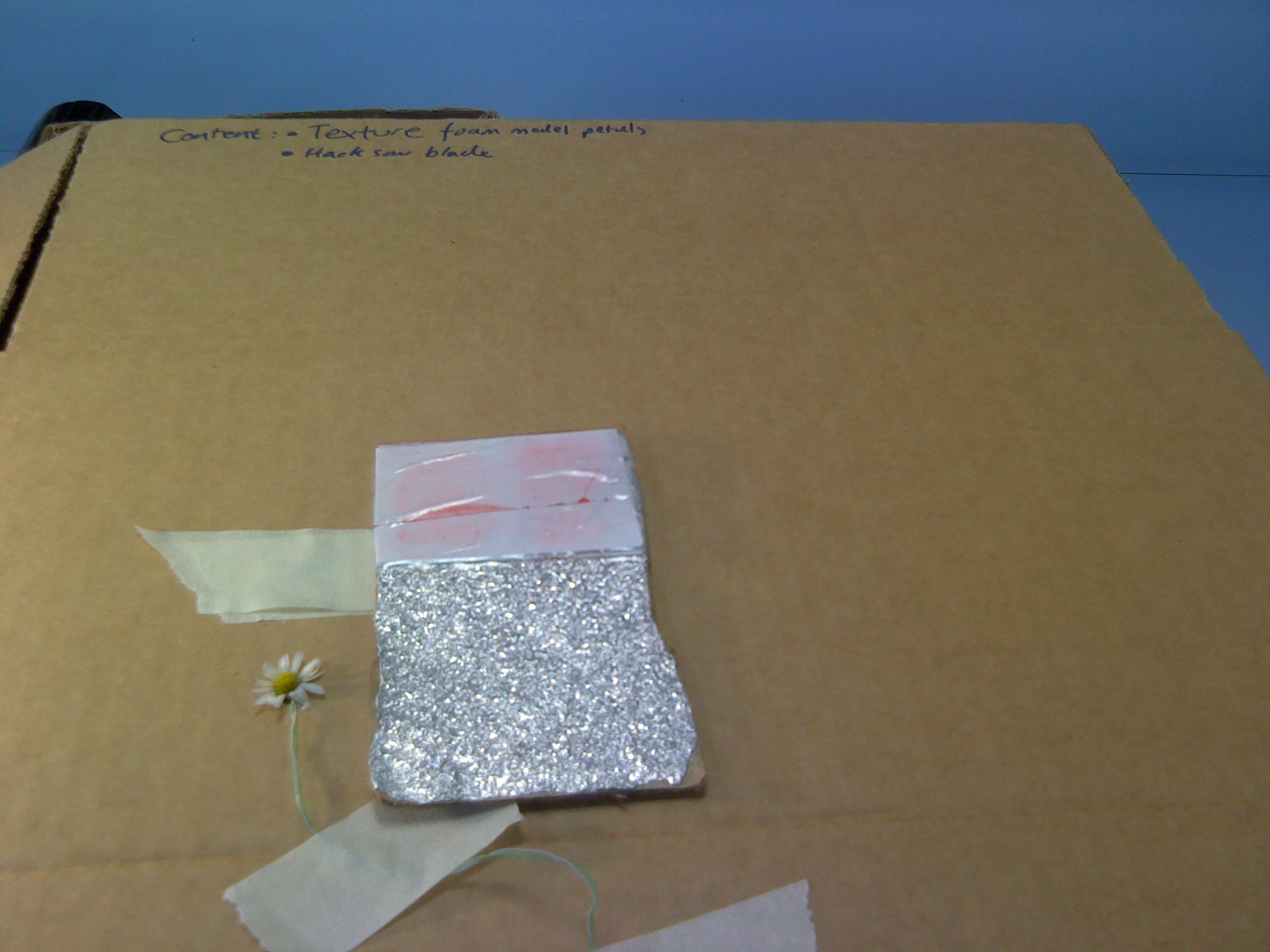

Supplement: Supplementary file 8 — Additional file 8. Thermocouple estimation IR images. File containing the thermal imaging (and paired photographs) of all images used in data collection for the thermocouple protocol. Images are sorted by species and then by individual flower, flower file names are formatted as [flower identifier used for sorting e.g. ‘D’][number]. [file 13007_2021_721_MOESM8_ESM.zip › Thermocouple IR images/Bellis/D16/DC_58654.jpg]

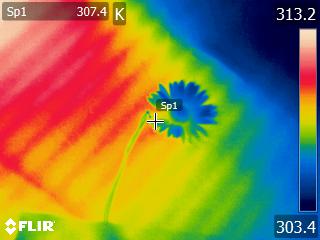

Supplement: Supplementary file 8 — Additional file 8. Thermocouple estimation IR images. File containing the thermal imaging (and paired photographs) of all images used in data collection for the thermocouple protocol. Images are sorted by species and then by individual flower, flower file names are formatted as [flower identifier used for sorting e.g. ‘D’][number]. [file 13007_2021_721_MOESM8_ESM.zip › Thermocouple IR images/Bellis/D16/IR_58647.jpg]

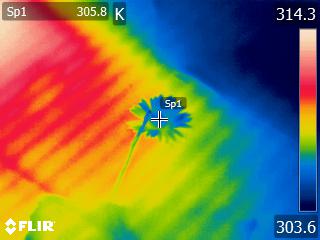

Supplement: Supplementary file 8 — Additional file 8. Thermocouple estimation IR images. File containing the thermal imaging (and paired photographs) of all images used in data collection for the thermocouple protocol. Images are sorted by species and then by individual flower, flower file names are formatted as [flower identifier used for sorting e.g. ‘D’][number]. [file 13007_2021_721_MOESM8_ESM.zip › Thermocouple IR images/Bellis/D16/IR_58649.jpg]

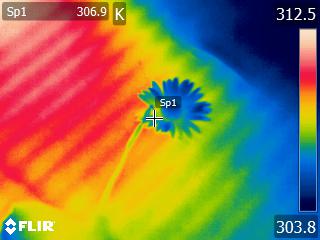

Supplement: Supplementary file 8 — Additional file 8. Thermocouple estimation IR images. File containing the thermal imaging (and paired photographs) of all images used in data collection for the thermocouple protocol. Images are sorted by species and then by individual flower, flower file names are formatted as [flower identifier used for sorting e.g. ‘D’][number]. [file 13007_2021_721_MOESM8_ESM.zip › Thermocouple IR images/Bellis/D16/IR_58651.jpg]

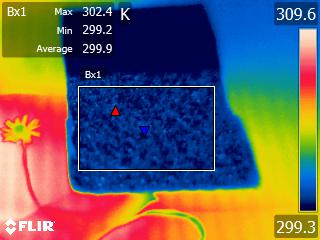

Supplement: Supplementary file 8 — Additional file 8. Thermocouple estimation IR images. File containing the thermal imaging (and paired photographs) of all images used in data collection for the thermocouple protocol. Images are sorted by species and then by individual flower, flower file names are formatted as [flower identifier used for sorting e.g. ‘D’][number]. [file 13007_2021_721_MOESM8_ESM.zip › Thermocouple IR images/Bellis/D16/IR_58653.jpg]

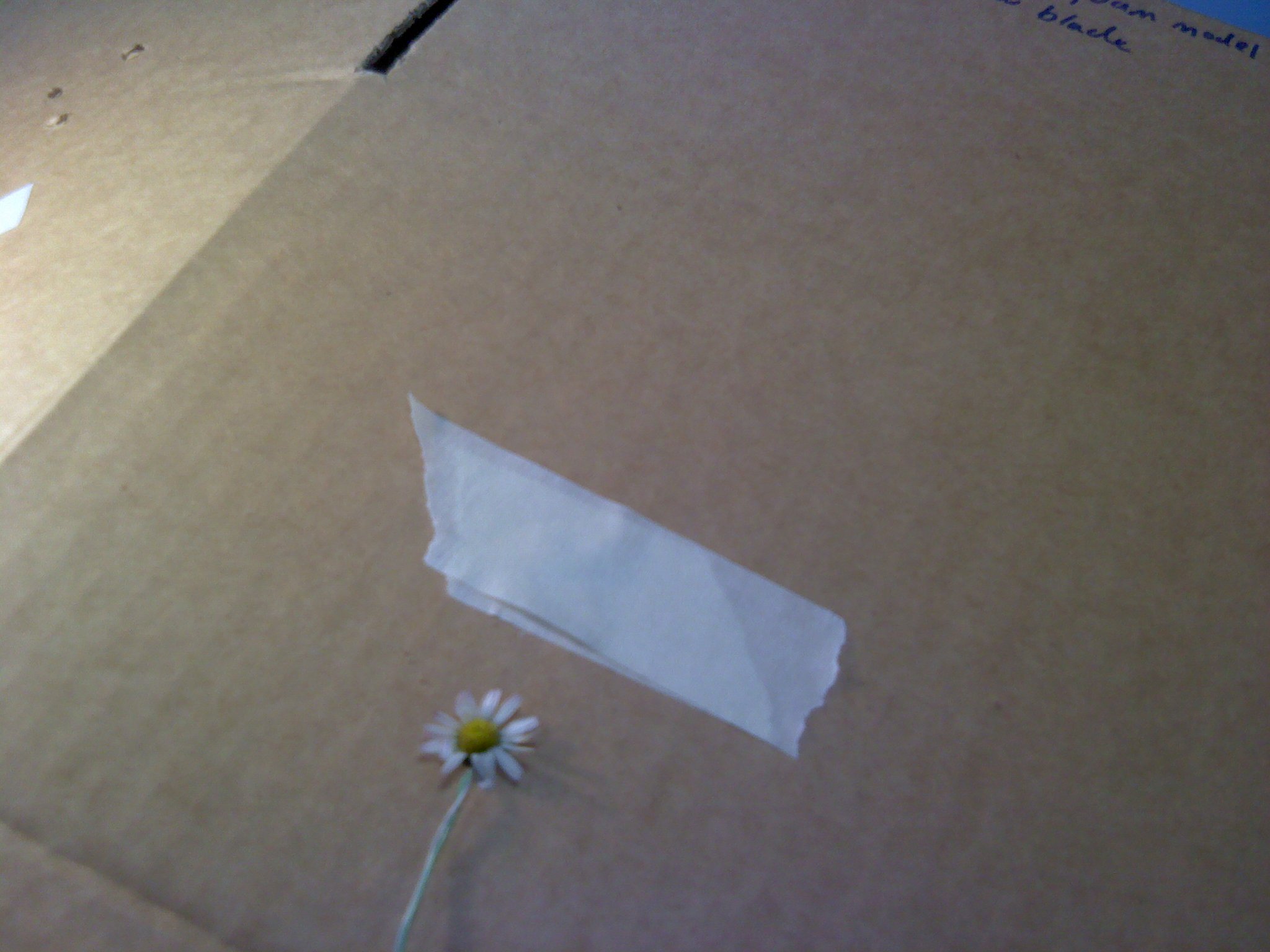

Supplement: Supplementary file 8 — Additional file 8. Thermocouple estimation IR images. File containing the thermal imaging (and paired photographs) of all images used in data collection for the thermocouple protocol. Images are sorted by species and then by individual flower, flower file names are formatted as [flower identifier used for sorting e.g. ‘D’][number]. [file 13007_2021_721_MOESM8_ESM.zip › Thermocouple IR images/Bellis/D17/DC_58656.jpg]

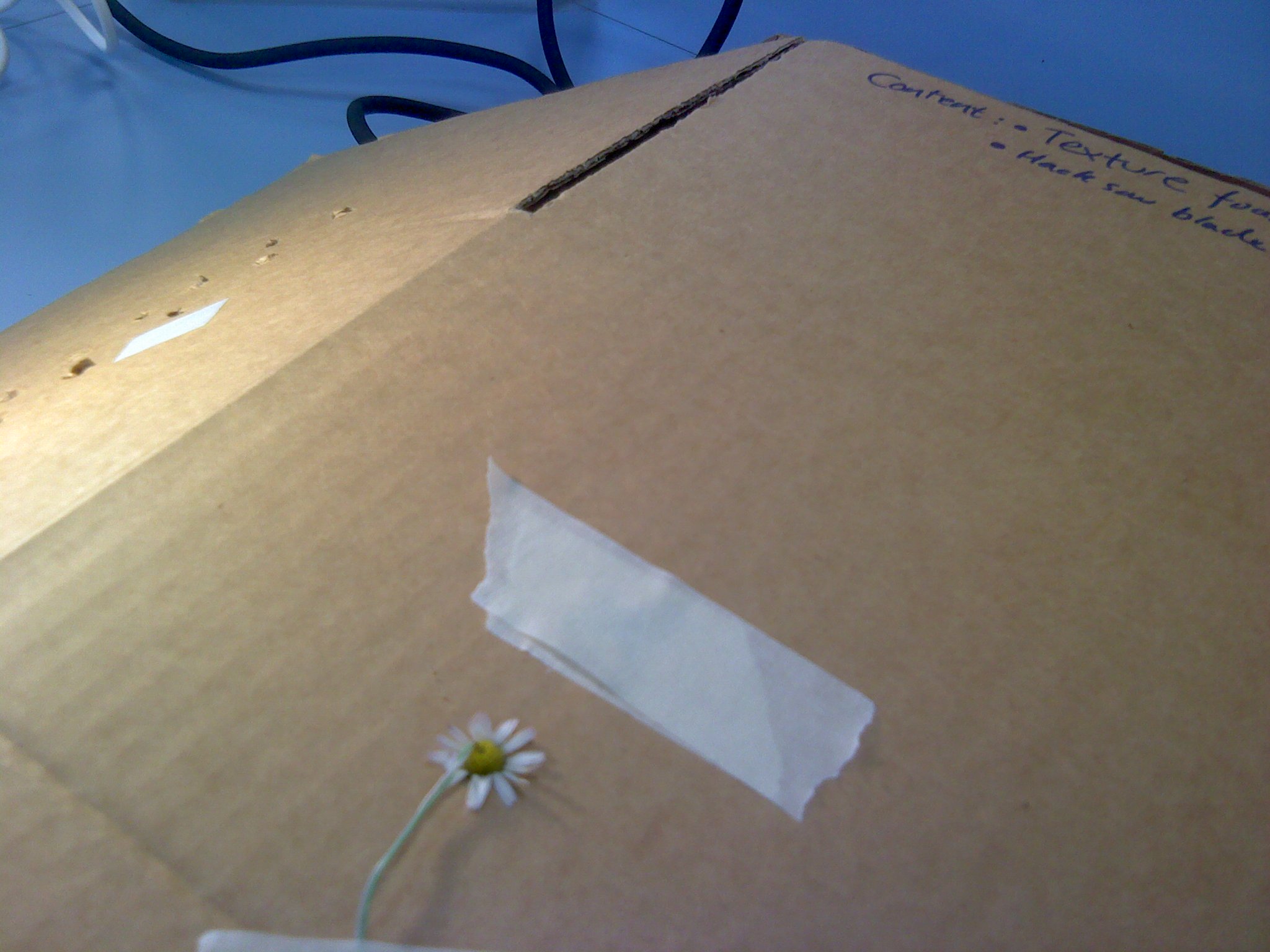

Supplement: Supplementary file 8 — Additional file 8. Thermocouple estimation IR images. File containing the thermal imaging (and paired photographs) of all images used in data collection for the thermocouple protocol. Images are sorted by species and then by individual flower, flower file names are formatted as [flower identifier used for sorting e.g. ‘D’][number]. [file 13007_2021_721_MOESM8_ESM.zip › Thermocouple IR images/Bellis/D17/DC_58658.jpg]

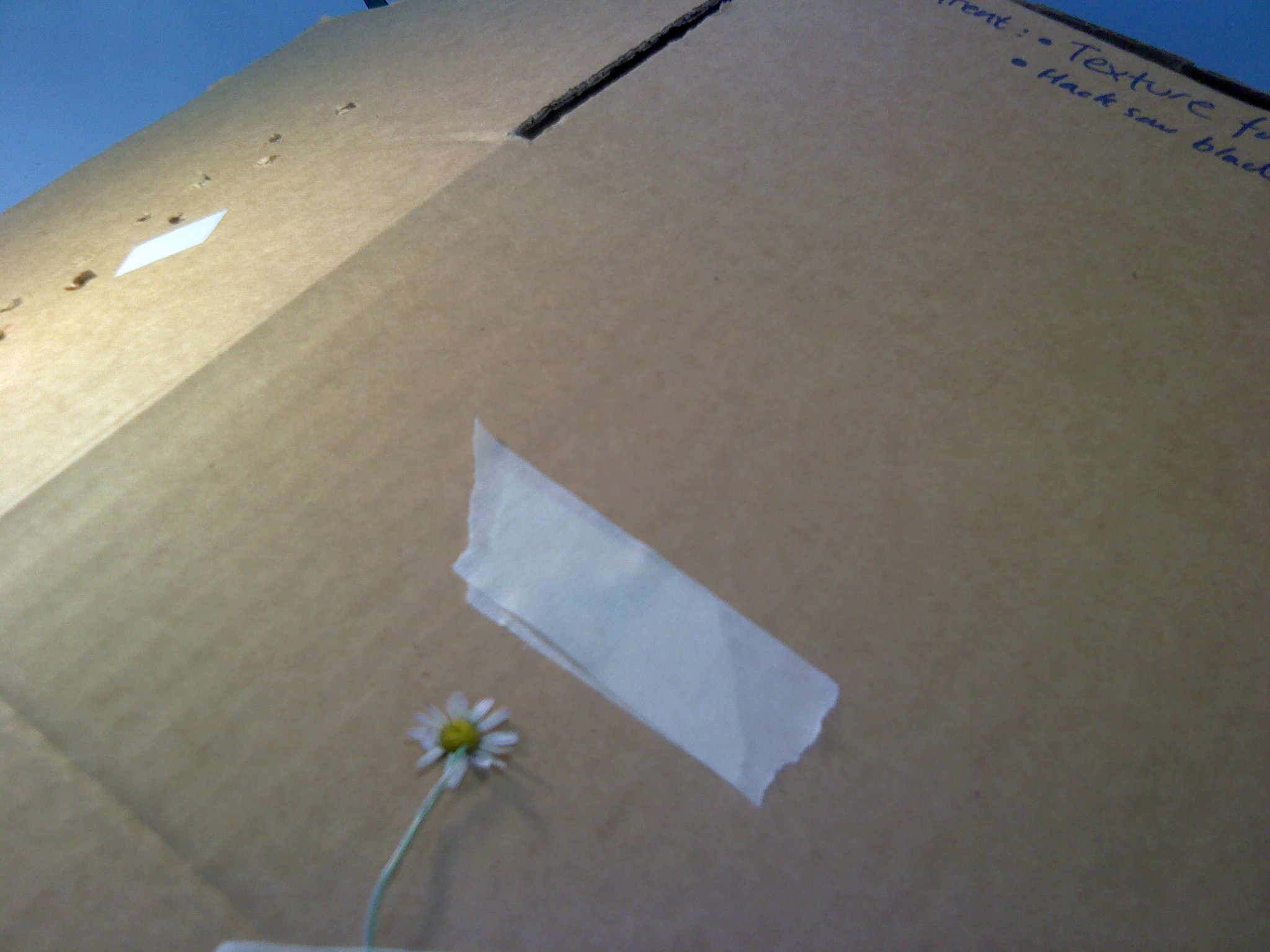

Supplement: Supplementary file 8 — Additional file 8. Thermocouple estimation IR images. File containing the thermal imaging (and paired photographs) of all images used in data collection for the thermocouple protocol. Images are sorted by species and then by individual flower, flower file names are formatted as [flower identifier used for sorting e.g. ‘D’][number]. [file 13007_2021_721_MOESM8_ESM.zip › Thermocouple IR images/Bellis/D17/DC_58662.jpg]

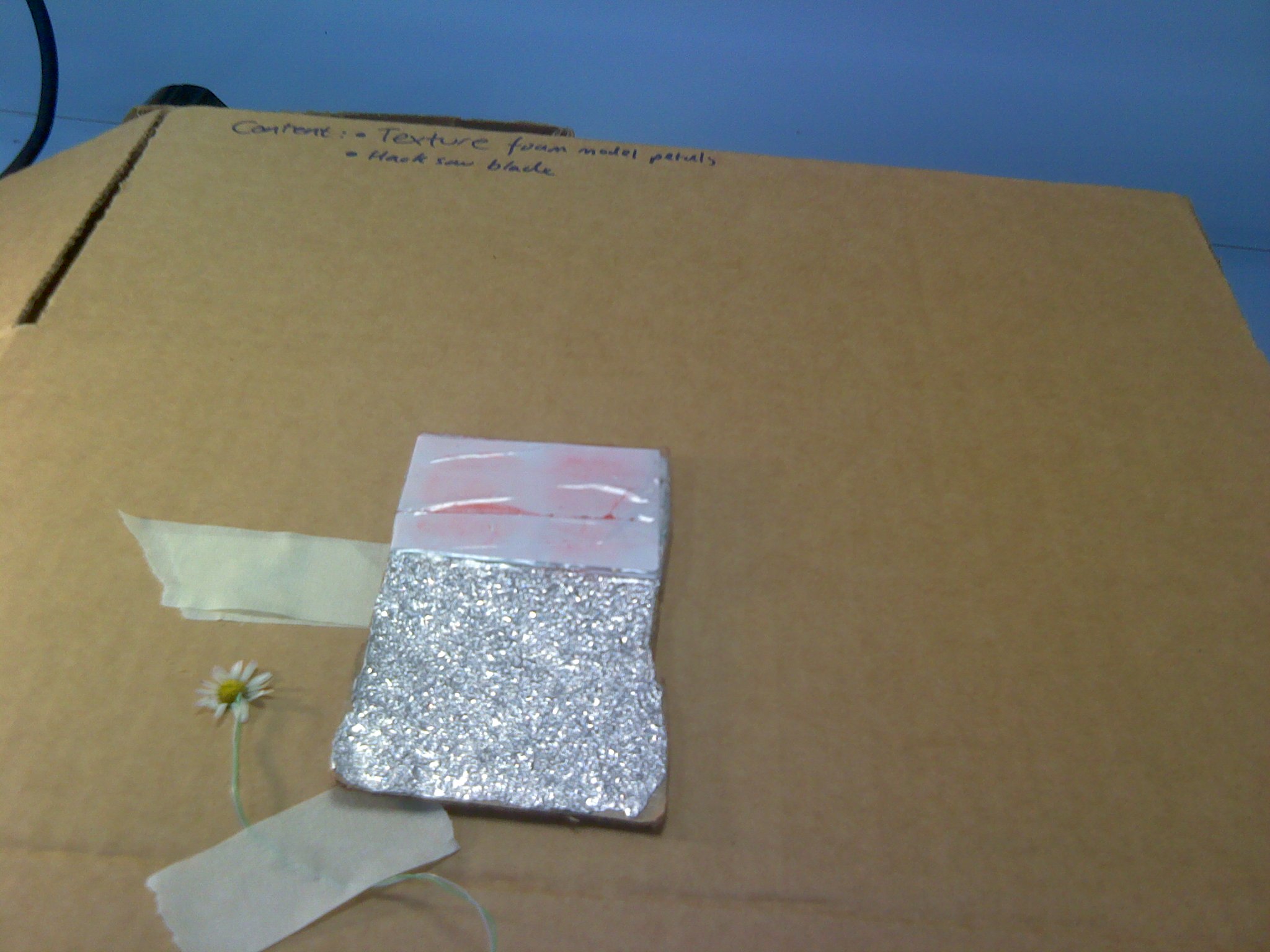

Supplement: Supplementary file 8 — Additional file 8. Thermocouple estimation IR images. File containing the thermal imaging (and paired photographs) of all images used in data collection for the thermocouple protocol. Images are sorted by species and then by individual flower, flower file names are formatted as [flower identifier used for sorting e.g. ‘D’][number]. [file 13007_2021_721_MOESM8_ESM.zip › Thermocouple IR images/Bellis/D17/DC_58664.jpg]

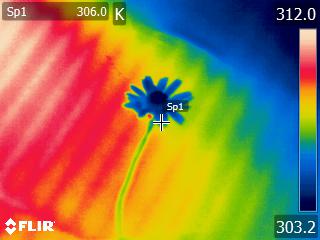

Supplement: Supplementary file 8 — Additional file 8. Thermocouple estimation IR images. File containing the thermal imaging (and paired photographs) of all images used in data collection for the thermocouple protocol. Images are sorted by species and then by individual flower, flower file names are formatted as [flower identifier used for sorting e.g. ‘D’][number]. [file 13007_2021_721_MOESM8_ESM.zip › Thermocouple IR images/Bellis/D17/IR_58655.jpg]

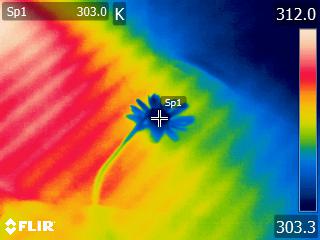

Supplement: Supplementary file 8 — Additional file 8. Thermocouple estimation IR images. File containing the thermal imaging (and paired photographs) of all images used in data collection for the thermocouple protocol. Images are sorted by species and then by individual flower, flower file names are formatted as [flower identifier used for sorting e.g. ‘D’][number]. [file 13007_2021_721_MOESM8_ESM.zip › Thermocouple IR images/Bellis/D17/IR_58657.jpg]

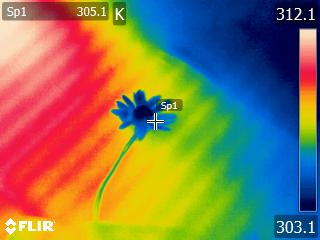

Supplement: Supplementary file 8 — Additional file 8. Thermocouple estimation IR images. File containing the thermal imaging (and paired photographs) of all images used in data collection for the thermocouple protocol. Images are sorted by species and then by individual flower, flower file names are formatted as [flower identifier used for sorting e.g. ‘D’][number]. [file 13007_2021_721_MOESM8_ESM.zip › Thermocouple IR images/Bellis/D17/IR_58661.jpg]

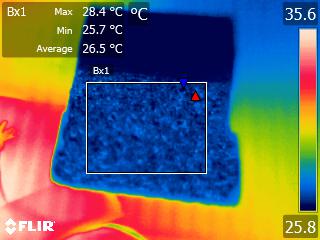

Supplement: Supplementary file 8 — Additional file 8. Thermocouple estimation IR images. File containing the thermal imaging (and paired photographs) of all images used in data collection for the thermocouple protocol. Images are sorted by species and then by individual flower, flower file names are formatted as [flower identifier used for sorting e.g. ‘D’][number]. [file 13007_2021_721_MOESM8_ESM.zip › Thermocouple IR images/Bellis/D17/IR_58663.jpg]

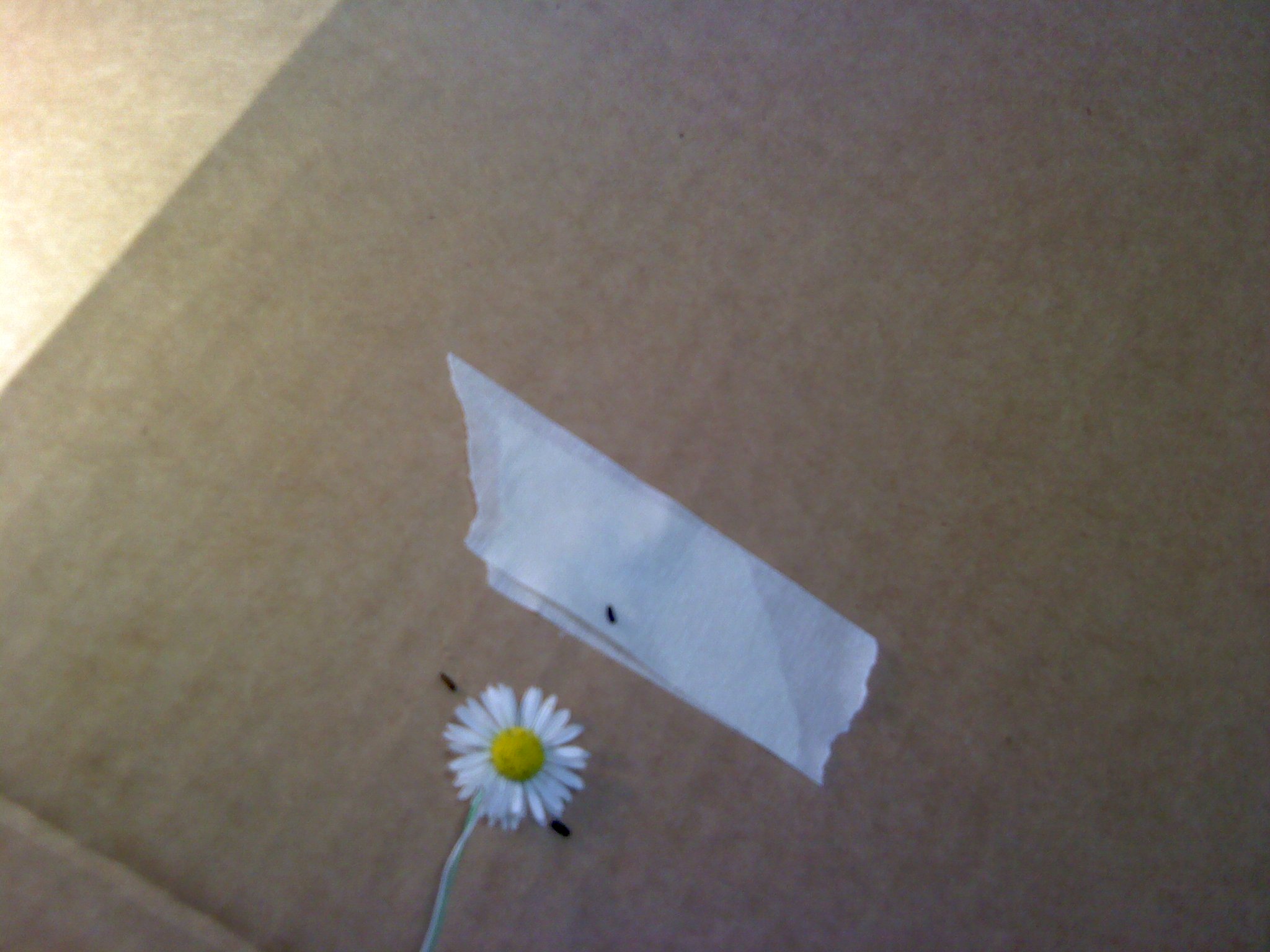

Supplement: Supplementary file 8 — Additional file 8. Thermocouple estimation IR images. File containing the thermal imaging (and paired photographs) of all images used in data collection for the thermocouple protocol. Images are sorted by species and then by individual flower, flower file names are formatted as [flower identifier used for sorting e.g. ‘D’][number]. [file 13007_2021_721_MOESM8_ESM.zip › Thermocouple IR images/Bellis/D18/DC_74888.jpg]

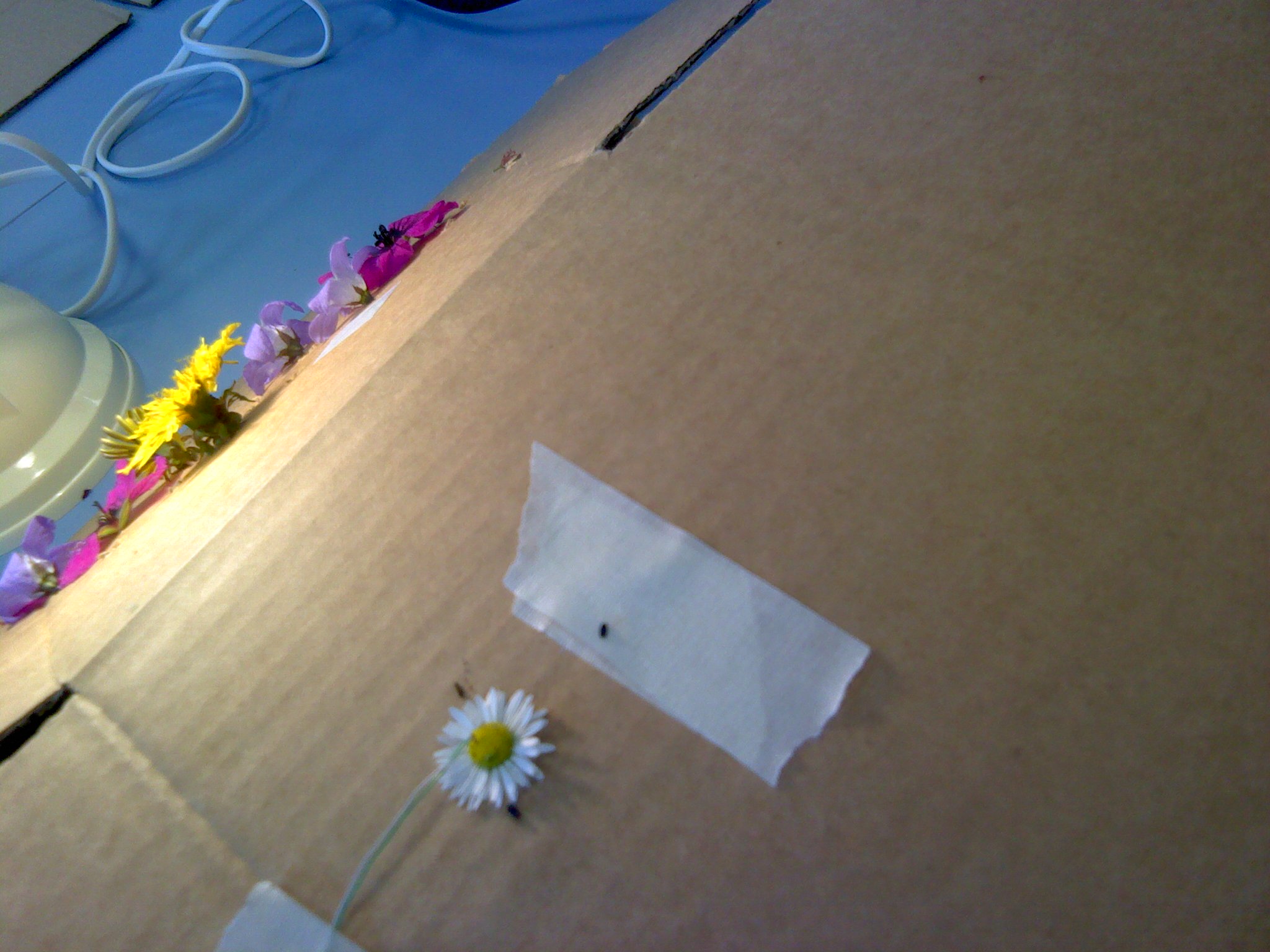

Supplement: Supplementary file 8 — Additional file 8. Thermocouple estimation IR images. File containing the thermal imaging (and paired photographs) of all images used in data collection for the thermocouple protocol. Images are sorted by species and then by individual flower, flower file names are formatted as [flower identifier used for sorting e.g. ‘D’][number]. [file 13007_2021_721_MOESM8_ESM.zip › Thermocouple IR images/Bellis/D18/DC_74892.jpg]

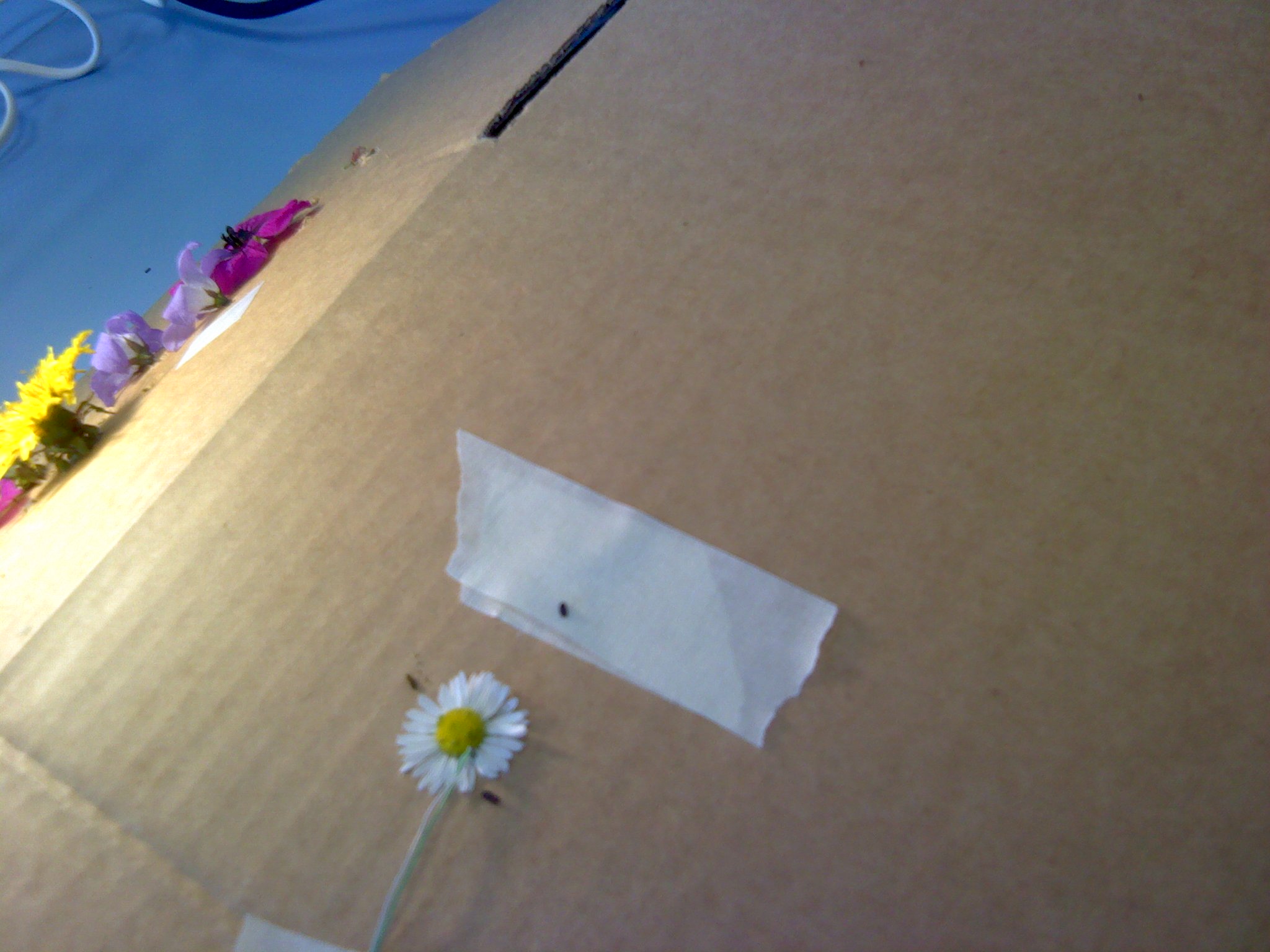

Supplement: Supplementary file 8 — Additional file 8. Thermocouple estimation IR images. File containing the thermal imaging (and paired photographs) of all images used in data collection for the thermocouple protocol. Images are sorted by species and then by individual flower, flower file names are formatted as [flower identifier used for sorting e.g. ‘D’][number]. [file 13007_2021_721_MOESM8_ESM.zip › Thermocouple IR images/Bellis/D18/DC_74896.jpg]

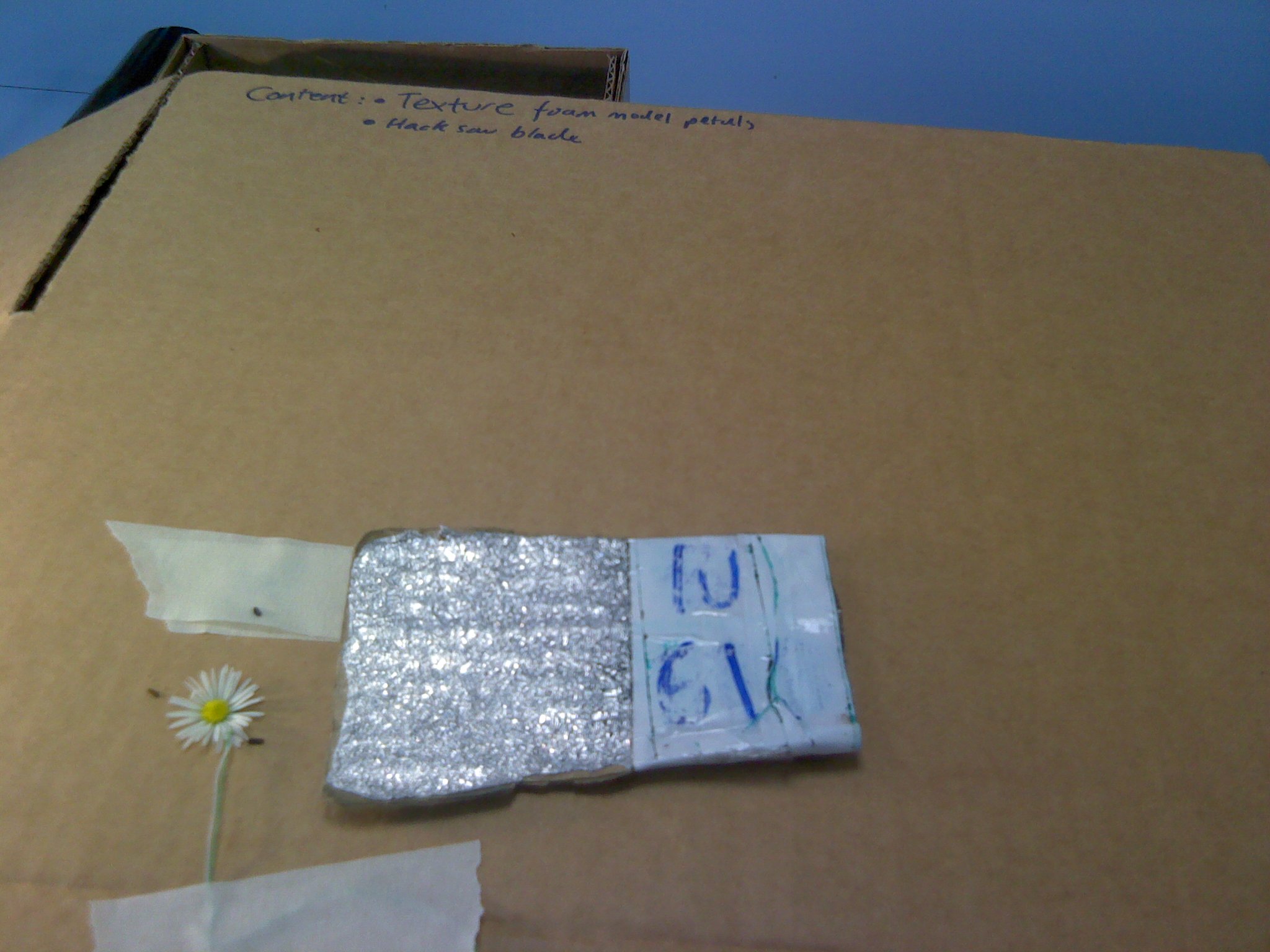

Supplement: Supplementary file 8 — Additional file 8. Thermocouple estimation IR images. File containing the thermal imaging (and paired photographs) of all images used in data collection for the thermocouple protocol. Images are sorted by species and then by individual flower, flower file names are formatted as [flower identifier used for sorting e.g. ‘D’][number]. [file 13007_2021_721_MOESM8_ESM.zip › Thermocouple IR images/Bellis/D18/DC_74898.jpg]

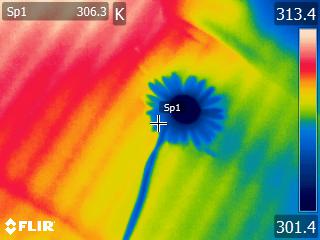

Supplement: Supplementary file 8 — Additional file 8. Thermocouple estimation IR images. File containing the thermal imaging (and paired photographs) of all images used in data collection for the thermocouple protocol. Images are sorted by species and then by individual flower, flower file names are formatted as [flower identifier used for sorting e.g. ‘D’][number]. [file 13007_2021_721_MOESM8_ESM.zip › Thermocouple IR images/Bellis/D18/IR_74887.jpg]

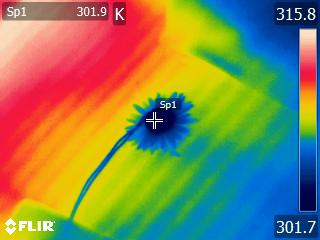

Supplement: Supplementary file 8 — Additional file 8. Thermocouple estimation IR images. File containing the thermal imaging (and paired photographs) of all images used in data collection for the thermocouple protocol. Images are sorted by species and then by individual flower, flower file names are formatted as [flower identifier used for sorting e.g. ‘D’][number]. [file 13007_2021_721_MOESM8_ESM.zip › Thermocouple IR images/Bellis/D18/IR_74891.jpg]

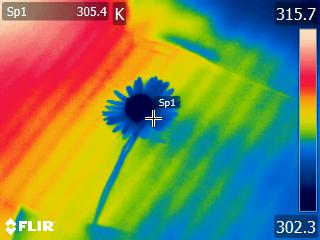

Supplement: Supplementary file 8 — Additional file 8. Thermocouple estimation IR images. File containing the thermal imaging (and paired photographs) of all images used in data collection for the thermocouple protocol. Images are sorted by species and then by individual flower, flower file names are formatted as [flower identifier used for sorting e.g. ‘D’][number]. [file 13007_2021_721_MOESM8_ESM.zip › Thermocouple IR images/Bellis/D18/IR_74895.jpg]

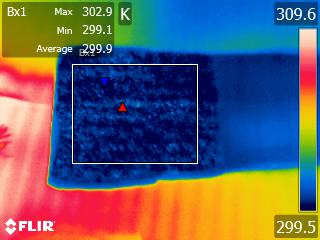

Supplement: Supplementary file 8 — Additional file 8. Thermocouple estimation IR images. File containing the thermal imaging (and paired photographs) of all images used in data collection for the thermocouple protocol. Images are sorted by species and then by individual flower, flower file names are formatted as [flower identifier used for sorting e.g. ‘D’][number]. [file 13007_2021_721_MOESM8_ESM.zip › Thermocouple IR images/Bellis/D18/IR_74897.jpg]

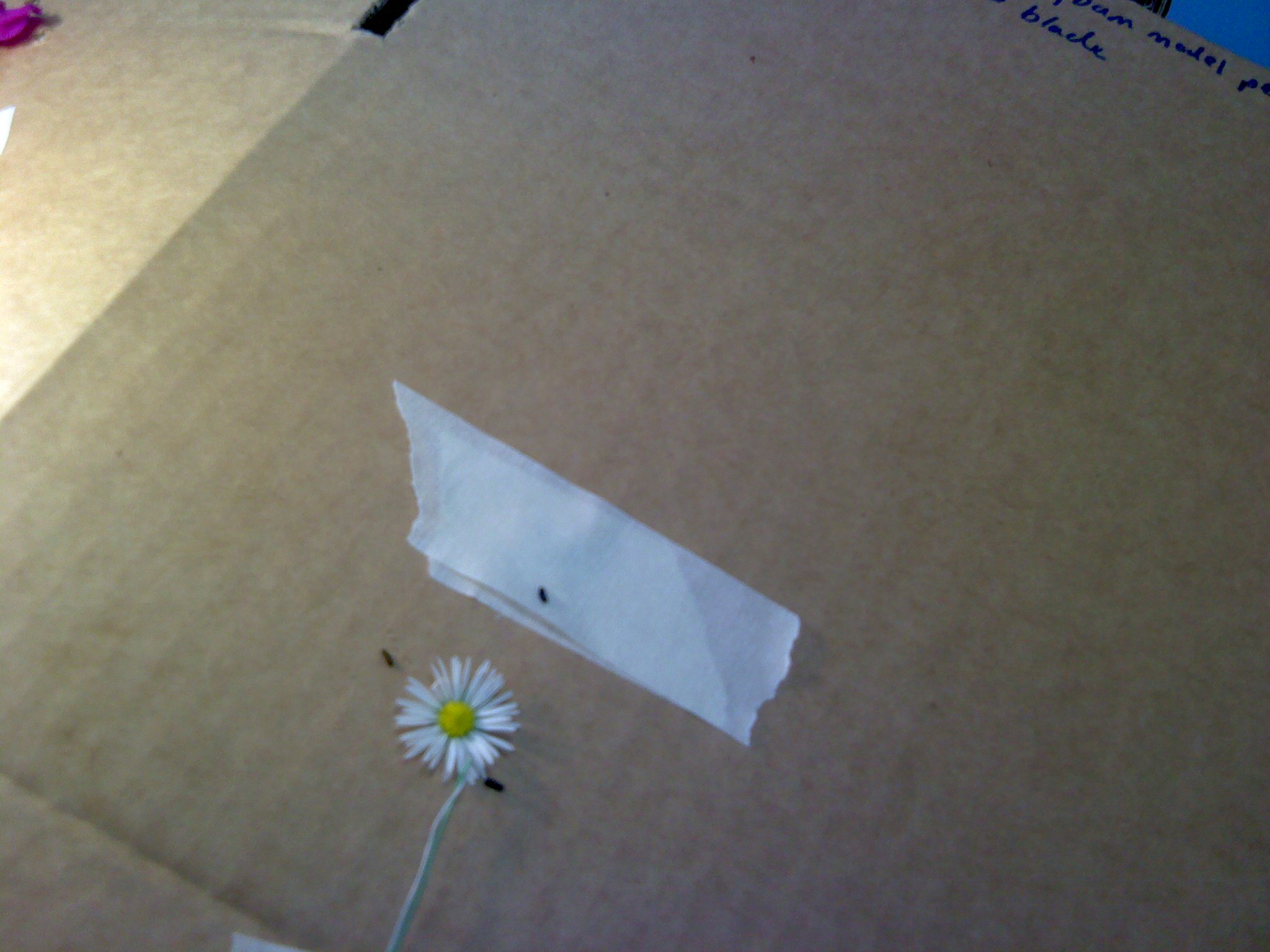

Supplement: Supplementary file 8 — Additional file 8. Thermocouple estimation IR images. File containing the thermal imaging (and paired photographs) of all images used in data collection for the thermocouple protocol. Images are sorted by species and then by individual flower, flower file names are formatted as [flower identifier used for sorting e.g. ‘D’][number]. [file 13007_2021_721_MOESM8_ESM.zip › Thermocouple IR images/Bellis/D19/DC_74900.jpg]

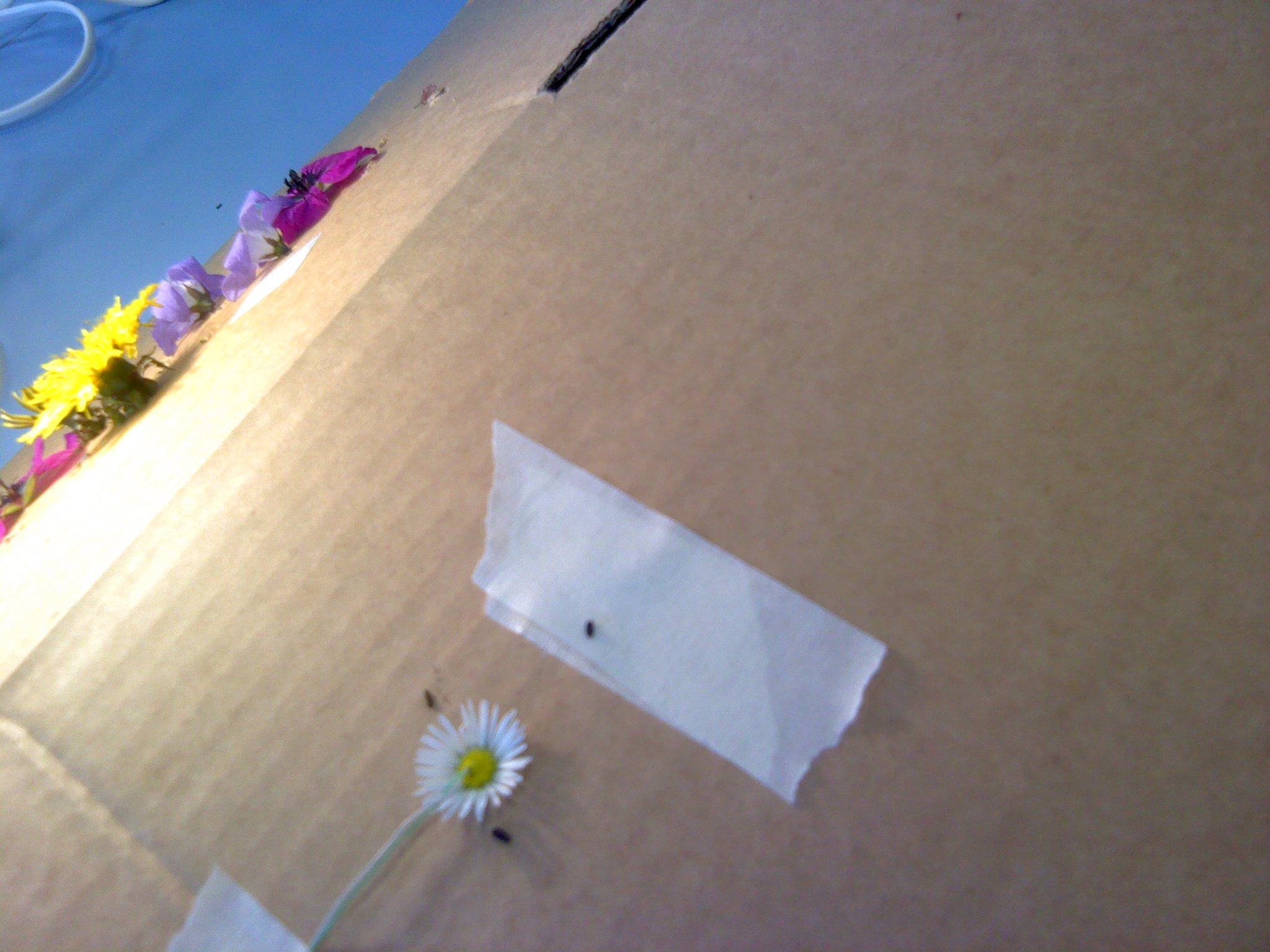

Supplement: Supplementary file 8 — Additional file 8. Thermocouple estimation IR images. File containing the thermal imaging (and paired photographs) of all images used in data collection for the thermocouple protocol. Images are sorted by species and then by individual flower, flower file names are formatted as [flower identifier used for sorting e.g. ‘D’][number]. [file 13007_2021_721_MOESM8_ESM.zip › Thermocouple IR images/Bellis/D19/DC_74902.jpg]

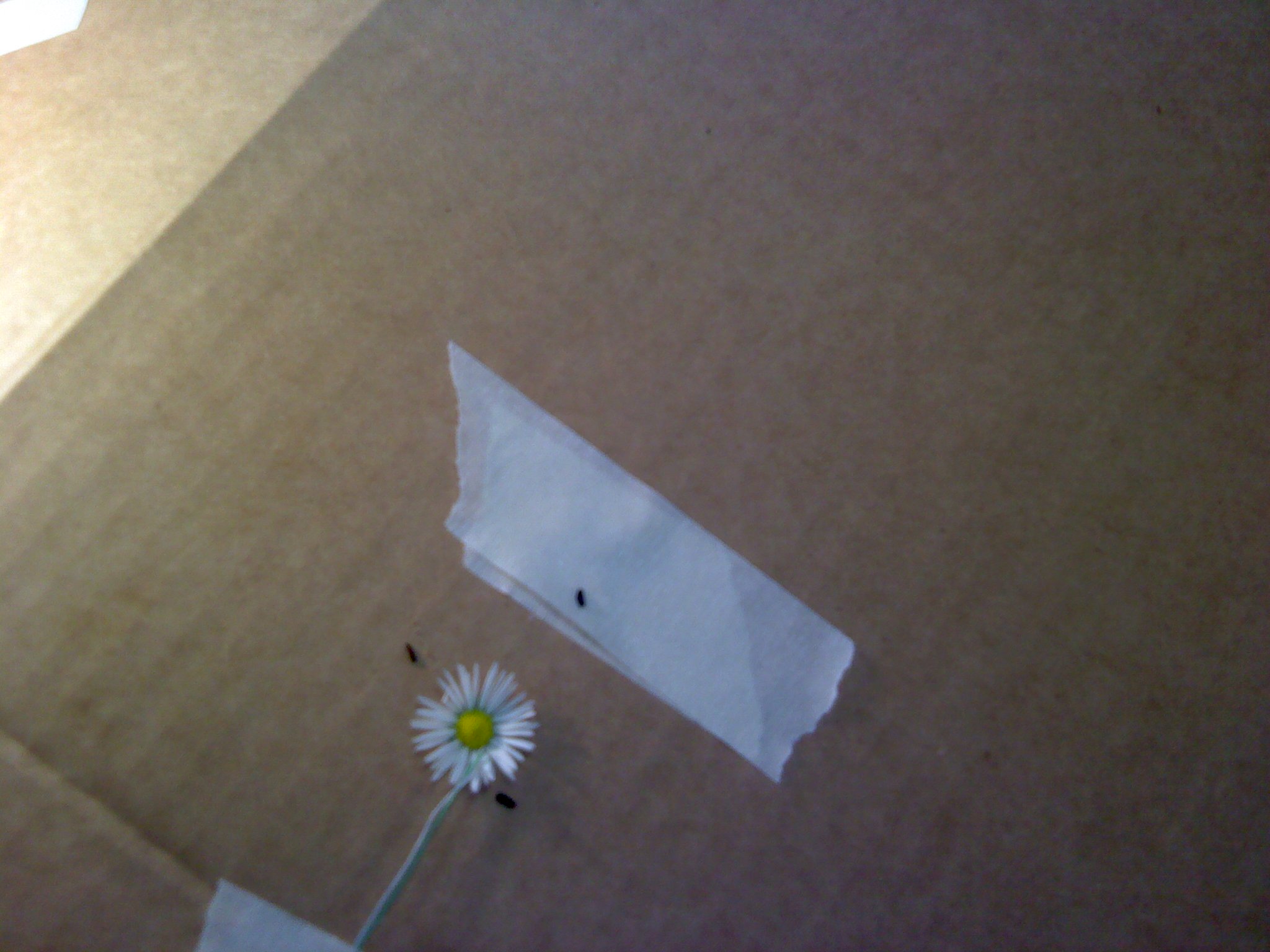

Supplement: Supplementary file 8 — Additional file 8. Thermocouple estimation IR images. File containing the thermal imaging (and paired photographs) of all images used in data collection for the thermocouple protocol. Images are sorted by species and then by individual flower, flower file names are formatted as [flower identifier used for sorting e.g. ‘D’][number]. [file 13007_2021_721_MOESM8_ESM.zip › Thermocouple IR images/Bellis/D19/DC_74906.jpg]

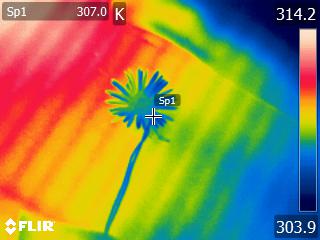

Supplement: Supplementary file 8 — Additional file 8. Thermocouple estimation IR images. File containing the thermal imaging (and paired photographs) of all images used in data collection for the thermocouple protocol. Images are sorted by species and then by individual flower, flower file names are formatted as [flower identifier used for sorting e.g. ‘D’][number]. [file 13007_2021_721_MOESM8_ESM.zip › Thermocouple IR images/Bellis/D19/IR_74899.jpg]

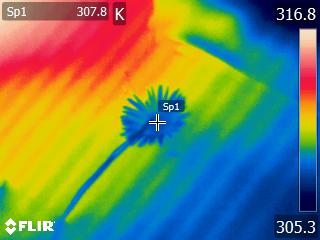

Supplement: Supplementary file 8 — Additional file 8. Thermocouple estimation IR images. File containing the thermal imaging (and paired photographs) of all images used in data collection for the thermocouple protocol. Images are sorted by species and then by individual flower, flower file names are formatted as [flower identifier used for sorting e.g. ‘D’][number]. [file 13007_2021_721_MOESM8_ESM.zip › Thermocouple IR images/Bellis/D19/IR_74901.jpg]

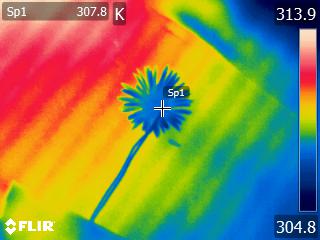

Supplement: Supplementary file 8 — Additional file 8. Thermocouple estimation IR images. File containing the thermal imaging (and paired photographs) of all images used in data collection for the thermocouple protocol. Images are sorted by species and then by individual flower, flower file names are formatted as [flower identifier used for sorting e.g. ‘D’][number]. [file 13007_2021_721_MOESM8_ESM.zip › Thermocouple IR images/Bellis/D19/IR_74905.jpg]

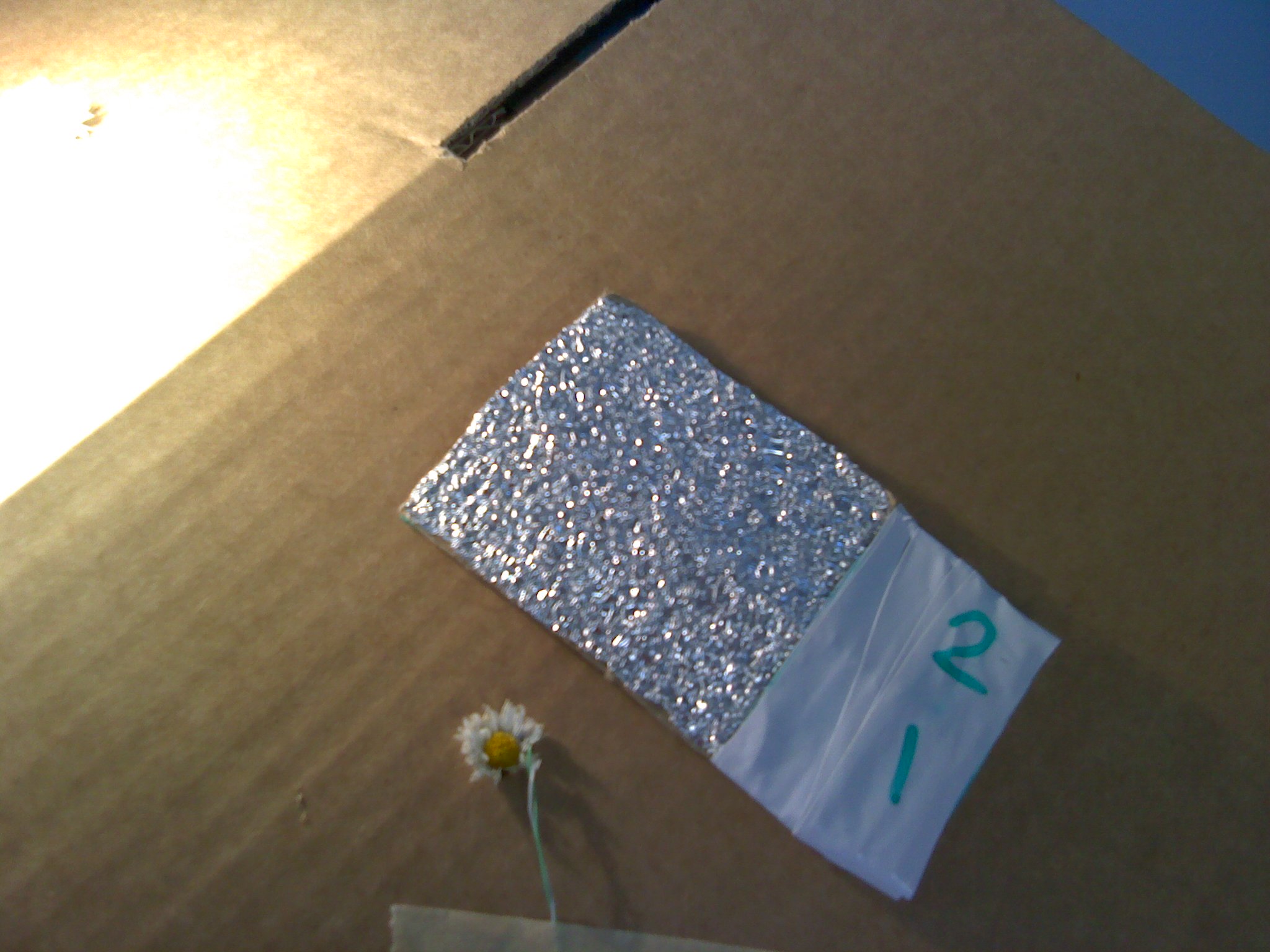

Supplement: Supplementary file 8 — Additional file 8. Thermocouple estimation IR images. File containing the thermal imaging (and paired photographs) of all images used in data collection for the thermocouple protocol. Images are sorted by species and then by individual flower, flower file names are formatted as [flower identifier used for sorting e.g. ‘D’][number]. [file 13007_2021_721_MOESM8_ESM.zip › Thermocouple IR images/Bellis/D2/DC_4882.jpg]

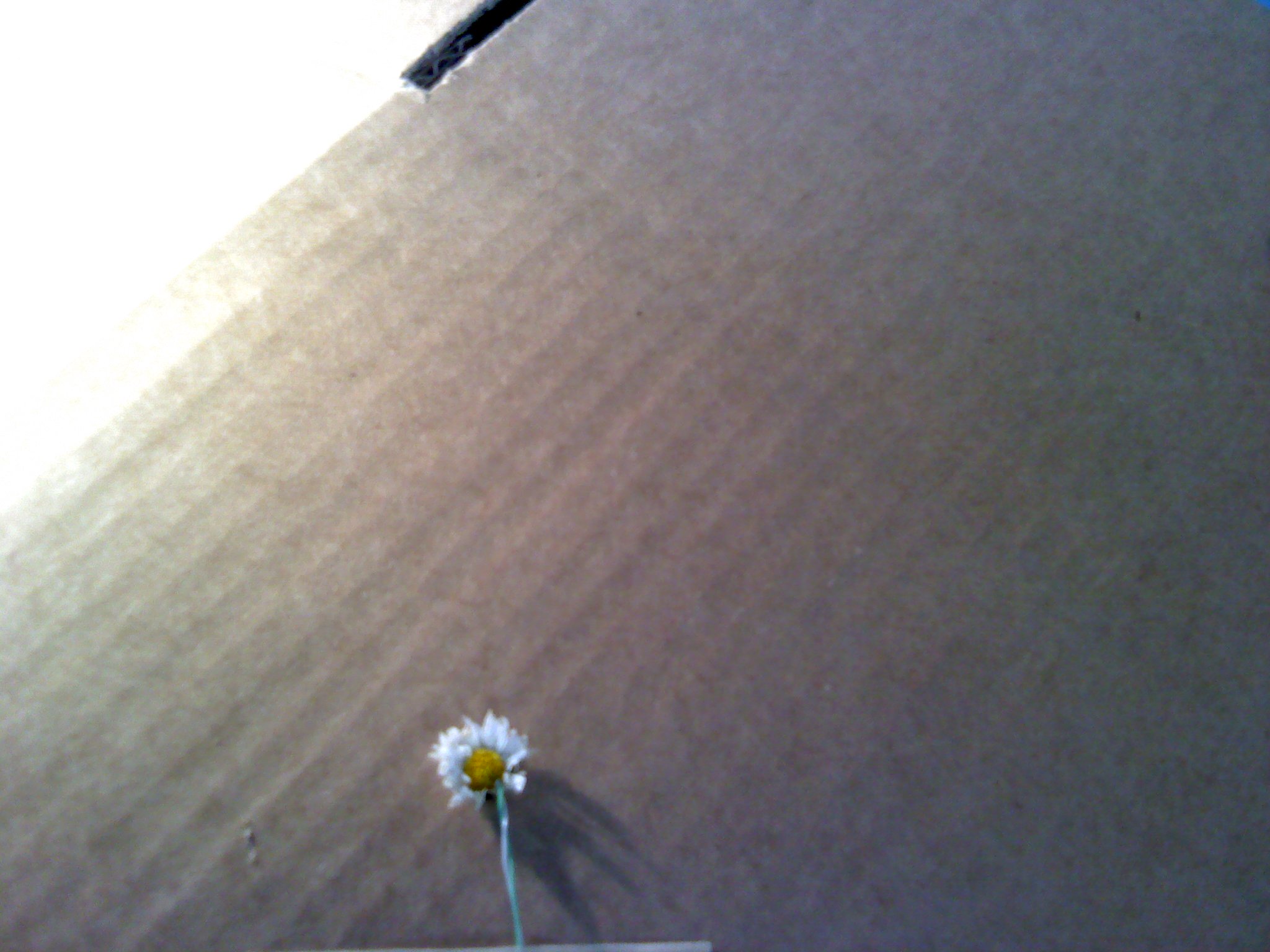

Supplement: Supplementary file 8 — Additional file 8. Thermocouple estimation IR images. File containing the thermal imaging (and paired photographs) of all images used in data collection for the thermocouple protocol. Images are sorted by species and then by individual flower, flower file names are formatted as [flower identifier used for sorting e.g. ‘D’][number]. [file 13007_2021_721_MOESM8_ESM.zip › Thermocouple IR images/Bellis/D2/DC_4884.jpg]

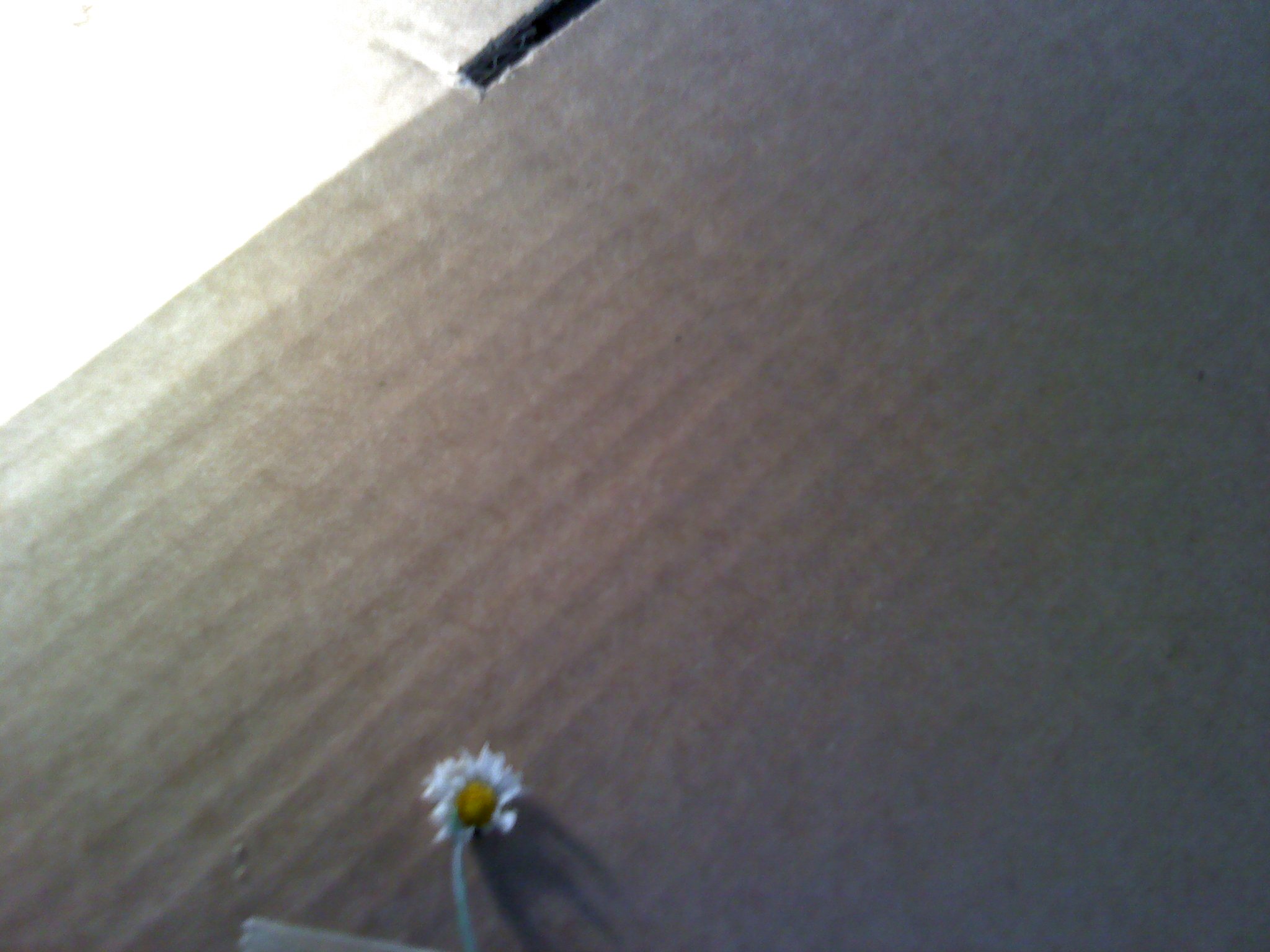

Supplement: Supplementary file 8 — Additional file 8. Thermocouple estimation IR images. File containing the thermal imaging (and paired photographs) of all images used in data collection for the thermocouple protocol. Images are sorted by species and then by individual flower, flower file names are formatted as [flower identifier used for sorting e.g. ‘D’][number]. [file 13007_2021_721_MOESM8_ESM.zip › Thermocouple IR images/Bellis/D2/DC_4886.jpg]

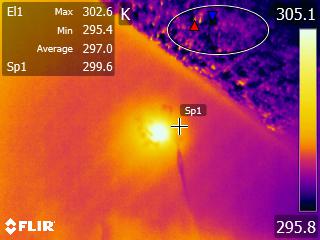

Supplement: Supplementary file 8 — Additional file 8. Thermocouple estimation IR images. File containing the thermal imaging (and paired photographs) of all images used in data collection for the thermocouple protocol. Images are sorted by species and then by individual flower, flower file names are formatted as [flower identifier used for sorting e.g. ‘D’][number]. [file 13007_2021_721_MOESM8_ESM.zip › Thermocouple IR images/Bellis/D2/IR_4881.jpg]

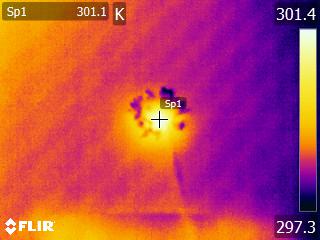

Supplement: Supplementary file 8 — Additional file 8. Thermocouple estimation IR images. File containing the thermal imaging (and paired photographs) of all images used in data collection for the thermocouple protocol. Images are sorted by species and then by individual flower, flower file names are formatted as [flower identifier used for sorting e.g. ‘D’][number]. [file 13007_2021_721_MOESM8_ESM.zip › Thermocouple IR images/Bellis/D2/IR_4883.jpg]

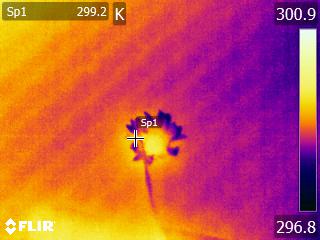

Supplement: Supplementary file 8 — Additional file 8. Thermocouple estimation IR images. File containing the thermal imaging (and paired photographs) of all images used in data collection for the thermocouple protocol. Images are sorted by species and then by individual flower, flower file names are formatted as [flower identifier used for sorting e.g. ‘D’][number]. [file 13007_2021_721_MOESM8_ESM.zip › Thermocouple IR images/Bellis/D2/IR_4885.jpg]

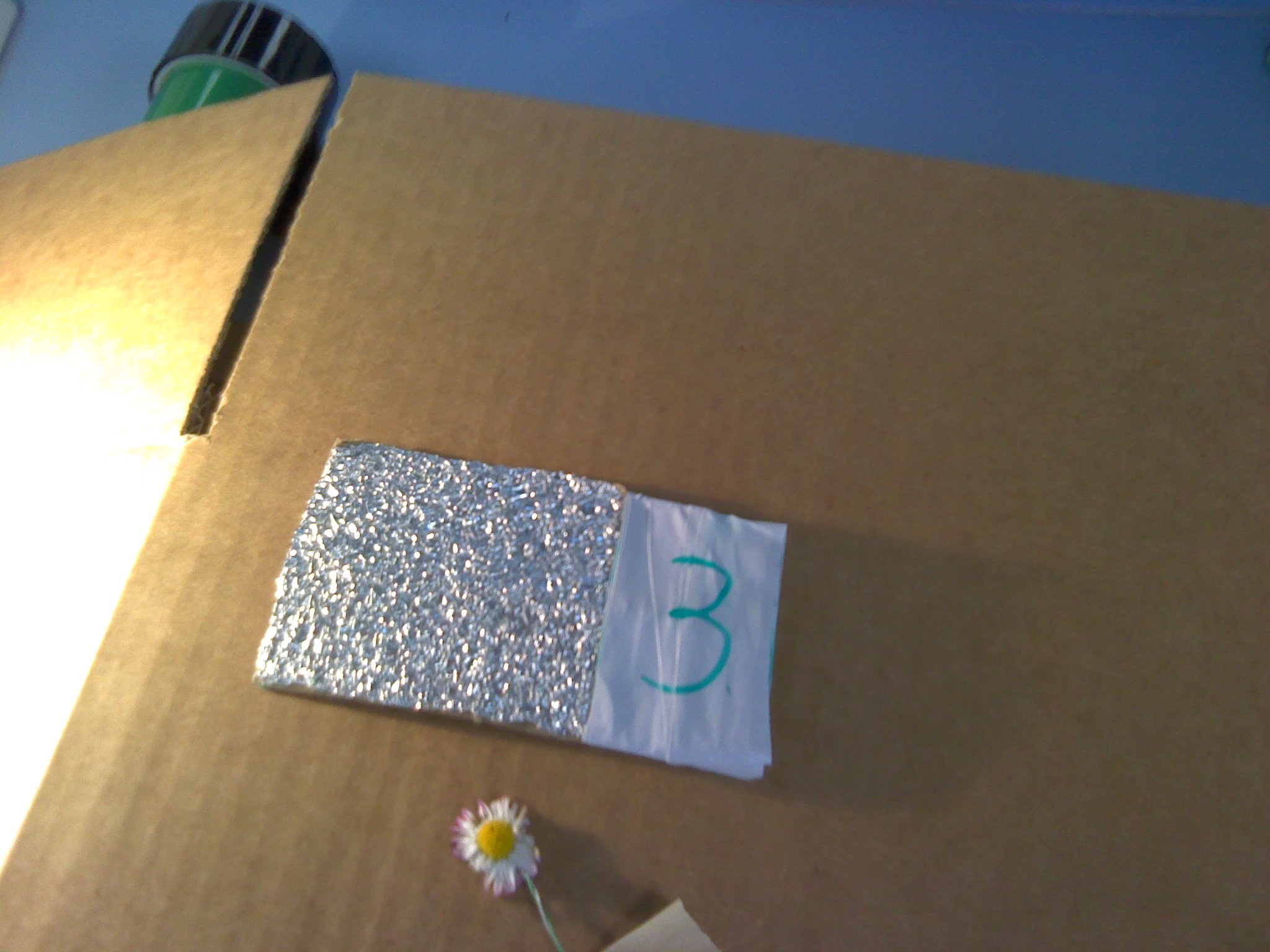

Supplement: Supplementary file 8 — Additional file 8. Thermocouple estimation IR images. File containing the thermal imaging (and paired photographs) of all images used in data collection for the thermocouple protocol. Images are sorted by species and then by individual flower, flower file names are formatted as [flower identifier used for sorting e.g. ‘D’][number]. [file 13007_2021_721_MOESM8_ESM.zip › Thermocouple IR images/Bellis/D3/DC_4888.jpg]

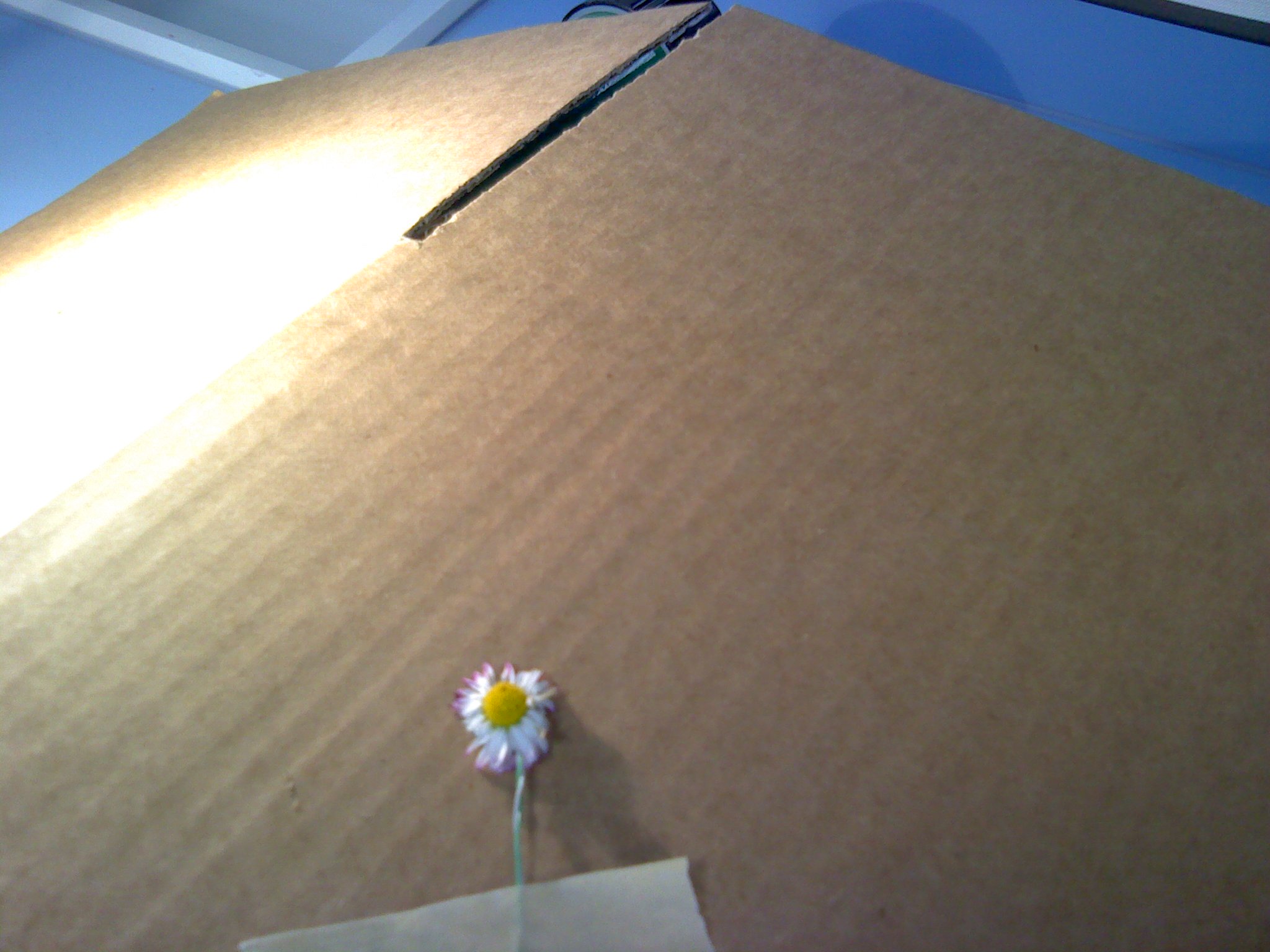

Supplement: Supplementary file 8 — Additional file 8. Thermocouple estimation IR images. File containing the thermal imaging (and paired photographs) of all images used in data collection for the thermocouple protocol. Images are sorted by species and then by individual flower, flower file names are formatted as [flower identifier used for sorting e.g. ‘D’][number]. [file 13007_2021_721_MOESM8_ESM.zip › Thermocouple IR images/Bellis/D3/DC_4892.jpg]

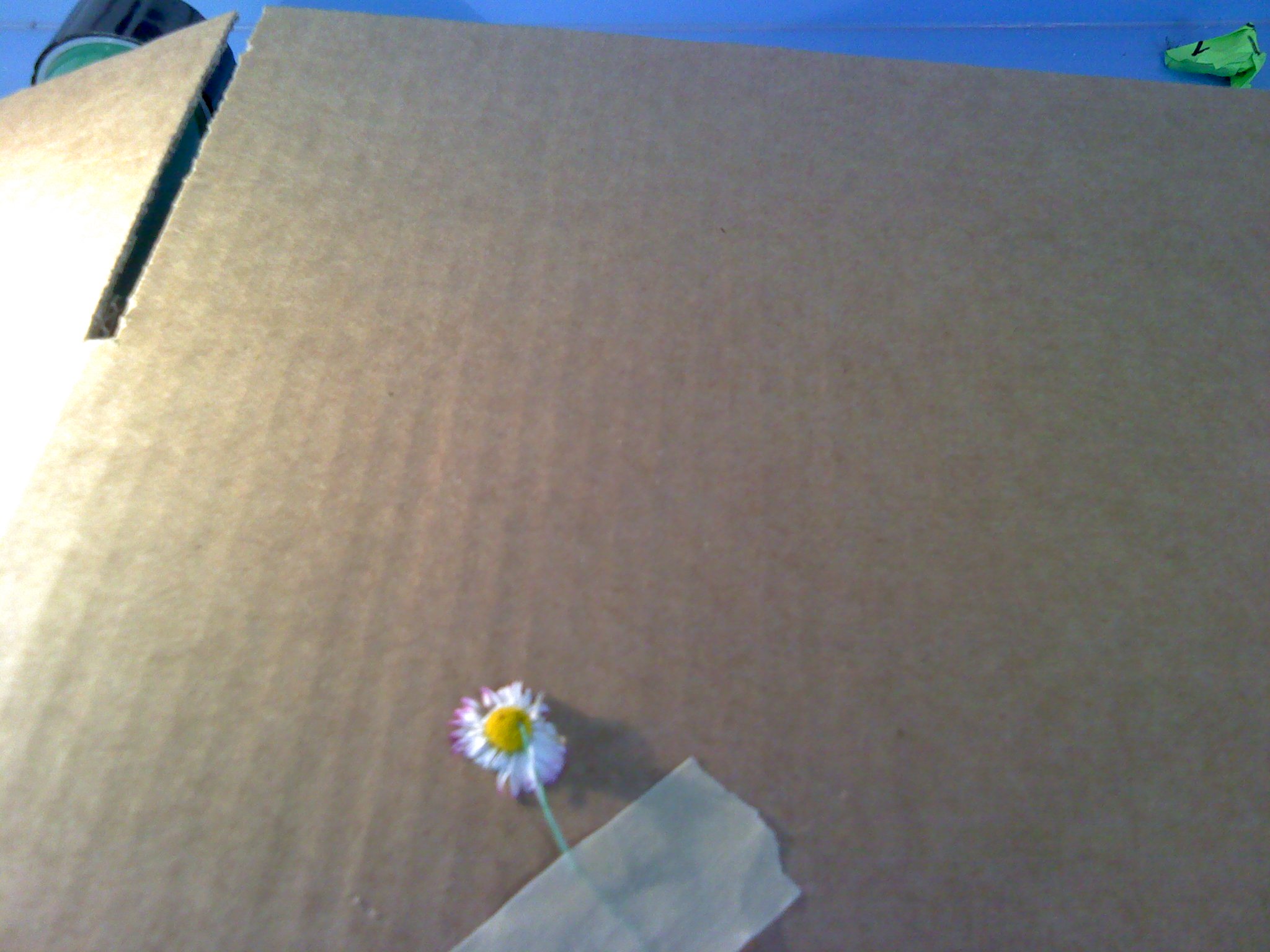

Supplement: Supplementary file 8 — Additional file 8. Thermocouple estimation IR images. File containing the thermal imaging (and paired photographs) of all images used in data collection for the thermocouple protocol. Images are sorted by species and then by individual flower, flower file names are formatted as [flower identifier used for sorting e.g. ‘D’][number]. [file 13007_2021_721_MOESM8_ESM.zip › Thermocouple IR images/Bellis/D3/DC_4896.jpg]

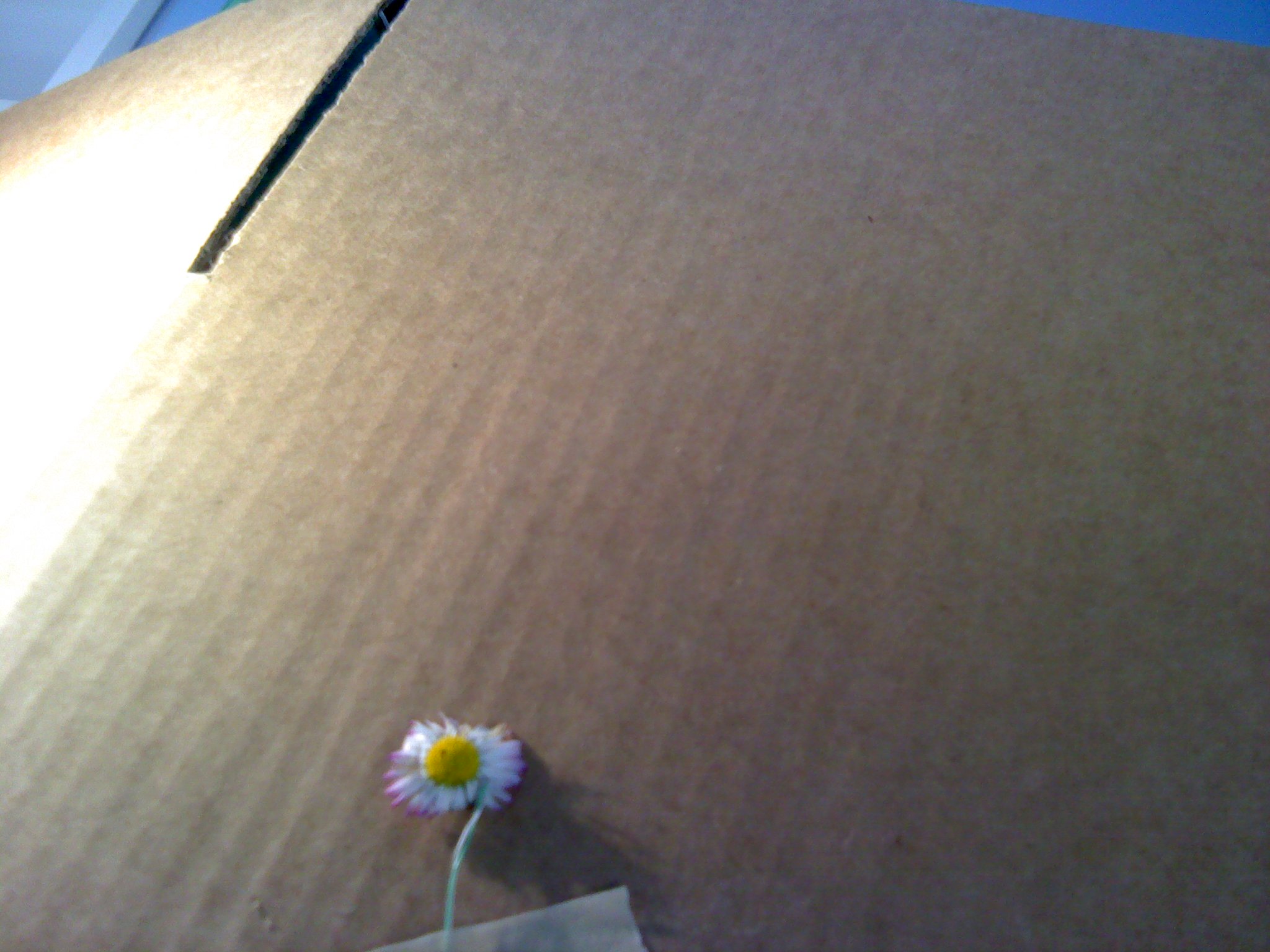

Supplement: Supplementary file 8 — Additional file 8. Thermocouple estimation IR images. File containing the thermal imaging (and paired photographs) of all images used in data collection for the thermocouple protocol. Images are sorted by species and then by individual flower, flower file names are formatted as [flower identifier used for sorting e.g. ‘D’][number]. [file 13007_2021_721_MOESM8_ESM.zip › Thermocouple IR images/Bellis/D3/DC_4898.jpg]

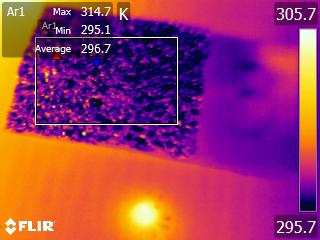

Supplement: Supplementary file 8 — Additional file 8. Thermocouple estimation IR images. File containing the thermal imaging (and paired photographs) of all images used in data collection for the thermocouple protocol. Images are sorted by species and then by individual flower, flower file names are formatted as [flower identifier used for sorting e.g. ‘D’][number]. [file 13007_2021_721_MOESM8_ESM.zip › Thermocouple IR images/Bellis/D3/IR_4887.jpg]

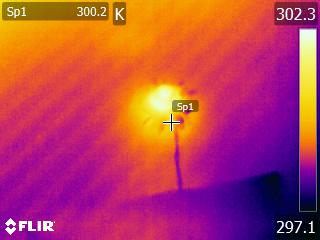

Supplement: Supplementary file 8 — Additional file 8. Thermocouple estimation IR images. File containing the thermal imaging (and paired photographs) of all images used in data collection for the thermocouple protocol. Images are sorted by species and then by individual flower, flower file names are formatted as [flower identifier used for sorting e.g. ‘D’][number]. [file 13007_2021_721_MOESM8_ESM.zip › Thermocouple IR images/Bellis/D3/IR_4891.jpg]

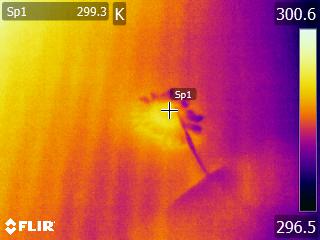

Supplement: Supplementary file 8 — Additional file 8. Thermocouple estimation IR images. File containing the thermal imaging (and paired photographs) of all images used in data collection for the thermocouple protocol. Images are sorted by species and then by individual flower, flower file names are formatted as [flower identifier used for sorting e.g. ‘D’][number]. [file 13007_2021_721_MOESM8_ESM.zip › Thermocouple IR images/Bellis/D3/IR_4895.jpg]

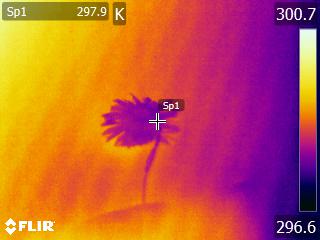

Supplement: Supplementary file 8 — Additional file 8. Thermocouple estimation IR images. File containing the thermal imaging (and paired photographs) of all images used in data collection for the thermocouple protocol. Images are sorted by species and then by individual flower, flower file names are formatted as [flower identifier used for sorting e.g. ‘D’][number]. [file 13007_2021_721_MOESM8_ESM.zip › Thermocouple IR images/Bellis/D3/IR_4897.jpg]

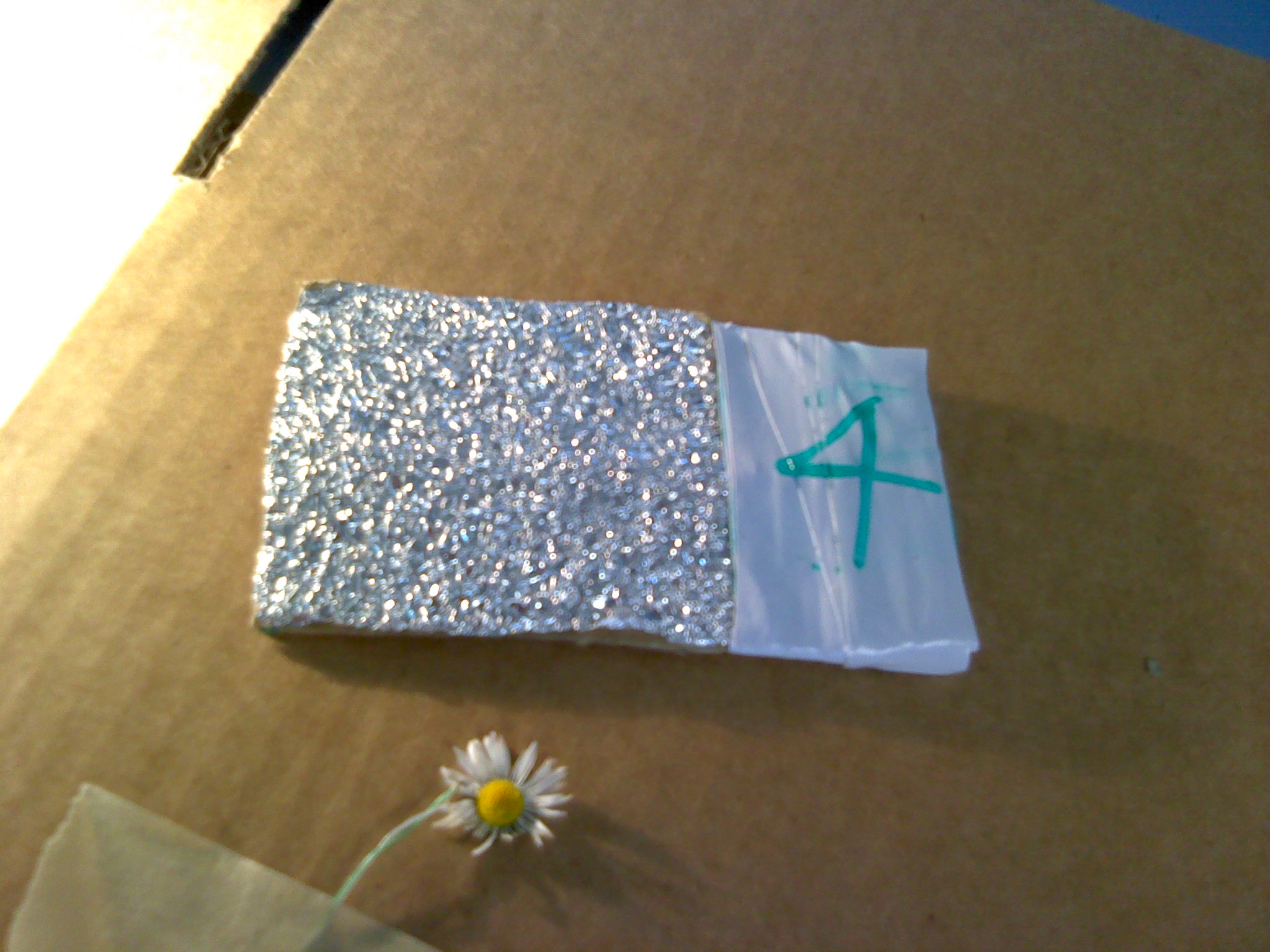

Supplement: Supplementary file 8 — Additional file 8. Thermocouple estimation IR images. File containing the thermal imaging (and paired photographs) of all images used in data collection for the thermocouple protocol. Images are sorted by species and then by individual flower, flower file names are formatted as [flower identifier used for sorting e.g. ‘D’][number]. [file 13007_2021_721_MOESM8_ESM.zip › Thermocouple IR images/Bellis/D4/DC_4902.jpg]
