# Supplementary figures and images for: Floral infrared emissivity estimates using simple tools (part 3 of 4)
Source: Plant Methods. 2021 Feb 25;17:23. doi: 10.1186/s13007-021-00721-w (PMC7905901; doi:10.1186/s13007-021-00721-w)

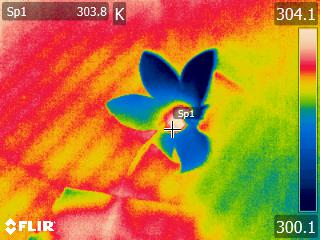

Supplement: Supplementary file 8 — Additional file 8. Thermocouple estimation IR images. File containing the thermal imaging (and paired photographs) of all images used in data collection for the thermocouple protocol. Images are sorted by species and then by individual flower, flower file names are formatted as [flower identifier used for sorting e.g. ‘D’][number]. [file 13007_2021_721_MOESM8_ESM.zip › Thermocouple IR images/Campanula/camp16/IR_58589.jpg]

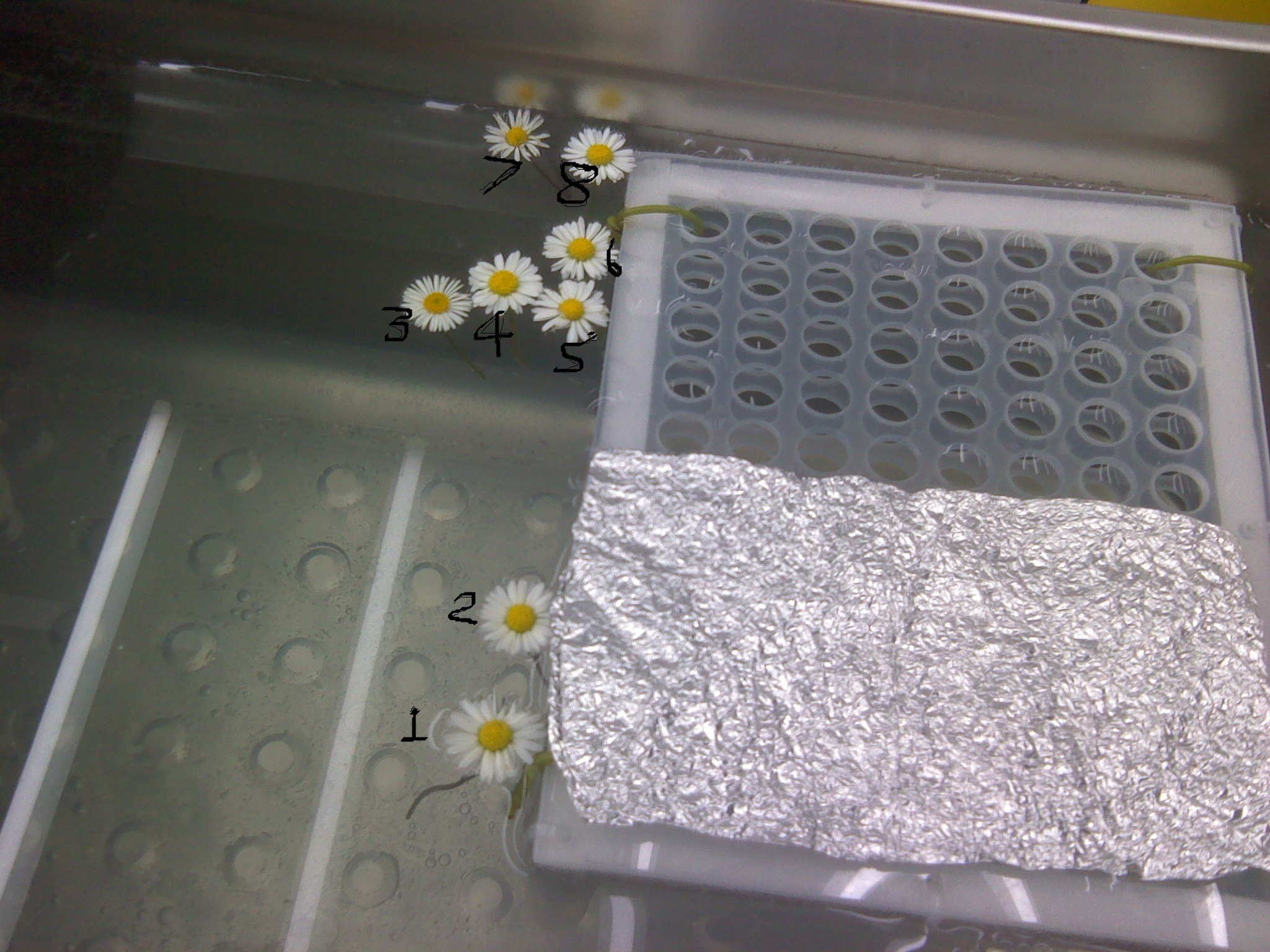

Supplement: Supplementary file 9 — Additional file 9. Water bath estimation IR images. File containing the thermal imaging (and paired photographs) of all images used in data collection for the water bath protocol. Images are sorted by species, set temperature of the water bath and then by individual flower, flower file names are formatted as [flower identifier used for sorting e.g. ‘D’][number]. Additionally, where available, photos are provided to aid identification of individual flowers. [file 13007_2021_721_MOESM9_ESM.zip › Bath IR images/Bellis/1to8.jpg]

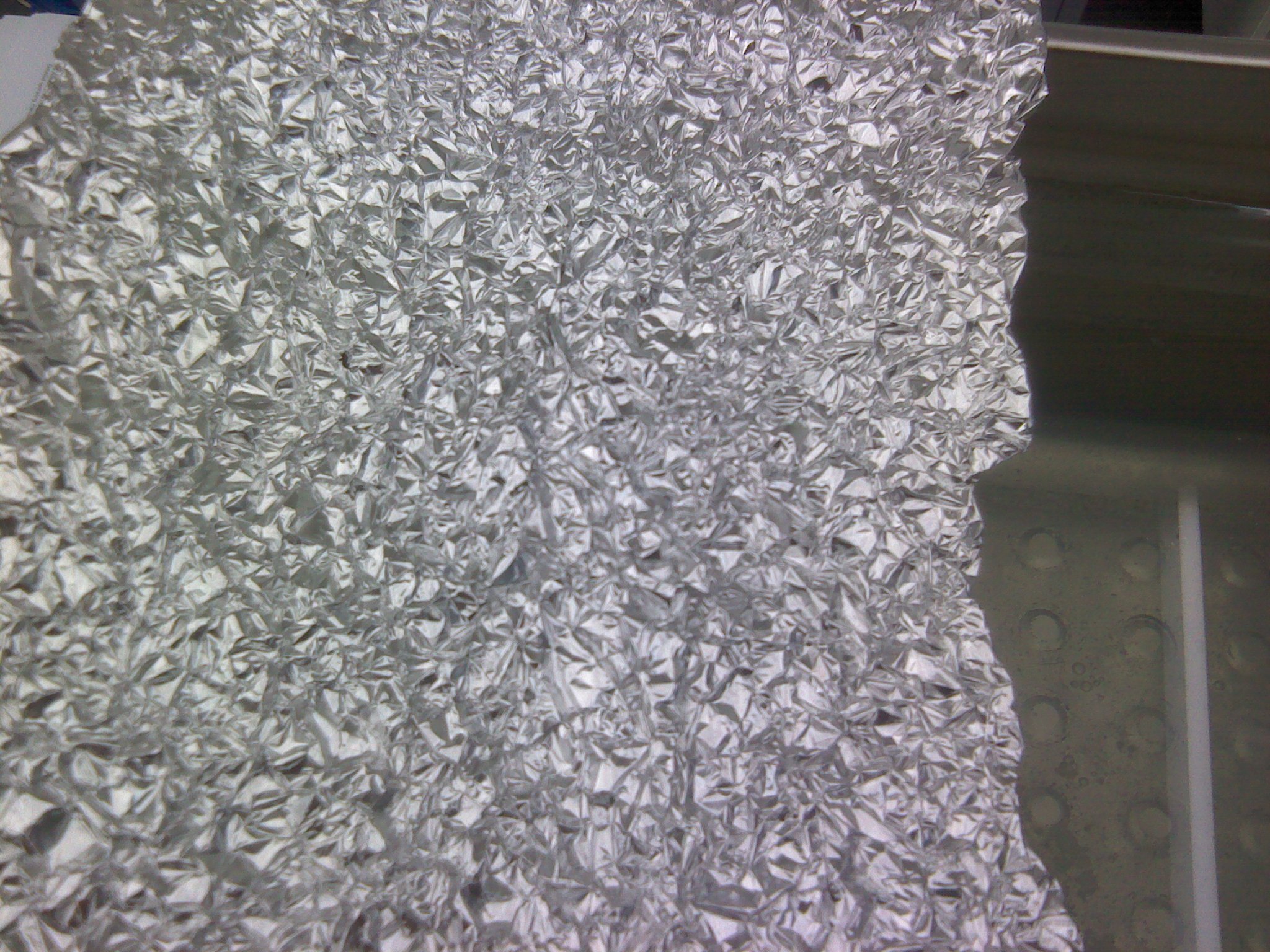

Supplement: Supplementary file 9 — Additional file 9. Water bath estimation IR images. File containing the thermal imaging (and paired photographs) of all images used in data collection for the water bath protocol. Images are sorted by species, set temperature of the water bath and then by individual flower, flower file names are formatted as [flower identifier used for sorting e.g. ‘D’][number]. Additionally, where available, photos are provided to aid identification of individual flowers. [file 13007_2021_721_MOESM9_ESM.zip › Bath IR images/Bellis/35c/B1/DC_89419.jpg]

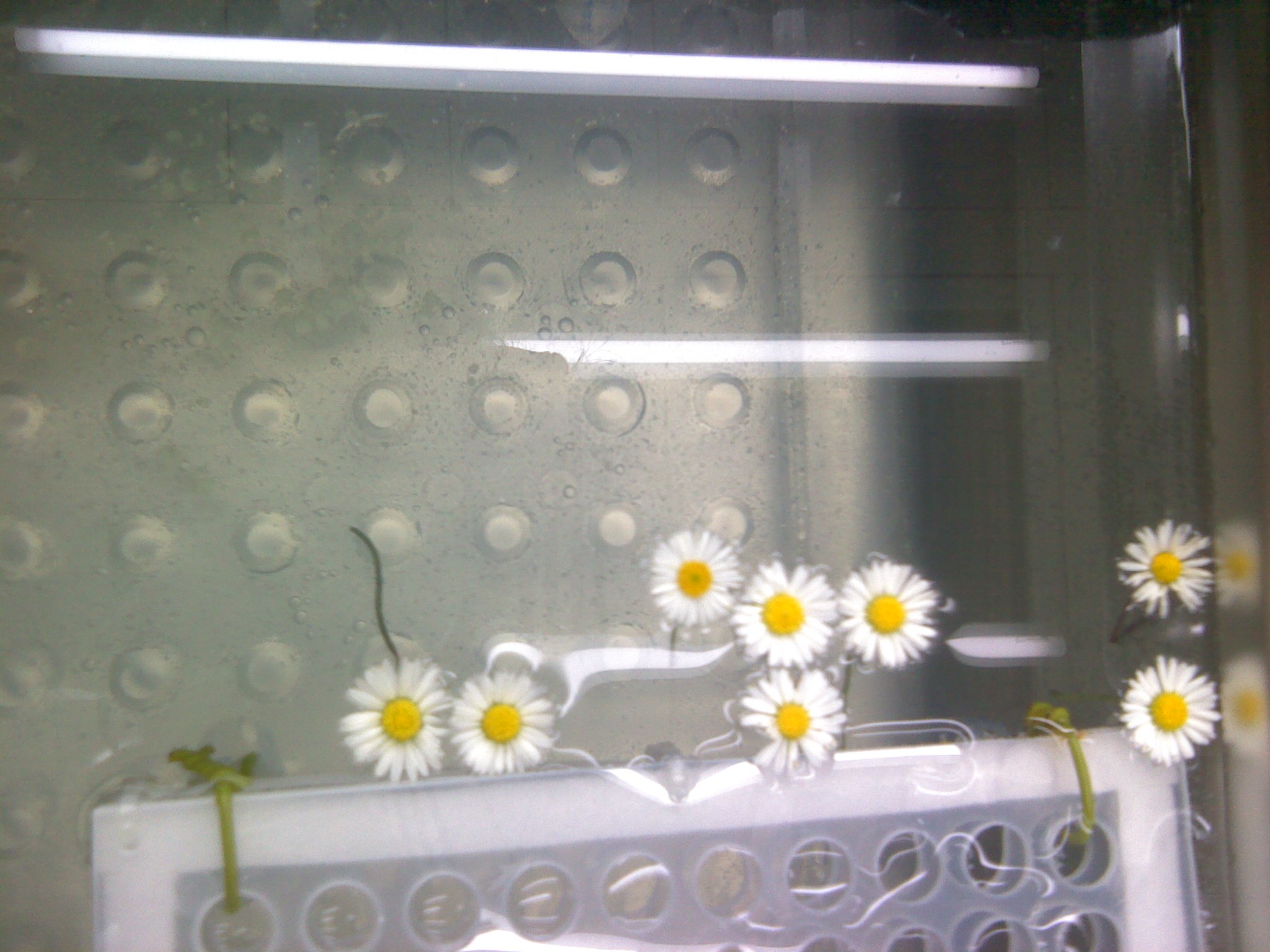

Supplement: Supplementary file 9 — Additional file 9. Water bath estimation IR images. File containing the thermal imaging (and paired photographs) of all images used in data collection for the water bath protocol. Images are sorted by species, set temperature of the water bath and then by individual flower, flower file names are formatted as [flower identifier used for sorting e.g. ‘D’][number]. Additionally, where available, photos are provided to aid identification of individual flowers. [file 13007_2021_721_MOESM9_ESM.zip › Bath IR images/Bellis/35c/B1/DC_89421.jpg]

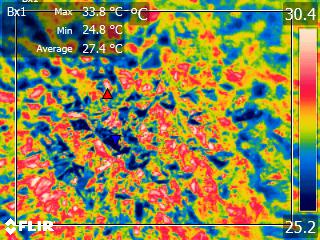

Supplement: Supplementary file 9 — Additional file 9. Water bath estimation IR images. File containing the thermal imaging (and paired photographs) of all images used in data collection for the water bath protocol. Images are sorted by species, set temperature of the water bath and then by individual flower, flower file names are formatted as [flower identifier used for sorting e.g. ‘D’][number]. Additionally, where available, photos are provided to aid identification of individual flowers. [file 13007_2021_721_MOESM9_ESM.zip › Bath IR images/Bellis/35c/B1/IR_89418.jpg]

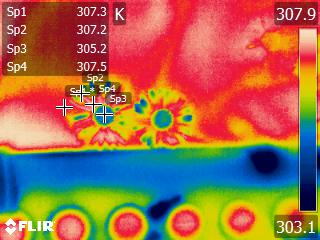

Supplement: Supplementary file 9 — Additional file 9. Water bath estimation IR images. File containing the thermal imaging (and paired photographs) of all images used in data collection for the water bath protocol. Images are sorted by species, set temperature of the water bath and then by individual flower, flower file names are formatted as [flower identifier used for sorting e.g. ‘D’][number]. Additionally, where available, photos are provided to aid identification of individual flowers. [file 13007_2021_721_MOESM9_ESM.zip › Bath IR images/Bellis/35c/B1/IR_89420.jpg]

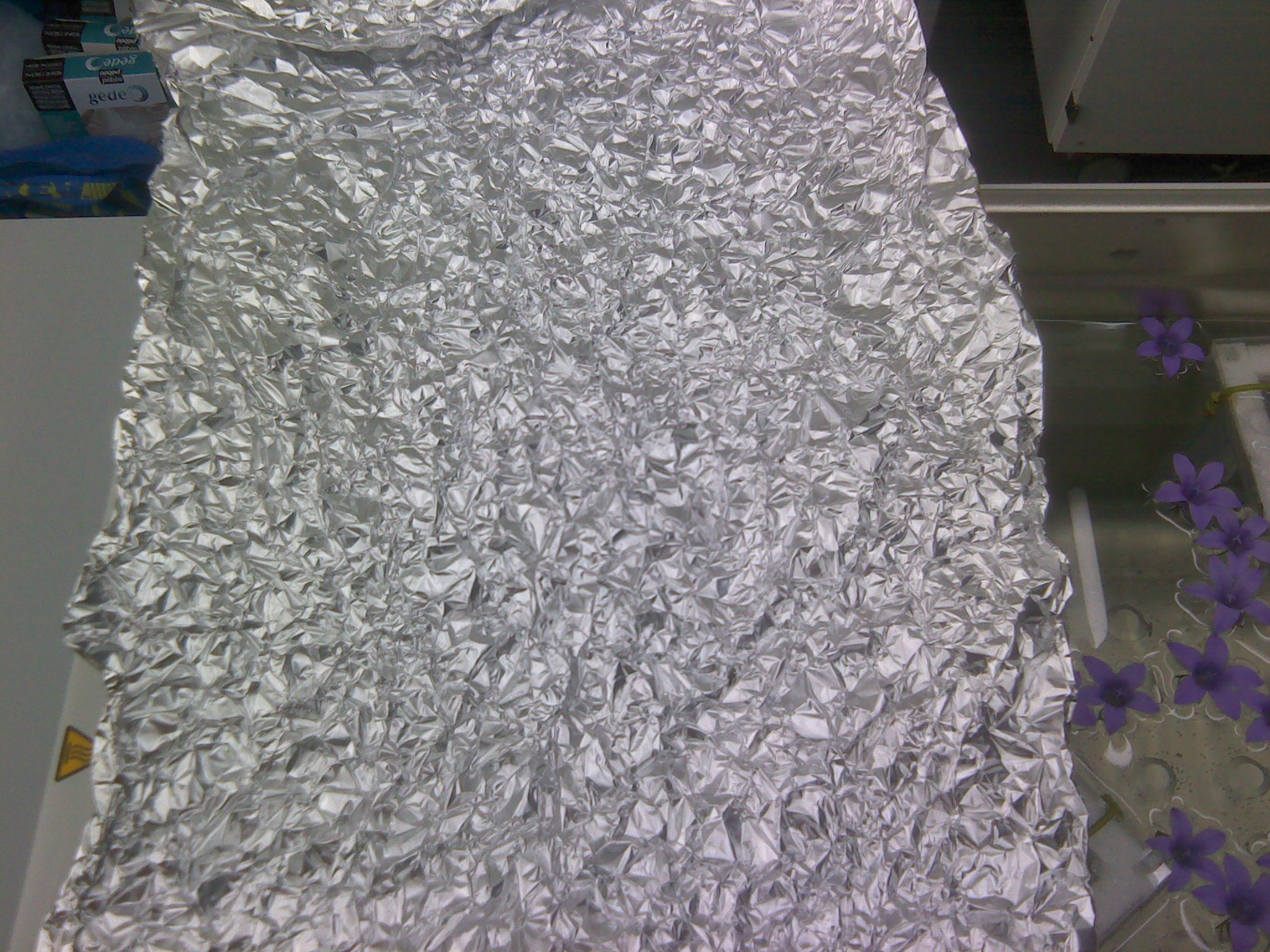

Supplement: Supplementary file 9 — Additional file 9. Water bath estimation IR images. File containing the thermal imaging (and paired photographs) of all images used in data collection for the water bath protocol. Images are sorted by species, set temperature of the water bath and then by individual flower, flower file names are formatted as [flower identifier used for sorting e.g. ‘D’][number]. Additionally, where available, photos are provided to aid identification of individual flowers. [file 13007_2021_721_MOESM9_ESM.zip › Bath IR images/Bellis/35c/B10/DC_89475.jpg]

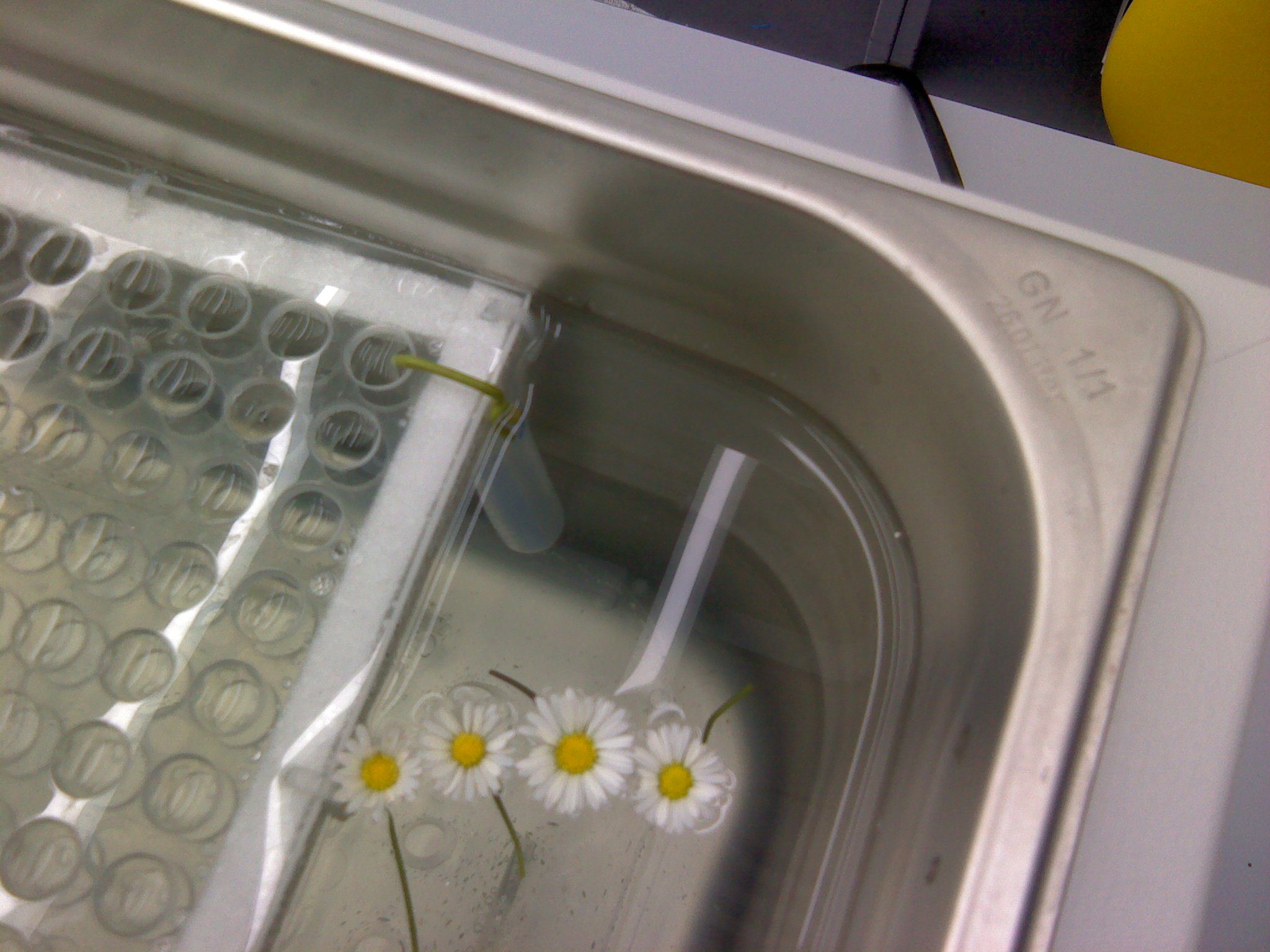

Supplement: Supplementary file 9 — Additional file 9. Water bath estimation IR images. File containing the thermal imaging (and paired photographs) of all images used in data collection for the water bath protocol. Images are sorted by species, set temperature of the water bath and then by individual flower, flower file names are formatted as [flower identifier used for sorting e.g. ‘D’][number]. Additionally, where available, photos are provided to aid identification of individual flowers. [file 13007_2021_721_MOESM9_ESM.zip › Bath IR images/Bellis/35c/B10/DC_89479.jpg]

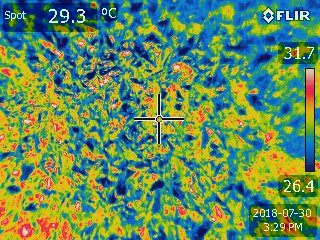

Supplement: Supplementary file 9 — Additional file 9. Water bath estimation IR images. File containing the thermal imaging (and paired photographs) of all images used in data collection for the water bath protocol. Images are sorted by species, set temperature of the water bath and then by individual flower, flower file names are formatted as [flower identifier used for sorting e.g. ‘D’][number]. Additionally, where available, photos are provided to aid identification of individual flowers. [file 13007_2021_721_MOESM9_ESM.zip › Bath IR images/Bellis/35c/B10/IR_89474.jpg]

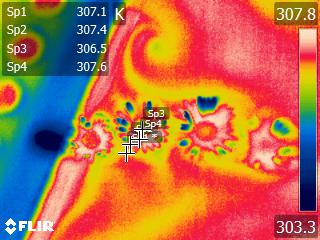

Supplement: Supplementary file 9 — Additional file 9. Water bath estimation IR images. File containing the thermal imaging (and paired photographs) of all images used in data collection for the water bath protocol. Images are sorted by species, set temperature of the water bath and then by individual flower, flower file names are formatted as [flower identifier used for sorting e.g. ‘D’][number]. Additionally, where available, photos are provided to aid identification of individual flowers. [file 13007_2021_721_MOESM9_ESM.zip › Bath IR images/Bellis/35c/B10/IR_89478.jpg]

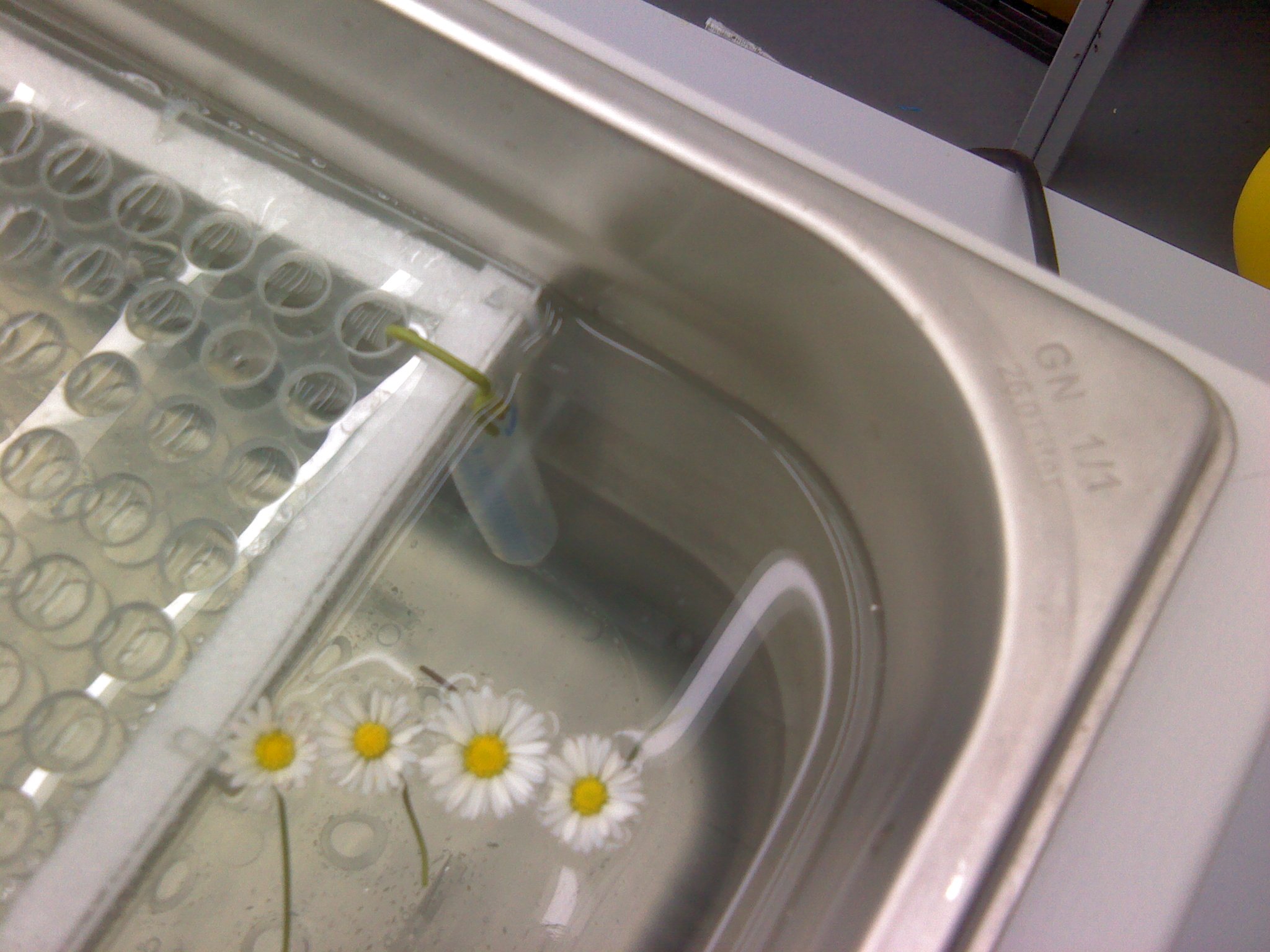

Supplement: Supplementary file 9 — Additional file 9. Water bath estimation IR images. File containing the thermal imaging (and paired photographs) of all images used in data collection for the water bath protocol. Images are sorted by species, set temperature of the water bath and then by individual flower, flower file names are formatted as [flower identifier used for sorting e.g. ‘D’][number]. Additionally, where available, photos are provided to aid identification of individual flowers. [file 13007_2021_721_MOESM9_ESM.zip › Bath IR images/Bellis/35c/B11/DC_89481.jpg]

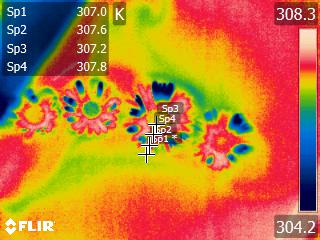

Supplement: Supplementary file 9 — Additional file 9. Water bath estimation IR images. File containing the thermal imaging (and paired photographs) of all images used in data collection for the water bath protocol. Images are sorted by species, set temperature of the water bath and then by individual flower, flower file names are formatted as [flower identifier used for sorting e.g. ‘D’][number]. Additionally, where available, photos are provided to aid identification of individual flowers. [file 13007_2021_721_MOESM9_ESM.zip › Bath IR images/Bellis/35c/B11/IR_89480.jpg]

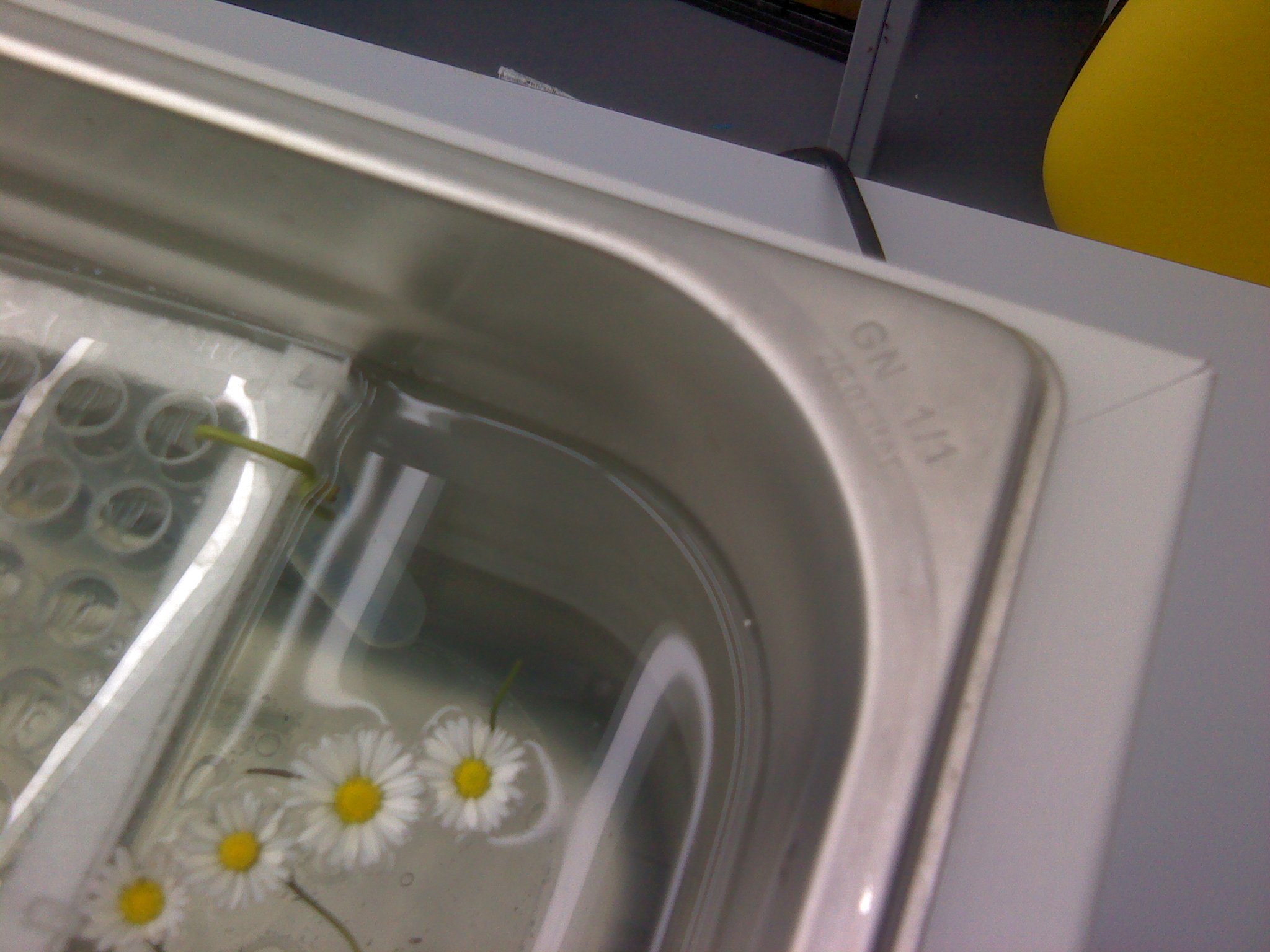

Supplement: Supplementary file 9 — Additional file 9. Water bath estimation IR images. File containing the thermal imaging (and paired photographs) of all images used in data collection for the water bath protocol. Images are sorted by species, set temperature of the water bath and then by individual flower, flower file names are formatted as [flower identifier used for sorting e.g. ‘D’][number]. Additionally, where available, photos are provided to aid identification of individual flowers. [file 13007_2021_721_MOESM9_ESM.zip › Bath IR images/Bellis/35c/B12/DC_89483.jpg]

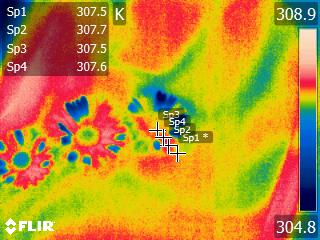

Supplement: Supplementary file 9 — Additional file 9. Water bath estimation IR images. File containing the thermal imaging (and paired photographs) of all images used in data collection for the water bath protocol. Images are sorted by species, set temperature of the water bath and then by individual flower, flower file names are formatted as [flower identifier used for sorting e.g. ‘D’][number]. Additionally, where available, photos are provided to aid identification of individual flowers. [file 13007_2021_721_MOESM9_ESM.zip › Bath IR images/Bellis/35c/B12/IR_89482.jpg]

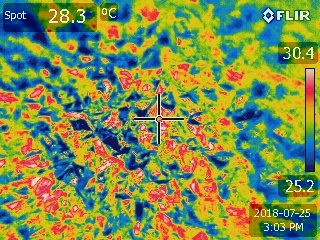

Supplement: Supplementary file 9 — Additional file 9. Water bath estimation IR images. File containing the thermal imaging (and paired photographs) of all images used in data collection for the water bath protocol. Images are sorted by species, set temperature of the water bath and then by individual flower, flower file names are formatted as [flower identifier used for sorting e.g. ‘D’][number]. Additionally, where available, photos are provided to aid identification of individual flowers. [file 13007_2021_721_MOESM9_ESM.zip › Bath IR images/Bellis/35c/B2/IR_89418.jpg]

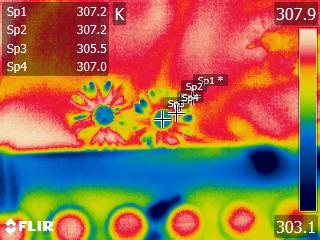

Supplement: Supplementary file 9 — Additional file 9. Water bath estimation IR images. File containing the thermal imaging (and paired photographs) of all images used in data collection for the water bath protocol. Images are sorted by species, set temperature of the water bath and then by individual flower, flower file names are formatted as [flower identifier used for sorting e.g. ‘D’][number]. Additionally, where available, photos are provided to aid identification of individual flowers. [file 13007_2021_721_MOESM9_ESM.zip › Bath IR images/Bellis/35c/B2/IR_89420.jpg]

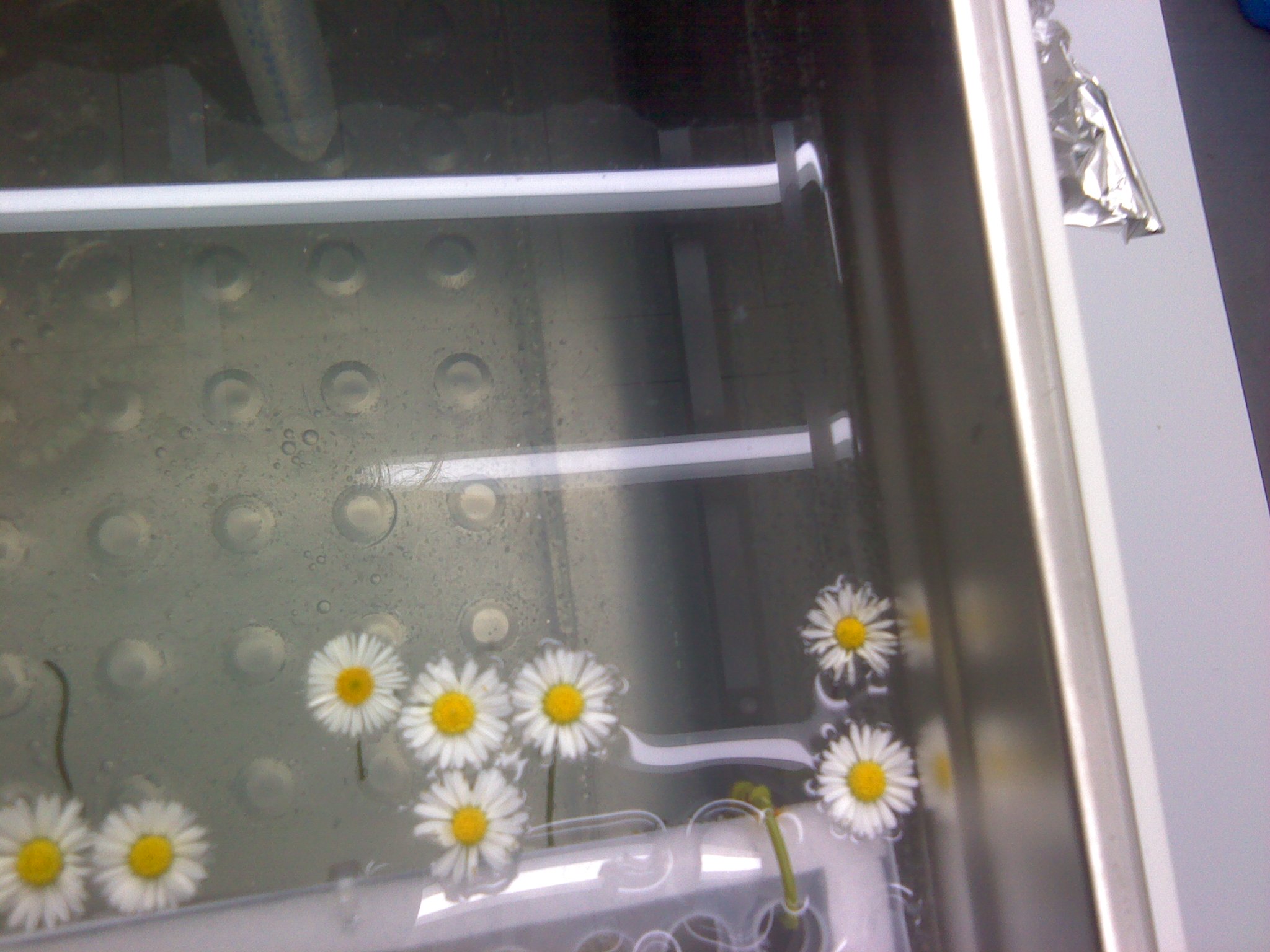

Supplement: Supplementary file 9 — Additional file 9. Water bath estimation IR images. File containing the thermal imaging (and paired photographs) of all images used in data collection for the water bath protocol. Images are sorted by species, set temperature of the water bath and then by individual flower, flower file names are formatted as [flower identifier used for sorting e.g. ‘D’][number]. Additionally, where available, photos are provided to aid identification of individual flowers. [file 13007_2021_721_MOESM9_ESM.zip › Bath IR images/Bellis/35c/B3/DC_89423.jpg]

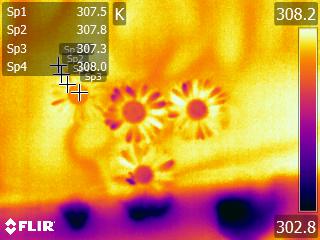

Supplement: Supplementary file 9 — Additional file 9. Water bath estimation IR images. File containing the thermal imaging (and paired photographs) of all images used in data collection for the water bath protocol. Images are sorted by species, set temperature of the water bath and then by individual flower, flower file names are formatted as [flower identifier used for sorting e.g. ‘D’][number]. Additionally, where available, photos are provided to aid identification of individual flowers. [file 13007_2021_721_MOESM9_ESM.zip › Bath IR images/Bellis/35c/B3/IR_89422.jpg]

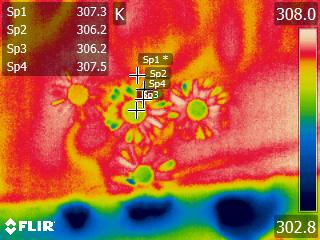

Supplement: Supplementary file 9 — Additional file 9. Water bath estimation IR images. File containing the thermal imaging (and paired photographs) of all images used in data collection for the water bath protocol. Images are sorted by species, set temperature of the water bath and then by individual flower, flower file names are formatted as [flower identifier used for sorting e.g. ‘D’][number]. Additionally, where available, photos are provided to aid identification of individual flowers. [file 13007_2021_721_MOESM9_ESM.zip › Bath IR images/Bellis/35c/B4/IR_89422.jpg]

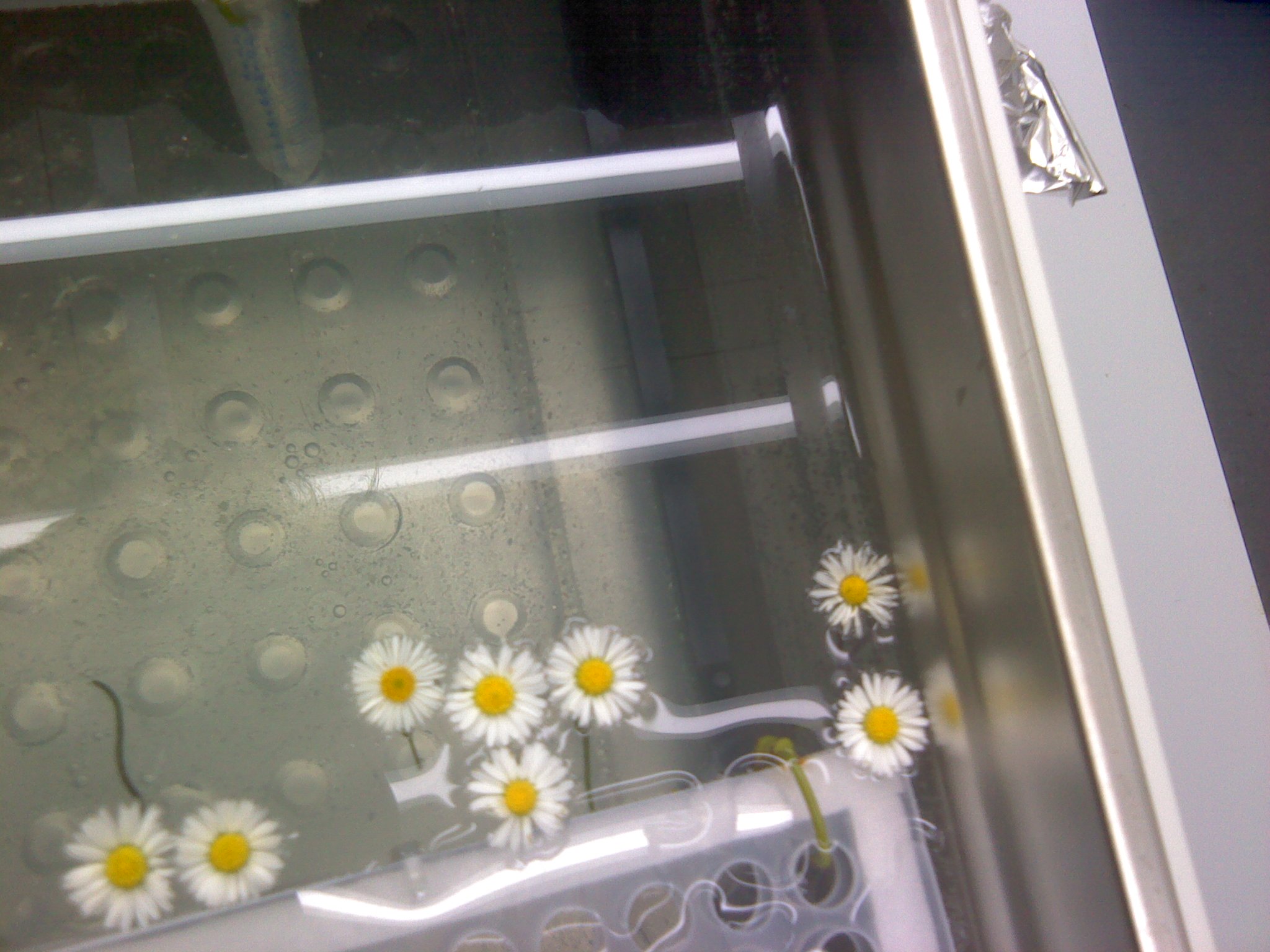

Supplement: Supplementary file 9 — Additional file 9. Water bath estimation IR images. File containing the thermal imaging (and paired photographs) of all images used in data collection for the water bath protocol. Images are sorted by species, set temperature of the water bath and then by individual flower, flower file names are formatted as [flower identifier used for sorting e.g. ‘D’][number]. Additionally, where available, photos are provided to aid identification of individual flowers. [file 13007_2021_721_MOESM9_ESM.zip › Bath IR images/Bellis/35c/B5/DC_89429.jpg]

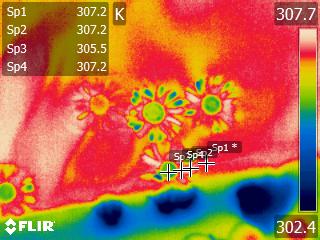

Supplement: Supplementary file 9 — Additional file 9. Water bath estimation IR images. File containing the thermal imaging (and paired photographs) of all images used in data collection for the water bath protocol. Images are sorted by species, set temperature of the water bath and then by individual flower, flower file names are formatted as [flower identifier used for sorting e.g. ‘D’][number]. Additionally, where available, photos are provided to aid identification of individual flowers. [file 13007_2021_721_MOESM9_ESM.zip › Bath IR images/Bellis/35c/B5/IR_89428.jpg]

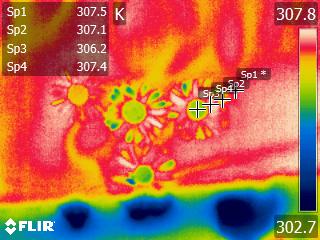

Supplement: Supplementary file 9 — Additional file 9. Water bath estimation IR images. File containing the thermal imaging (and paired photographs) of all images used in data collection for the water bath protocol. Images are sorted by species, set temperature of the water bath and then by individual flower, flower file names are formatted as [flower identifier used for sorting e.g. ‘D’][number]. Additionally, where available, photos are provided to aid identification of individual flowers. [file 13007_2021_721_MOESM9_ESM.zip › Bath IR images/Bellis/35c/B6/IR_89422.jpg]

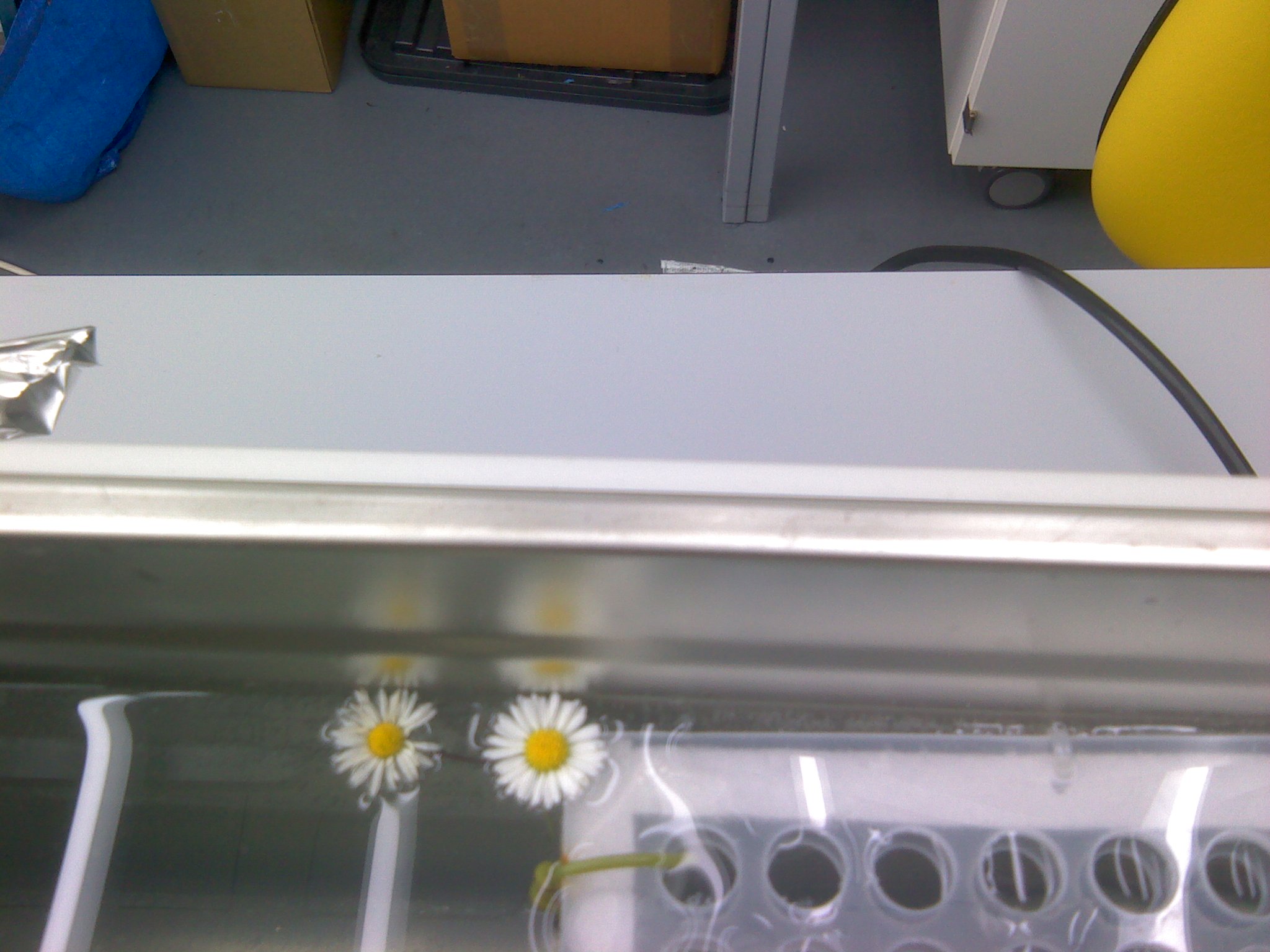

Supplement: Supplementary file 9 — Additional file 9. Water bath estimation IR images. File containing the thermal imaging (and paired photographs) of all images used in data collection for the water bath protocol. Images are sorted by species, set temperature of the water bath and then by individual flower, flower file names are formatted as [flower identifier used for sorting e.g. ‘D’][number]. Additionally, where available, photos are provided to aid identification of individual flowers. [file 13007_2021_721_MOESM9_ESM.zip › Bath IR images/Bellis/35c/B7/DC_89425.jpg]

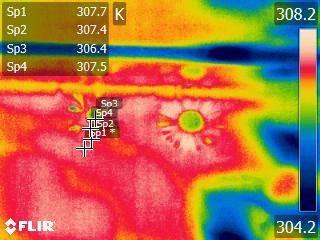

Supplement: Supplementary file 9 — Additional file 9. Water bath estimation IR images. File containing the thermal imaging (and paired photographs) of all images used in data collection for the water bath protocol. Images are sorted by species, set temperature of the water bath and then by individual flower, flower file names are formatted as [flower identifier used for sorting e.g. ‘D’][number]. Additionally, where available, photos are provided to aid identification of individual flowers. [file 13007_2021_721_MOESM9_ESM.zip › Bath IR images/Bellis/35c/B7/IR_89424.jpg]

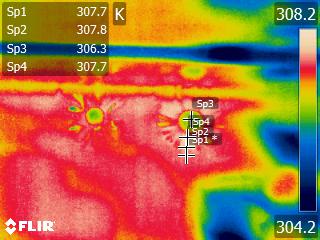

Supplement: Supplementary file 9 — Additional file 9. Water bath estimation IR images. File containing the thermal imaging (and paired photographs) of all images used in data collection for the water bath protocol. Images are sorted by species, set temperature of the water bath and then by individual flower, flower file names are formatted as [flower identifier used for sorting e.g. ‘D’][number]. Additionally, where available, photos are provided to aid identification of individual flowers. [file 13007_2021_721_MOESM9_ESM.zip › Bath IR images/Bellis/35c/B8/IR_89424.jpg]

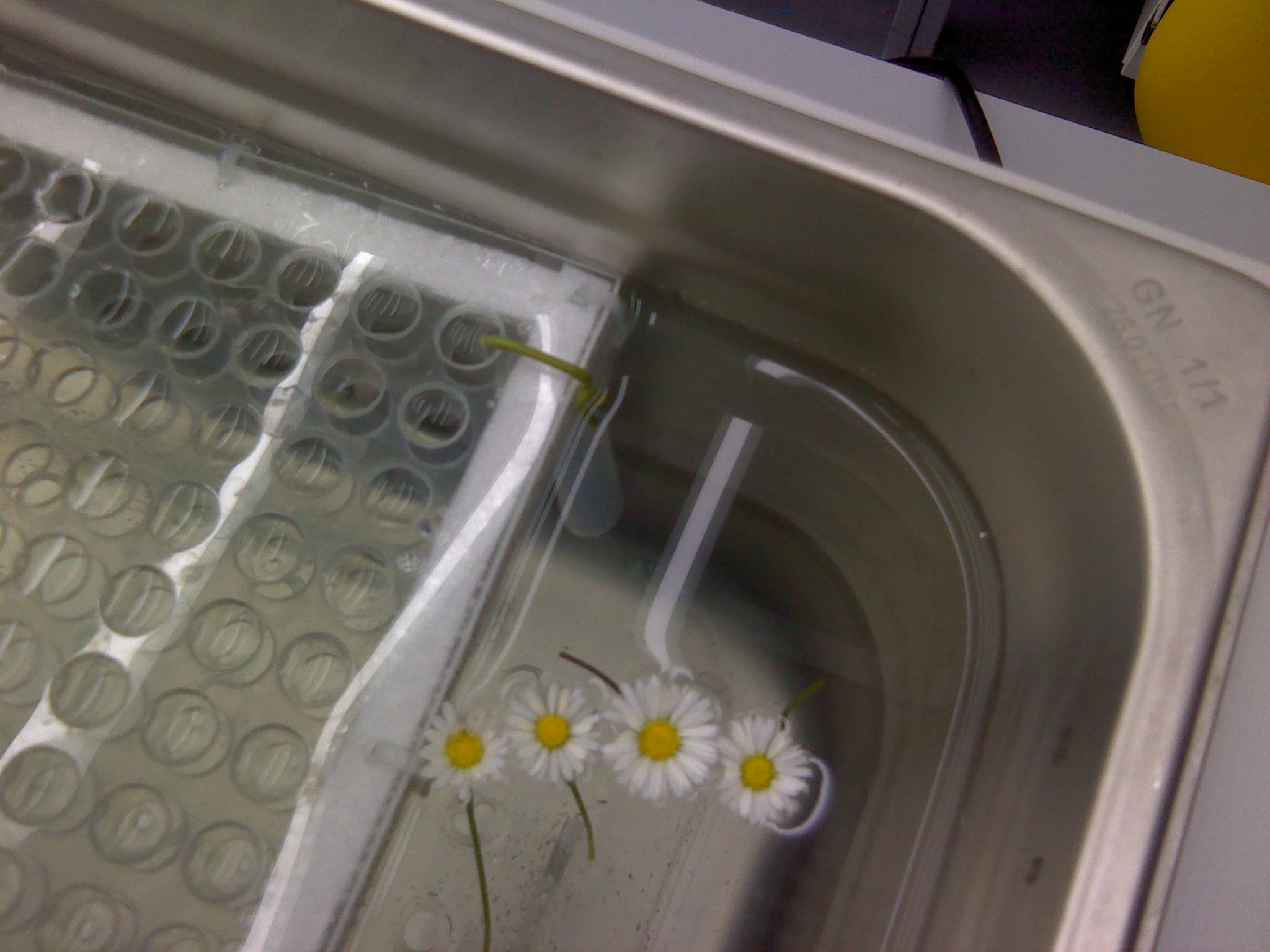

Supplement: Supplementary file 9 — Additional file 9. Water bath estimation IR images. File containing the thermal imaging (and paired photographs) of all images used in data collection for the water bath protocol. Images are sorted by species, set temperature of the water bath and then by individual flower, flower file names are formatted as [flower identifier used for sorting e.g. ‘D’][number]. Additionally, where available, photos are provided to aid identification of individual flowers. [file 13007_2021_721_MOESM9_ESM.zip › Bath IR images/Bellis/35c/B9/DC_89477.jpg]

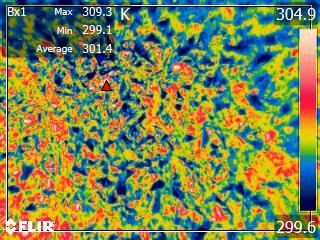

Supplement: Supplementary file 9 — Additional file 9. Water bath estimation IR images. File containing the thermal imaging (and paired photographs) of all images used in data collection for the water bath protocol. Images are sorted by species, set temperature of the water bath and then by individual flower, flower file names are formatted as [flower identifier used for sorting e.g. ‘D’][number]. Additionally, where available, photos are provided to aid identification of individual flowers. [file 13007_2021_721_MOESM9_ESM.zip › Bath IR images/Bellis/35c/B9/IR_89474.jpg]

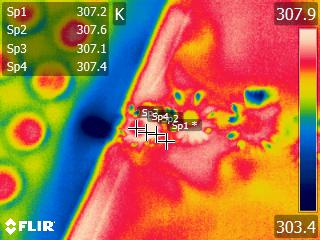

Supplement: Supplementary file 9 — Additional file 9. Water bath estimation IR images. File containing the thermal imaging (and paired photographs) of all images used in data collection for the water bath protocol. Images are sorted by species, set temperature of the water bath and then by individual flower, flower file names are formatted as [flower identifier used for sorting e.g. ‘D’][number]. Additionally, where available, photos are provided to aid identification of individual flowers. [file 13007_2021_721_MOESM9_ESM.zip › Bath IR images/Bellis/35c/B9/IR_89476.jpg]

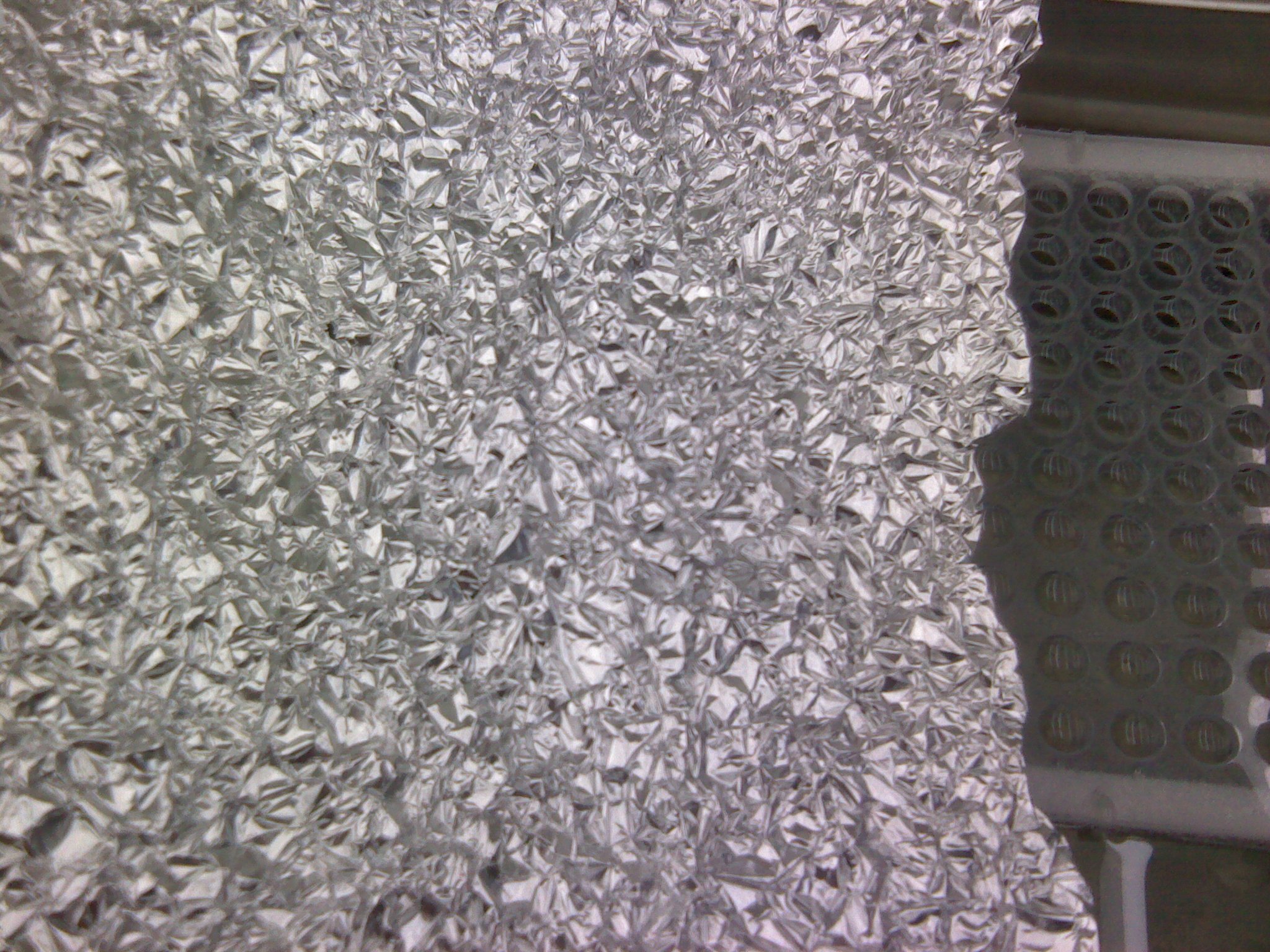

Supplement: Supplementary file 9 — Additional file 9. Water bath estimation IR images. File containing the thermal imaging (and paired photographs) of all images used in data collection for the water bath protocol. Images are sorted by species, set temperature of the water bath and then by individual flower, flower file names are formatted as [flower identifier used for sorting e.g. ‘D’][number]. Additionally, where available, photos are provided to aid identification of individual flowers. [file 13007_2021_721_MOESM9_ESM.zip › Bath IR images/Bellis/45c/B1/DC_89459.jpg]

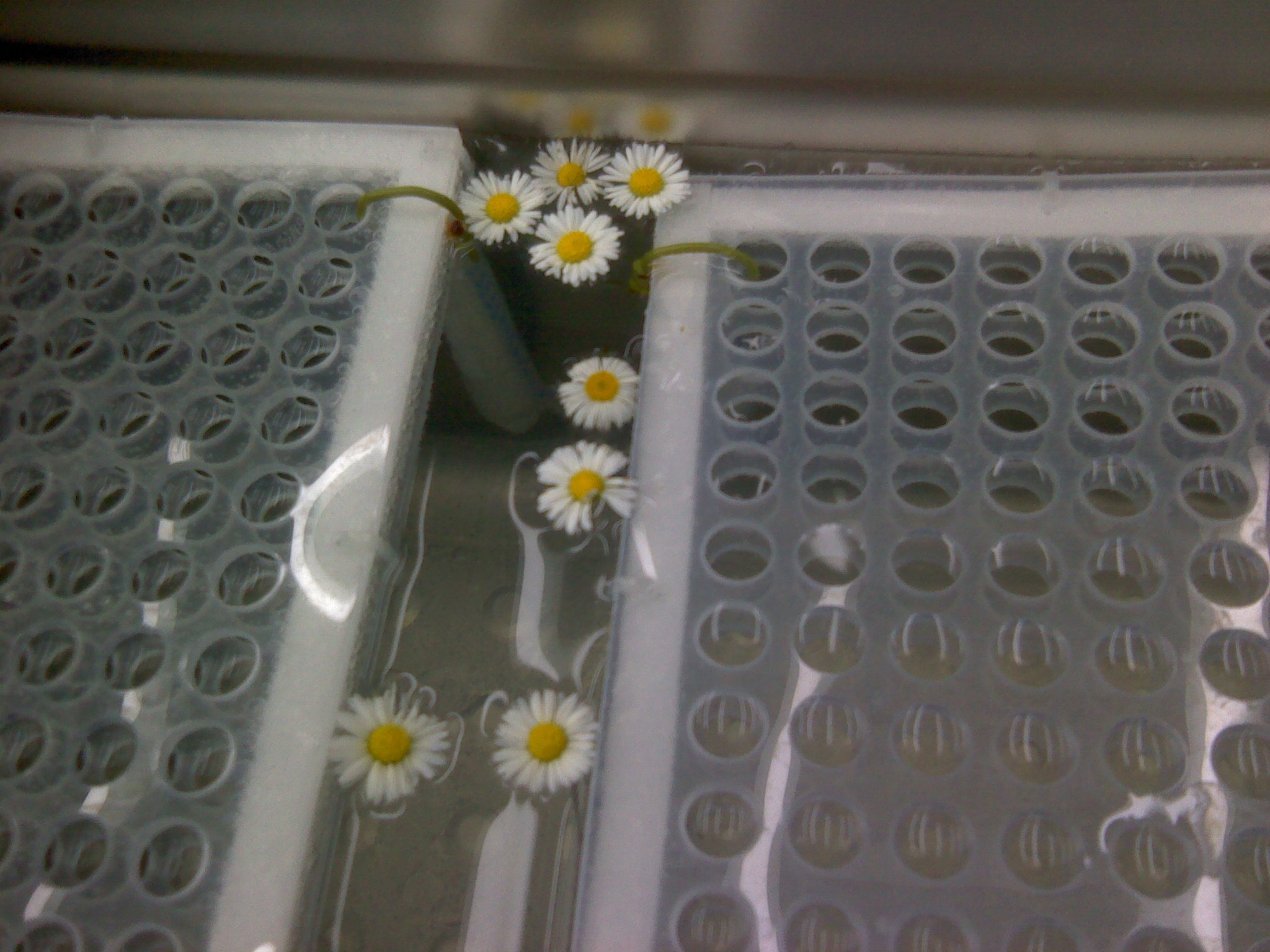

Supplement: Supplementary file 9 — Additional file 9. Water bath estimation IR images. File containing the thermal imaging (and paired photographs) of all images used in data collection for the water bath protocol. Images are sorted by species, set temperature of the water bath and then by individual flower, flower file names are formatted as [flower identifier used for sorting e.g. ‘D’][number]. Additionally, where available, photos are provided to aid identification of individual flowers. [file 13007_2021_721_MOESM9_ESM.zip › Bath IR images/Bellis/45c/B1/DC_89465.jpg]

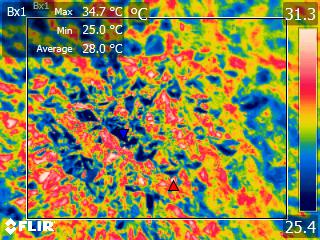

Supplement: Supplementary file 9 — Additional file 9. Water bath estimation IR images. File containing the thermal imaging (and paired photographs) of all images used in data collection for the water bath protocol. Images are sorted by species, set temperature of the water bath and then by individual flower, flower file names are formatted as [flower identifier used for sorting e.g. ‘D’][number]. Additionally, where available, photos are provided to aid identification of individual flowers. [file 13007_2021_721_MOESM9_ESM.zip › Bath IR images/Bellis/45c/B1/IR_89458.jpg]

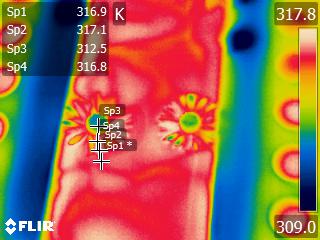

Supplement: Supplementary file 9 — Additional file 9. Water bath estimation IR images. File containing the thermal imaging (and paired photographs) of all images used in data collection for the water bath protocol. Images are sorted by species, set temperature of the water bath and then by individual flower, flower file names are formatted as [flower identifier used for sorting e.g. ‘D’][number]. Additionally, where available, photos are provided to aid identification of individual flowers. [file 13007_2021_721_MOESM9_ESM.zip › Bath IR images/Bellis/45c/B1/IR_89464.jpg]

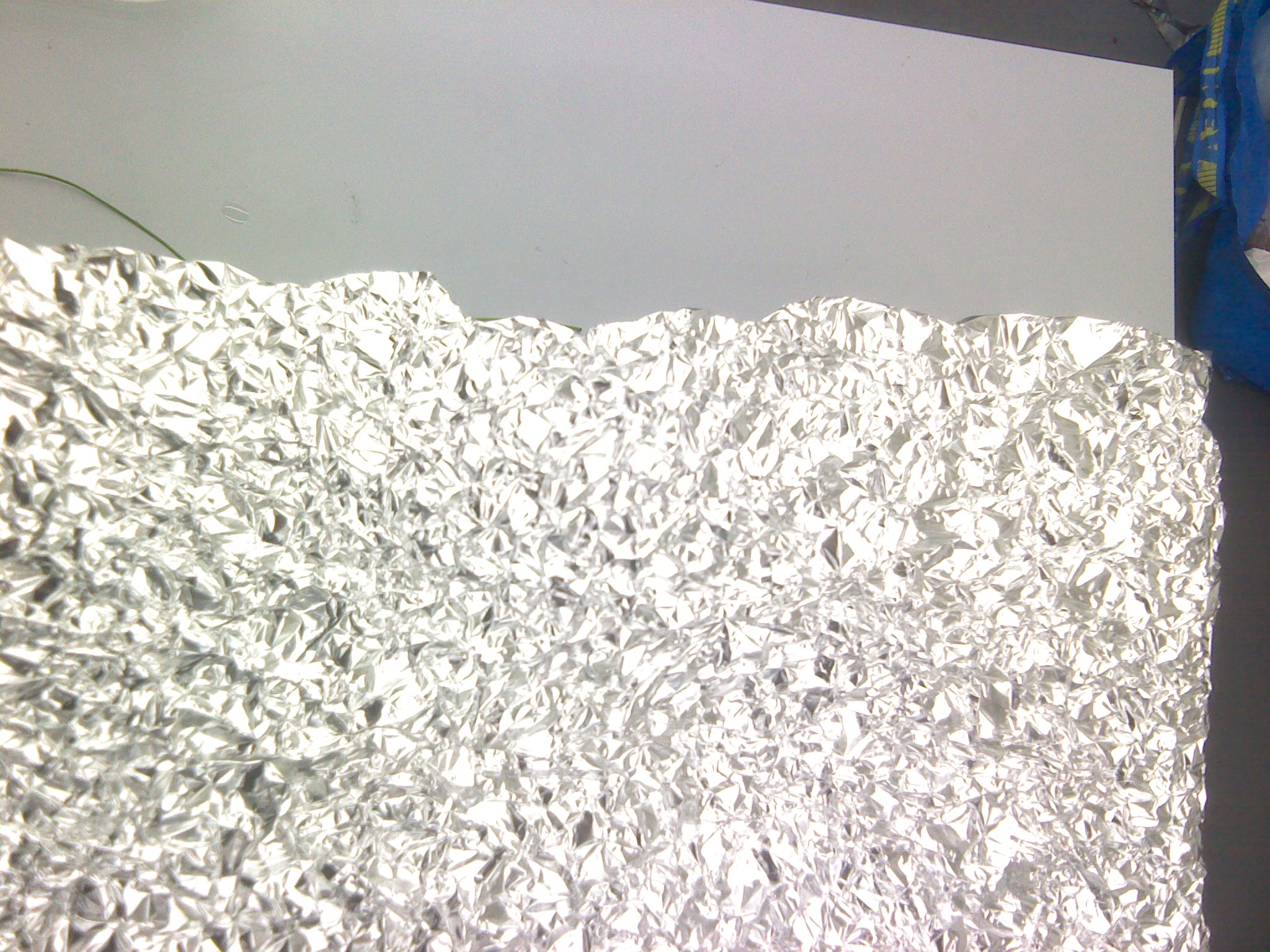

Supplement: Supplementary file 9 — Additional file 9. Water bath estimation IR images. File containing the thermal imaging (and paired photographs) of all images used in data collection for the water bath protocol. Images are sorted by species, set temperature of the water bath and then by individual flower, flower file names are formatted as [flower identifier used for sorting e.g. ‘D’][number]. Additionally, where available, photos are provided to aid identification of individual flowers. [file 13007_2021_721_MOESM9_ESM.zip › Bath IR images/Bellis/45c/B10/DC_89547.jpg]

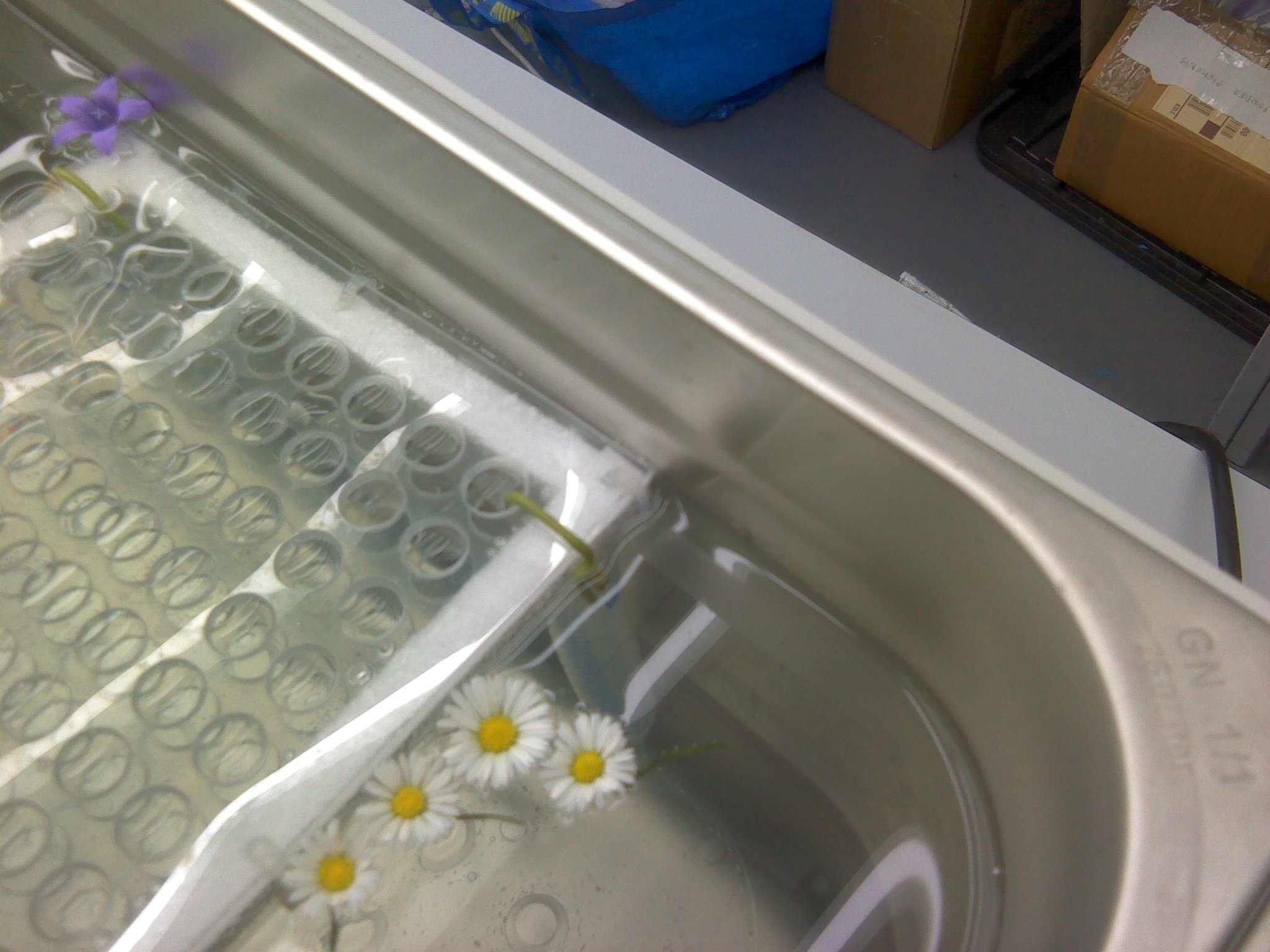

Supplement: Supplementary file 9 — Additional file 9. Water bath estimation IR images. File containing the thermal imaging (and paired photographs) of all images used in data collection for the water bath protocol. Images are sorted by species, set temperature of the water bath and then by individual flower, flower file names are formatted as [flower identifier used for sorting e.g. ‘D’][number]. Additionally, where available, photos are provided to aid identification of individual flowers. [file 13007_2021_721_MOESM9_ESM.zip › Bath IR images/Bellis/45c/B10/DC_89553.jpg]

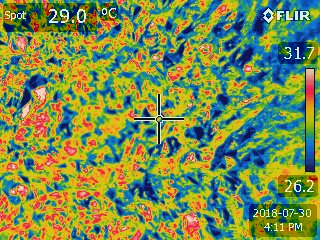

Supplement: Supplementary file 9 — Additional file 9. Water bath estimation IR images. File containing the thermal imaging (and paired photographs) of all images used in data collection for the water bath protocol. Images are sorted by species, set temperature of the water bath and then by individual flower, flower file names are formatted as [flower identifier used for sorting e.g. ‘D’][number]. Additionally, where available, photos are provided to aid identification of individual flowers. [file 13007_2021_721_MOESM9_ESM.zip › Bath IR images/Bellis/45c/B10/IR_89546.jpg]

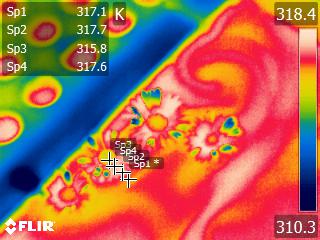

Supplement: Supplementary file 9 — Additional file 9. Water bath estimation IR images. File containing the thermal imaging (and paired photographs) of all images used in data collection for the water bath protocol. Images are sorted by species, set temperature of the water bath and then by individual flower, flower file names are formatted as [flower identifier used for sorting e.g. ‘D’][number]. Additionally, where available, photos are provided to aid identification of individual flowers. [file 13007_2021_721_MOESM9_ESM.zip › Bath IR images/Bellis/45c/B10/IR_89552.jpg]

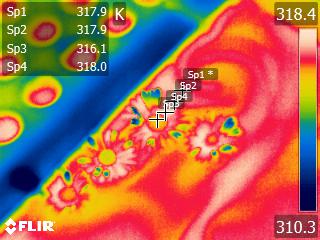

Supplement: Supplementary file 9 — Additional file 9. Water bath estimation IR images. File containing the thermal imaging (and paired photographs) of all images used in data collection for the water bath protocol. Images are sorted by species, set temperature of the water bath and then by individual flower, flower file names are formatted as [flower identifier used for sorting e.g. ‘D’][number]. Additionally, where available, photos are provided to aid identification of individual flowers. [file 13007_2021_721_MOESM9_ESM.zip › Bath IR images/Bellis/45c/B11/IR_89552.jpg]

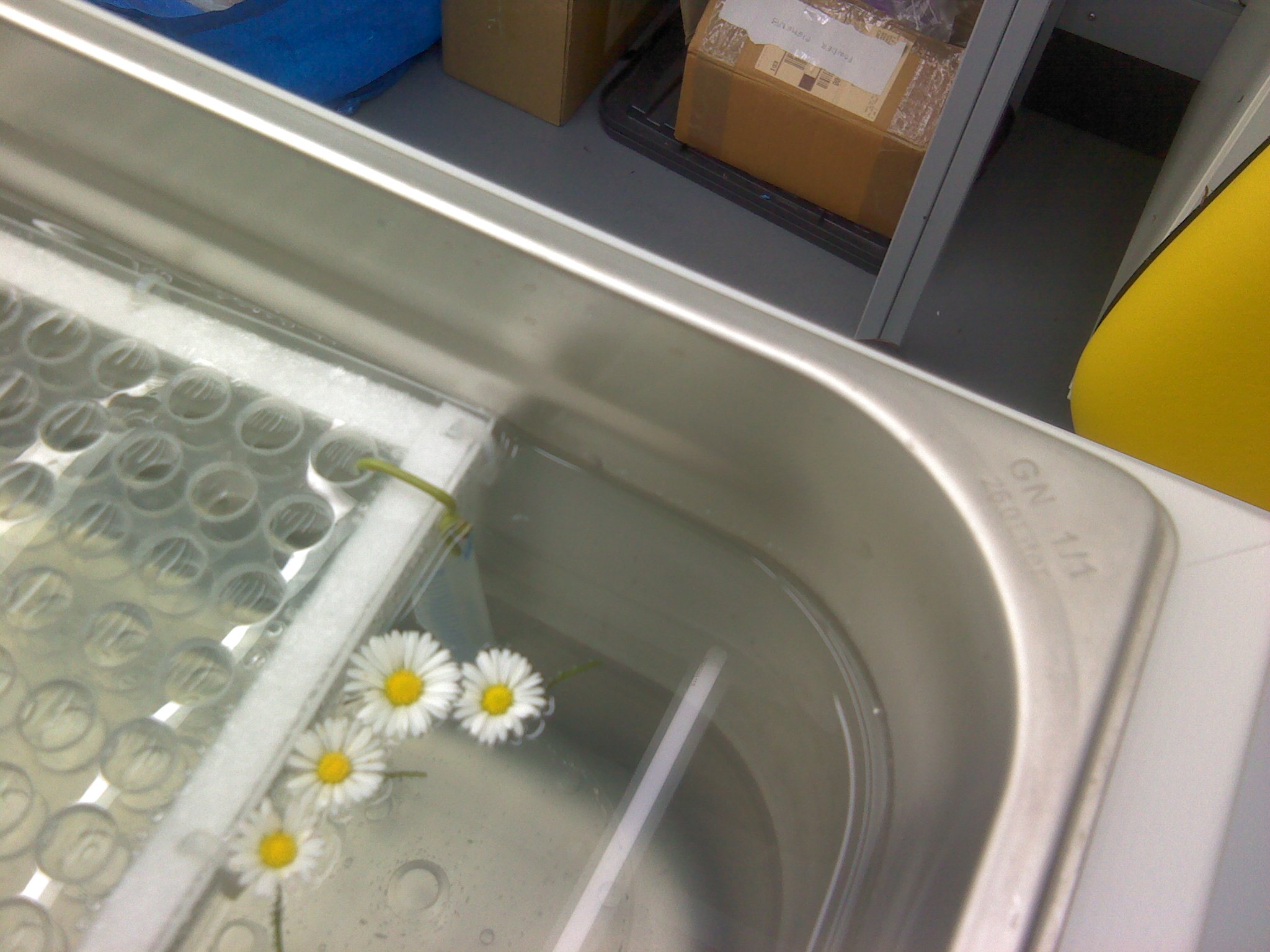

Supplement: Supplementary file 9 — Additional file 9. Water bath estimation IR images. File containing the thermal imaging (and paired photographs) of all images used in data collection for the water bath protocol. Images are sorted by species, set temperature of the water bath and then by individual flower, flower file names are formatted as [flower identifier used for sorting e.g. ‘D’][number]. Additionally, where available, photos are provided to aid identification of individual flowers. [file 13007_2021_721_MOESM9_ESM.zip › Bath IR images/Bellis/45c/B12/DC_89555.jpg]

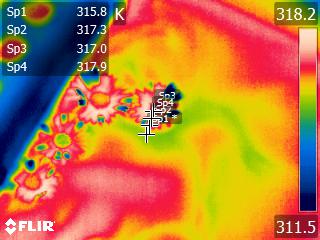

Supplement: Supplementary file 9 — Additional file 9. Water bath estimation IR images. File containing the thermal imaging (and paired photographs) of all images used in data collection for the water bath protocol. Images are sorted by species, set temperature of the water bath and then by individual flower, flower file names are formatted as [flower identifier used for sorting e.g. ‘D’][number]. Additionally, where available, photos are provided to aid identification of individual flowers. [file 13007_2021_721_MOESM9_ESM.zip › Bath IR images/Bellis/45c/B12/IR_89554.jpg]

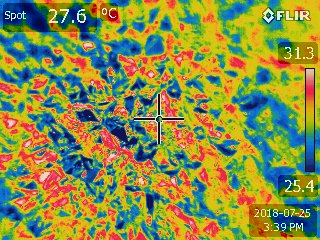

Supplement: Supplementary file 9 — Additional file 9. Water bath estimation IR images. File containing the thermal imaging (and paired photographs) of all images used in data collection for the water bath protocol. Images are sorted by species, set temperature of the water bath and then by individual flower, flower file names are formatted as [flower identifier used for sorting e.g. ‘D’][number]. Additionally, where available, photos are provided to aid identification of individual flowers. [file 13007_2021_721_MOESM9_ESM.zip › Bath IR images/Bellis/45c/B2/IR_89458.jpg]

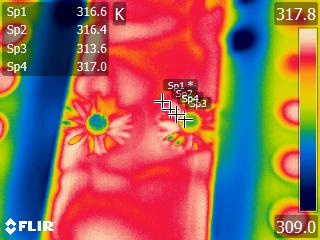

Supplement: Supplementary file 9 — Additional file 9. Water bath estimation IR images. File containing the thermal imaging (and paired photographs) of all images used in data collection for the water bath protocol. Images are sorted by species, set temperature of the water bath and then by individual flower, flower file names are formatted as [flower identifier used for sorting e.g. ‘D’][number]. Additionally, where available, photos are provided to aid identification of individual flowers. [file 13007_2021_721_MOESM9_ESM.zip › Bath IR images/Bellis/45c/B2/IR_89464.jpg]

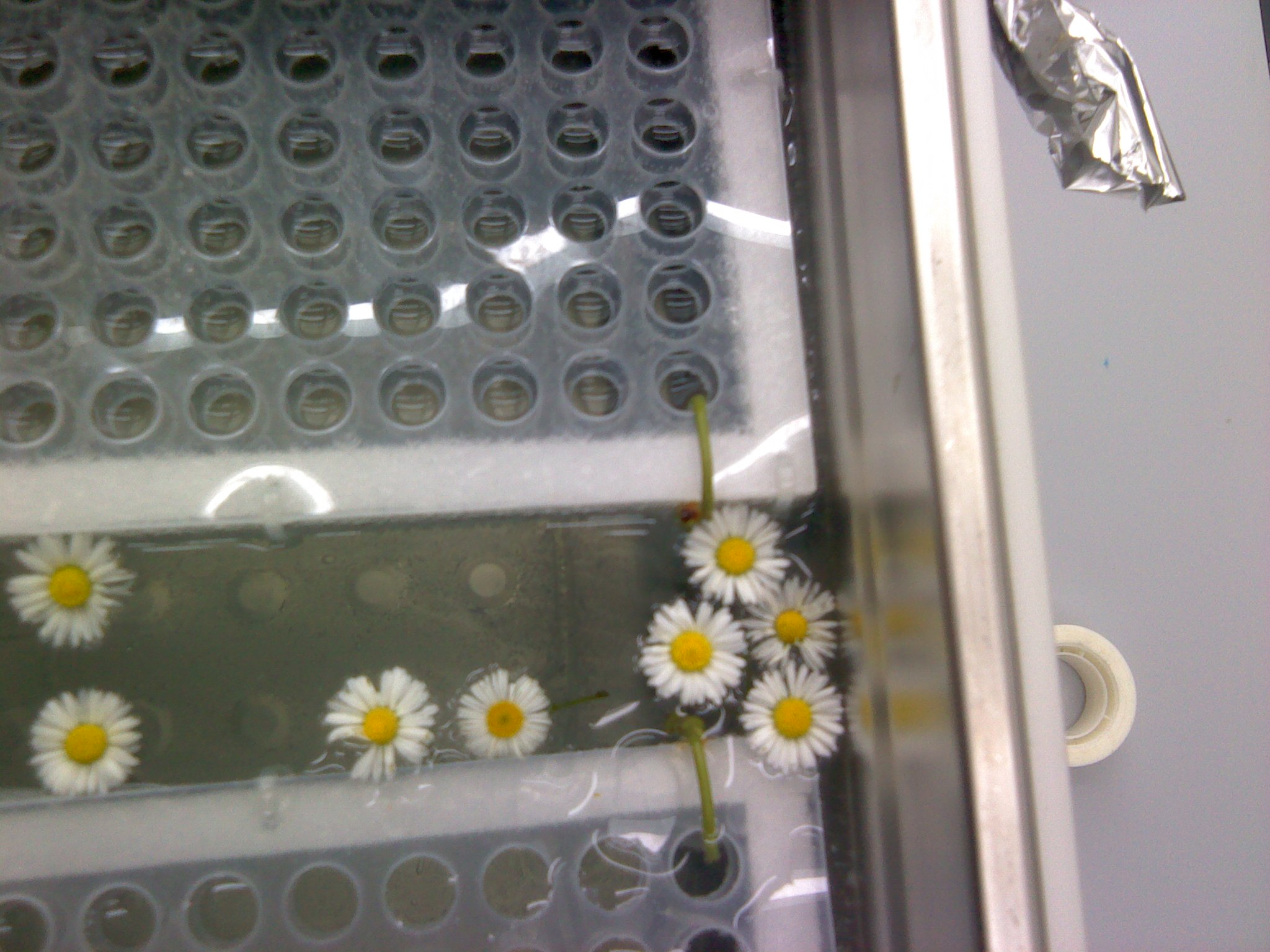

Supplement: Supplementary file 9 — Additional file 9. Water bath estimation IR images. File containing the thermal imaging (and paired photographs) of all images used in data collection for the water bath protocol. Images are sorted by species, set temperature of the water bath and then by individual flower, flower file names are formatted as [flower identifier used for sorting e.g. ‘D’][number]. Additionally, where available, photos are provided to aid identification of individual flowers. [file 13007_2021_721_MOESM9_ESM.zip › Bath IR images/Bellis/45c/B3/DC_89469.jpg]

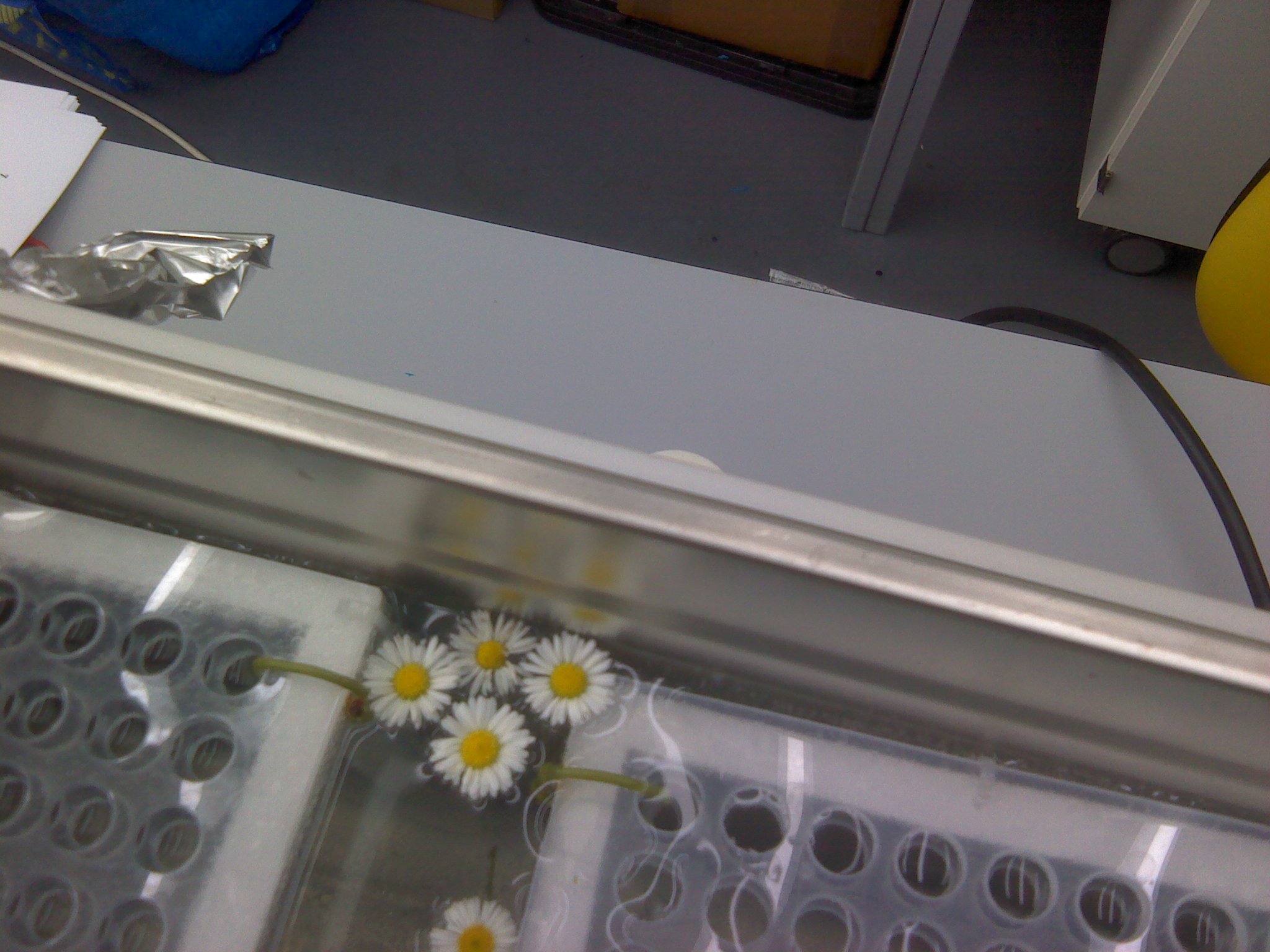

Supplement: Supplementary file 9 — Additional file 9. Water bath estimation IR images. File containing the thermal imaging (and paired photographs) of all images used in data collection for the water bath protocol. Images are sorted by species, set temperature of the water bath and then by individual flower, flower file names are formatted as [flower identifier used for sorting e.g. ‘D’][number]. Additionally, where available, photos are provided to aid identification of individual flowers. [file 13007_2021_721_MOESM9_ESM.zip › Bath IR images/Bellis/45c/B3/DC_89473.jpg]

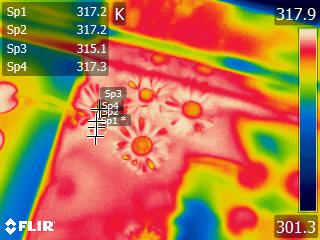

Supplement: Supplementary file 9 — Additional file 9. Water bath estimation IR images. File containing the thermal imaging (and paired photographs) of all images used in data collection for the water bath protocol. Images are sorted by species, set temperature of the water bath and then by individual flower, flower file names are formatted as [flower identifier used for sorting e.g. ‘D’][number]. Additionally, where available, photos are provided to aid identification of individual flowers. [file 13007_2021_721_MOESM9_ESM.zip › Bath IR images/Bellis/45c/B3/IR_89472.jpg]

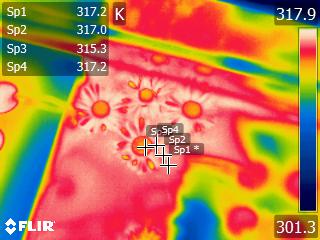

Supplement: Supplementary file 9 — Additional file 9. Water bath estimation IR images. File containing the thermal imaging (and paired photographs) of all images used in data collection for the water bath protocol. Images are sorted by species, set temperature of the water bath and then by individual flower, flower file names are formatted as [flower identifier used for sorting e.g. ‘D’][number]. Additionally, where available, photos are provided to aid identification of individual flowers. [file 13007_2021_721_MOESM9_ESM.zip › Bath IR images/Bellis/45c/B4/IR_89472.jpg]

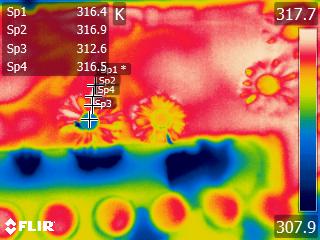

Supplement: Supplementary file 9 — Additional file 9. Water bath estimation IR images. File containing the thermal imaging (and paired photographs) of all images used in data collection for the water bath protocol. Images are sorted by species, set temperature of the water bath and then by individual flower, flower file names are formatted as [flower identifier used for sorting e.g. ‘D’][number]. Additionally, where available, photos are provided to aid identification of individual flowers. [file 13007_2021_721_MOESM9_ESM.zip › Bath IR images/Bellis/45c/B5/IR_89468.jpg]

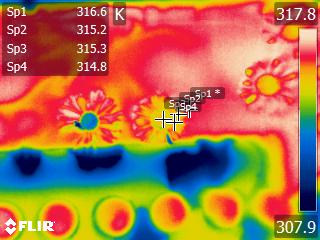

Supplement: Supplementary file 9 — Additional file 9. Water bath estimation IR images. File containing the thermal imaging (and paired photographs) of all images used in data collection for the water bath protocol. Images are sorted by species, set temperature of the water bath and then by individual flower, flower file names are formatted as [flower identifier used for sorting e.g. ‘D’][number]. Additionally, where available, photos are provided to aid identification of individual flowers. [file 13007_2021_721_MOESM9_ESM.zip › Bath IR images/Bellis/45c/B6/IR_89468.jpg]

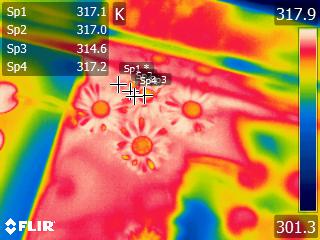

Supplement: Supplementary file 9 — Additional file 9. Water bath estimation IR images. File containing the thermal imaging (and paired photographs) of all images used in data collection for the water bath protocol. Images are sorted by species, set temperature of the water bath and then by individual flower, flower file names are formatted as [flower identifier used for sorting e.g. ‘D’][number]. Additionally, where available, photos are provided to aid identification of individual flowers. [file 13007_2021_721_MOESM9_ESM.zip › Bath IR images/Bellis/45c/B7/IR_89472.jpg]

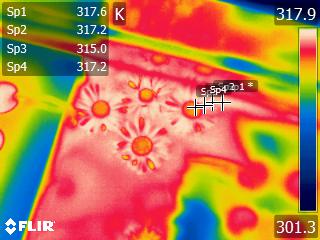

Supplement: Supplementary file 9 — Additional file 9. Water bath estimation IR images. File containing the thermal imaging (and paired photographs) of all images used in data collection for the water bath protocol. Images are sorted by species, set temperature of the water bath and then by individual flower, flower file names are formatted as [flower identifier used for sorting e.g. ‘D’][number]. Additionally, where available, photos are provided to aid identification of individual flowers. [file 13007_2021_721_MOESM9_ESM.zip › Bath IR images/Bellis/45c/B8/IR_89472.jpg]

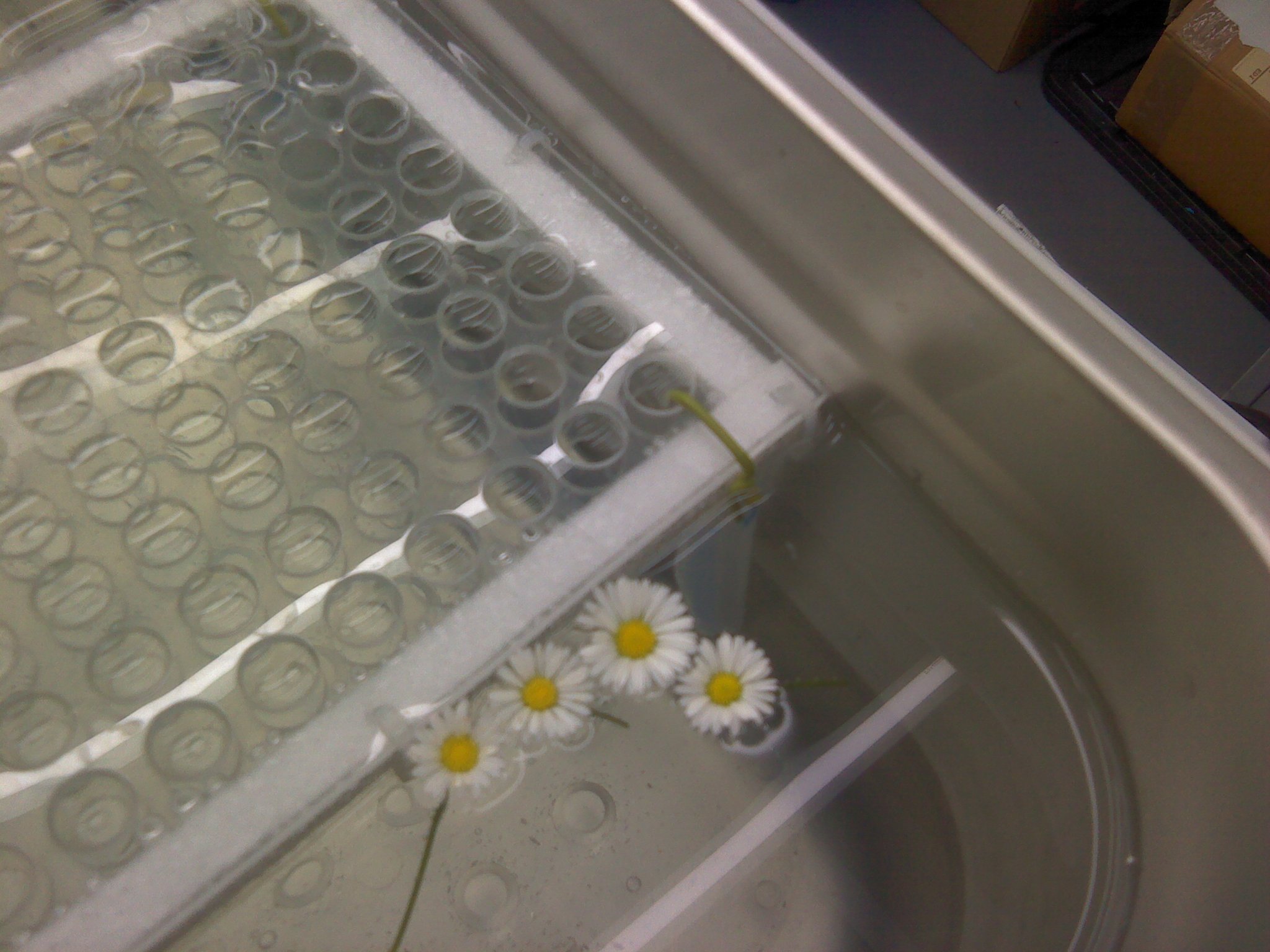

Supplement: Supplementary file 9 — Additional file 9. Water bath estimation IR images. File containing the thermal imaging (and paired photographs) of all images used in data collection for the water bath protocol. Images are sorted by species, set temperature of the water bath and then by individual flower, flower file names are formatted as [flower identifier used for sorting e.g. ‘D’][number]. Additionally, where available, photos are provided to aid identification of individual flowers. [file 13007_2021_721_MOESM9_ESM.zip › Bath IR images/Bellis/45c/B9/DC_89549.jpg]

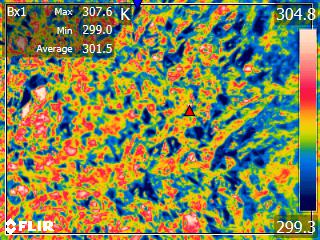

Supplement: Supplementary file 9 — Additional file 9. Water bath estimation IR images. File containing the thermal imaging (and paired photographs) of all images used in data collection for the water bath protocol. Images are sorted by species, set temperature of the water bath and then by individual flower, flower file names are formatted as [flower identifier used for sorting e.g. ‘D’][number]. Additionally, where available, photos are provided to aid identification of individual flowers. [file 13007_2021_721_MOESM9_ESM.zip › Bath IR images/Bellis/45c/B9/IR_89546.jpg]

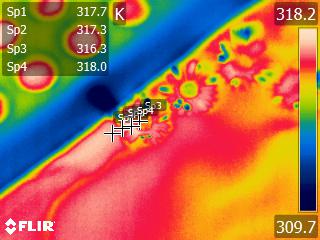

Supplement: Supplementary file 9 — Additional file 9. Water bath estimation IR images. File containing the thermal imaging (and paired photographs) of all images used in data collection for the water bath protocol. Images are sorted by species, set temperature of the water bath and then by individual flower, flower file names are formatted as [flower identifier used for sorting e.g. ‘D’][number]. Additionally, where available, photos are provided to aid identification of individual flowers. [file 13007_2021_721_MOESM9_ESM.zip › Bath IR images/Bellis/45c/B9/IR_89548.jpg]

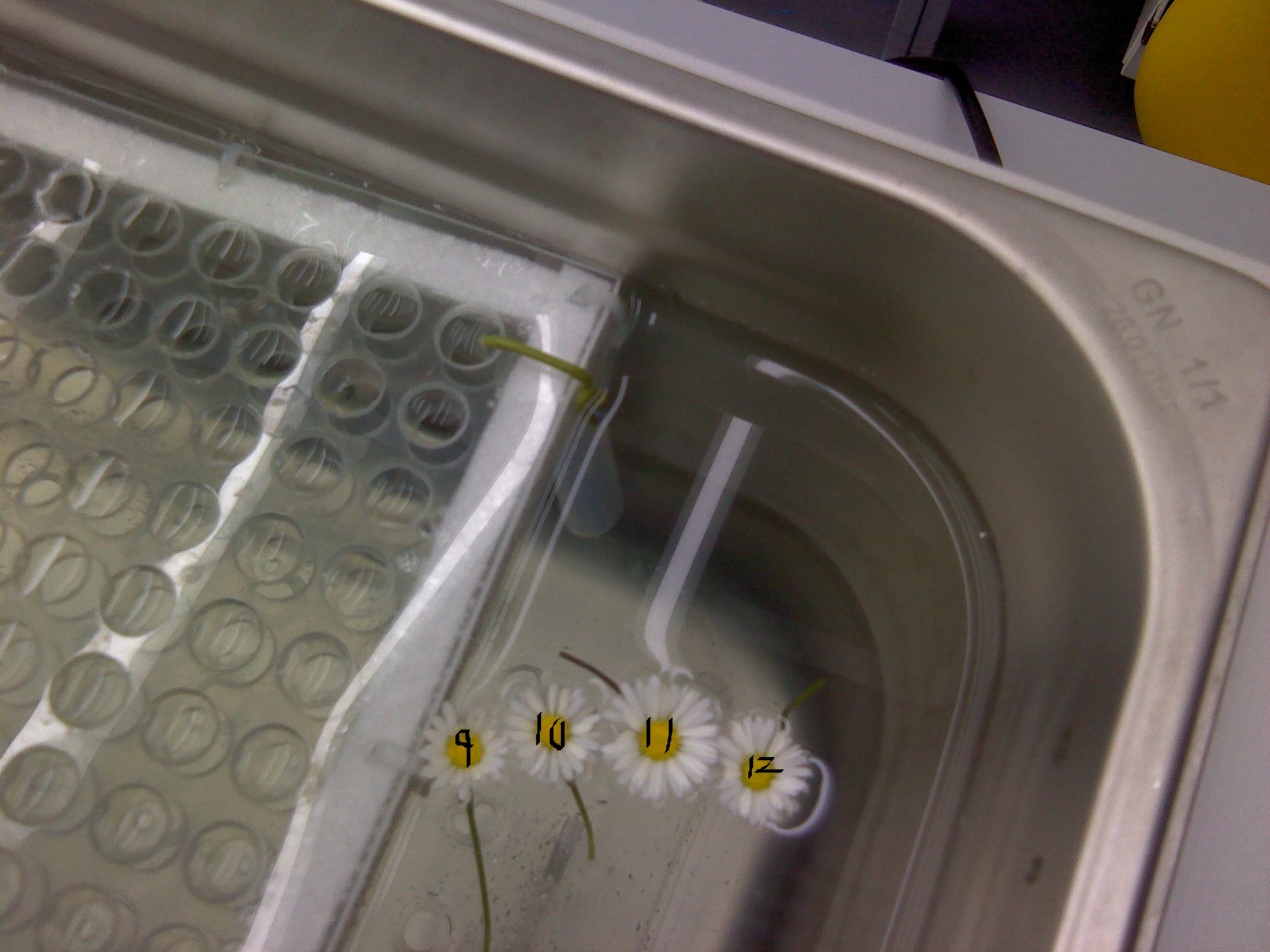

Supplement: Supplementary file 9 — Additional file 9. Water bath estimation IR images. File containing the thermal imaging (and paired photographs) of all images used in data collection for the water bath protocol. Images are sorted by species, set temperature of the water bath and then by individual flower, flower file names are formatted as [flower identifier used for sorting e.g. ‘D’][number]. Additionally, where available, photos are provided to aid identification of individual flowers. [file 13007_2021_721_MOESM9_ESM.zip › Bath IR images/Bellis/9to12.jpg]

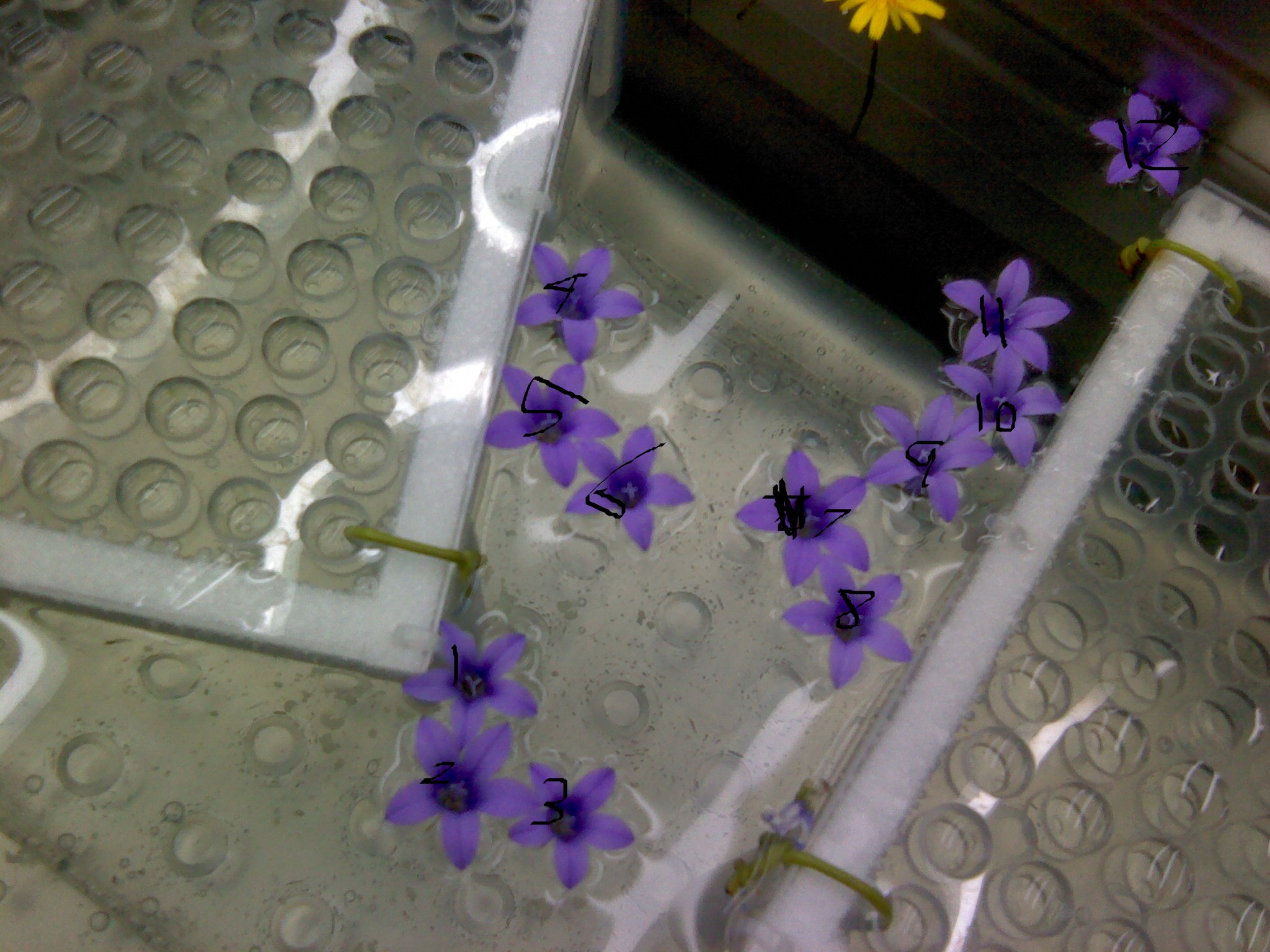

Supplement: Supplementary file 9 — Additional file 9. Water bath estimation IR images. File containing the thermal imaging (and paired photographs) of all images used in data collection for the water bath protocol. Images are sorted by species, set temperature of the water bath and then by individual flower, flower file names are formatted as [flower identifier used for sorting e.g. ‘D’][number]. Additionally, where available, photos are provided to aid identification of individual flowers. [file 13007_2021_721_MOESM9_ESM.zip › Bath IR images/campanula/1tp12.jpg]

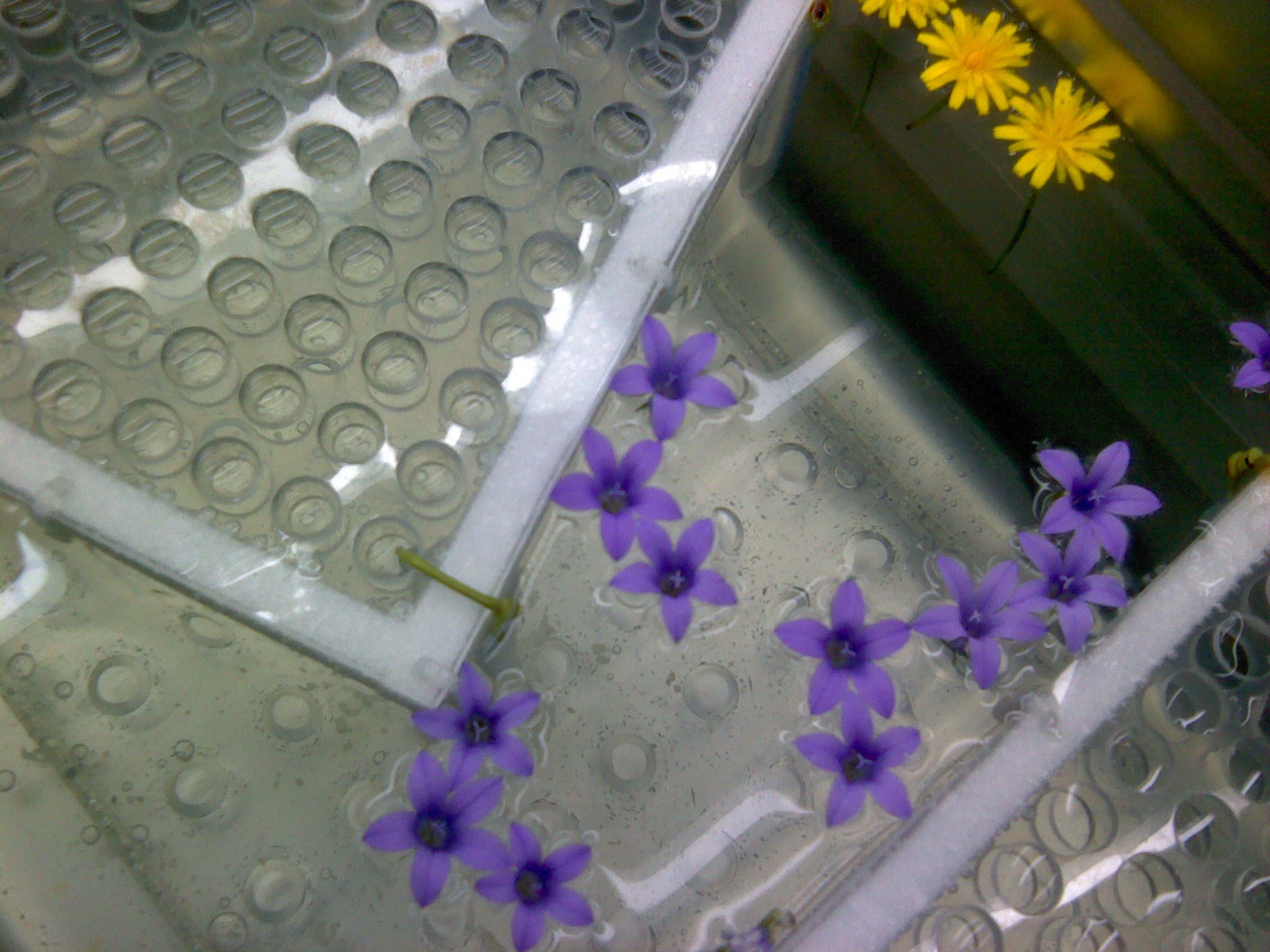

Supplement: Supplementary file 9 — Additional file 9. Water bath estimation IR images. File containing the thermal imaging (and paired photographs) of all images used in data collection for the water bath protocol. Images are sorted by species, set temperature of the water bath and then by individual flower, flower file names are formatted as [flower identifier used for sorting e.g. ‘D’][number]. Additionally, where available, photos are provided to aid identification of individual flowers. [file 13007_2021_721_MOESM9_ESM.zip › Bath IR images/campanula/35c/C1/DC_89497.jpg]

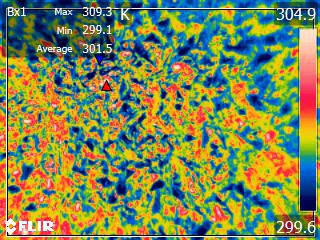

Supplement: Supplementary file 9 — Additional file 9. Water bath estimation IR images. File containing the thermal imaging (and paired photographs) of all images used in data collection for the water bath protocol. Images are sorted by species, set temperature of the water bath and then by individual flower, flower file names are formatted as [flower identifier used for sorting e.g. ‘D’][number]. Additionally, where available, photos are provided to aid identification of individual flowers. [file 13007_2021_721_MOESM9_ESM.zip › Bath IR images/campanula/35c/C1/IR_89474.jpg]

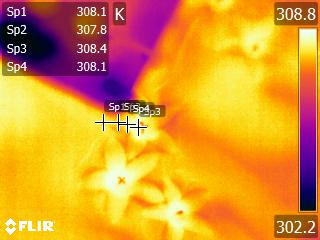

Supplement: Supplementary file 9 — Additional file 9. Water bath estimation IR images. File containing the thermal imaging (and paired photographs) of all images used in data collection for the water bath protocol. Images are sorted by species, set temperature of the water bath and then by individual flower, flower file names are formatted as [flower identifier used for sorting e.g. ‘D’][number]. Additionally, where available, photos are provided to aid identification of individual flowers. [file 13007_2021_721_MOESM9_ESM.zip › Bath IR images/campanula/35c/C1/IR_89496.jpg]

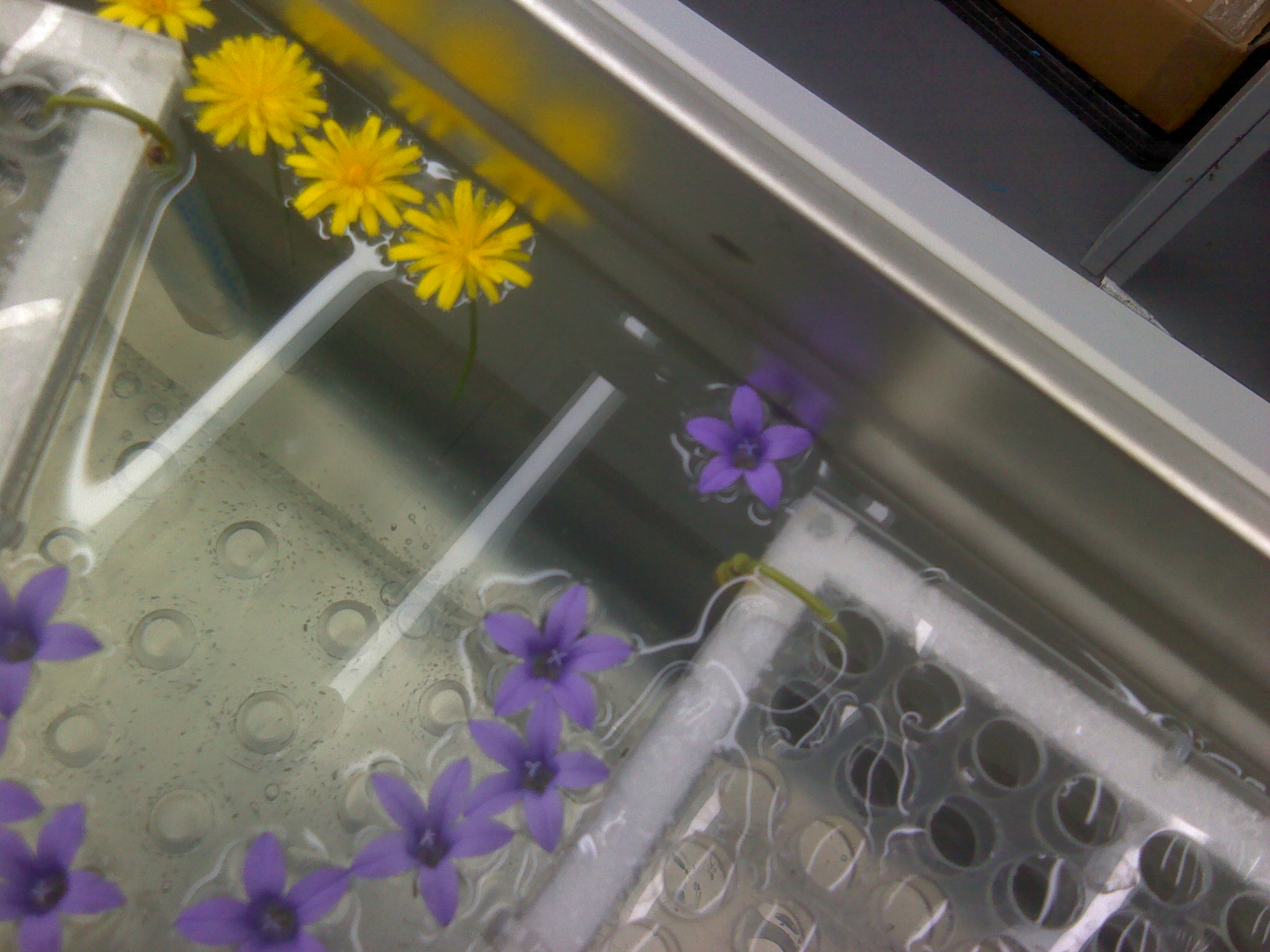

Supplement: Supplementary file 9 — Additional file 9. Water bath estimation IR images. File containing the thermal imaging (and paired photographs) of all images used in data collection for the water bath protocol. Images are sorted by species, set temperature of the water bath and then by individual flower, flower file names are formatted as [flower identifier used for sorting e.g. ‘D’][number]. Additionally, where available, photos are provided to aid identification of individual flowers. [file 13007_2021_721_MOESM9_ESM.zip › Bath IR images/campanula/35c/C10/DC_89515.jpg]

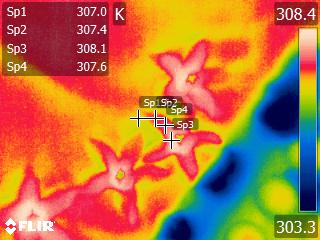

Supplement: Supplementary file 9 — Additional file 9. Water bath estimation IR images. File containing the thermal imaging (and paired photographs) of all images used in data collection for the water bath protocol. Images are sorted by species, set temperature of the water bath and then by individual flower, flower file names are formatted as [flower identifier used for sorting e.g. ‘D’][number]. Additionally, where available, photos are provided to aid identification of individual flowers. [file 13007_2021_721_MOESM9_ESM.zip › Bath IR images/campanula/35c/C10/IR_89514.jpg]

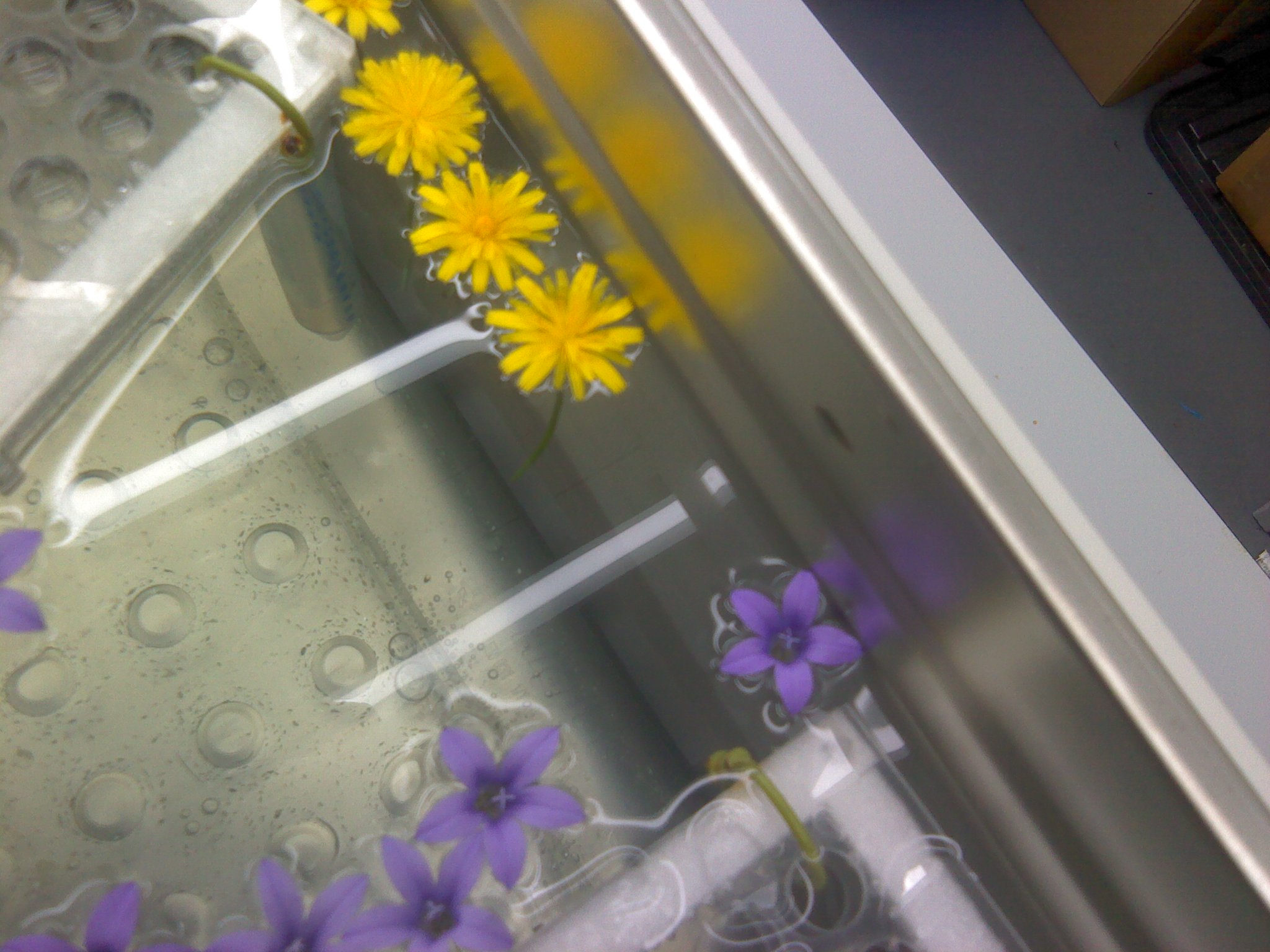

Supplement: Supplementary file 9 — Additional file 9. Water bath estimation IR images. File containing the thermal imaging (and paired photographs) of all images used in data collection for the water bath protocol. Images are sorted by species, set temperature of the water bath and then by individual flower, flower file names are formatted as [flower identifier used for sorting e.g. ‘D’][number]. Additionally, where available, photos are provided to aid identification of individual flowers. [file 13007_2021_721_MOESM9_ESM.zip › Bath IR images/campanula/35c/C11/DC_89519.jpg]

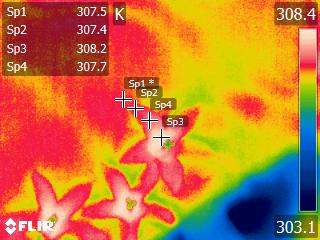

Supplement: Supplementary file 9 — Additional file 9. Water bath estimation IR images. File containing the thermal imaging (and paired photographs) of all images used in data collection for the water bath protocol. Images are sorted by species, set temperature of the water bath and then by individual flower, flower file names are formatted as [flower identifier used for sorting e.g. ‘D’][number]. Additionally, where available, photos are provided to aid identification of individual flowers. [file 13007_2021_721_MOESM9_ESM.zip › Bath IR images/campanula/35c/C11/IR_89518.jpg]

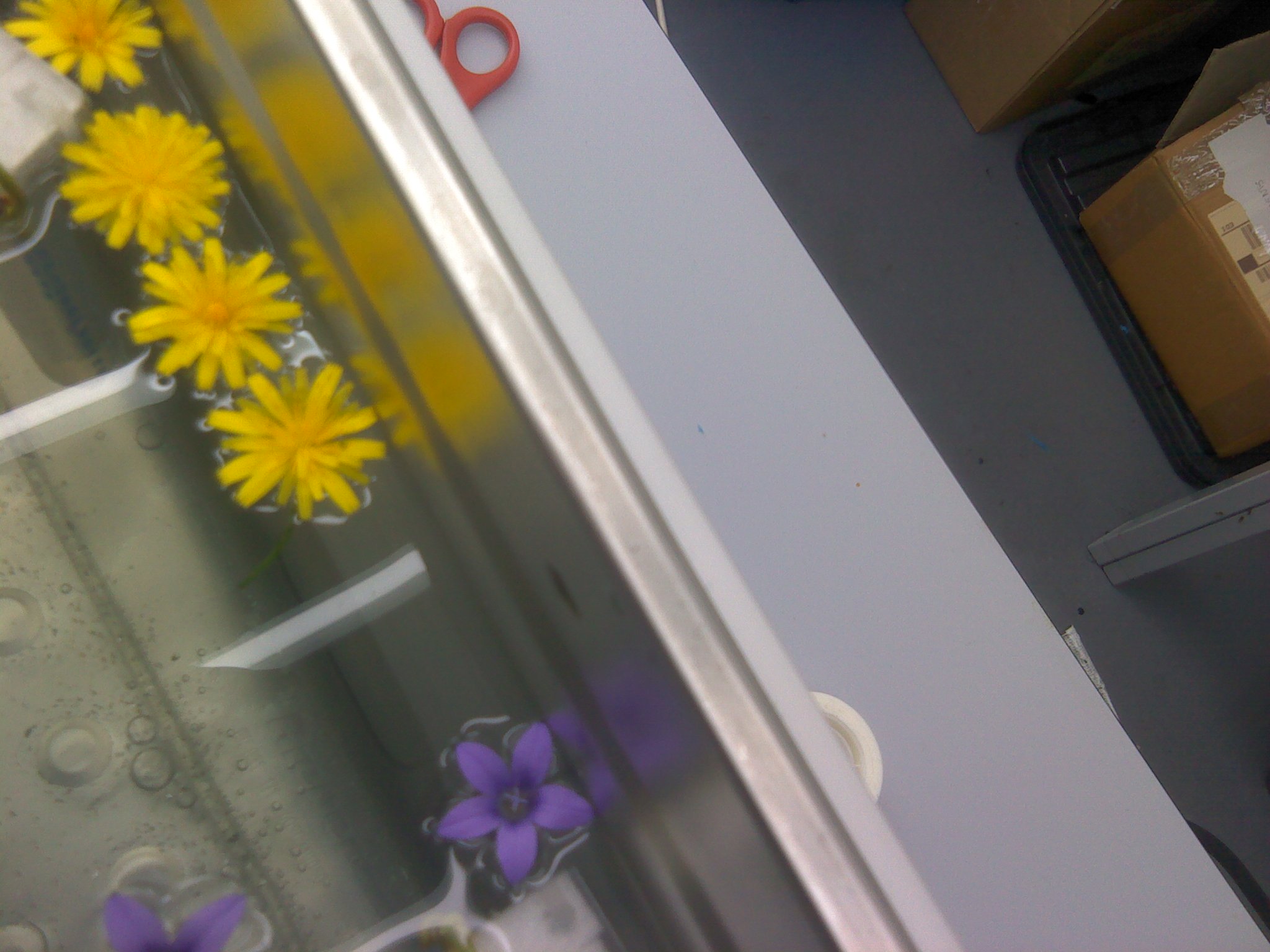

Supplement: Supplementary file 9 — Additional file 9. Water bath estimation IR images. File containing the thermal imaging (and paired photographs) of all images used in data collection for the water bath protocol. Images are sorted by species, set temperature of the water bath and then by individual flower, flower file names are formatted as [flower identifier used for sorting e.g. ‘D’][number]. Additionally, where available, photos are provided to aid identification of individual flowers. [file 13007_2021_721_MOESM9_ESM.zip › Bath IR images/campanula/35c/C12/DC_89521.jpg]

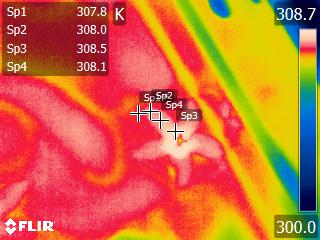

Supplement: Supplementary file 9 — Additional file 9. Water bath estimation IR images. File containing the thermal imaging (and paired photographs) of all images used in data collection for the water bath protocol. Images are sorted by species, set temperature of the water bath and then by individual flower, flower file names are formatted as [flower identifier used for sorting e.g. ‘D’][number]. Additionally, where available, photos are provided to aid identification of individual flowers. [file 13007_2021_721_MOESM9_ESM.zip › Bath IR images/campanula/35c/C12/IR_89520.jpg]

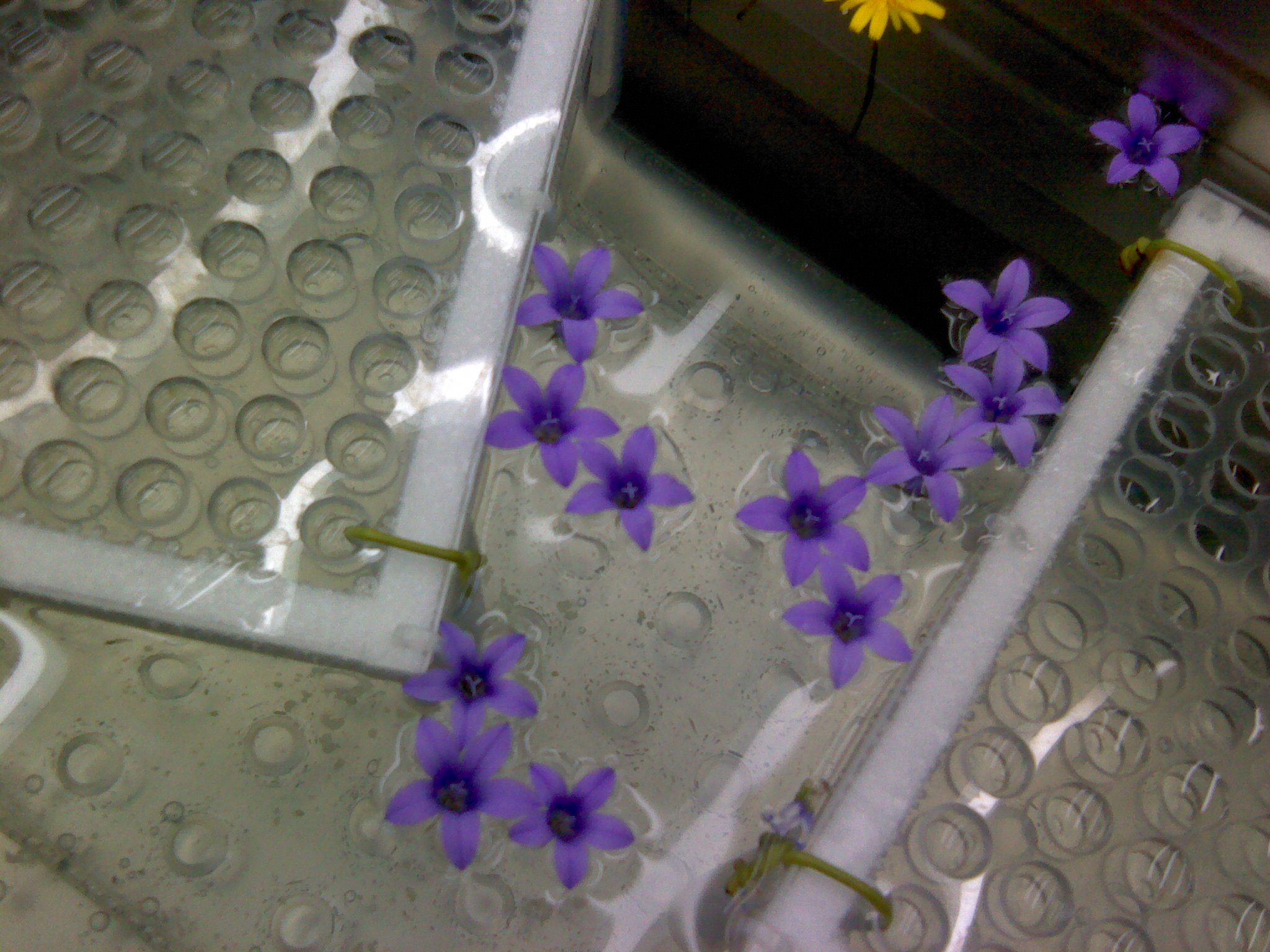

Supplement: Supplementary file 9 — Additional file 9. Water bath estimation IR images. File containing the thermal imaging (and paired photographs) of all images used in data collection for the water bath protocol. Images are sorted by species, set temperature of the water bath and then by individual flower, flower file names are formatted as [flower identifier used for sorting e.g. ‘D’][number]. Additionally, where available, photos are provided to aid identification of individual flowers. [file 13007_2021_721_MOESM9_ESM.zip › Bath IR images/campanula/35c/C2/DC_89499.jpg]

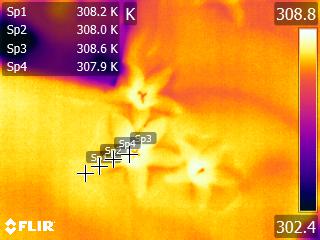

Supplement: Supplementary file 9 — Additional file 9. Water bath estimation IR images. File containing the thermal imaging (and paired photographs) of all images used in data collection for the water bath protocol. Images are sorted by species, set temperature of the water bath and then by individual flower, flower file names are formatted as [flower identifier used for sorting e.g. ‘D’][number]. Additionally, where available, photos are provided to aid identification of individual flowers. [file 13007_2021_721_MOESM9_ESM.zip › Bath IR images/campanula/35c/C2/IR_89498.jpg]

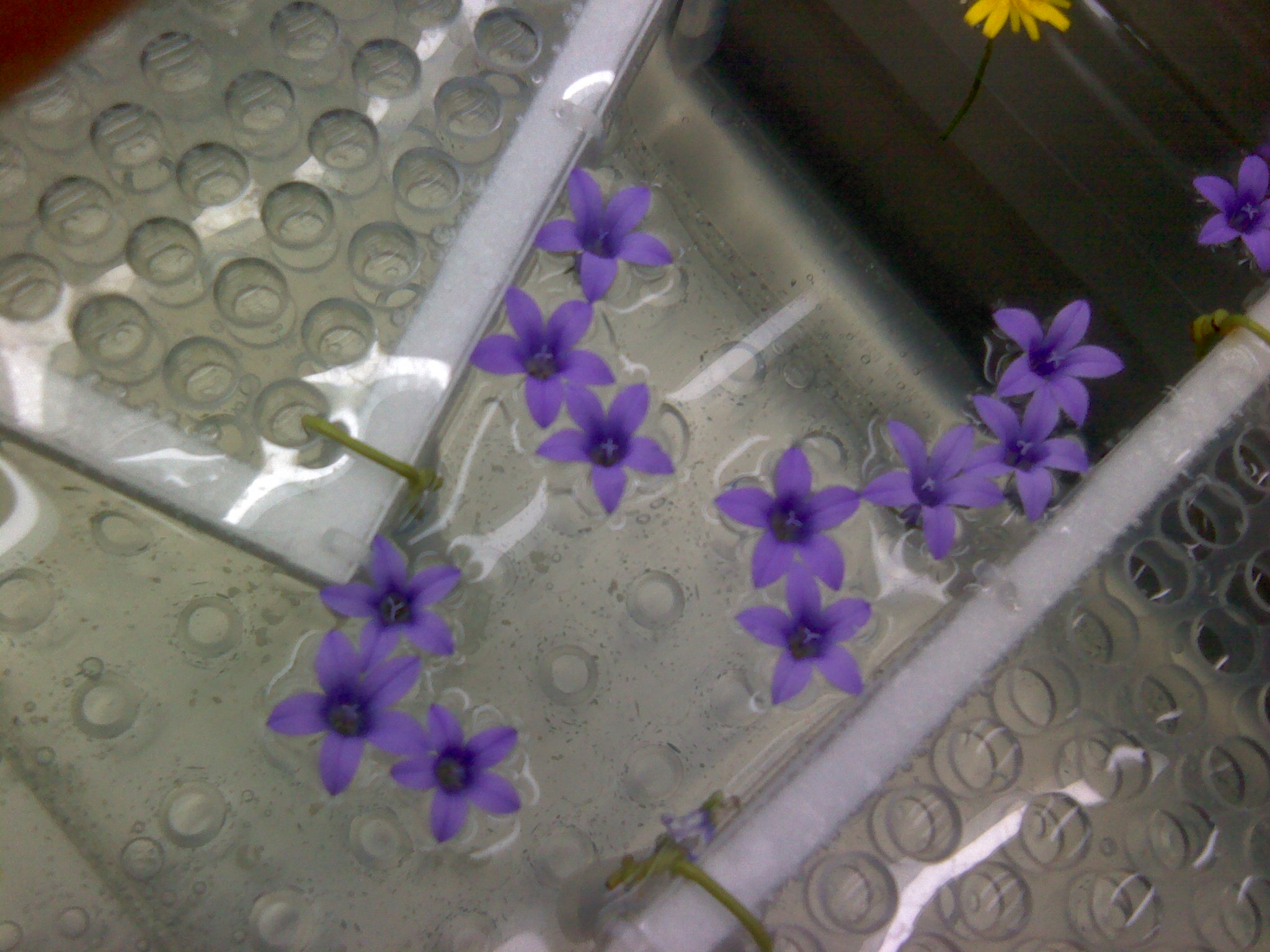

Supplement: Supplementary file 9 — Additional file 9. Water bath estimation IR images. File containing the thermal imaging (and paired photographs) of all images used in data collection for the water bath protocol. Images are sorted by species, set temperature of the water bath and then by individual flower, flower file names are formatted as [flower identifier used for sorting e.g. ‘D’][number]. Additionally, where available, photos are provided to aid identification of individual flowers. [file 13007_2021_721_MOESM9_ESM.zip › Bath IR images/campanula/35c/C3/DC_89501.jpg]

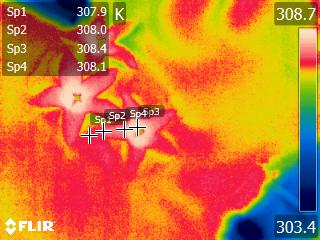

Supplement: Supplementary file 9 — Additional file 9. Water bath estimation IR images. File containing the thermal imaging (and paired photographs) of all images used in data collection for the water bath protocol. Images are sorted by species, set temperature of the water bath and then by individual flower, flower file names are formatted as [flower identifier used for sorting e.g. ‘D’][number]. Additionally, where available, photos are provided to aid identification of individual flowers. [file 13007_2021_721_MOESM9_ESM.zip › Bath IR images/campanula/35c/C3/IR_89500.jpg]

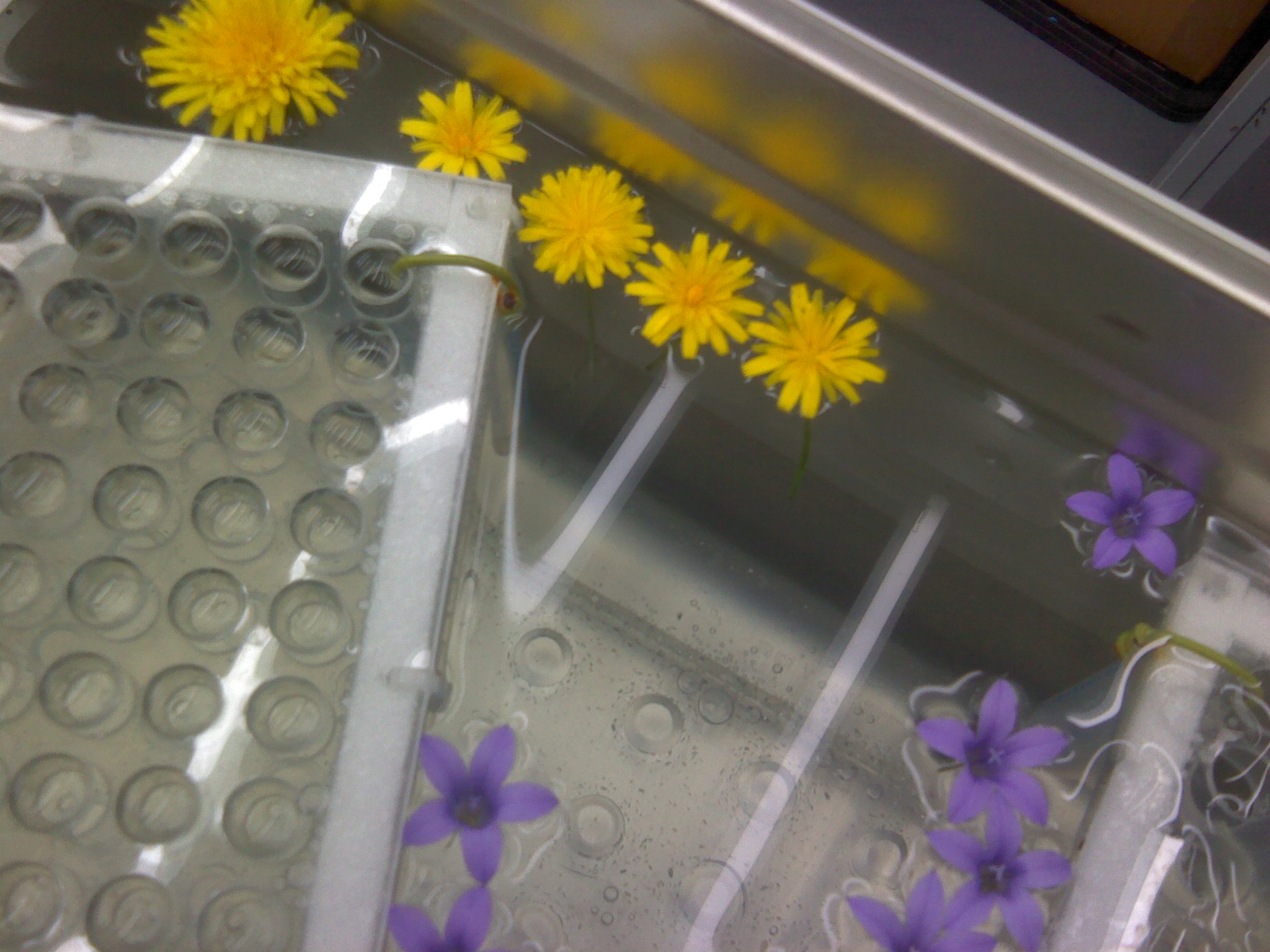

Supplement: Supplementary file 9 — Additional file 9. Water bath estimation IR images. File containing the thermal imaging (and paired photographs) of all images used in data collection for the water bath protocol. Images are sorted by species, set temperature of the water bath and then by individual flower, flower file names are formatted as [flower identifier used for sorting e.g. ‘D’][number]. Additionally, where available, photos are provided to aid identification of individual flowers. [file 13007_2021_721_MOESM9_ESM.zip › Bath IR images/campanula/35c/C4/DC_89503.jpg]

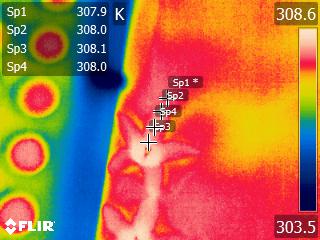

Supplement: Supplementary file 9 — Additional file 9. Water bath estimation IR images. File containing the thermal imaging (and paired photographs) of all images used in data collection for the water bath protocol. Images are sorted by species, set temperature of the water bath and then by individual flower, flower file names are formatted as [flower identifier used for sorting e.g. ‘D’][number]. Additionally, where available, photos are provided to aid identification of individual flowers. [file 13007_2021_721_MOESM9_ESM.zip › Bath IR images/campanula/35c/C4/IR_89502.jpg]

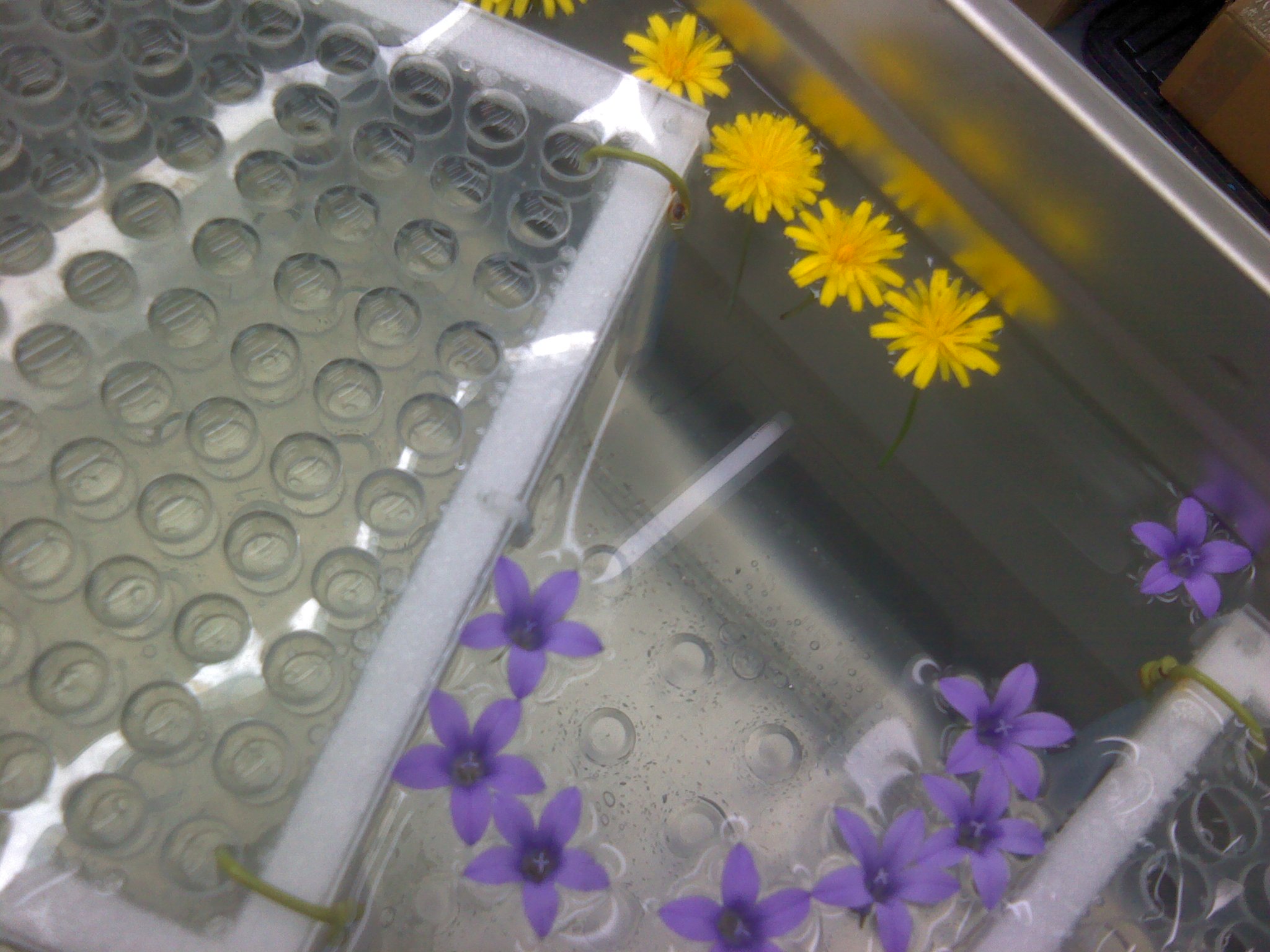

Supplement: Supplementary file 9 — Additional file 9. Water bath estimation IR images. File containing the thermal imaging (and paired photographs) of all images used in data collection for the water bath protocol. Images are sorted by species, set temperature of the water bath and then by individual flower, flower file names are formatted as [flower identifier used for sorting e.g. ‘D’][number]. Additionally, where available, photos are provided to aid identification of individual flowers. [file 13007_2021_721_MOESM9_ESM.zip › Bath IR images/campanula/35c/C5/DC_89505.jpg]

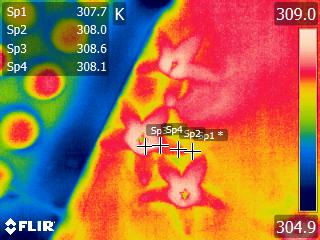

Supplement: Supplementary file 9 — Additional file 9. Water bath estimation IR images. File containing the thermal imaging (and paired photographs) of all images used in data collection for the water bath protocol. Images are sorted by species, set temperature of the water bath and then by individual flower, flower file names are formatted as [flower identifier used for sorting e.g. ‘D’][number]. Additionally, where available, photos are provided to aid identification of individual flowers. [file 13007_2021_721_MOESM9_ESM.zip › Bath IR images/campanula/35c/C5/IR_89504.jpg]

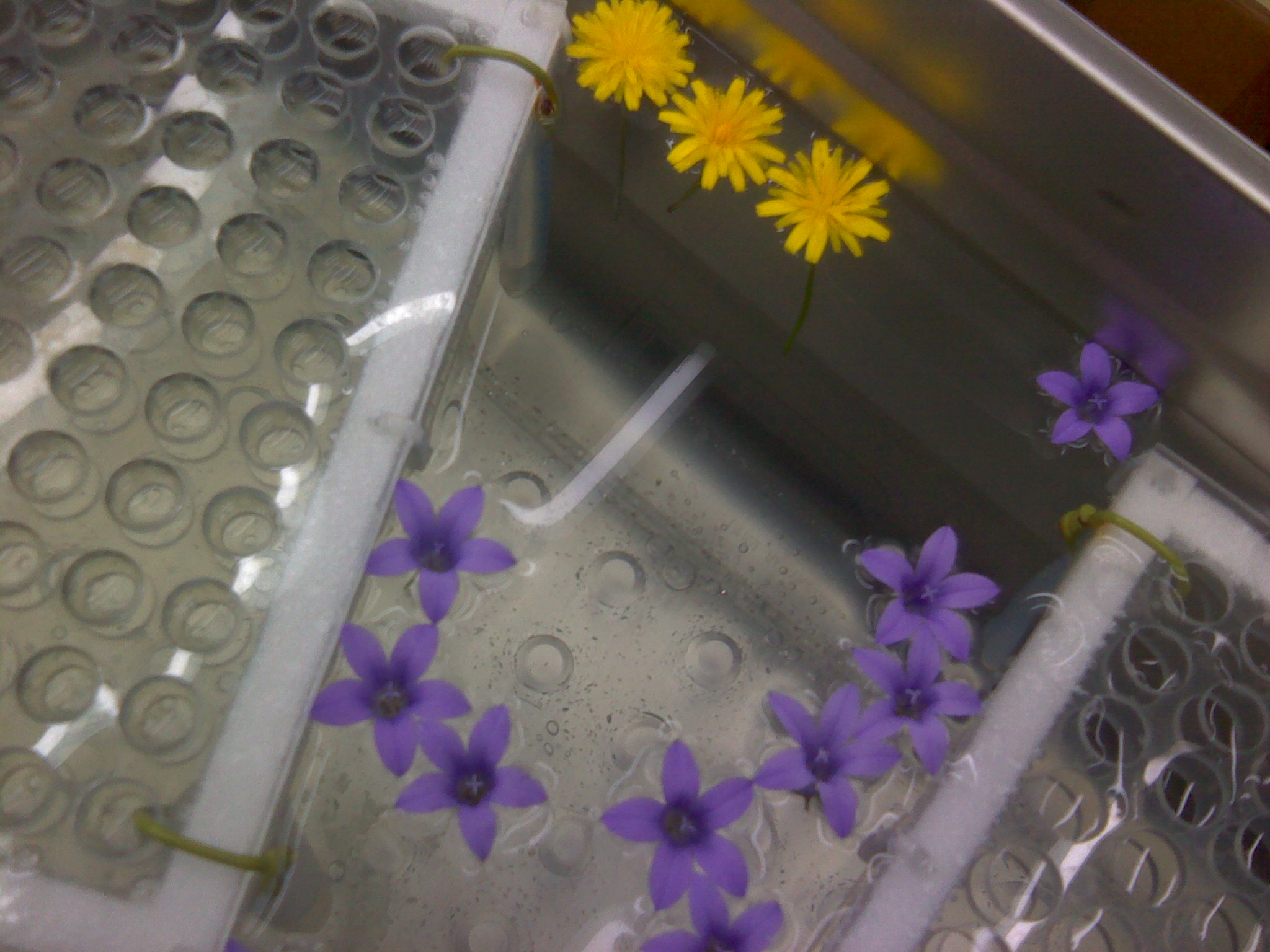

Supplement: Supplementary file 9 — Additional file 9. Water bath estimation IR images. File containing the thermal imaging (and paired photographs) of all images used in data collection for the water bath protocol. Images are sorted by species, set temperature of the water bath and then by individual flower, flower file names are formatted as [flower identifier used for sorting e.g. ‘D’][number]. Additionally, where available, photos are provided to aid identification of individual flowers. [file 13007_2021_721_MOESM9_ESM.zip › Bath IR images/campanula/35c/C6/DC_89507.jpg]

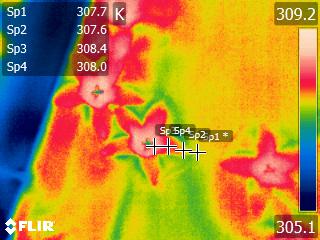

Supplement: Supplementary file 9 — Additional file 9. Water bath estimation IR images. File containing the thermal imaging (and paired photographs) of all images used in data collection for the water bath protocol. Images are sorted by species, set temperature of the water bath and then by individual flower, flower file names are formatted as [flower identifier used for sorting e.g. ‘D’][number]. Additionally, where available, photos are provided to aid identification of individual flowers. [file 13007_2021_721_MOESM9_ESM.zip › Bath IR images/campanula/35c/C6/IR_89506.jpg]

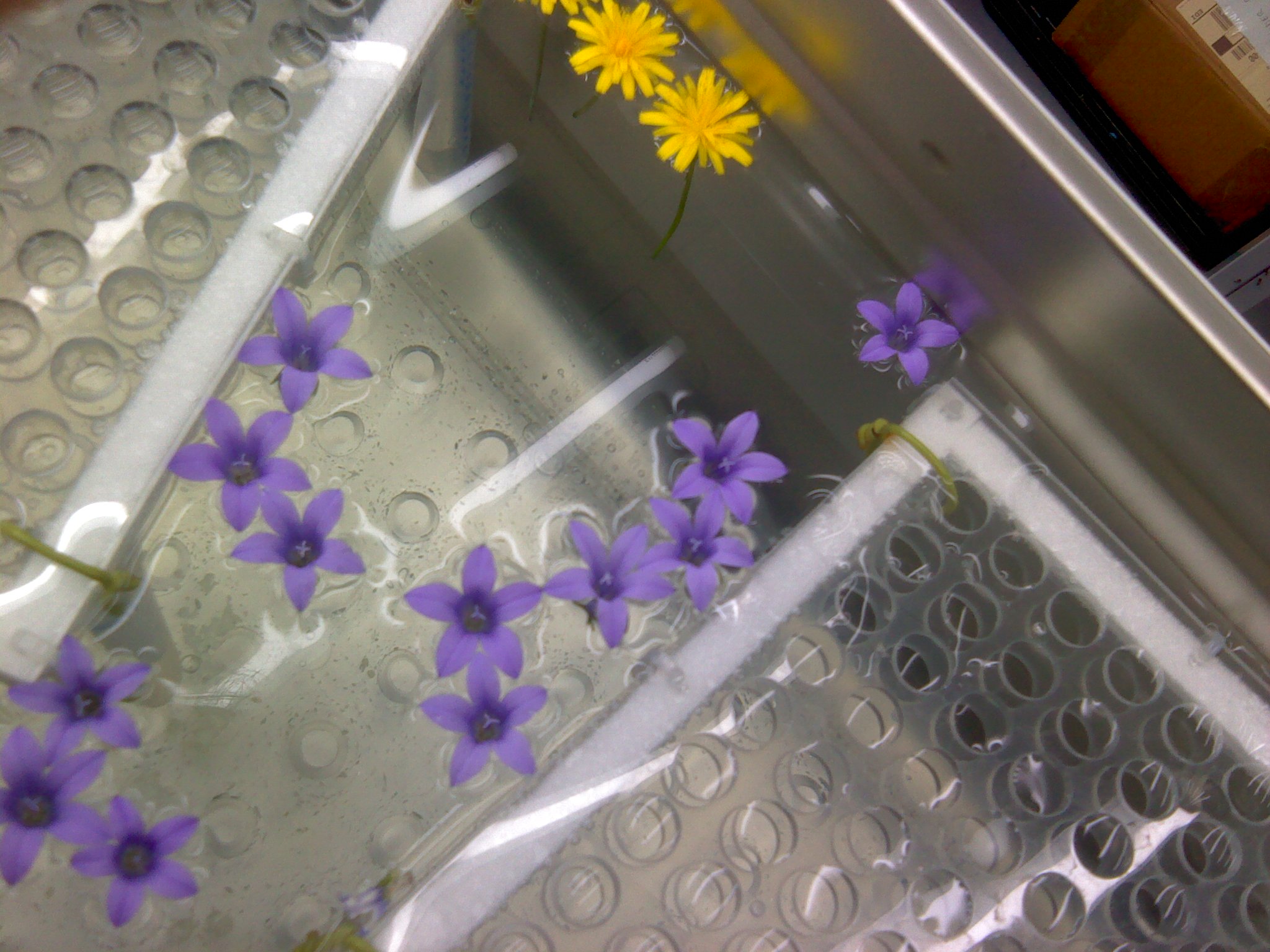

Supplement: Supplementary file 9 — Additional file 9. Water bath estimation IR images. File containing the thermal imaging (and paired photographs) of all images used in data collection for the water bath protocol. Images are sorted by species, set temperature of the water bath and then by individual flower, flower file names are formatted as [flower identifier used for sorting e.g. ‘D’][number]. Additionally, where available, photos are provided to aid identification of individual flowers. [file 13007_2021_721_MOESM9_ESM.zip › Bath IR images/campanula/35c/C7/DC_89511.jpg]

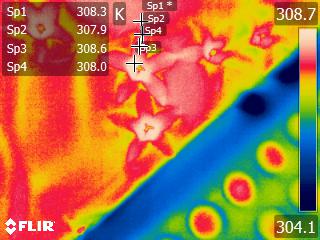

Supplement: Supplementary file 9 — Additional file 9. Water bath estimation IR images. File containing the thermal imaging (and paired photographs) of all images used in data collection for the water bath protocol. Images are sorted by species, set temperature of the water bath and then by individual flower, flower file names are formatted as [flower identifier used for sorting e.g. ‘D’][number]. Additionally, where available, photos are provided to aid identification of individual flowers. [file 13007_2021_721_MOESM9_ESM.zip › Bath IR images/campanula/35c/C7/IR_89510.jpg]

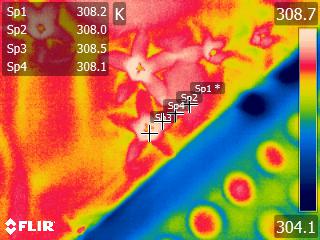

Supplement: Supplementary file 9 — Additional file 9. Water bath estimation IR images. File containing the thermal imaging (and paired photographs) of all images used in data collection for the water bath protocol. Images are sorted by species, set temperature of the water bath and then by individual flower, flower file names are formatted as [flower identifier used for sorting e.g. ‘D’][number]. Additionally, where available, photos are provided to aid identification of individual flowers. [file 13007_2021_721_MOESM9_ESM.zip › Bath IR images/campanula/35c/C8/IR_89510.jpg]

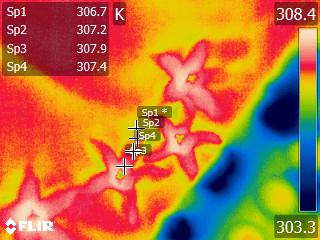

Supplement: Supplementary file 9 — Additional file 9. Water bath estimation IR images. File containing the thermal imaging (and paired photographs) of all images used in data collection for the water bath protocol. Images are sorted by species, set temperature of the water bath and then by individual flower, flower file names are formatted as [flower identifier used for sorting e.g. ‘D’][number]. Additionally, where available, photos are provided to aid identification of individual flowers. [file 13007_2021_721_MOESM9_ESM.zip › Bath IR images/campanula/35c/C9/IR_89514.jpg]

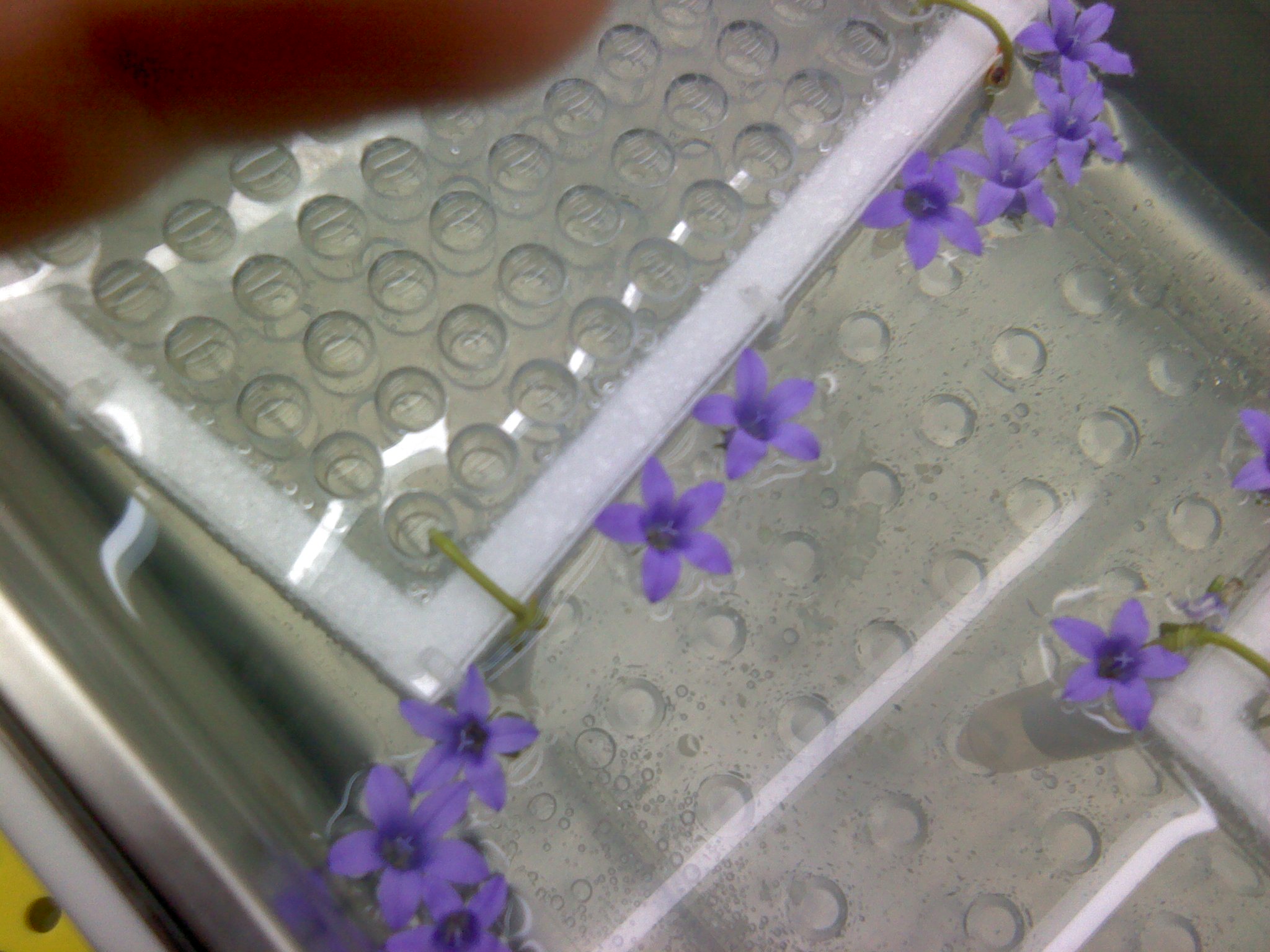

Supplement: Supplementary file 9 — Additional file 9. Water bath estimation IR images. File containing the thermal imaging (and paired photographs) of all images used in data collection for the water bath protocol. Images are sorted by species, set temperature of the water bath and then by individual flower, flower file names are formatted as [flower identifier used for sorting e.g. ‘D’][number]. Additionally, where available, photos are provided to aid identification of individual flowers. [file 13007_2021_721_MOESM9_ESM.zip › Bath IR images/campanula/45c/C1/DC_89569.jpg]

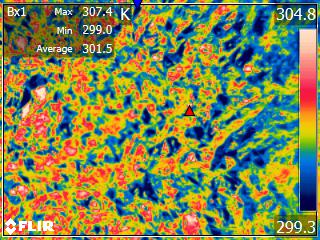

Supplement: Supplementary file 9 — Additional file 9. Water bath estimation IR images. File containing the thermal imaging (and paired photographs) of all images used in data collection for the water bath protocol. Images are sorted by species, set temperature of the water bath and then by individual flower, flower file names are formatted as [flower identifier used for sorting e.g. ‘D’][number]. Additionally, where available, photos are provided to aid identification of individual flowers. [file 13007_2021_721_MOESM9_ESM.zip › Bath IR images/campanula/45c/C1/IR_89546.jpg]

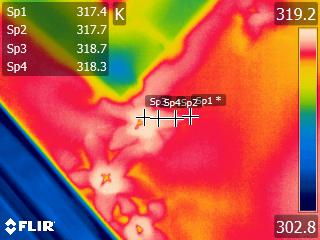

Supplement: Supplementary file 9 — Additional file 9. Water bath estimation IR images. File containing the thermal imaging (and paired photographs) of all images used in data collection for the water bath protocol. Images are sorted by species, set temperature of the water bath and then by individual flower, flower file names are formatted as [flower identifier used for sorting e.g. ‘D’][number]. Additionally, where available, photos are provided to aid identification of individual flowers. [file 13007_2021_721_MOESM9_ESM.zip › Bath IR images/campanula/45c/C1/IR_89568.jpg]

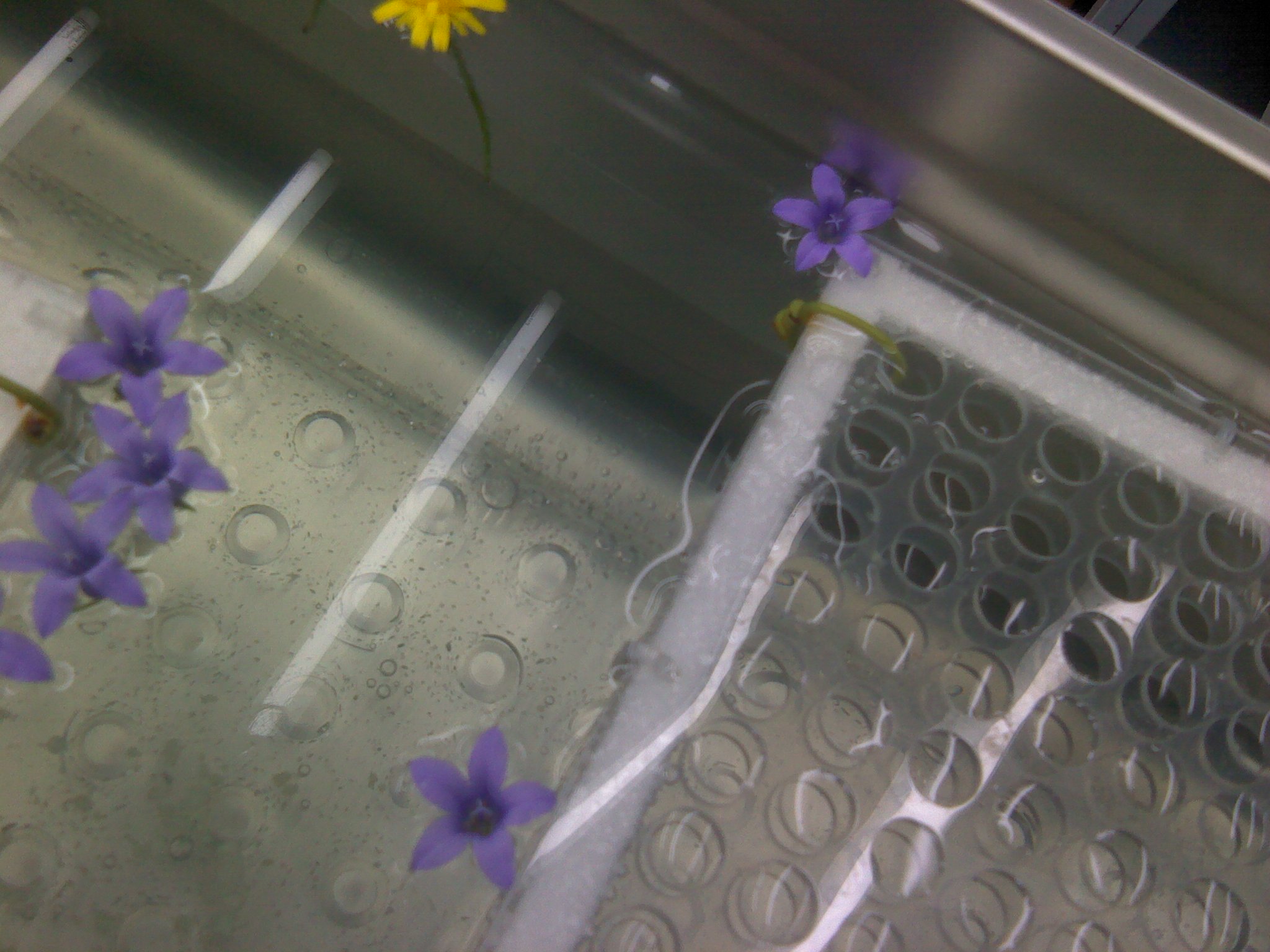

Supplement: Supplementary file 9 — Additional file 9. Water bath estimation IR images. File containing the thermal imaging (and paired photographs) of all images used in data collection for the water bath protocol. Images are sorted by species, set temperature of the water bath and then by individual flower, flower file names are formatted as [flower identifier used for sorting e.g. ‘D’][number]. Additionally, where available, photos are provided to aid identification of individual flowers. [file 13007_2021_721_MOESM9_ESM.zip › Bath IR images/campanula/45c/C10/DC_89589.jpg]

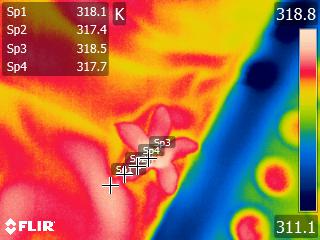

Supplement: Supplementary file 9 — Additional file 9. Water bath estimation IR images. File containing the thermal imaging (and paired photographs) of all images used in data collection for the water bath protocol. Images are sorted by species, set temperature of the water bath and then by individual flower, flower file names are formatted as [flower identifier used for sorting e.g. ‘D’][number]. Additionally, where available, photos are provided to aid identification of individual flowers. [file 13007_2021_721_MOESM9_ESM.zip › Bath IR images/campanula/45c/C10/IR_89588.jpg]

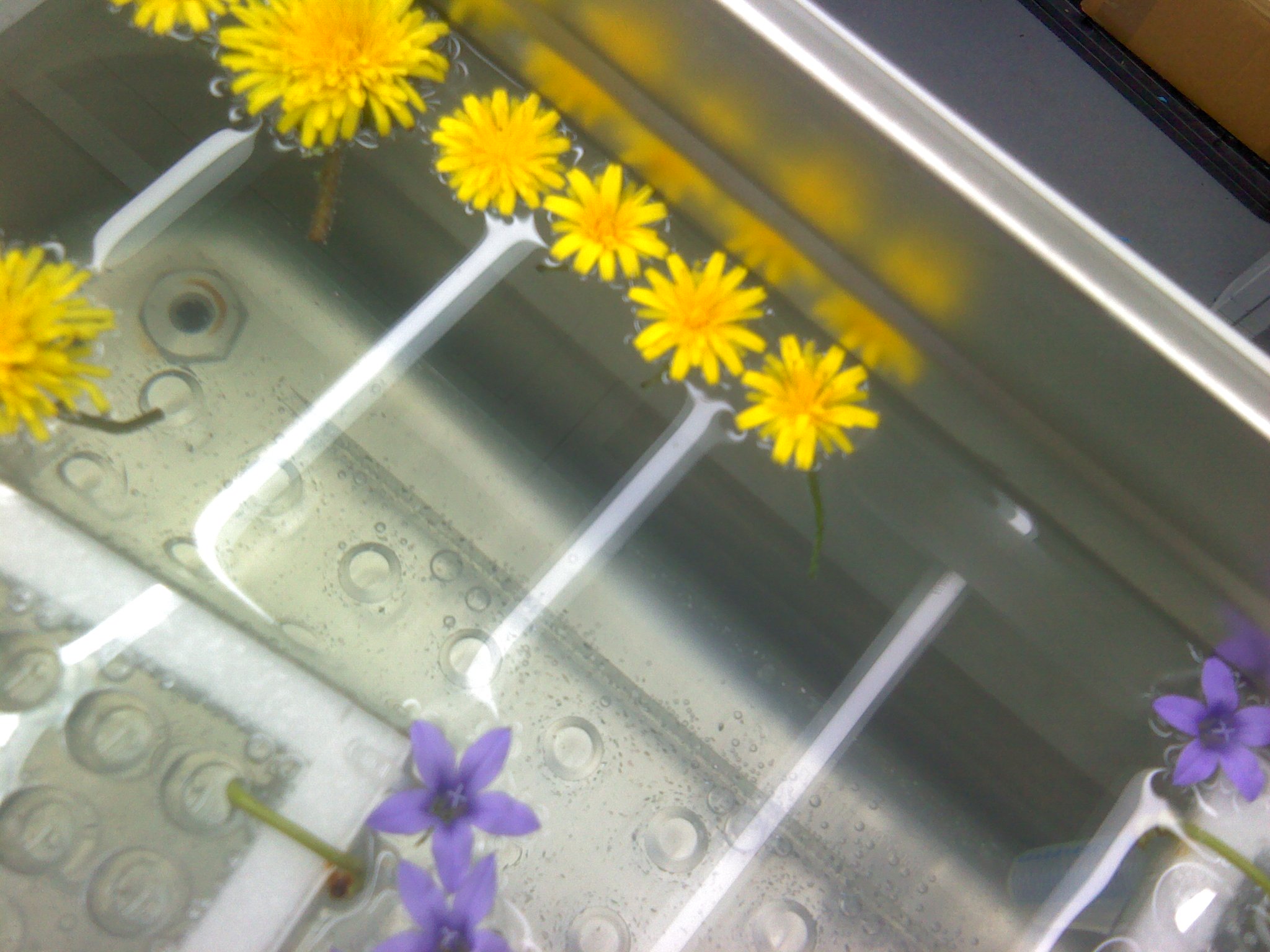

Supplement: Supplementary file 9 — Additional file 9. Water bath estimation IR images. File containing the thermal imaging (and paired photographs) of all images used in data collection for the water bath protocol. Images are sorted by species, set temperature of the water bath and then by individual flower, flower file names are formatted as [flower identifier used for sorting e.g. ‘D’][number]. Additionally, where available, photos are provided to aid identification of individual flowers. [file 13007_2021_721_MOESM9_ESM.zip › Bath IR images/campanula/45c/C11/DC_89585.jpg]

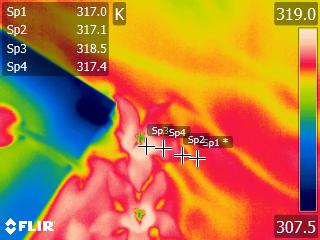

Supplement: Supplementary file 9 — Additional file 9. Water bath estimation IR images. File containing the thermal imaging (and paired photographs) of all images used in data collection for the water bath protocol. Images are sorted by species, set temperature of the water bath and then by individual flower, flower file names are formatted as [flower identifier used for sorting e.g. ‘D’][number]. Additionally, where available, photos are provided to aid identification of individual flowers. [file 13007_2021_721_MOESM9_ESM.zip › Bath IR images/campanula/45c/C11/IR_89584.jpg]

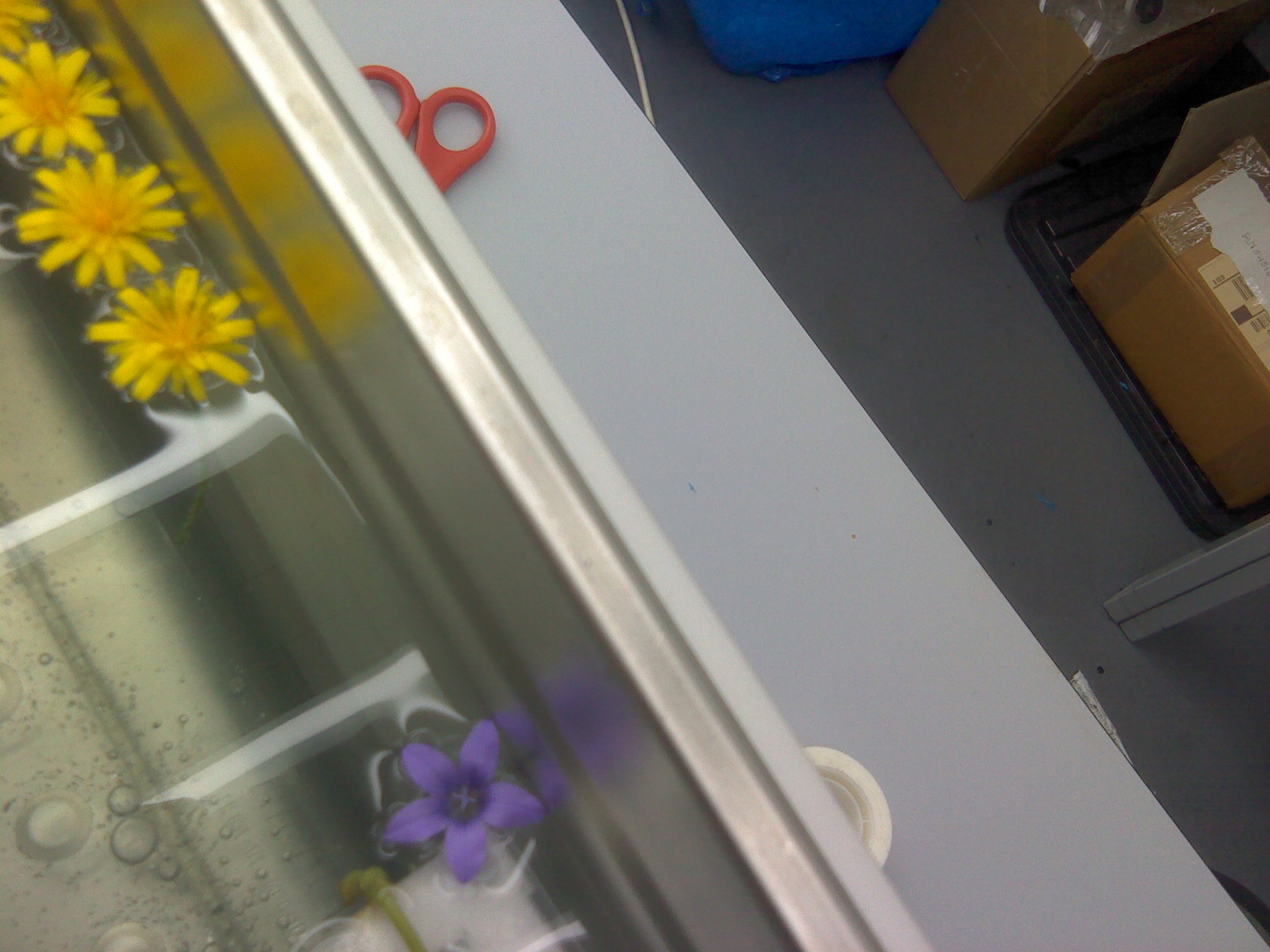

Supplement: Supplementary file 9 — Additional file 9. Water bath estimation IR images. File containing the thermal imaging (and paired photographs) of all images used in data collection for the water bath protocol. Images are sorted by species, set temperature of the water bath and then by individual flower, flower file names are formatted as [flower identifier used for sorting e.g. ‘D’][number]. Additionally, where available, photos are provided to aid identification of individual flowers. [file 13007_2021_721_MOESM9_ESM.zip › Bath IR images/campanula/45c/C12/DC_89591.jpg]

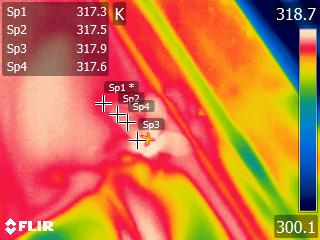

Supplement: Supplementary file 9 — Additional file 9. Water bath estimation IR images. File containing the thermal imaging (and paired photographs) of all images used in data collection for the water bath protocol. Images are sorted by species, set temperature of the water bath and then by individual flower, flower file names are formatted as [flower identifier used for sorting e.g. ‘D’][number]. Additionally, where available, photos are provided to aid identification of individual flowers. [file 13007_2021_721_MOESM9_ESM.zip › Bath IR images/campanula/45c/C12/IR_89590.jpg]

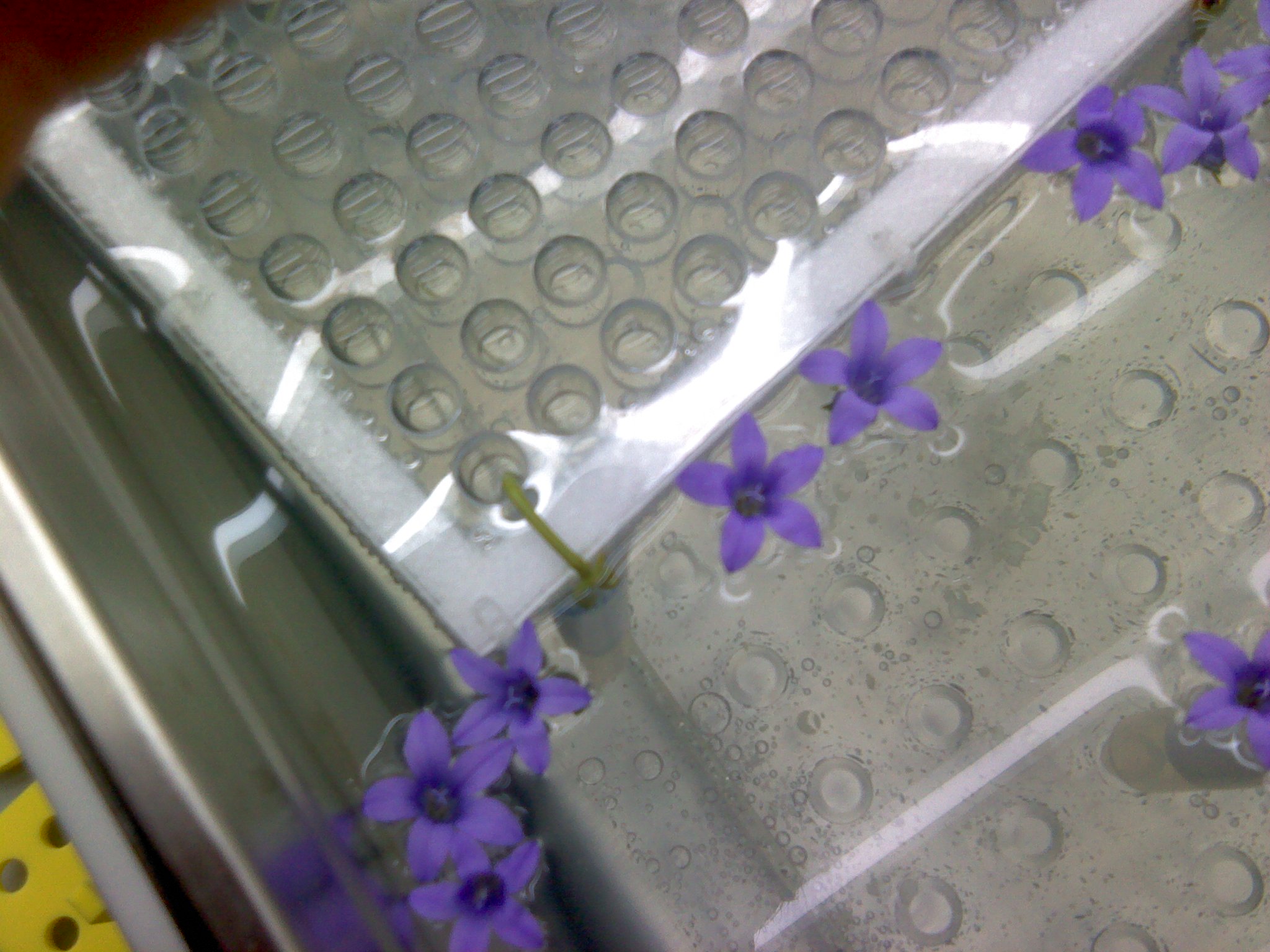

Supplement: Supplementary file 9 — Additional file 9. Water bath estimation IR images. File containing the thermal imaging (and paired photographs) of all images used in data collection for the water bath protocol. Images are sorted by species, set temperature of the water bath and then by individual flower, flower file names are formatted as [flower identifier used for sorting e.g. ‘D’][number]. Additionally, where available, photos are provided to aid identification of individual flowers. [file 13007_2021_721_MOESM9_ESM.zip › Bath IR images/campanula/45c/C2/DC_89571.jpg]

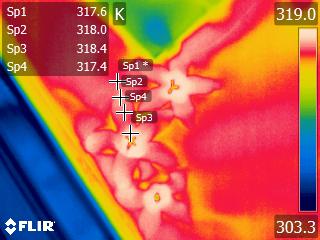

Supplement: Supplementary file 9 — Additional file 9. Water bath estimation IR images. File containing the thermal imaging (and paired photographs) of all images used in data collection for the water bath protocol. Images are sorted by species, set temperature of the water bath and then by individual flower, flower file names are formatted as [flower identifier used for sorting e.g. ‘D’][number]. Additionally, where available, photos are provided to aid identification of individual flowers. [file 13007_2021_721_MOESM9_ESM.zip › Bath IR images/campanula/45c/C2/IR_89570.jpg]

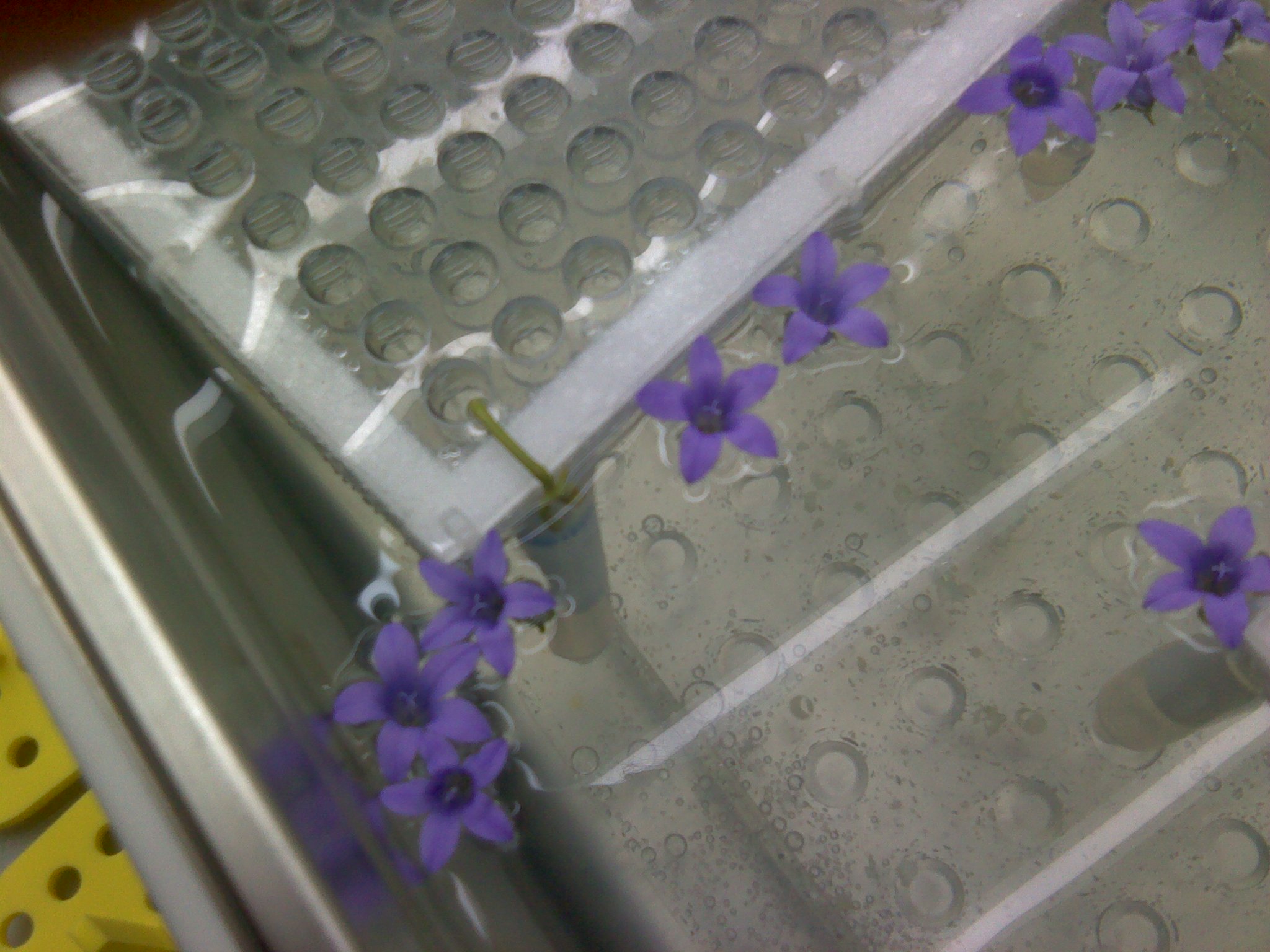

Supplement: Supplementary file 9 — Additional file 9. Water bath estimation IR images. File containing the thermal imaging (and paired photographs) of all images used in data collection for the water bath protocol. Images are sorted by species, set temperature of the water bath and then by individual flower, flower file names are formatted as [flower identifier used for sorting e.g. ‘D’][number]. Additionally, where available, photos are provided to aid identification of individual flowers. [file 13007_2021_721_MOESM9_ESM.zip › Bath IR images/campanula/45c/C3/DC_89573.jpg]

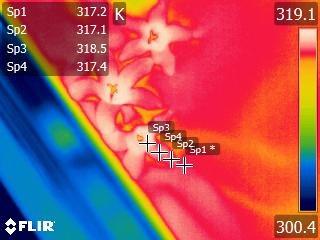

Supplement: Supplementary file 9 — Additional file 9. Water bath estimation IR images. File containing the thermal imaging (and paired photographs) of all images used in data collection for the water bath protocol. Images are sorted by species, set temperature of the water bath and then by individual flower, flower file names are formatted as [flower identifier used for sorting e.g. ‘D’][number]. Additionally, where available, photos are provided to aid identification of individual flowers. [file 13007_2021_721_MOESM9_ESM.zip › Bath IR images/campanula/45c/C3/IR_89572.jpg]

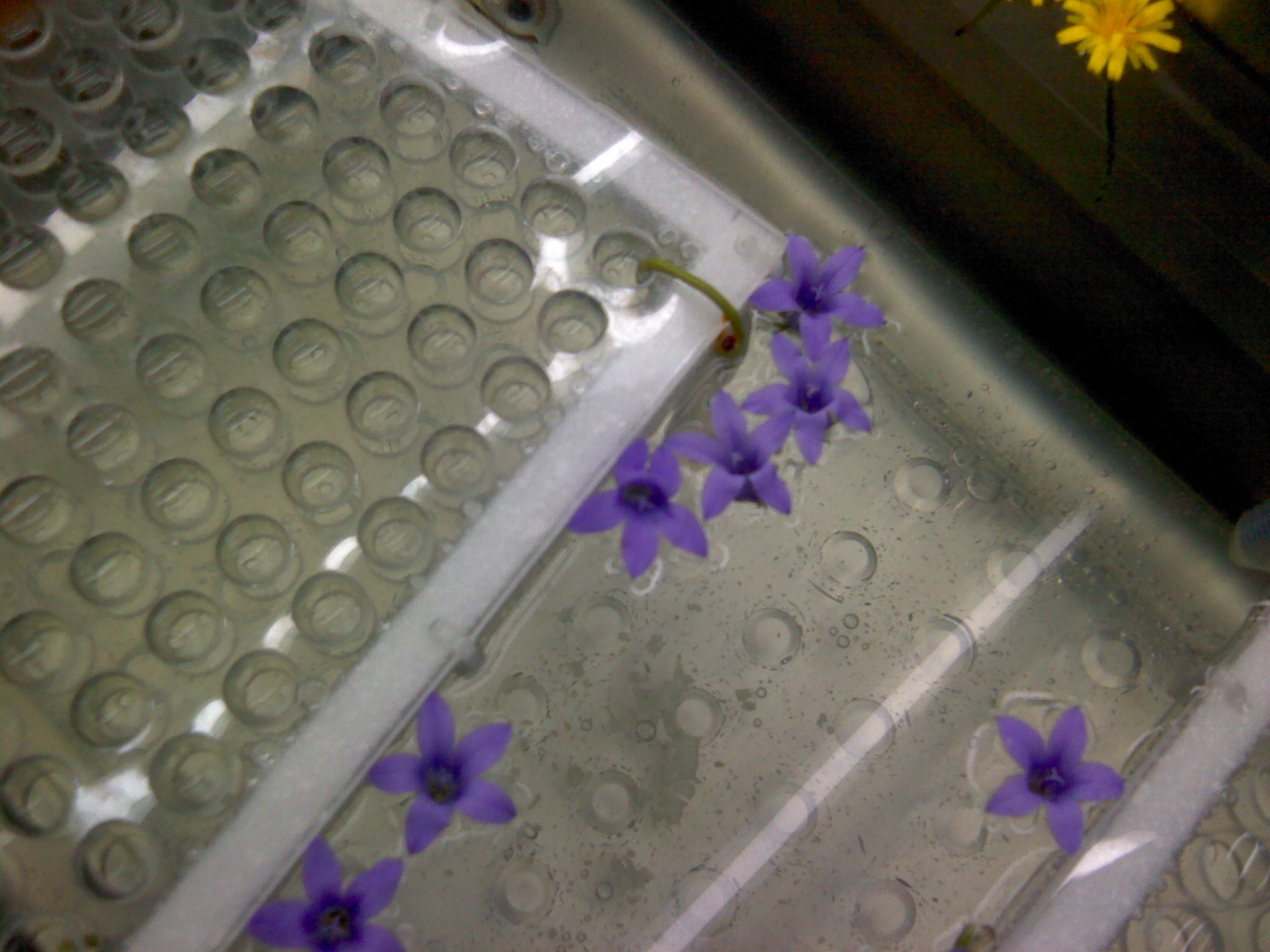

Supplement: Supplementary file 9 — Additional file 9. Water bath estimation IR images. File containing the thermal imaging (and paired photographs) of all images used in data collection for the water bath protocol. Images are sorted by species, set temperature of the water bath and then by individual flower, flower file names are formatted as [flower identifier used for sorting e.g. ‘D’][number]. Additionally, where available, photos are provided to aid identification of individual flowers. [file 13007_2021_721_MOESM9_ESM.zip › Bath IR images/campanula/45c/C4/DC_89577.jpg]

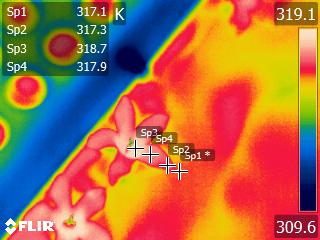

Supplement: Supplementary file 9 — Additional file 9. Water bath estimation IR images. File containing the thermal imaging (and paired photographs) of all images used in data collection for the water bath protocol. Images are sorted by species, set temperature of the water bath and then by individual flower, flower file names are formatted as [flower identifier used for sorting e.g. ‘D’][number]. Additionally, where available, photos are provided to aid identification of individual flowers. [file 13007_2021_721_MOESM9_ESM.zip › Bath IR images/campanula/45c/C4/IR_89576.jpg]

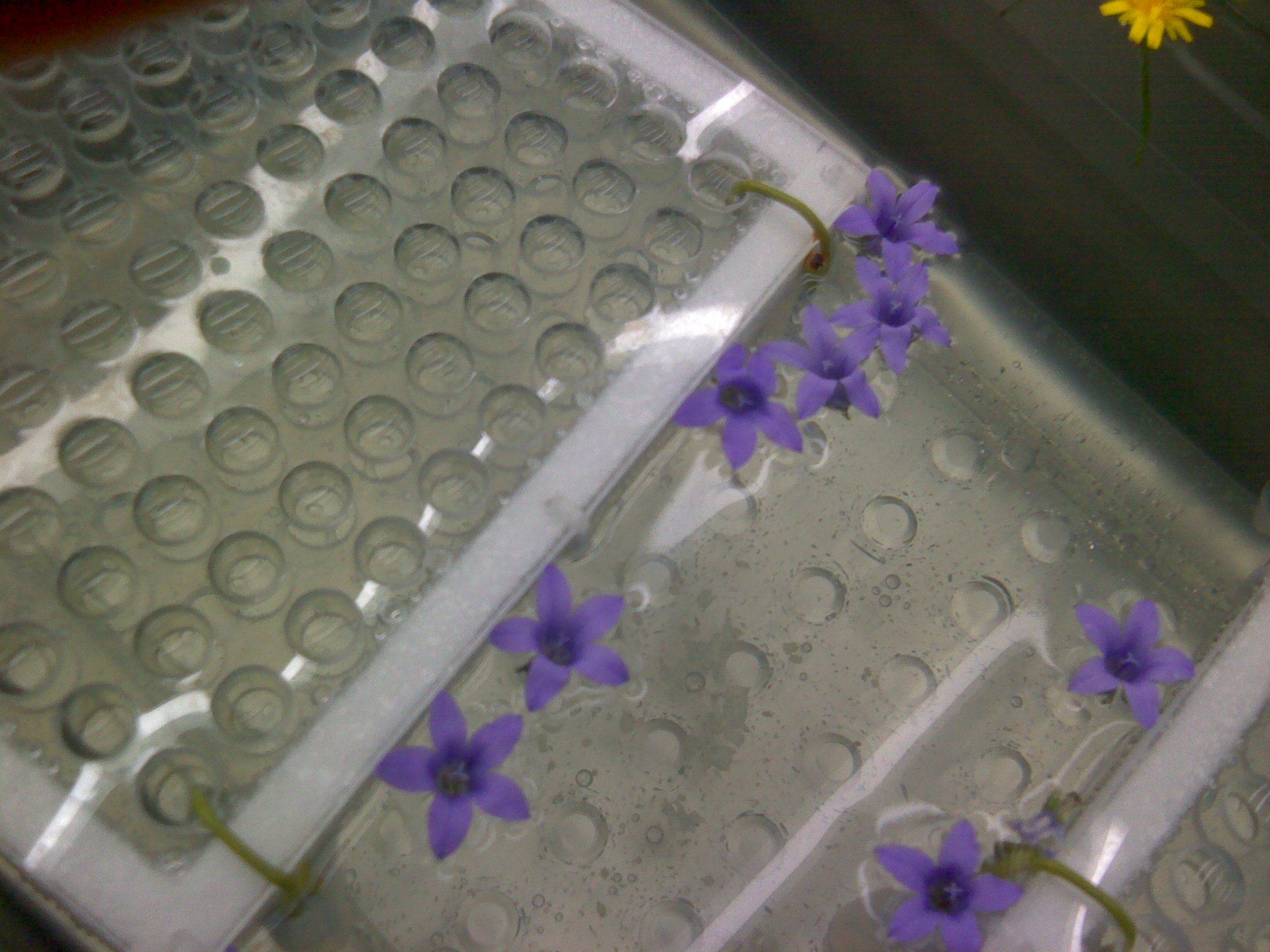

Supplement: Supplementary file 9 — Additional file 9. Water bath estimation IR images. File containing the thermal imaging (and paired photographs) of all images used in data collection for the water bath protocol. Images are sorted by species, set temperature of the water bath and then by individual flower, flower file names are formatted as [flower identifier used for sorting e.g. ‘D’][number]. Additionally, where available, photos are provided to aid identification of individual flowers. [file 13007_2021_721_MOESM9_ESM.zip › Bath IR images/campanula/45c/C5/DC_89575.jpg]

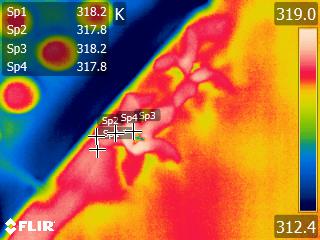

Supplement: Supplementary file 9 — Additional file 9. Water bath estimation IR images. File containing the thermal imaging (and paired photographs) of all images used in data collection for the water bath protocol. Images are sorted by species, set temperature of the water bath and then by individual flower, flower file names are formatted as [flower identifier used for sorting e.g. ‘D’][number]. Additionally, where available, photos are provided to aid identification of individual flowers. [file 13007_2021_721_MOESM9_ESM.zip › Bath IR images/campanula/45c/C5/IR_89574.jpg]

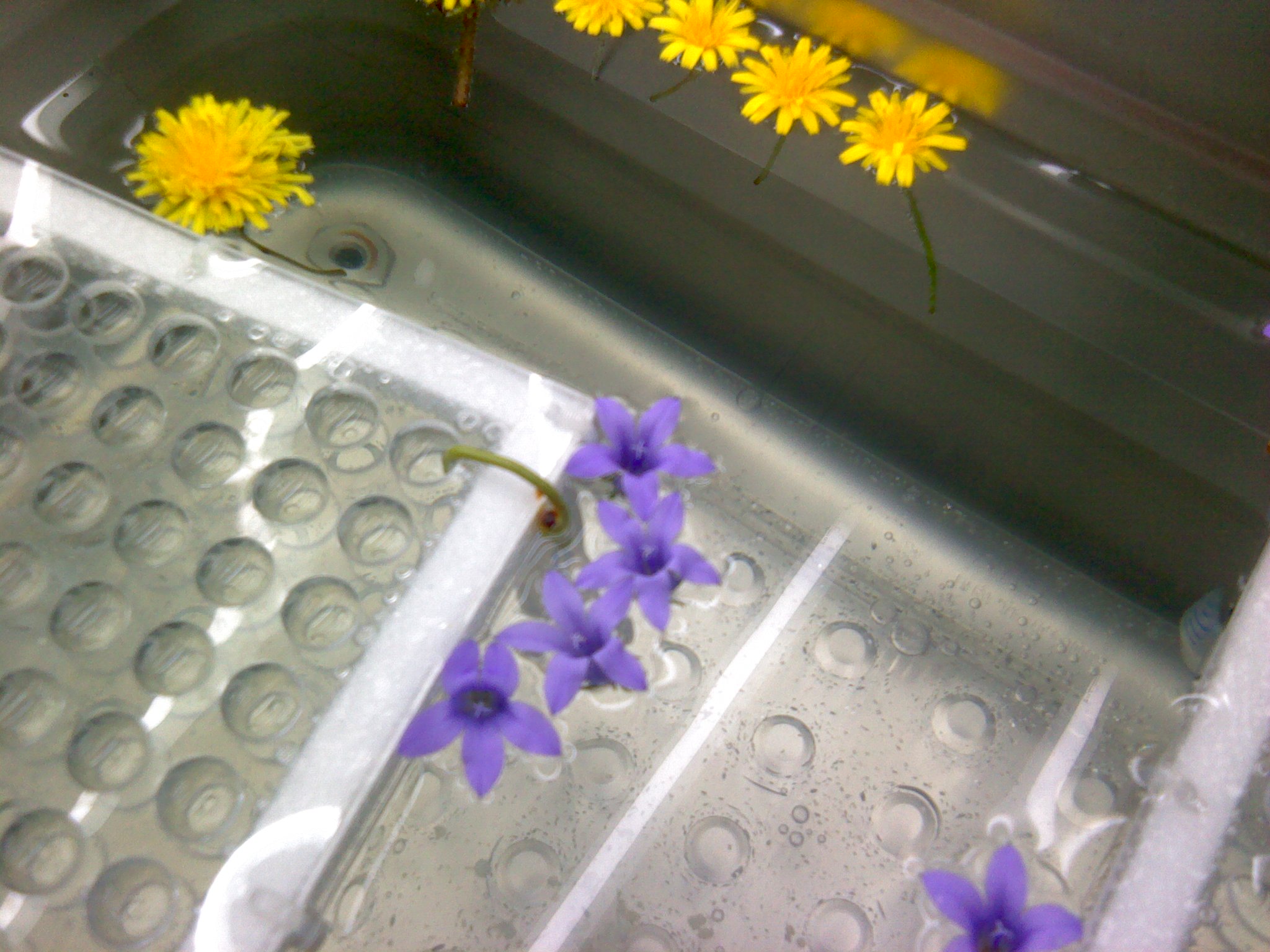

Supplement: Supplementary file 9 — Additional file 9. Water bath estimation IR images. File containing the thermal imaging (and paired photographs) of all images used in data collection for the water bath protocol. Images are sorted by species, set temperature of the water bath and then by individual flower, flower file names are formatted as [flower identifier used for sorting e.g. ‘D’][number]. Additionally, where available, photos are provided to aid identification of individual flowers. [file 13007_2021_721_MOESM9_ESM.zip › Bath IR images/campanula/45c/C6/DC_89579.jpg]

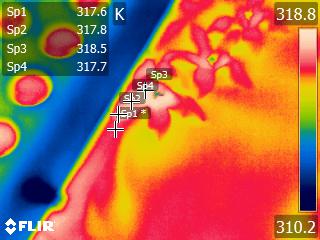

Supplement: Supplementary file 9 — Additional file 9. Water bath estimation IR images. File containing the thermal imaging (and paired photographs) of all images used in data collection for the water bath protocol. Images are sorted by species, set temperature of the water bath and then by individual flower, flower file names are formatted as [flower identifier used for sorting e.g. ‘D’][number]. Additionally, where available, photos are provided to aid identification of individual flowers. [file 13007_2021_721_MOESM9_ESM.zip › Bath IR images/campanula/45c/C6/IR_89578.jpg]

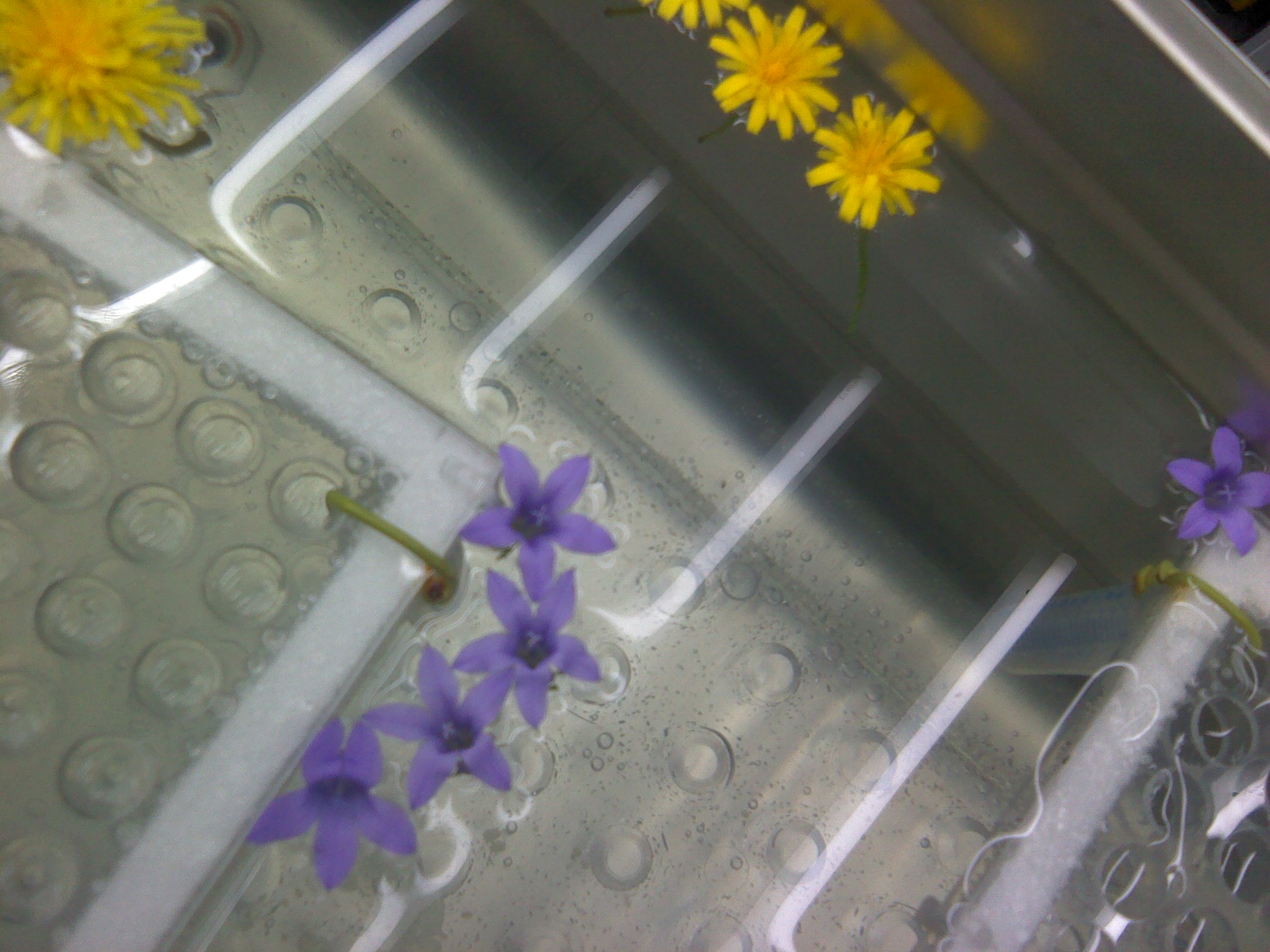

Supplement: Supplementary file 9 — Additional file 9. Water bath estimation IR images. File containing the thermal imaging (and paired photographs) of all images used in data collection for the water bath protocol. Images are sorted by species, set temperature of the water bath and then by individual flower, flower file names are formatted as [flower identifier used for sorting e.g. ‘D’][number]. Additionally, where available, photos are provided to aid identification of individual flowers. [file 13007_2021_721_MOESM9_ESM.zip › Bath IR images/campanula/45c/C7/DC_89581.jpg]

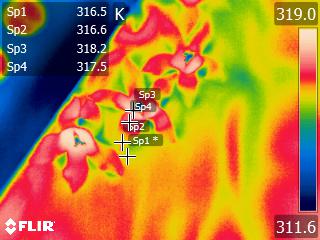

Supplement: Supplementary file 9 — Additional file 9. Water bath estimation IR images. File containing the thermal imaging (and paired photographs) of all images used in data collection for the water bath protocol. Images are sorted by species, set temperature of the water bath and then by individual flower, flower file names are formatted as [flower identifier used for sorting e.g. ‘D’][number]. Additionally, where available, photos are provided to aid identification of individual flowers. [file 13007_2021_721_MOESM9_ESM.zip › Bath IR images/campanula/45c/C7/IR_89580.jpg]

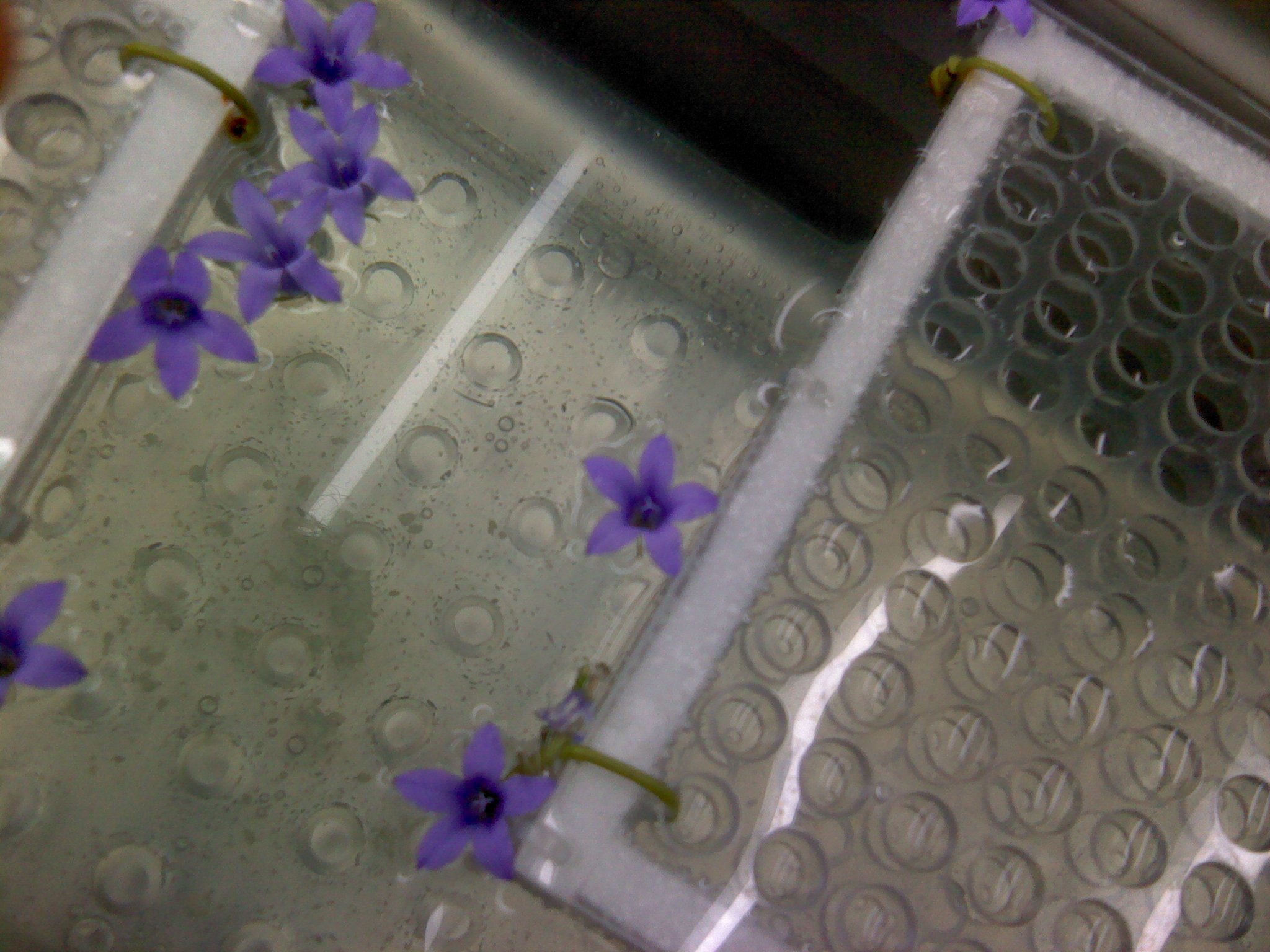

Supplement: Supplementary file 9 — Additional file 9. Water bath estimation IR images. File containing the thermal imaging (and paired photographs) of all images used in data collection for the water bath protocol. Images are sorted by species, set temperature of the water bath and then by individual flower, flower file names are formatted as [flower identifier used for sorting e.g. ‘D’][number]. Additionally, where available, photos are provided to aid identification of individual flowers. [file 13007_2021_721_MOESM9_ESM.zip › Bath IR images/campanula/45c/C8/DC_89587.jpg]

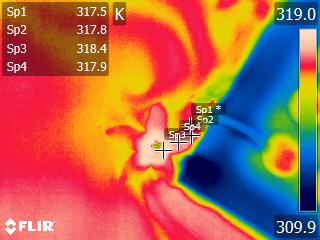

Supplement: Supplementary file 9 — Additional file 9. Water bath estimation IR images. File containing the thermal imaging (and paired photographs) of all images used in data collection for the water bath protocol. Images are sorted by species, set temperature of the water bath and then by individual flower, flower file names are formatted as [flower identifier used for sorting e.g. ‘D’][number]. Additionally, where available, photos are provided to aid identification of individual flowers. [file 13007_2021_721_MOESM9_ESM.zip › Bath IR images/campanula/45c/C8/IR_89586.jpg]
